# Supplementary material for: Predicting the structural basis of targeted protein degradation by integrating molecular dynamics simulations with structural mass spectrometry
Source: Nat Commun. 2022 Oct 6;13:5884. doi: 10.1038/s41467-022-33575-4 (PMC9537307; doi:10.1038/s41467-022-33575-4)
Supplement: Supplementary file 1 — Supplementary Information [file 41467_2022_33575_MOESM1_ESM.pdf]

# Predicting the structural basis of targeted protein degradation by integrating molecular dynamics simulations with structural mass spectrometry - Supplementary Information

Tom Dixon,[†,‡,¶,§] Derek MacPherson,[†,§] Barmak Mostofian,[†,§] Taras Dauzhenka,[†] Samuel Lotz,[†] Dwight McGee,[†] Sharon Shechter,[†] Utsab R. Shrestha,[†] Rafal Wiewiora,[†] Zachary A. McDargh,[†] Fen Pei,[†] Rajat Pal,[†] João V. Ribeiro,[†] Tanner Wilkerson,[†] Vipin Sachdeva,[†] Ning Gao,[†] Shourya Jain,[†] Samuel Sparks,[†] Yunxing Li,[†] Alexander Vinitsky,[†] Xin Zhang,[†] Asghar M. Razavi,[†] István Kolossváry,[†] Jason Imbriglio,[†] Artem Evdokimov,[†] Louise Bergeron,[†] Wenchang Zhou,[†] Jagat Adhikari,[†] Benjamin Ruprecht,[†] Alex Dickson,[*,‡,¶] Huafeng Xu,[*,†] Woody Sherman,[*,†] and Jesus A. Izaguirre[*,†]

†*Roivant Discovery, New York City, New York, 10036, USA and Boston, Massachusetts, 02210, USA*

‡*Department of Computational Mathematics, Science, and Engineering, Michigan State University, East Lansing, Michigan, 48824, USA*

¶*Department of Biochemistry and Molecular Biology, Michigan State University, East Lansing, Michigan, 48824, USA*

§*These authors contributed equally*

E-mail: magentaalexrd@msu.edu; magentahuafeng.xu@roivant.com; magentawoody.sherman@roivant.com; magentajesus.izaguirre@roivant.com

# Supplementary Note 1

## WE-HDX simulations

The weighted-ensemble (WE) strategy enhances the sampling of rare events by running in parallel multiple simulations with well-defined probabilities. The periodic pruning and replication of trajectories allows progress to be made along a collective variable in conformational space, either by simulating trajectories in pre-defined regions (binned method) or by optimizing an objective function (bin-less method). For the formation of degrader ternary complexes, we have applied both WE simulation variants. Mainly, we run the bin-less REVO simulations[1] that maximize an objective function called the trajectory variation, defined as the sum of distances between individual trajectories. The distance metric itself is based on observables such as the number of atomic contacts or the warhead-RMSD (w-RMSD) with respect to the crystal structure of the target-warhead complex. Alternatively, in the binned WE method, we use these observables as collective variables to sample less-visited regions of conformational space.

Data from HDX-MS experiments are integrated with the WE simulations in a straightforward manner to facilitate the formation of contacts between the protected residues on SMARCA2$^{BD}$ and those on VHL as determined from HDX-MS (see Supplementary Table 8). This is achieved by replacing any SMARCA2$^{BD}$:VHL residue contact, in the (non-HDX) WE simulations, specifically with experimentally derived protected-residue contacts at the interface of the two binding partners, in the WE-HDX simulations. Detailed information on the WE-HDX simulations is provided in the Methods.

Supplementary Figure 3 reveals that both WE variants yield similar results in that the bulk of simulated SMARCA2$^{BD}$:PROTAC 2:VHL ternary complexes, in particular when guided by the HDX-MS data, have (minimum) interface-RMSDs (I-RMSDs) $< 4$ Å. The inclusion of the HDX-MS data, i.e., in WE-HDX and Docking-HDX, yields distributions of tighter bound ternary complexes as they are shifted toward smaller minimum I-RMSD

values – in particular for the WE-HDX simulations. Although the (non-HDX) WE simulations can sporadically produce SMARCA2$^{BD}$:PROTAC 2:VHL ternary complexes with a minimum I-RMSD that is even $< 0.5$ Å (see red profiles in Supplementary Figures 3, 4), the notion of tighter bound ternary complexes is clearly confirmed by the discrepancy in the solvent-accessible surface area (SASA) measured for the HDX-derived protected residues, that is observed between WE and WE-HDX simulations (shown as a function of the minimum I-RMSD in Supplementary Figure 4a). The reduced solvent-accessibility of protected residues in WE-HDX compared to the same residues in WE simulations suggests that the protein-protein interfaces of the corresponding structures are less solvent-exposed, i.e., the simulated SMARCA2$^{BD}$:PROTAC 2:VHL ternary complexes are bound tighter, in the WE-HDX simulations.

The enhanced ternary complex simulation by WE-HDX compared to WE is further emphasized in Supplementary Figure 4b, showing that the HDX-augmented method converges toward structures with low minimum I-RMSD as well as low $C_\alpha$-RMSD values (with respect to a diverse set of ternary complex structures, as discussed below). These results demonstrate that the addition of HDX-MS information significantly improves the prediction accuracy of ternary complex WE simulations. In previous work, the $C_\alpha$-RMSD of the entire ternary complex has been used as a metric to assess the accuracy of ternary complex predictions.[2] However, the enhanced simulations as well as the experiments presented in the main text reveal that the key structural determinants of differences in degradation efficiencies among the three degrader molecules studied are found at the SMARCA2$^{BD}$:VHL interface. Therefore, we focus on the interface-RMSD (I-RMSD) with respect to bound reference structures as the main parameter to evaluate the ternary complexes simulated with the WE-HDX method.

Importantly, in order to not only compare to a single reference structure, which, most likely, would be the experimentally obtained crystal structure, we apply an approach that compares the simulated ternary complex to a set of structurally diverse conformations. This procedure is crucial for a more accurate estimation of the validity of our simulated ternary

conformations, which, as demonstrated in the main text, are highly flexible aggregates.

We derive the set of reference ternary structures from long ($> 1$ $\mu s$) brute-force MD simulations (see Methods). These simulations started from the corresponding ternary complex crystal structures with PROTAC 1, PROTAC 2, or ACBI1 and they sample, for each system, a variety of different binding poses. A $k$-means clustering based on interface residue distances, divides all sampled conformations into $k = 25$ subsets of relatively diverse ternary complexes. The 25 representative structures (or cluster centers) obtained, together with the experimental X-ray crystal structure, constitute the set of reference conformations used for the aforementioned I-RMSD calculations. Supplementary Figure 5 presents superpositions of these reference sets for the three degraders connecting SMARCA2$^{BD}$ to VHL, highlighting the structural heterogeneity among them.

We use minimum I-RMSD with respect to this set of structures as the metric to assess the quality of predicted ternary complexes. As illustrated in Supplementary Figure 4b, all sampled conformations with a minimum I-RMSD $< 2$ Å have a corresponding $C_\alpha$-RMSD $\leq \sim 5$ Å, which is clearly below the $C_\alpha$-RMSD threshold of 10 Å used by Drummond et al.,[3] thus suggesting that 2 Å is an appropriate I-RMSD threshold value to define bound ternary complexes in our study.

The synergy of WE simulations with information on protected residues obtained from HDX experiments is particularly useful for the formation of multi-component aggregates, such as the degrader ternary complexes, when a relatively large solvent-exposed surface is buried upon binding. More generally, the combination of experimental hydrogen-deuterium exchange data with computational modeling and simulation, which has surged over the last decade, can be divided into qualitative and quantitative approaches (as reviewed by Devaurs et al.[4]). In a vast number of recently published studies, HDX protection data are converted into distance or binding restraints that aid in the selection of docked protein complexes,[5–8] such as antibody-antigen[9] or enzyme-inhibitor[10] pairs, and have ultimately led to the development of integrated strategies.[11–13] Notably, Eron et al. have applied this approach for the

prediction of degrader ternary complexes,[14] as described in the main text. These methods have recently also been augmented by molecular simulations, which were either informed by restraints[15,16] or were used to provide atomistic detail on experimentally derived interactions of important membrane and cytosolic proteins, thus establishing a qualitative connection between HDX experiments and simulation.[17–24] On the other hand, all quantitative methods have in common that a protection factor is estimated (usually from molecular simulations) and correlated with results from HDX experiments.[25] This estimate has previously been computed in various ways, such as through the solvent-exposure of amide groups,[26,27] their hydrogen bond propensity,[28–30] or even the protein backbone acidity/reactivity[31,32] and flexibility,[33] in combination with packing densities.[34,35] Since the correlation to experimental protection factors is better when predictions stem from an ensemble of structures rather than from a single structure,[36,37] recent studies have relied on docking[38] and coarse-grained models[39] or enhanced and accelerated simulation techniques[40,41] with reweighting protocols.[42]

Clearly, the WE-HDX method, as implemented in our study, is a qualitative approach that correlates protection data with molecular simulations, as no protection factors or intensities are computed based on the simulated structures. Nevertheless, WE-HDX is distinctly different from most qualitative HDX-modeling protocols in that the WE simulations, which themselves are often referred to as unbiased due to the omission of steering forces or biasing potentials, are guided by information from HDX experiments. The conversion of protection data into interface residue contacts used as a collective variable in the WE-HDX simulations is, to our knowledge, a novel and, based on the results of degrader ternary complex formation, a most intriguing strategy to experimentally augment the simulation of rare events with minimal bias.

# Supplementary Note 2

## Docking protocol

The docking protocol relies on the core assumption that high-fidelity structures are available for both the SMARCA2$^{BD}$:warhead and the VHL:E3-ligand binary complexes. The RosettaDock keeps relative poses of the degrader moieties fixed with respect to their bound protein partners. Thus, if $A$ is the chain ID of SMARCA2$^{BD}$, $X$ is the chain ID of the warhead attached to SMARCA2$^{BD}$, $B$ is the chain ID of VHL and $Y$ is the chain ID of the E3-ligand attached to VHL, the command-line to run RosettaDock is the following:

```
docking_protocol.linuxgccrelease -database database/
-s input_0001.pdb -nstruct $NUM_STR -in:file:extra_res_fa
warhead.params  ligand.params -use_input_sc -docking
-dock_pert 2.7 15 -partners AX_BY -ex1 -ex2aro
-constraints:cst_file constraints.txt
-constraints:cst_fa_weight 10 -out:file:scorefile score.sc
```

This will keep the chains $A$ and $X$ fixed and dock chains $B$ and $Y$ as a single docking partner with respect to $AX$.

The input PDB file input_0001.pdb is a structure with pre-packed side-chains obtained by running:

```
docking_prepack_protocol.linuxgccrelease -database database/
-s input.pdb -use_input_sc -extra_res_fa warhead.params ligand.params
```

Alignment of the linker conformers is performed with the tool presented in:[43]

```
Chapman-Kolmogorov tests for PROTAC 2 MSM.
    python ternary_model_prediction.py -da decoy_atom_list.txt
    -la linker_atom_list.txt -wd decoy_atom_list_delete.txt
```

```
-ld linker_atom_list_delete.txt -dl listDecoysPDB.txt
-ll listConformersPDB.txt -c 0.3 -r rmsd_0.3A.txt -t specify
```

Generation of conformers for the linker is performed with a set of in-house developed Python programs that wrap the CREST software.[44] Pre-processing, post-processing and re-ranking is performed with a set of in-house Python scripts. Calculation of CAPRI parameters is performed by RosettaDock with respect to the input co-crystallized structures.

Supplementary Figure 6 shows distributions of the top-N docking predictions into the four conventional CAPRI quality categories:[45] Incorrect, Acceptable, Medium and High. Obviously, the prediction accuracy is higher when HDX-derived restraints are incorporated (green bars) compared to the conventional docking protocol (orange bars) for Acceptable and Medium and High quality categories at all values of top-N. At the same time, the count of Incorrect predictions is lower for docking "with HDX" data, indicating the advantage of using the restraints derived from the HDX data. This advantage is especially striking for the High-quality predictions: without the HDX restraints, there are no High-quality structures within top-10 and top-50 predictions (see Table 10).

Supplementary Figure 7 (a, b, c) shows the mean values for the fraction of native contacts, $fNat$, which are evaluated for docking predictions with respect to the crystal structure (PDB ID: 6HAX). The observation that HDX-derived restraints lead to higher values of $fNat$ (green versus orange bars) is a direct consequence of the nature of information determined in the experiments: sets of most protected residues that allegedly form the interface. We see this as an evidence for complementarity of employed methods and consistency of our research. Supplementary Figure 7 (d, e, f) and (g, h, i) show the distributions of I-RMSD and C$\alpha$-RMSD values. Following Drummond et al.,[3] the $C_\alpha$-RMSD is defined as the root-mean-square deviation of $C_\alpha$ coordinates after a rigid-body superposition of the whole predicted structure onto the corresponding co-crystallized one. As seen from Supplementary Figure 7, most of the predictions have $C_\alpha$-RMSD values less than 10 Å and fall into the crystal-like category as defined by Drummond et al.[3] Also, the values of I-RMSD and $C_\alpha$-RMSD are

lower when the HDX-derived restraints are used. Similar results are obtained for the ternary complexes with PROTAC 1 and ACBI1 (see Supplementary Figures 11 – 14).

Supplementary Figure 8 shows examples of the docking structures that fall within different categories of quality of predictions. The structures of SMARCA2$^{BD}$ are aligned with the corresponding crystal structure (PDB ID: 6HAX) (salmon) and different predicted poses of the VHL are depicted in purple. The reference pose of the VHL from the same crystal structure is depicted in orange.

# Supplementary Note 3

## HREMD simulations

The Hamiltonian replica-exchange molecular dynamics (HREMD) simulation is a computationally efficient method to enhance the configurational sampling of a biomolecular system.[46,47] Recent studies revealed that HREMD-generated structures of intrinsically disordered proteins[48,49] and G protein-coupled receptors[50,51] achieved an excellent agreement with experiments. Here we implemented HREMD (specifically replica-exchange with solute tempering, REST2)[46,47] method using the software package GROMACS (v2018.8)[52–54] patched with PLUMED (v2.5.4)[55–59] to explore the conformational free energy landscape of the SMARCA2$^{BD}$:VHL degrader ternary complexes. Using this approach, we ran several parallel simulations (replicas) with scaled Hamiltonians by dividing the system into two regions as "hot" and "cold". The Hamiltonian of the "hot" region (viz., SMARCA2$^{BD}$, VHL, and degrader atoms) was scaled by a factor $\lambda$ (Supplementary Equation ( 1)) in higher rank replicas. Whereas the Hamiltonian of the "cold" region (solvent) was unaltered for all the replicas. Specifically, the Lennard-Jones parameter ($\epsilon$), the dihedral term, and the charge of the atoms in the "hot" region of the $i^{th}$ replica are scaled by $\lambda_i$, $\lambda_i$, and $\sqrt{\lambda_i}$ respectively given by Equation ( 1),

$$\lambda_i = \frac{T_0}{T_i} = exp\Big( - \frac{i}{(n-1)} ln\Big(\frac{T_{max}}{T_0}\Big)\Big) \tag{1}$$

where $n$ is the total number of replicas, and $T_0$, $T_i$ and $T_{max}$ are the effective temperatures of the lowest (unscaled), the $i^{th}$, and the highest rank replicas, respectively. Therefore, only the force field terms that contribute to the energy barriers were scaled.[46] The exchange of coordinates was allowed after every 500 MD steps (1 ps) between the neighboring replicas if the Monte Carlo metropolis criterion was satisfied.[46] In principle, this scheme helps to rapidly sample the conformational substates of a "hot" region.

# Supplementary Figures

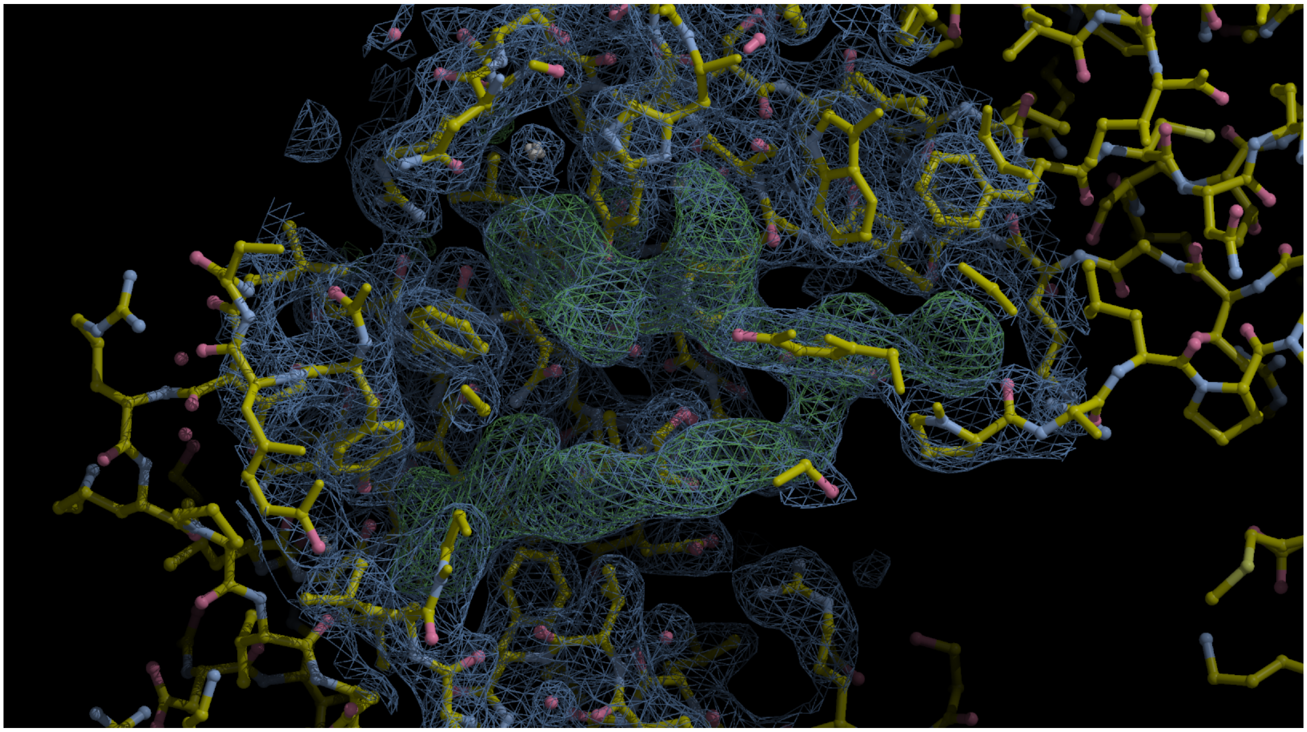

**Supplementary Figure 1:** Omit map of ACBI1 bound to SMARCA2$^{BD}$ and VHL contoured at 1.00 sigma weighted map and 3.00 sigma difference map.

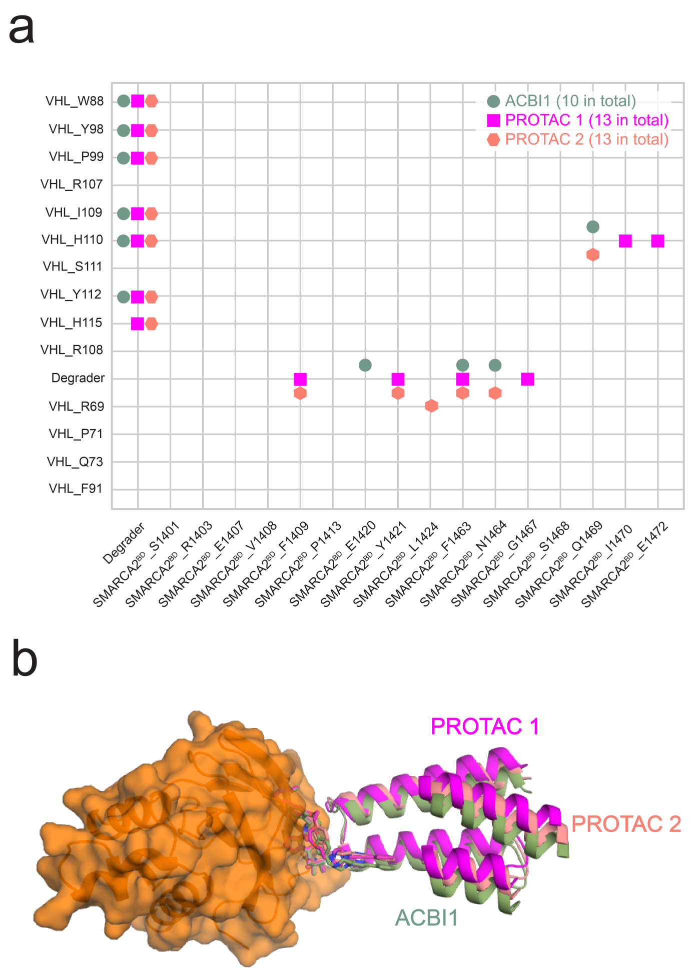


**Supplementary Figure 2:** Comparison of the crystal structures of SMARCA2$^{BD}$:VHL complex bound to PROTAC 1 (PDB ID: 6HAY), PROTAC 2 (PDB ID: 6HAX), or ACBI1 (PDB ID: 7SE4). **a** Protein-protein and protein-degrader contacts observed in the ternary complex crystal structures. The total number of contacts are indicated for each complex in the top right. **b** The alignment of the three crystal structures. VHL is colored in orange and shown in cartoon and surface representation. SMARCA2$^{BD}$ of the ternary complexes with PROTAC 1, PROTAC 2 and ACBI1 are shown in cartoon and colored in magenta, salmon, and green respectively.

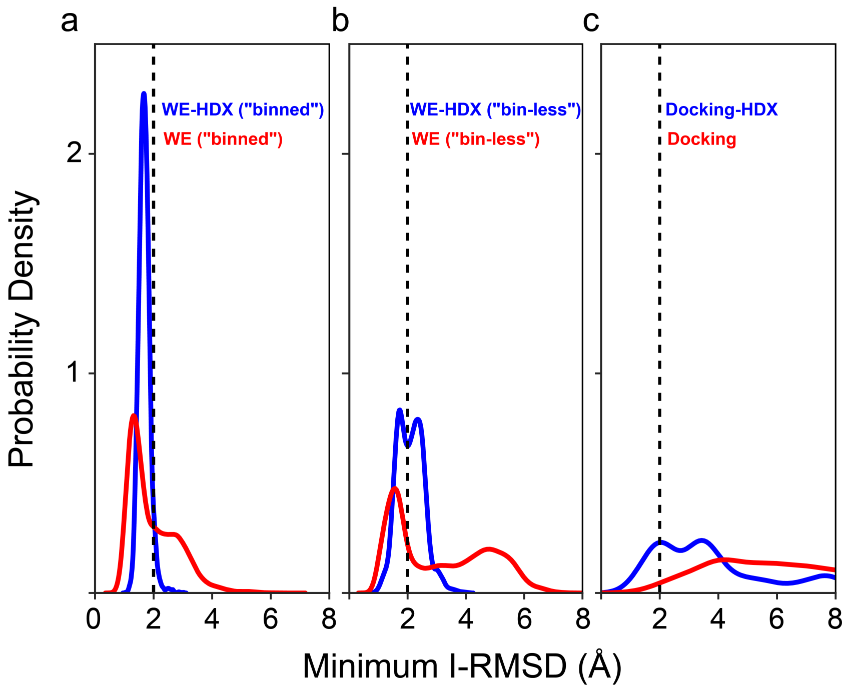


**Supplementary Figure 3:** Comparison of, **a**, binned, **b**, bin-less WE simulations of ternary complex formation, and, **c**, ternary complex docking for SMARCA2$^{BD}$:PROTAC 2:VHL with (red) and without (blue) data from HDX-MS experiments. The vertical dashed lines indicate the thresholds at 2 Å used to define a bound ternary complex (as described in Supplementary Note 1). Source data are provided as a Source Data file.

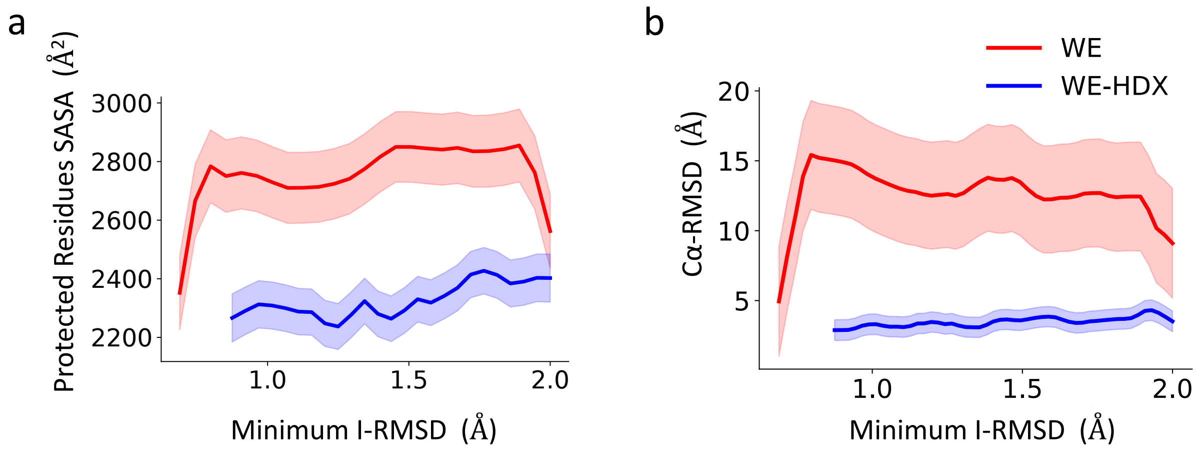


**Supplementary Figure 4:** Minimum I-RMSD of SMARCA2$^{BD}$:PROTAC 2:VHL versus, **a**, the solvent-accessible surface area (SASA) of the HDX-protected residues (see Supplementary Table 8) and, **b**, the $C_\alpha$-RMSD during WE (red) and WE-HDX (blue) simulations of ternary complex formation. The minimum I-RMSD is with respect to a diverse set of reference structures (as described in Supplementary Note 1) and the distribution of SASAs or $C_\alpha$-RMSDs is obtained for those structures with the minimum I-RMSDs. The solid lines and shaded regions are the arithmetic mean and one standard error of the SASAs or the $C_\alpha$-RMSDs, respectively. Source data are provided as a Source Data file.

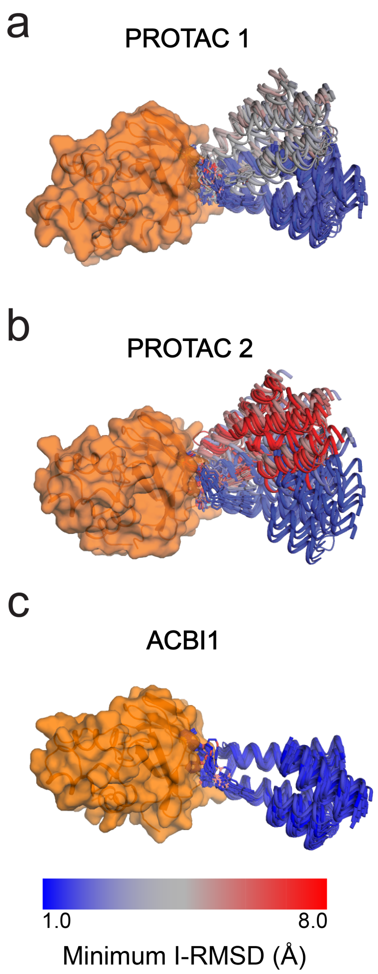


**Supplementary Figure 5:** Superposition of the set of reference structures used for the analysis of SMARCA2$^{BD}$:degrader:VHL ternary complex predictions with, **a**, PROTAC 1, **b**, PROTAC 2, and **c**, ACBI1. The individual VHL molecules (orange, surface representation) are aligned, the degraders are shown in stick, and the SMARCA2$^{BD}$ in cartoon representations. SMARCA2$^{BD}$ is colored based on the I-RMSD value of the ternary complex with respect to the corresponding crystal structure (see color bar). Obviously, the ternary complex reference structures with PROTAC 2 are most diverse among the three degraders.

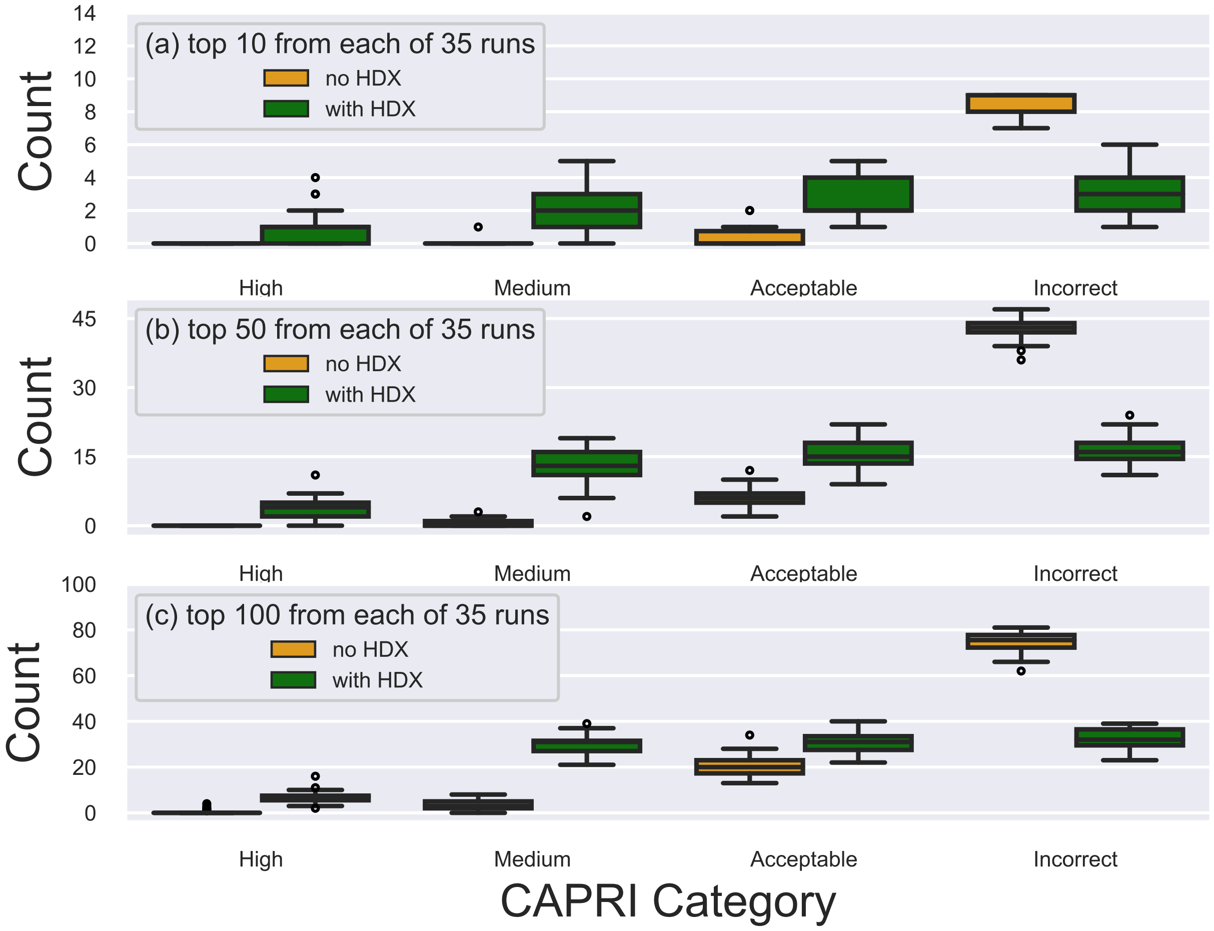


**Supplementary Figure 6:** Distributions of the top-N docking predictions into the CAPRI quality categories High, Medium, Acceptable, and Incorrect. 35 independent docking runs have been performed and mean values have been calculated for, **a**, the top-10, **b**, the top-50, and, **c**, the top-100 docking predictions with (green) and without (orange) HDX-derived restraints. The horizontal box lines indicate the median (middle), first (Q1; bottom) and third quartiles (Q3; top) and the vertical box lines describe the interquartile range (IQR); the lower and upper whisker lines indicate the minimum and maximum value, respectively, excluding outliers, which are the shown individual data points that are smaller than Q1-1.5*IQR or larger than Q3+1.5*IQR. Results presented are for SMARCA2$^{BD}$:PROTAC 2:VHL (PDB ID: 6HAX). See also Supplementary Table 10. Source data are provided as a Source Data file.

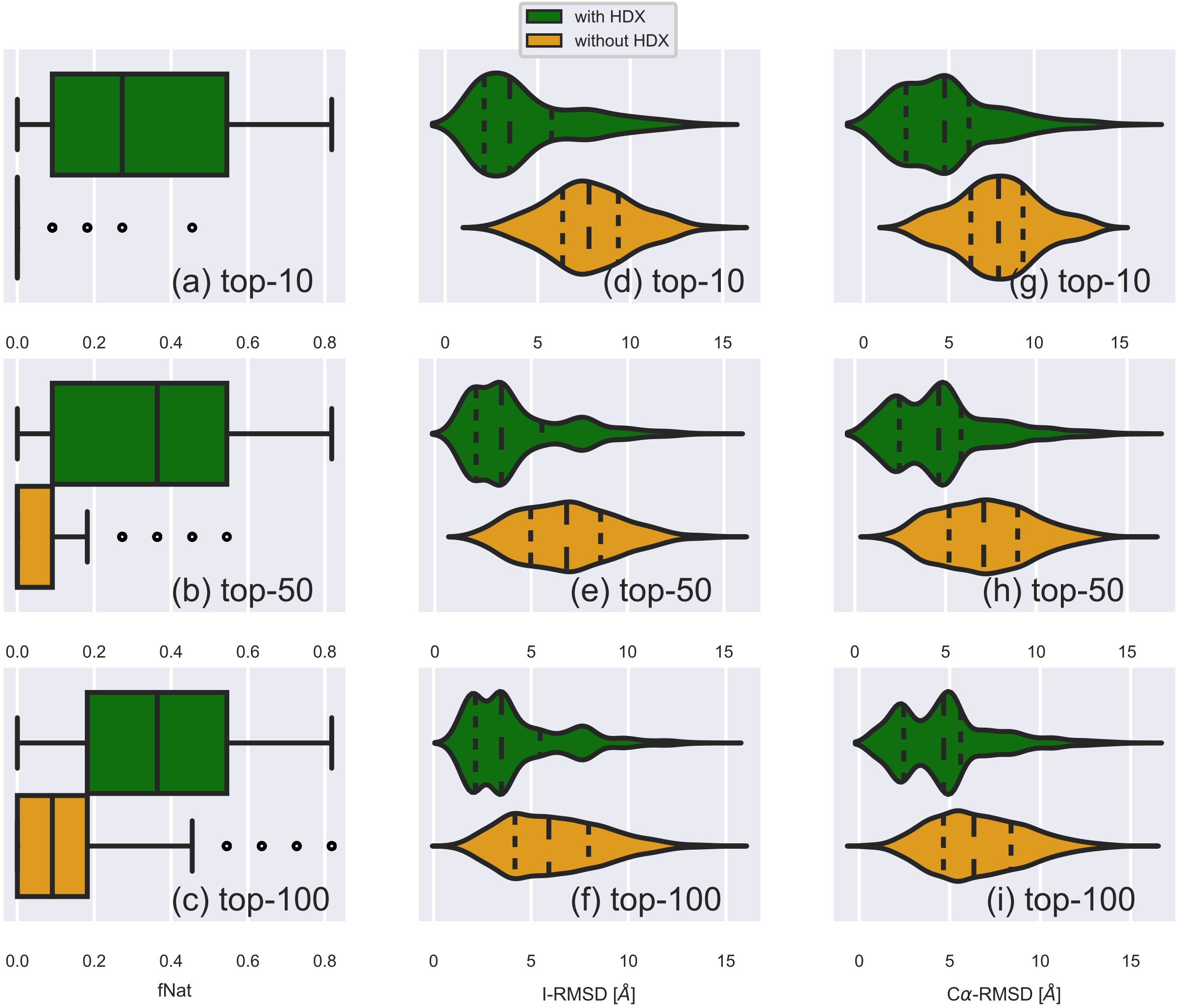


**Supplementary Figure 7:** Values of the fNat (**a, b, c**) and distributions of the I-RMSDs (**d, e, f**) and $C_\alpha$-RMSDs (**g, h, i**) calculated over 35 independent runs for the top-10 (**a, d, g**), the top-50 (**b, e, h**), and the top-100 (**c, f, i**) docking predictions with (green) and without (orange) HDX-derived restraints. In panels a-c, the vertical box lines indicate the median (middle), first (Q1; left) and third quartiles (Q3; right) and the horizontal box lines describe the interquartile range (IQR); the left and right whisker lines indicate the minimum and maximum value, respectively, excluding outliers, which are the shown individual data points that are smaller than Q1-1.5*IQR or larger than Q3+1.5*IQR. In panels d-i, the median and first and third quartiles are shown as long and short-dashed vertical lines. Results presented are for SMARCA2$^{BD}$:PROTAC 2:VHL (PDB ID: 6HAX). Source data are provided as a Source Data file.

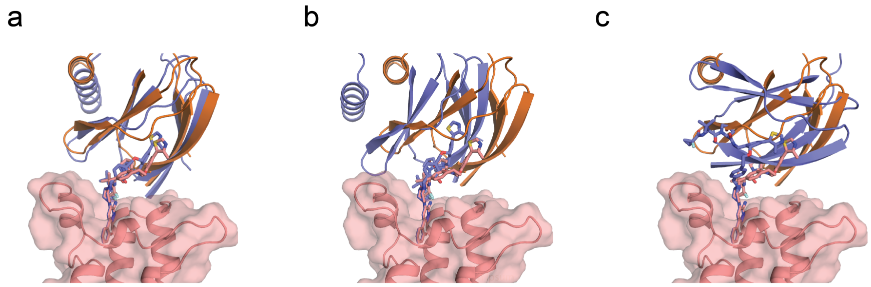


**Supplementary Figure 8:** Illustration of the docking-based predicted ternary structures superimposed onto the corresponding co-crystallized structure (PDB ID: 6HAX). **a** High quality prediction; **b** Medium quality prediction; **c** Incorrect prediction. SMARCA2$^{BD}$ is shown in salmon cartoon and surface representation, the VHL pose from the crystal structure is shown in orange cartoon representation, and the predicted docking pose of VHL is shown in purple cartoon representation.

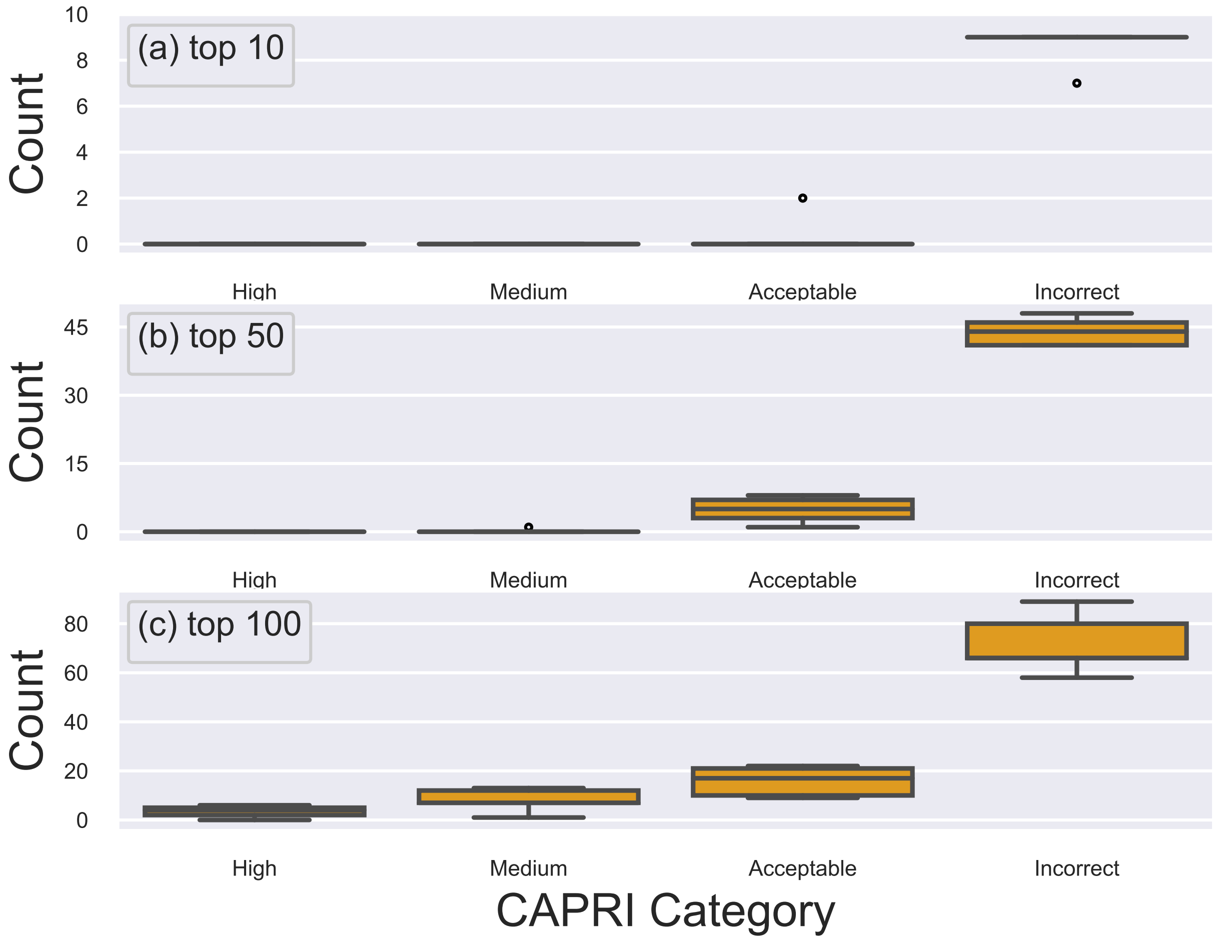


**Supplementary Figure 9:** Distributions of the Top-N docking predictions into the CAPRI quality categories High, Medium, Acceptable and Incorrect. 5 independent docking runs (without any HDX-MS-derived restraints) have been performed and mean values have been calculated for, **a**, the top-10, **b**, the top-50, and, **c**, the top-100 docking predictions. The horizontal box lines indicate the median (middle), first (Q1; bottom) and third quartiles (Q3; top) and the vertical box lines describe the interquartile range (IQR); the lower and upper whisker lines indicate the minimum and maximum value, respectively, excluding outliers, which are the shown individual data points that are smaller than Q1-1.5*IQR or larger than Q3+1.5*IQR. Results presented are for SMARCA2$^{BD}$:PROTAC 1:VHL (PDB ID: 6HAY). Source data are provided as a Source Data file.

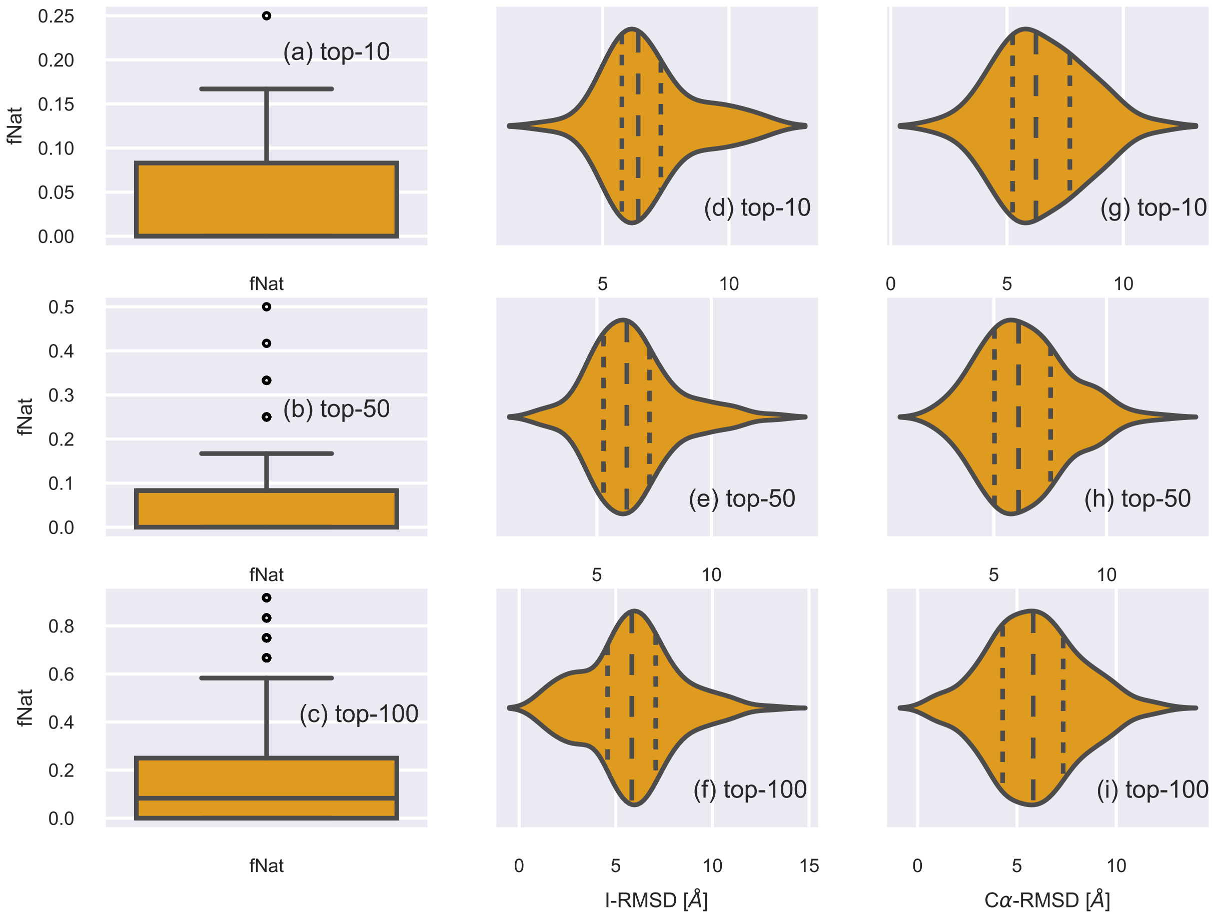


**Supplementary Figure 10:** Values of the fNat (**a,b,c**) and distributions of the I-RMSD (**d, e, f**) and $C_\alpha$-RMSD (**g, h, i**) calculated over 5 independent runs (without any HDX-MS-derived restraints) for the top-10 (**a, d, g**), the top-50 (**b, e, h**), and the top-100 (**c, f, i**) docking predictions. In panels a-c, the horizontal box lines indicate the median (middle), first (Q1; bottom) and third quartiles (Q3; top) and the vertical box lines describe the interquartile range (IQR); the lower and upper whisker lines indicate the minimum and maximum value, respectively, excluding outliers, which are the shown individual data points that are smaller than Q1-1.5*IQR or larger than Q3+1.5*IQR. In panels d-i, the median and first and third quartiles are shown as long and short-dashed vertical lines. Results presented are for SMARCA2$^{BD}$:PROTAC 1:VHL (PDB ID: 6HAY). Source data are provided as a Source Data file)

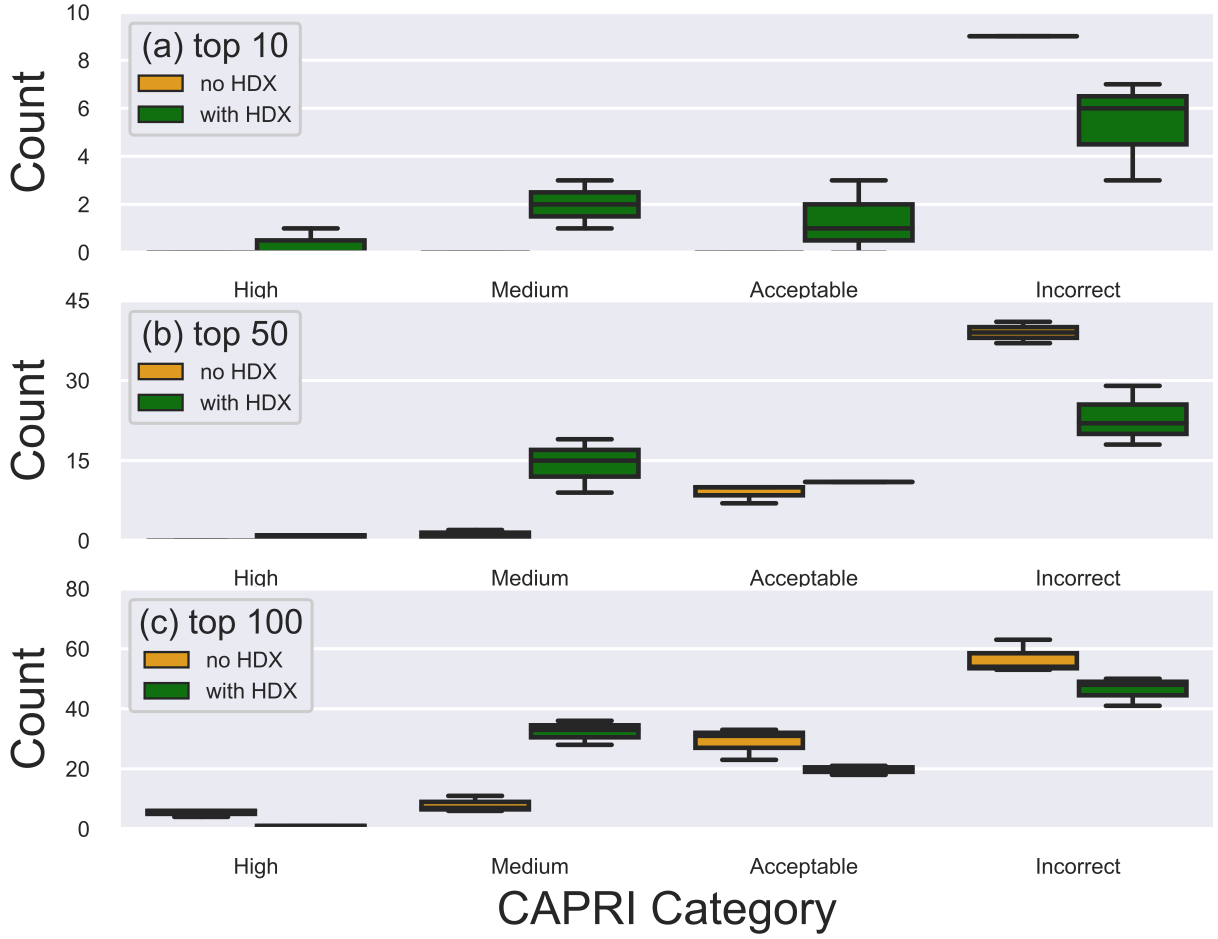


**Supplementary Figure 11:** Distributions of the Top-N docking predictions into the CAPRI quality categories High, Medium, Acceptable, and Incorrect. 3 independent docking runs have been performed and mean values have been calculated for, **a**, the top-10, **b**, the top-50, and, **c**, the top-100 docking predictions with (green) and without (orange) HDX-derived restraints. The horizontal box lines indicate the median (middle), first (Q1; bottom) and third quartiles (Q3; top) and the vertical box lines describe the interquartile range (IQR); the lower and upper whisker lines indicate the minimum and maximum value, respectively, excluding outliers, which are the shown individual data points that are smaller than Q1-1.5*IQR or larger than Q3+1.5*IQR. Results presented are for iso1 – SMARCA2$^{BD}$:ACBI1:VHL. Source data are provided as a Source Data file.

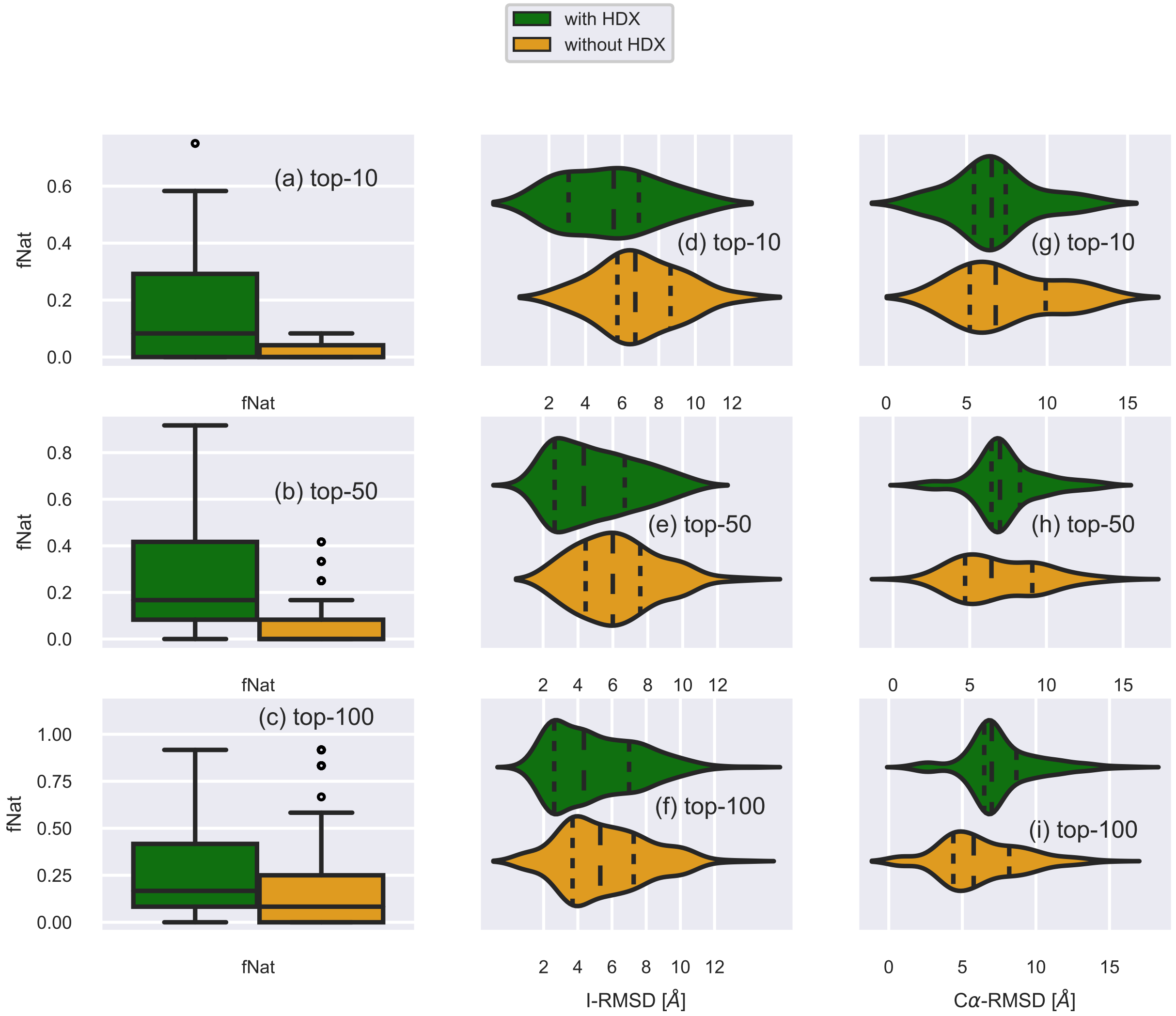


**Supplementary Figure 12:** Values of the fNat (**a, b, c**) and distributions of the I-RMSDs (**d, e, f**) and $C_\alpha$-RMSDs (**g, h, i**) calculated over 3 independent runs for the top-10 (**a, d, g**), the top-50 (**b, e, h**) and the top-100 (**c, f, i**) docking predictions with (green) and without (orange) HDX-derived restraints. In panels a-c, the horizontal box lines indicate the median (middle), first (Q1; bottom) and third quartiles (Q3; top) and the vertical box lines describe the interquartile range (IQR); the lower and upper whisker lines indicate the minimum and maximum value, respectively, excluding outliers, which are the shown individual data points that are smaller than Q1-1.5*IQR or larger than Q3+1.5*IQR. In panels d-i, the median and first and third quartiles are shown as long and short-dashed vertical lines. Results presented are for iso1 – SMARCA2$^{BD}$:ACBI1:VHL. Source data are provided as a Source Data file.

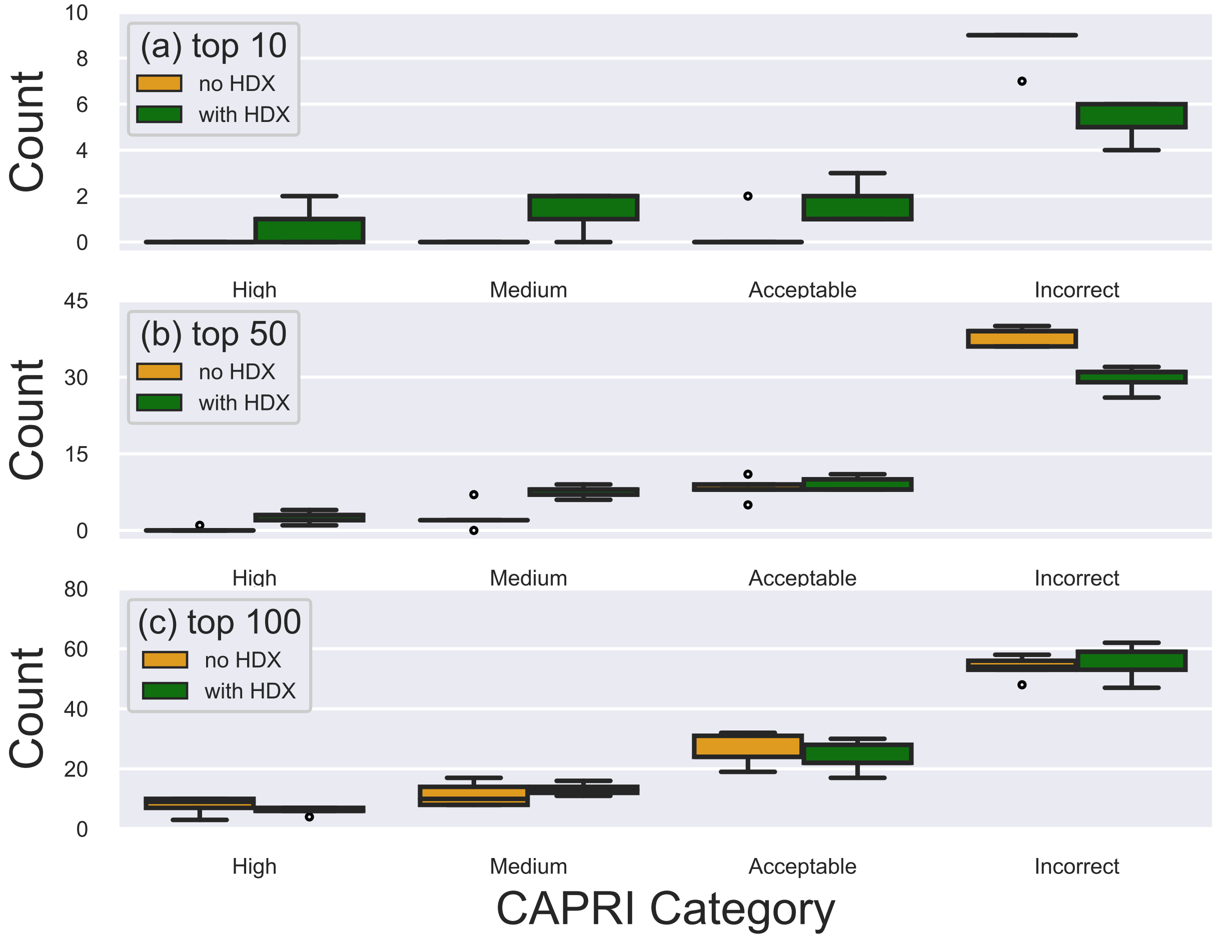


**Supplementary Figure 13:** Distributions of the Top-N docking predictions into the CAPRI quality categories High, Medium, Acceptable and Incorrect. 5 Independent docking runs have been performed and mean values have been calculated for, **a**, the top-10, **b**, the top-50, and, **c**, the top-100 docking predictions with (green) and without (orange) HDX-derived restraints. The horizontal box lines indicate the median (middle), first (Q1; bottom) and third quartiles (Q3; top) and the vertical box lines describe the interquartile range (IQR); the lower and upper whisker lines indicate the minimum and maximum value, respectively, excluding outliers, which are the shown individual data points that are smaller than Q1-1.5*IQR or larger than Q3+1.5*IQR. Results presented are for iso2-SMARCA2$^{BD}$:ACBI1:VHL. Source data are provided as a Source Data file.

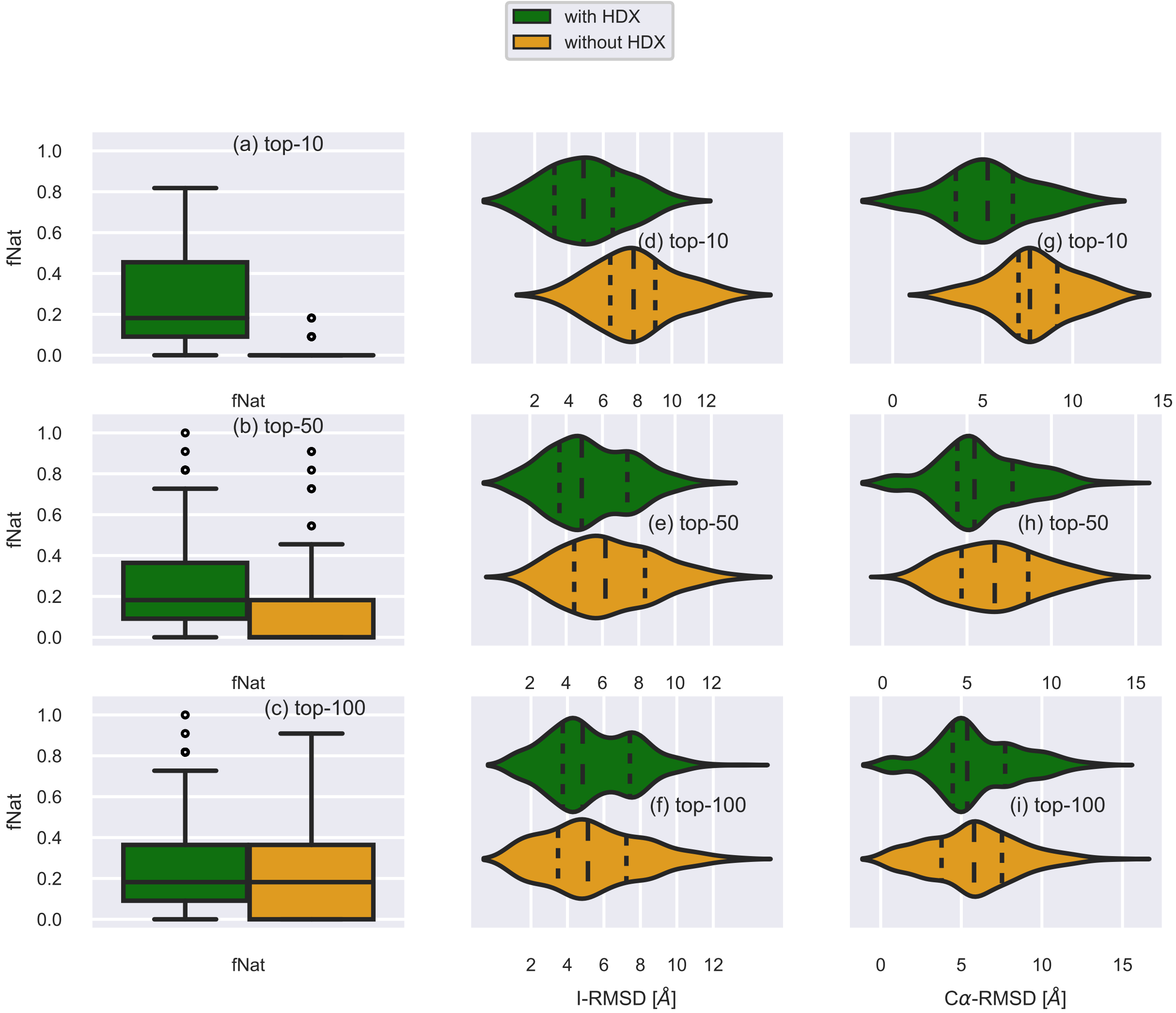


**Supplementary Figure 14:** Values of the fNat (**a, b, c**) and distributions of the I-RMSDs (**d, e, f**) and $C_\alpha$-RMSDs (**g, h, i**) calculated over 5 independent runs for the top-10 (**a, d, g**), the top-50 (**b, e, h**) and the top-100 (**c, f, i**) docking predictions with (green) and without (orange) HDX-derived restraints. In panels a-c, the horizontal box lines indicate the median (middle), first (Q1; bottom) and third quartiles (Q3; top) and the vertical box lines describe the interquartile range (IQR); the lower and upper whisker lines indicate the minimum and maximum value, respectively, excluding outliers, which are the shown individual data points that are smaller than Q1-1.5*IQR or larger than Q3+1.5*IQR. In panels d-i, the median and first and third quartiles are shown as long and short-dashed vertical lines. Results presented are for iso2-SMARCA2$^{BD}$:ACBI1:VHL. Source data are provided as a Source Data file.

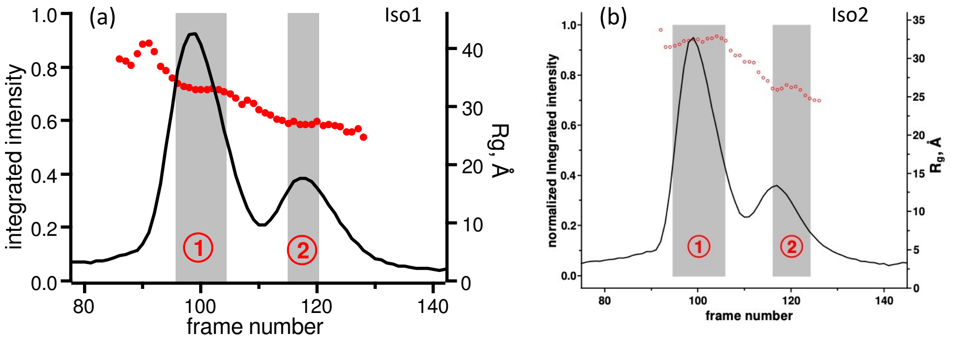


**Supplementary Figure 15:** The normalized integrated SAXS intensity (left y-axis, black lines) and the corresponding radius of gyration, $R_g$ (right y-axis, red circles), for each SEC frame for, **a**, iso1-SMARCA2$^{BD}$:ACBI1:VCB and, **b**, iso2-SMARCA2$^{BD}$:ACBI1:VCB. The highlighted elution peaks 1 (frames 96-103 for iso1-SMARCA2$^{BD}$:ACBI1:VCB and frames 94-105 for iso2-SMARCA2$^{BD}$:ACBI1:VCB) correspond to the respective (full) ternary complexes, whereas the elution peaks 2 indicate uncomplexed or binary species. $R_g$ is derived from the Guinier approximation (see Supplementary Figure 16) based on Equation (1) in the main text. Source data are provided as a Source Data file.

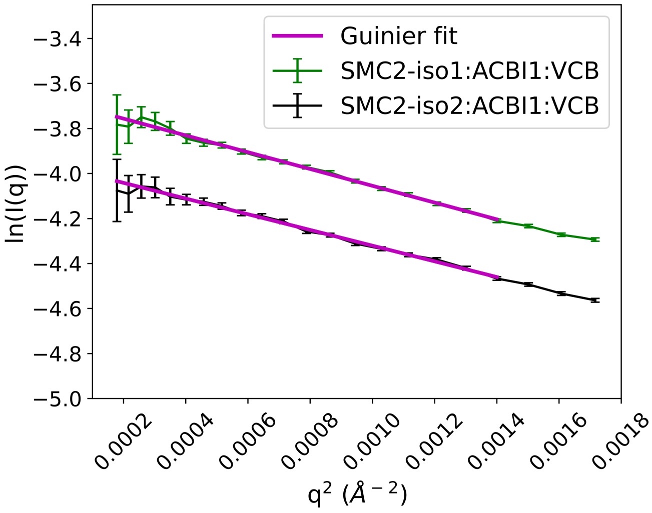


**Supplementary Figure 16:** Guinier analysis. $R_g$ of a ternary complex is determined using the Guinier approximation at low $q$-values (see Equation (1) in the main text). The magenta solid lines are the Guinier fits to the SAXS data shown in green and black, respectively. Error bars describe the experimental uncertainty of ln(I(q)) at a given q (see Methods in the main text). Source data are provided as a Source Data file.

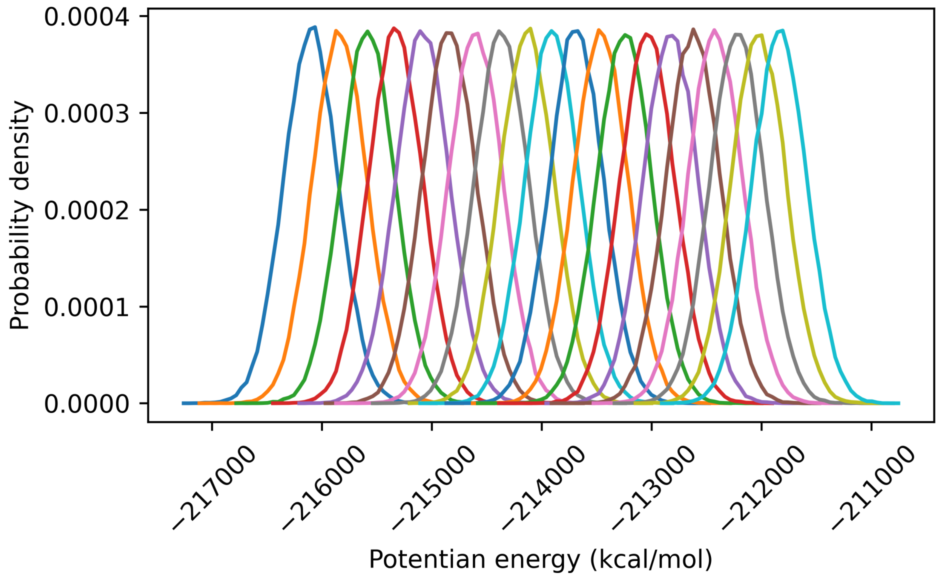


**Supplementary Figure 17:** Potential energy of all replicas from HREMD simulation of **Sys7** (see Supplementary Table 13). Left to right: rank 0 to rank 19. A good overlap between adjacent replicas suggests a sufficient number of replicas were employed and also confirms that no phase transition took place during the HREMD simulation. Source data are provided as a Source Data file.

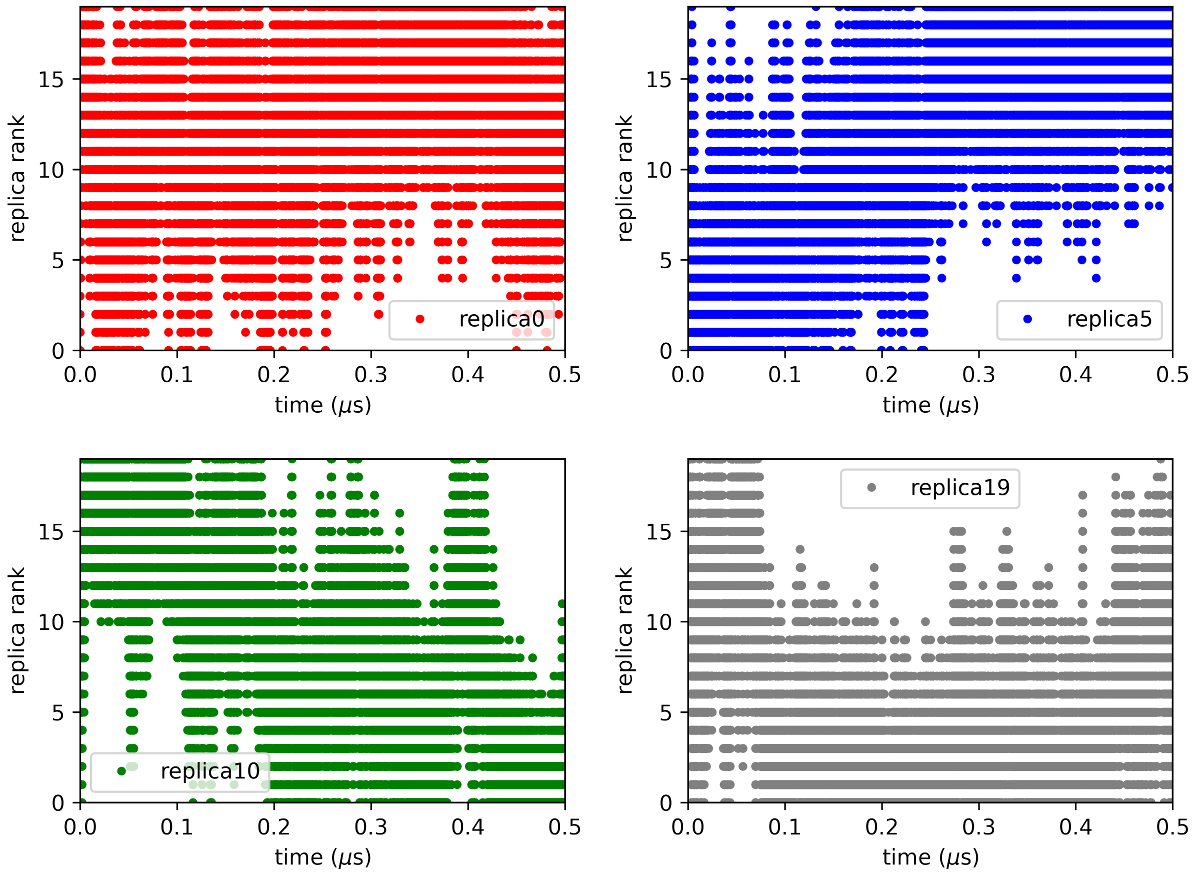


**Supplementary Figure 18:** Effective temperature trajectories of replicas ranked 0 (red), 5 (blue), 10 (green) and 19 (grey) from HREMD simulation of **Sys7** (see Supplementary Table 13). Source data are provided as a Source Data file.

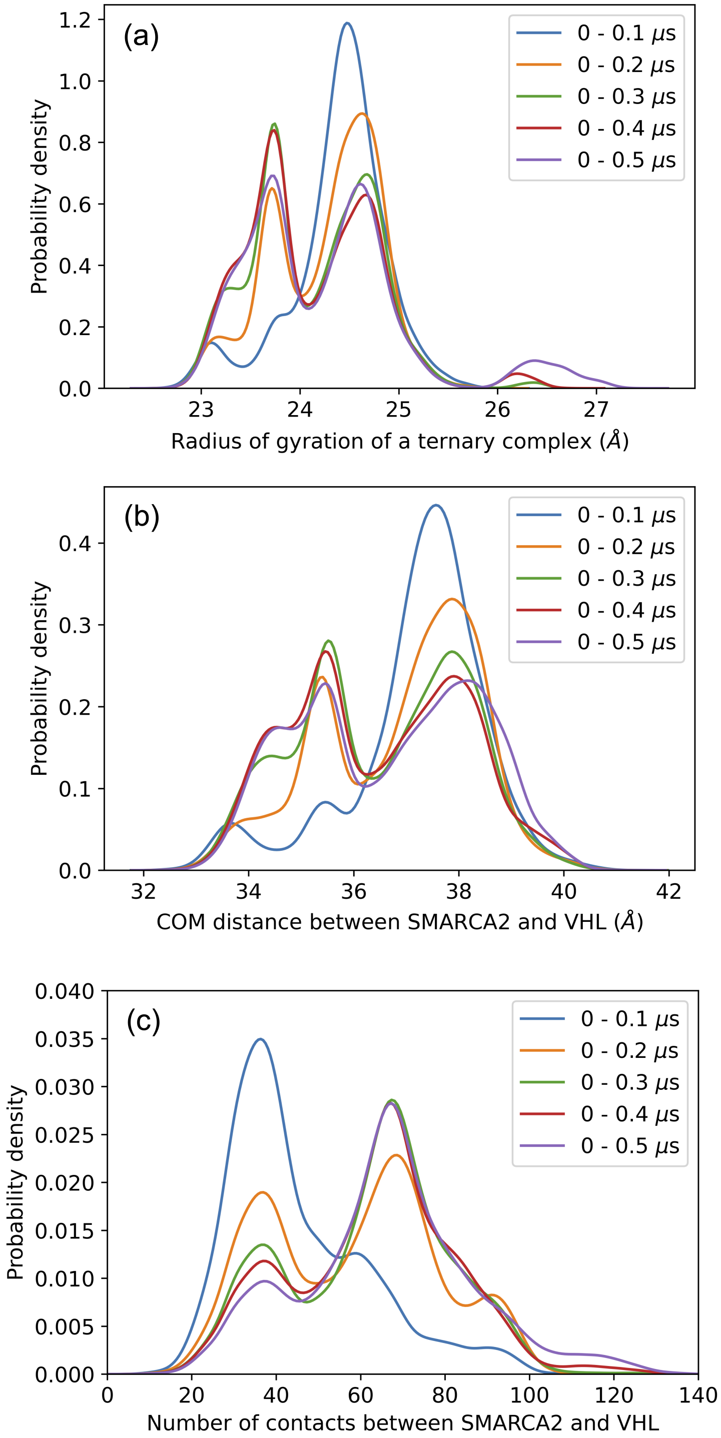


**Supplementary Figure 19:** HREMD convergence test. The distribution (using kernel density estimation) of, **a**, radius of gyration of the ternary complex, **b**, center of mass (COM) distance between $SMARCA2^{BD}$ and VHL, and, **c**, heavy atom contacts within 5 Å between $SMARCA2^{BD}$ and VHL are plotted with cumulative length of HREMD simulation for **Sys7** (see Supplementary Table 13). Source data are provided as a Source Data file.

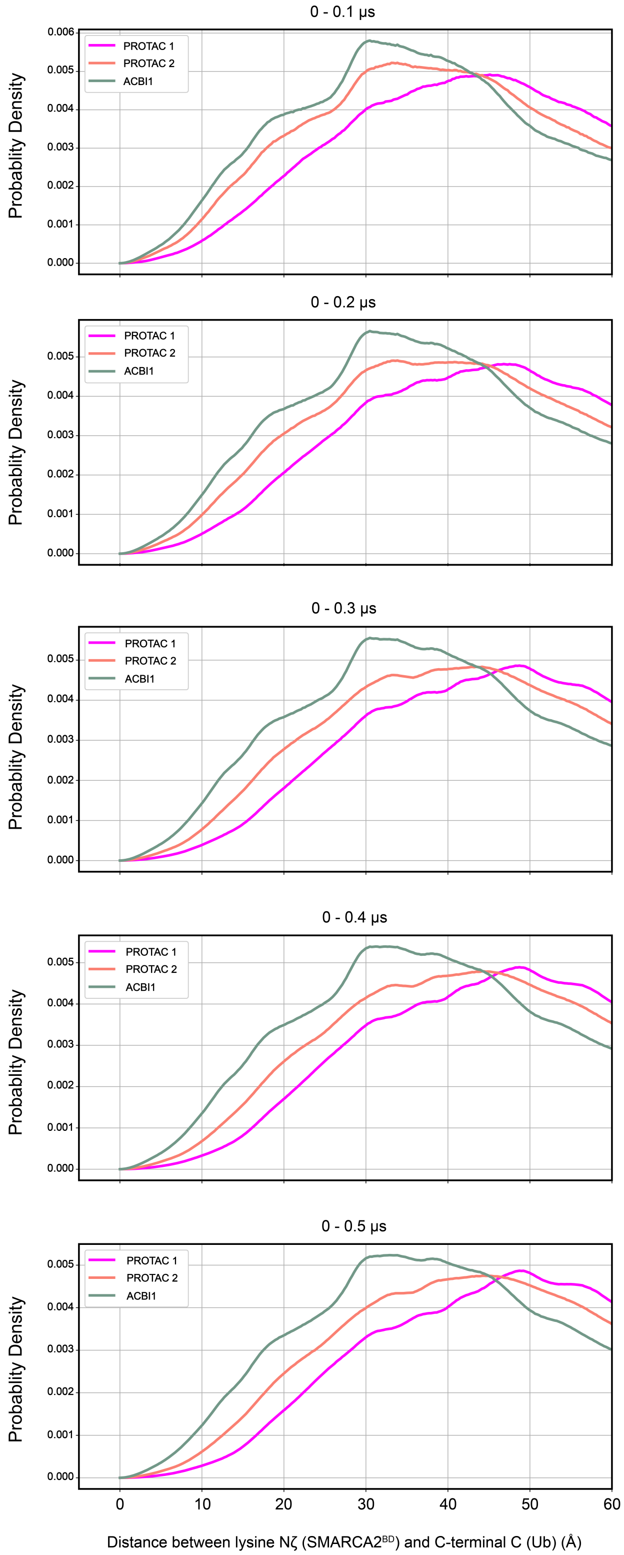


**Supplementary Figure 20:** HREMD convergence test. Distance of lysine residues (side-chain nitrogen atom) from SMARCA2 to the C-terminus glycine C atom of ubiquitin for three different degraders (PROTAC 1, PROTAC 2 and ACBI1) plotted with simulation lengths to confirm the convergence of the result. Source data are provided as a Source Data file.

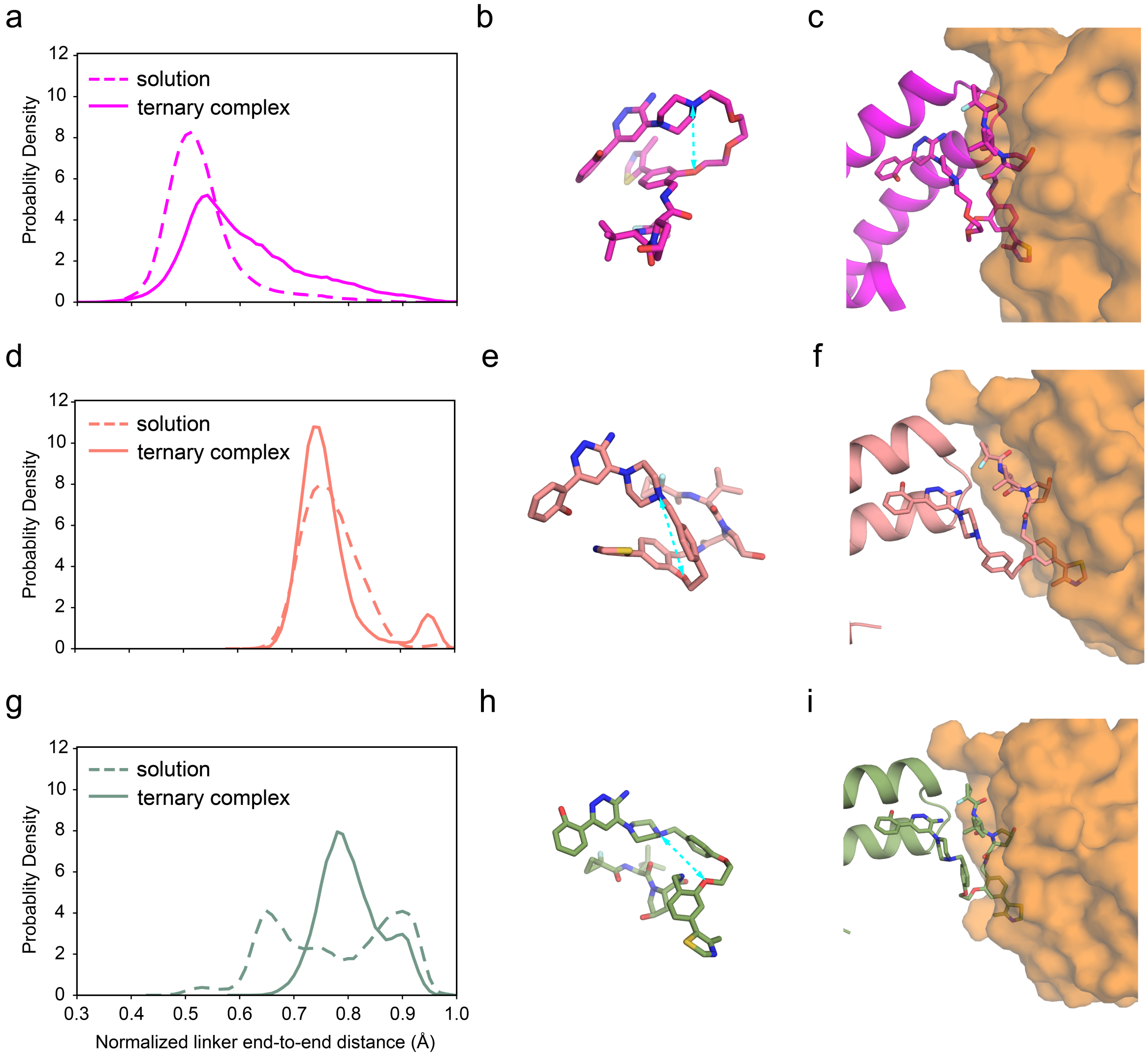


**Supplementary Figure 21:** Degrader compatibility in a ternary complex. Comparison of linker end-to-end distance normalized by the number of linker backbone atoms in solution (dashed line) vs. in ternary complex (solid line) for, **a**, PROTAC 1, **d**, PROTAC 2, and, **g**, ACBI1. Snapshots of conformation of PROTAC 1 (magenta, **b** and **c**), PROTAC 2 (salmon, **e** and **f**) and ACBI1 (green, **h** and **i**) in solution and in ternary complex are shown by stick representation. The cyan dashed line with arrows indicates the linker end-to-end distance. Source data are provided as a Source Data file.

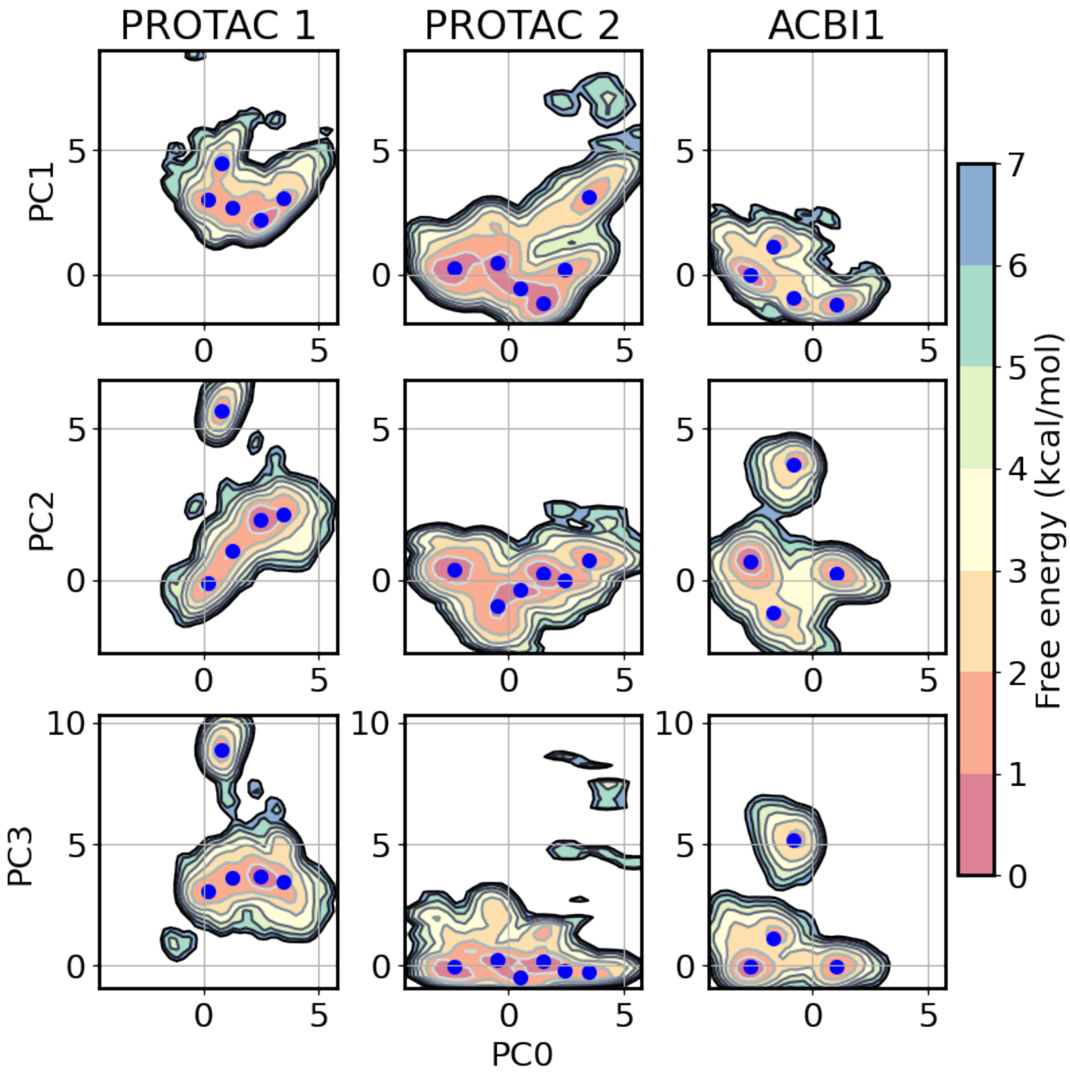


**Supplementary Figure 22:** Free energy landscapes determined from PCA projections of iso2-SMARCA2$^{BD}$ bound to VHL via PROTAC 1 (left), PROTAC 2 (middle), and ACBI1 (right). Blue points indicate $k$-means centroids, which show an approximate correspondence with local minima of the free energy surface. To facilitate comparison, the landscapes are here shown projected onto the same PCA space, determined from interface distances of the PROTAC 2 system.

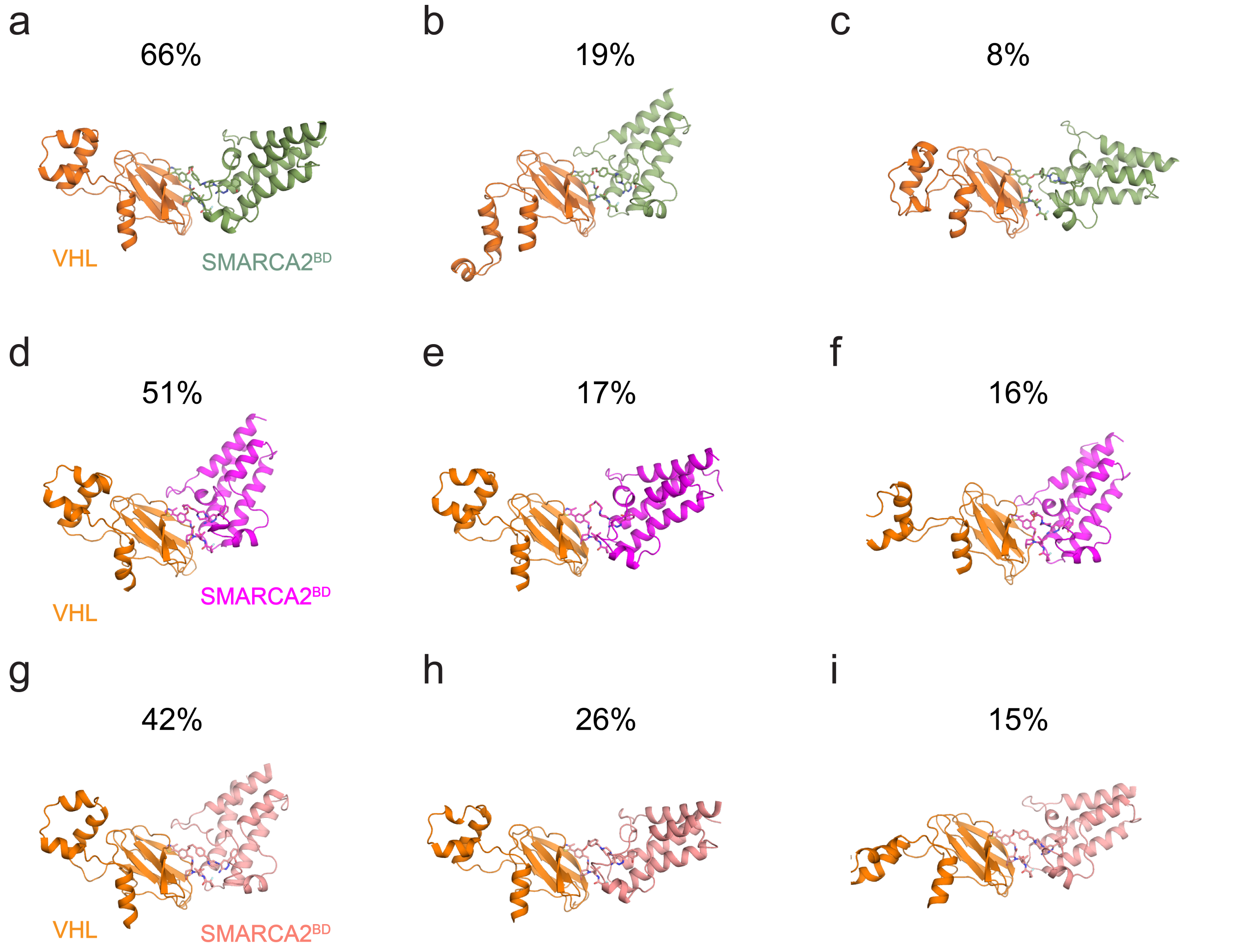


**Supplementary Figure 23:** Cluster centroids from the three highest populated structures of iso2-SMARCA2$^{BD}$ bound to VHL via (**a-c**) ACBI1, (**d-f**) PROTAC 1, and (**g-i**) PROTAC 2, along with their populations. Less populated structures are omitted.

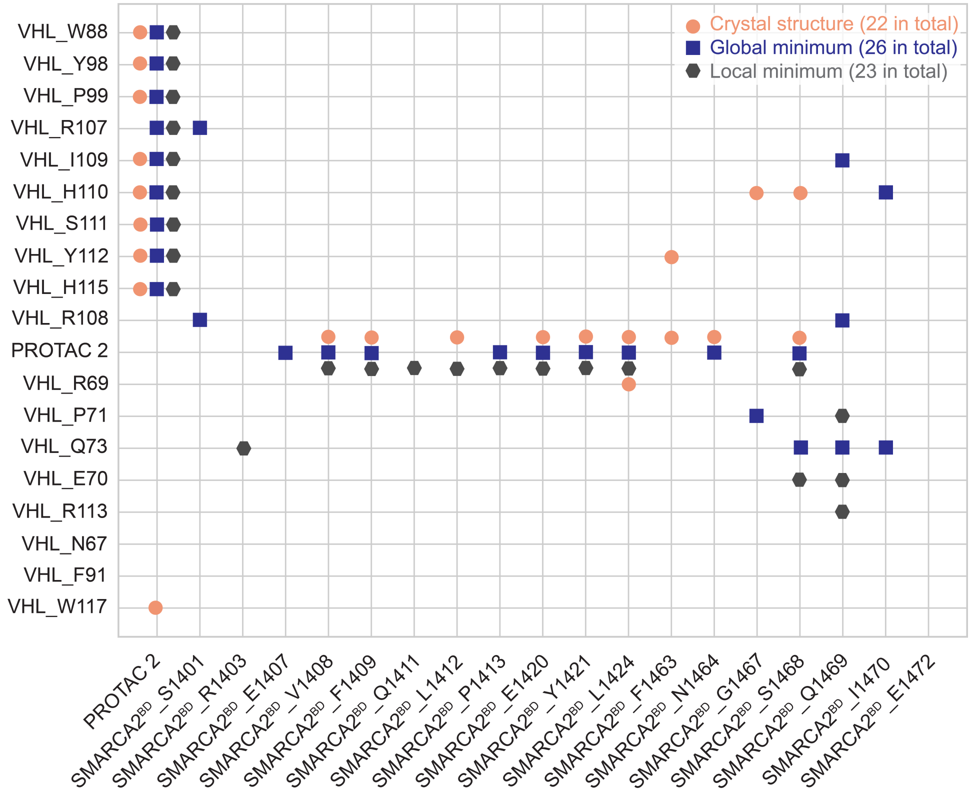


**Supplementary Figure 24:** Contact maps for the iso2-SMARCA2$^{BD}$:PROTAC 2:VHL ternary complex crystal structure (PDB ID: 6HAX; orange circles) and its global minimum (blue squares) and metastable (gray hexagons) states identified by our MSM.

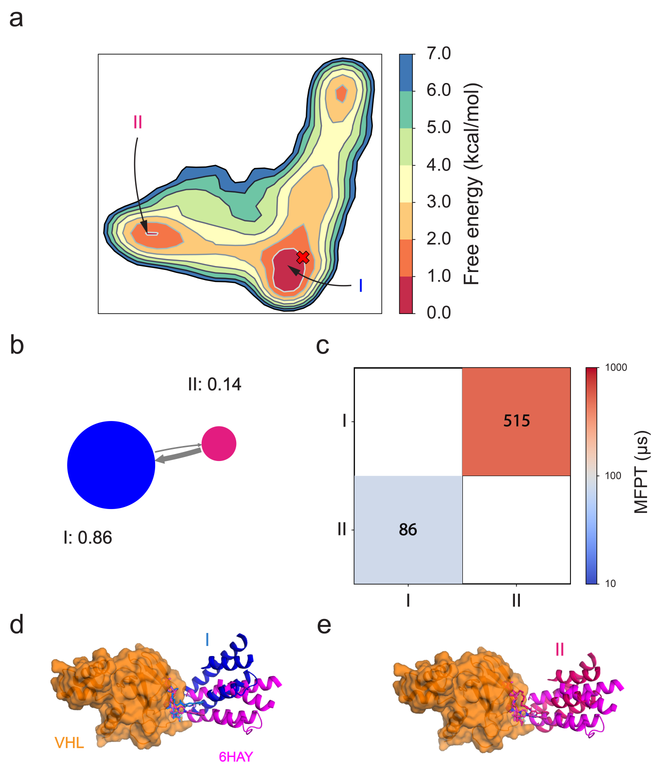


**Supplementary Figure 25:** Markov-state Model for the iso2-SMARCA2$^{BD}$:PROTAC 1:VHL system. **a** Conformational free energy landscape as a function of the first two tICA features. The crystal structure (PDB ID: 6HAY) is shown as a red X. **b** Network diagram of the coarse-grained MSM calculated with a lag time of 100 ns, with the stationary probabilities associated with each state indicated. **c** Mean-first passage times (MFPTs) to transition from one state to another in the coarse-grained MSM. Numbers indicate predicted MFPTs in $\mu s$. **d**, **e** Comparison of the crystal structure (magenta) with the lowest free energy state (blue) and the metastable state (red) predicted by the MSM.

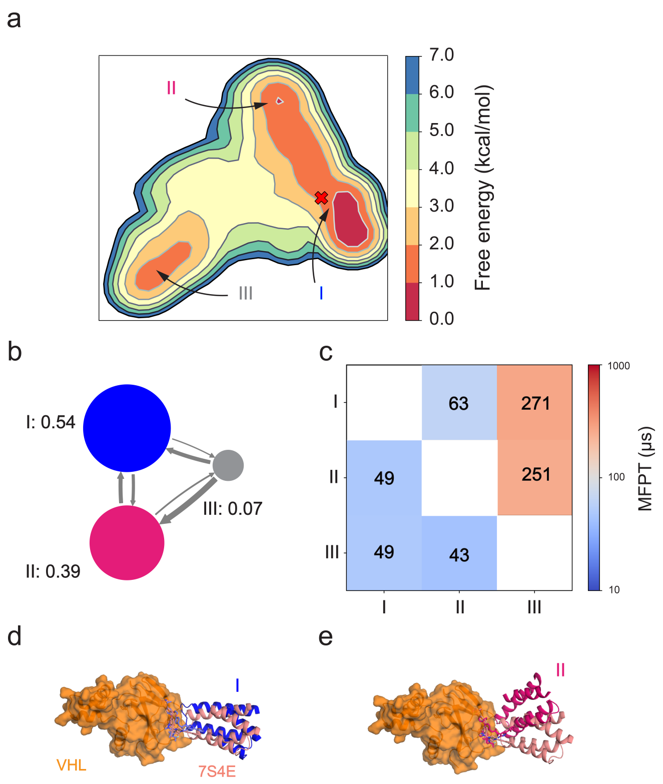


**Supplementary Figure 26:** Markov-state Model for the iso2-SMARCA2$^{BD}$:ACBI1:VHL system. **a** Conformational free energy landscape as a function of the first two tICA features. The crystal structure (PDB ID: 7S4E) is shown as a red X. **b** Network diagram of the coarse-grained MSM calculated with a lag time of 100 ns, with the stationary probabilities associated with each state indicated. **c** Mean-first passage times (MFPTs) to transition from one state to another in the coarse-grained MSM. Numbers indicate predicted MFPTs in $\mu s$. **d**. **e** Comparison of the crystal structure (salmon) with the lowest free energy state (blue) and the metastable state (red) predicted by the MSM.

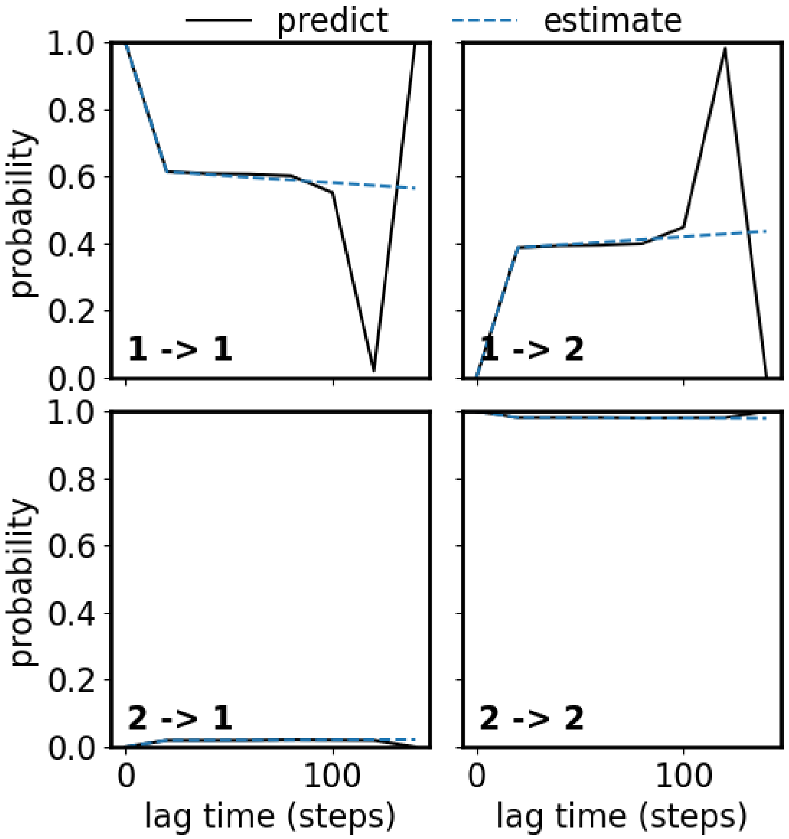


**Supplementary Figure 27:** Chapman-Kolmogorov tests for PROTAC 1 MSM. Source data are provided as a Source Data file.

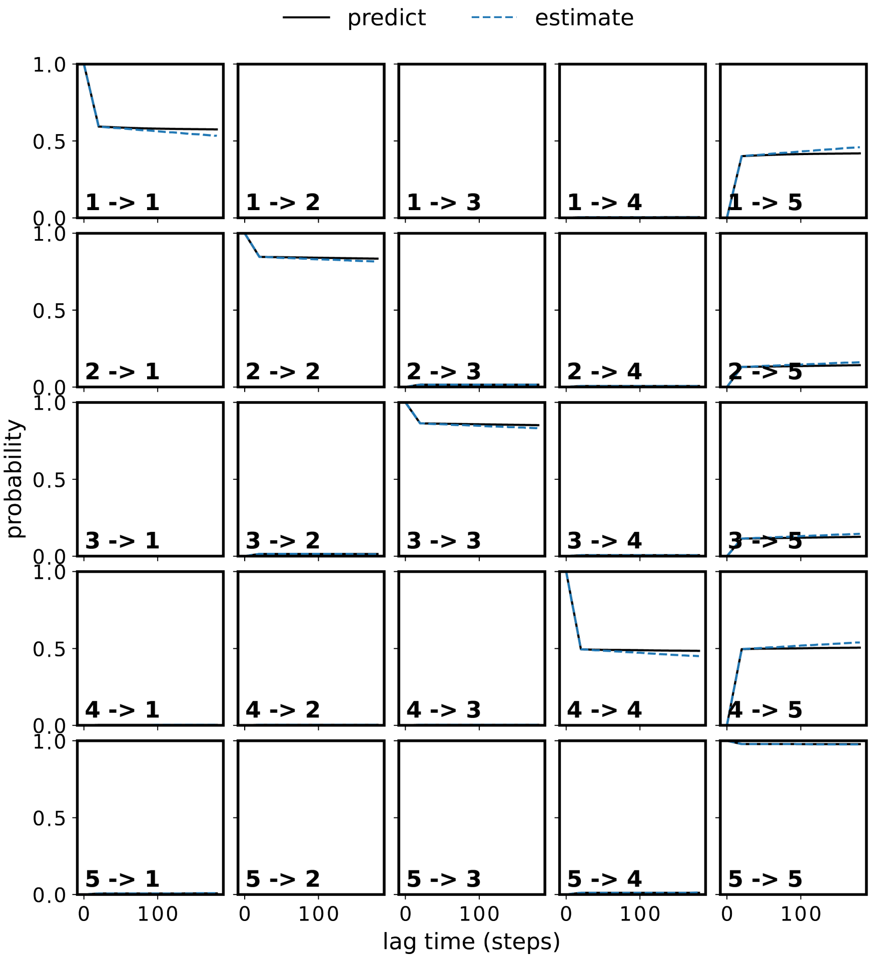


**Supplementary Figure 28:** Chapman-Kolmogorov tests for PROTAC 2 MSM. Source data are provided as a Source Data file.

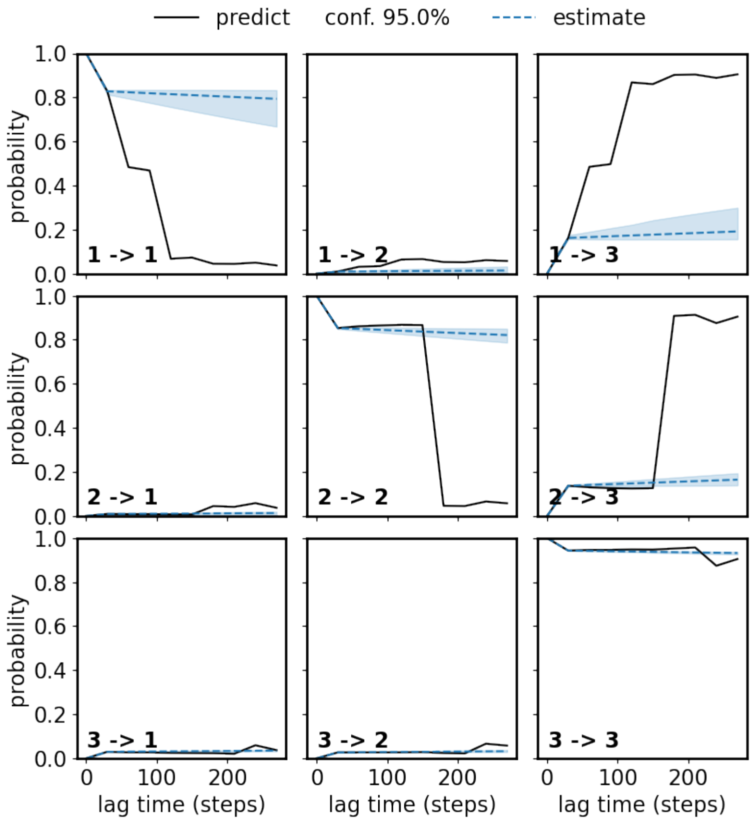


**Supplementary Figure 29:** Chapman-Kolmogorov tests for ACBI1 MSM. Source data are provided as a Source Data file.

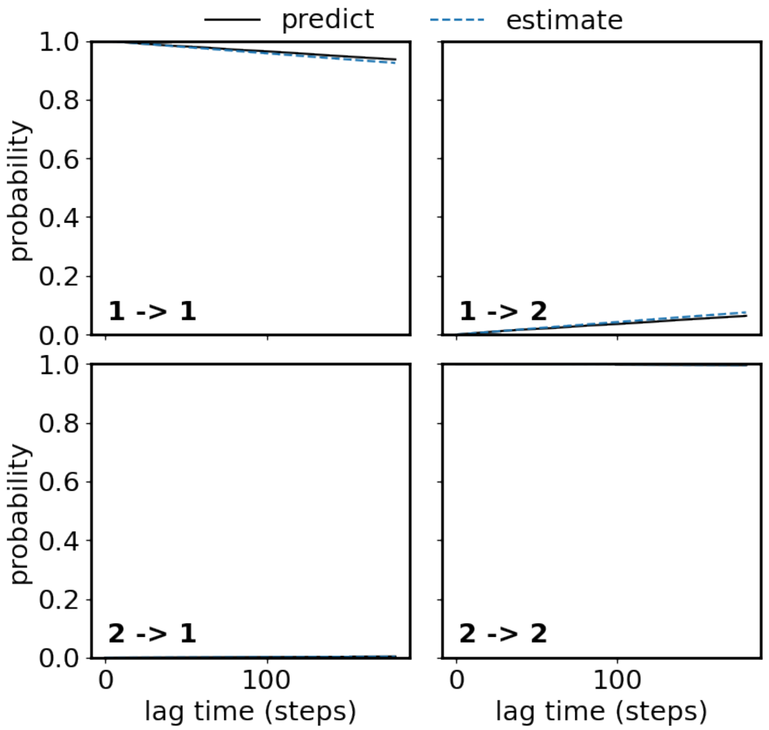


**Supplementary Figure 30:** Chapman-Kolmogorov tests for PROTAC 1 HMM. Source data are provided as a Source Data file.

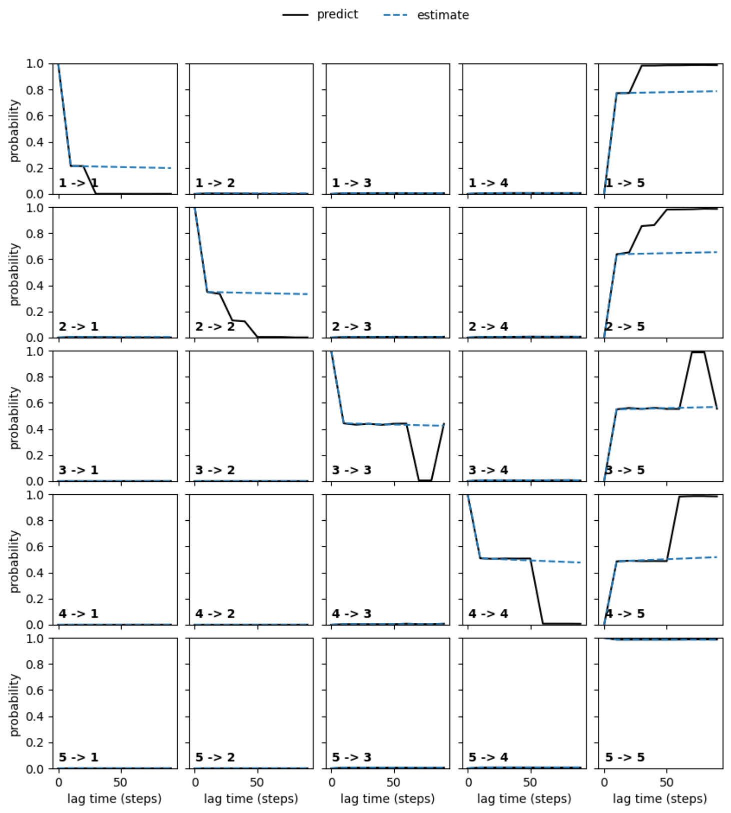


**Supplementary Figure 31:** Chapman-Kolmogorov tests for PROTAC 2 HMM. Source data are provided as a Source Data file.

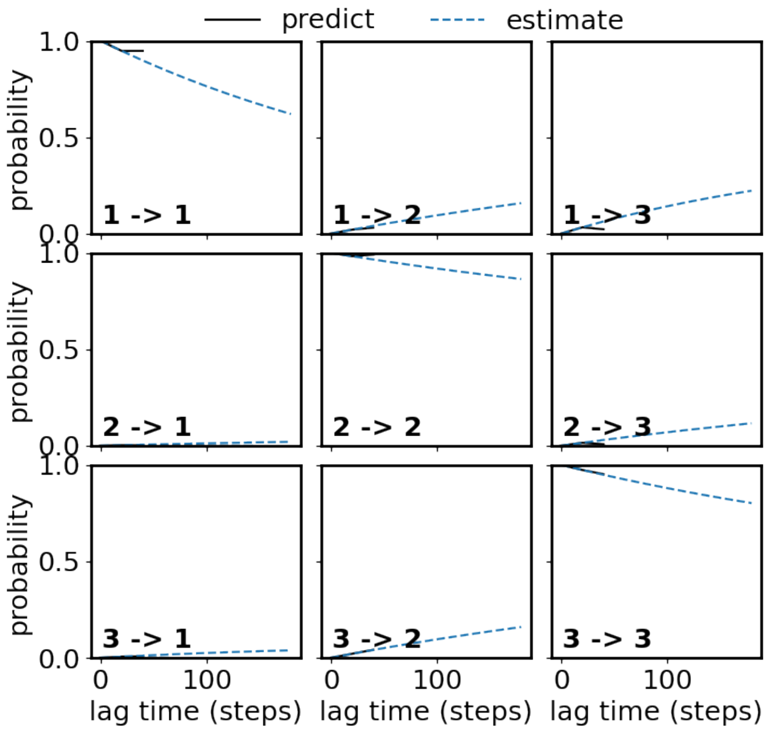


**Supplementary Figure 32:** Chapman-Kolmogorov tests for ACBI1 HMM. Source data are provided as a Source Data file.

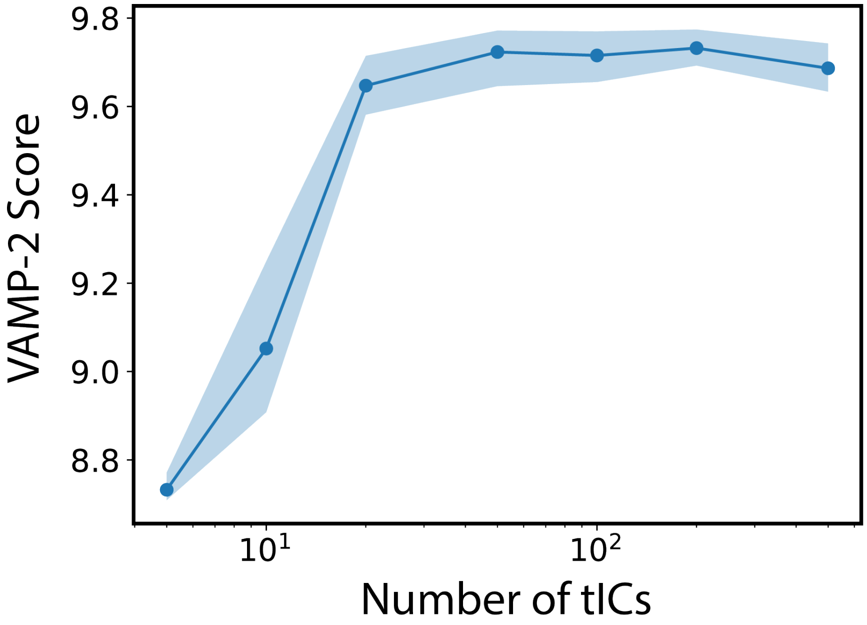


**Supplementary Figure 33:** VAMP-2 score as a function of number of tICs used during fitting of our MSM for PROTAC 2. Beyond ∼50 tICs, the VAMP-2 score saturates. The scores are the arithmetic mean determined from 5 independently fitted MSMs and the shaded region indicates the 90% confidence interval. Source data are provided as a Source Data file.

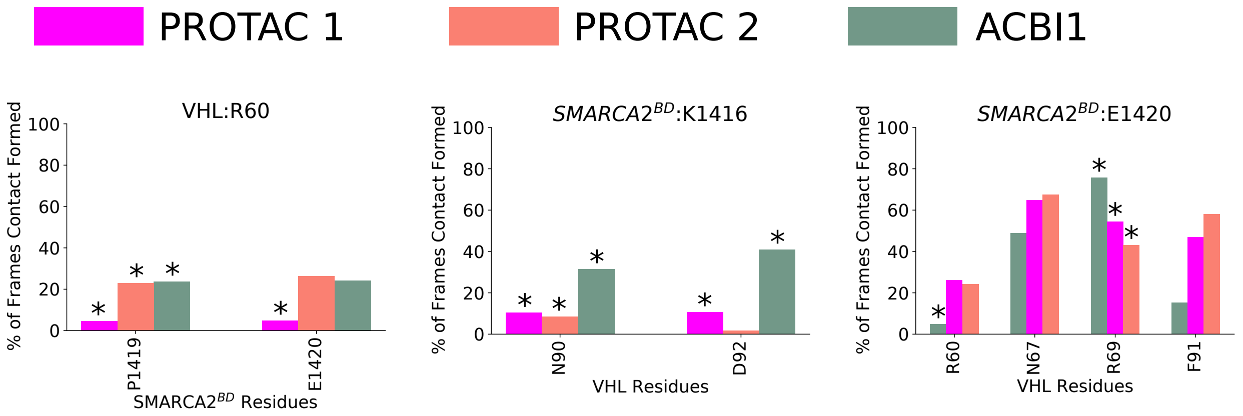


**Supplementary Figure 34:** Comparison of the contacts formed between protected charged interface residues of iso2-SMARCA2$^{BD}$ or VHL, identified in the section Hydrogen-deuterium exchange reveals extended protein-protein interfaces in the main text, and any residue from the opposite protein. A contact is defined as a minimum distance between heavy atoms of $\leq$ 5 Å. Results are shown for contacts that were formed 20% of the time or longer in at least one of the systems with PROTAC 1 (magenta), PROTAC 2 (salmon), or ACBI1 (green). This leaves three distinct charged interface residues, i.e., VHL:R60 (left), SMARCA2$^{BD}$:K1416 (middle), and SMARCA2$^{BD}$:E1420 (right). The asterisks indicate that the specific residue pairs are not observed to form contacts in the corresponding crystal structures. Source data are provided as a Source Data file.

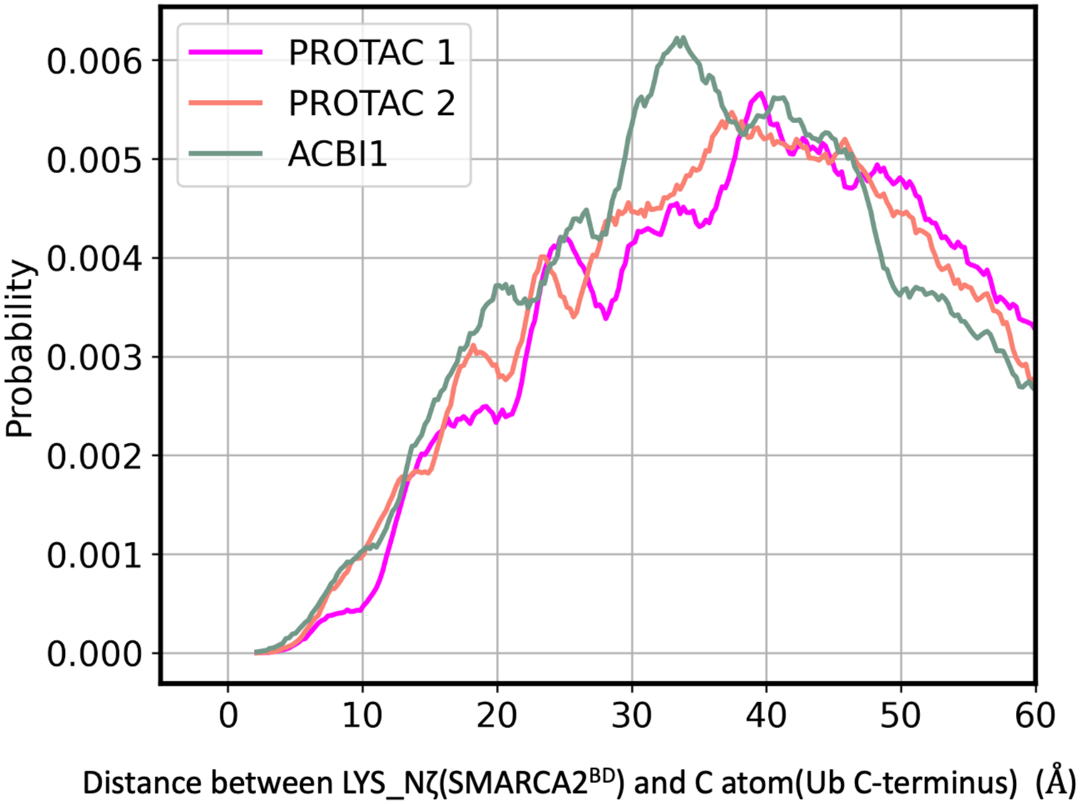


**Supplementary Figure 35:** Probability of distances of lysine residues (side-chain nitrogen atom) from SMARCA2$^{BD}$ to the C-terminal glycine C atom of ubiquitin for the ternary complex crystal structures with three different degraders PROTAC 1, PROTAC 2, and ACBI1. Source data are provided as a Source Data file.

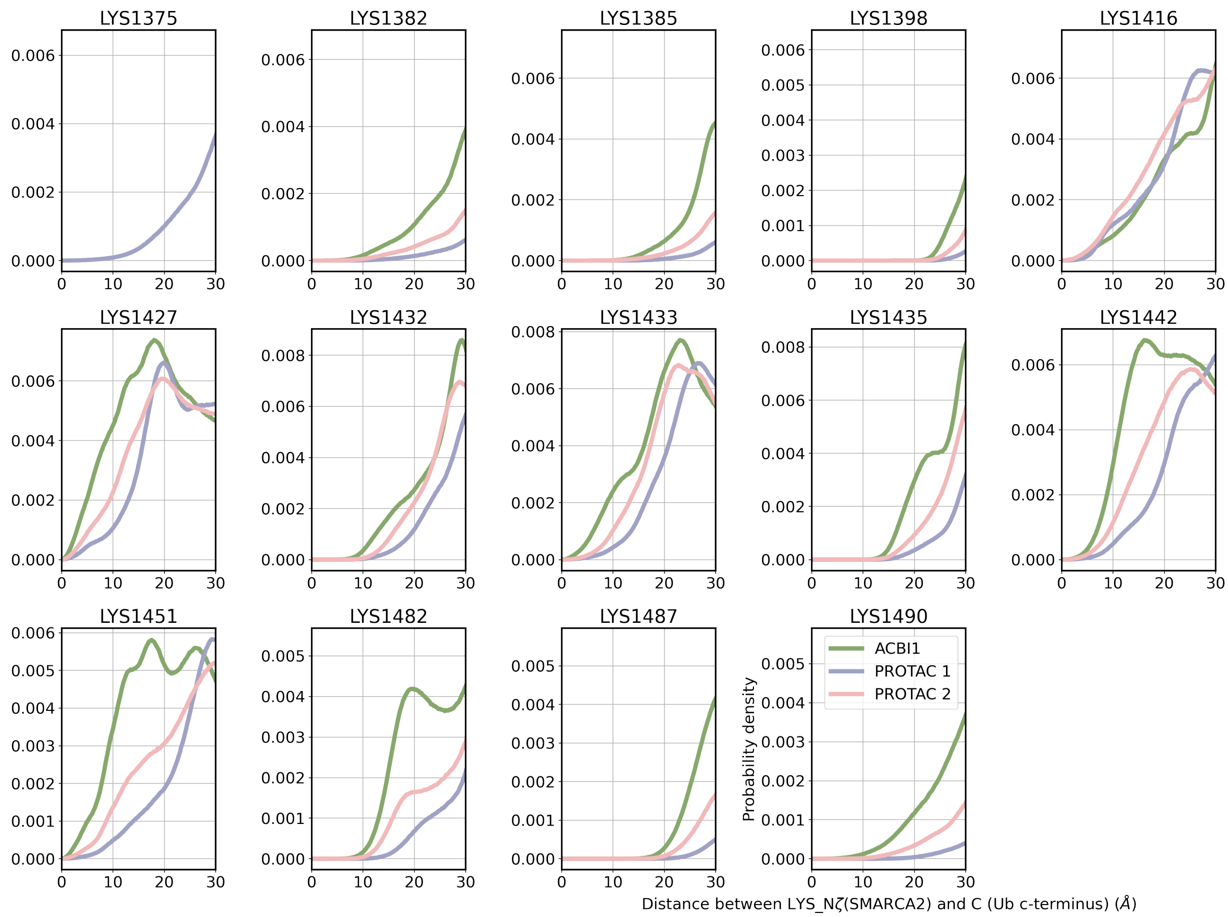


**Supplementary Figure 36:** Probability distribution of individual lysine residue distances from ubiquitin based on HREMD simulations of ternary complexes with PROTAC 1, PROTAC 2, or ACBI1. The crystal structures with PROTAC 2 and ACBI1 lack LYS1375 on SMARCA2$^{BD}$, thus there is only a corresponding distribution for PROTAC 1 (top left panel). Source data are provided as a Source Data file.

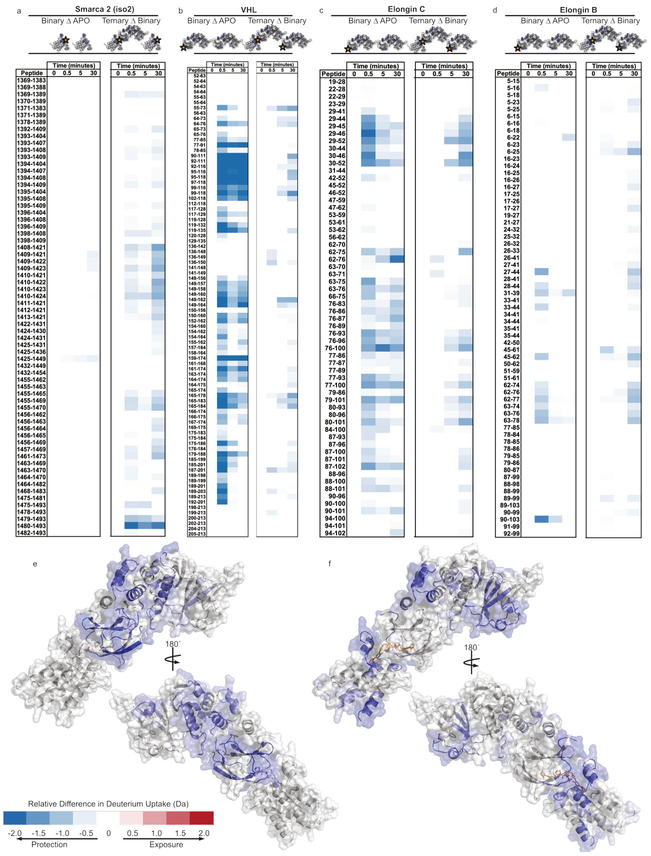


**Supplementary Figure 37:** ACBI1-induced ternary complex formation of SMARCA2$^{\mathrm{BD}}$:VCB leads to protection of specific sites: **a** iso2-SMARCA2$^{\mathrm{BD}}$, **b** VHL, **c** Elongin C, and **d** Elongin B monitored for hydrogen-deuterium exchange over time. The difference plots of each protein are generated by subtracting the deuterium exchange of like peptides of the APO or binary from the binary or ternary states (defined as Binary$\Delta$APO and Ternary$\Delta$Binary), respectively. Regions that exchange significantly less than the comparative state are depicted in blue (negative), whereas regions that exchange significantly more appear in red (positive). The resultant difference plots of the binary, **e**, or ternary complex, **f**, were mapped onto the crystal structure (PDB ID: 7S4E). The experiments were repeated on 2 separate days. All raw relative uptake plots of the deuterium exchange for each state and experiment can be found in Supplementary Data 1.

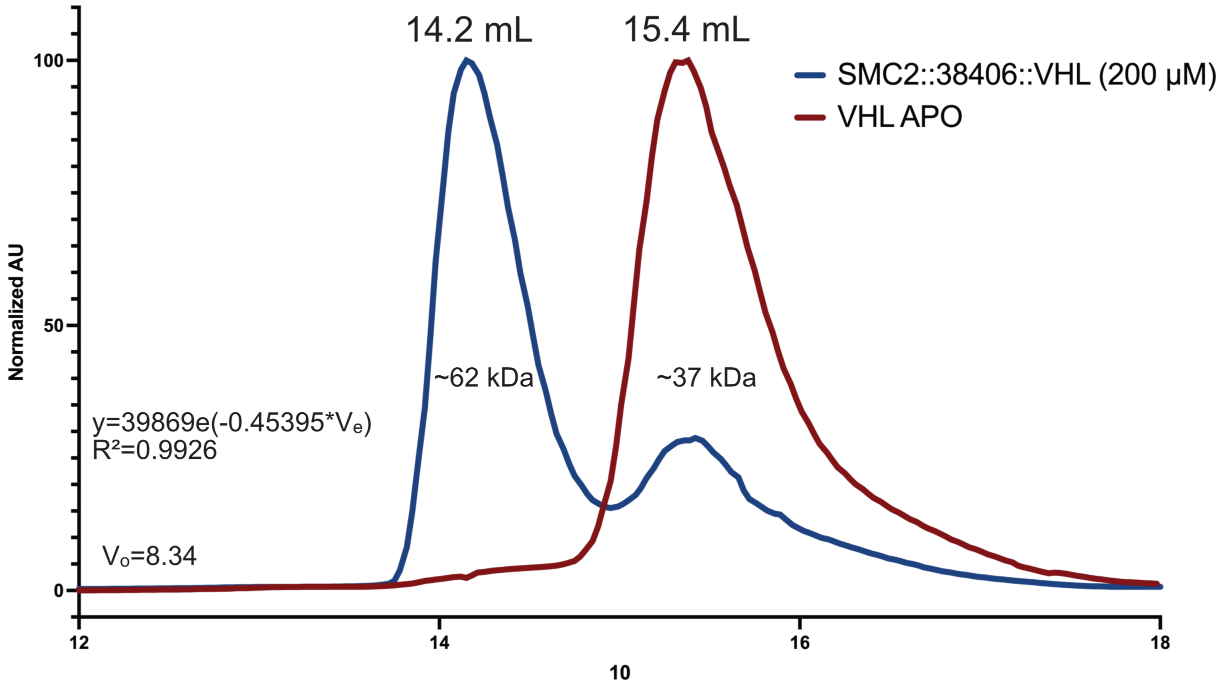


**Supplementary Figure 38:** Analytical Size Exclusion with Superdex 200 GL-10/300 increase (Cytiva), ternary complex of iso2-SMARCA2$^{BD}$:ACBI1:VCB, separation from binary or APO state proteins. Source data are provided as a Source Data file.

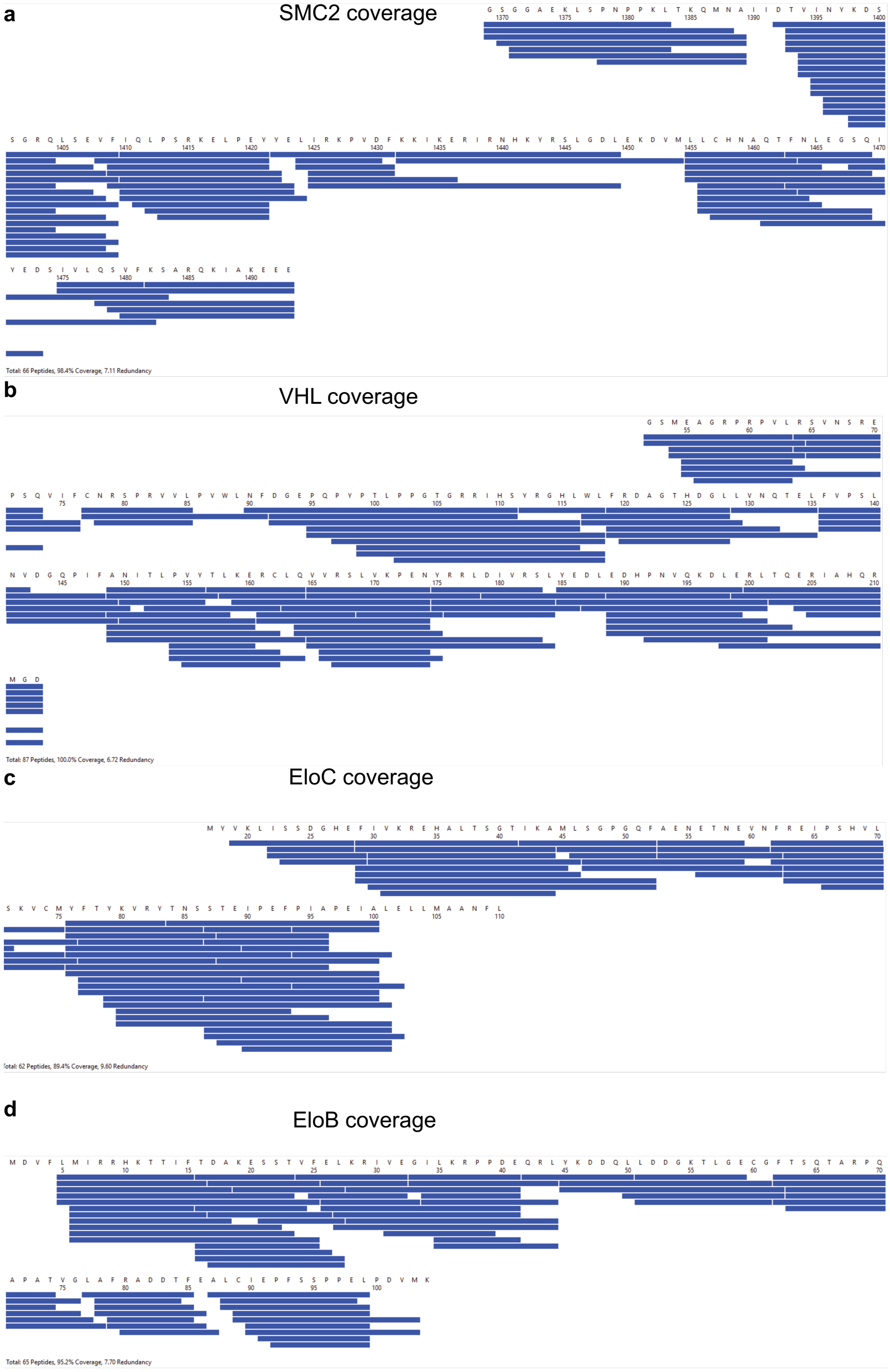


**Supplementary Figure 39:** Peptic coverage map of proteolyzed proteins SMARCA2$^{BD}$, VHL, Elongin C and Elongin B.

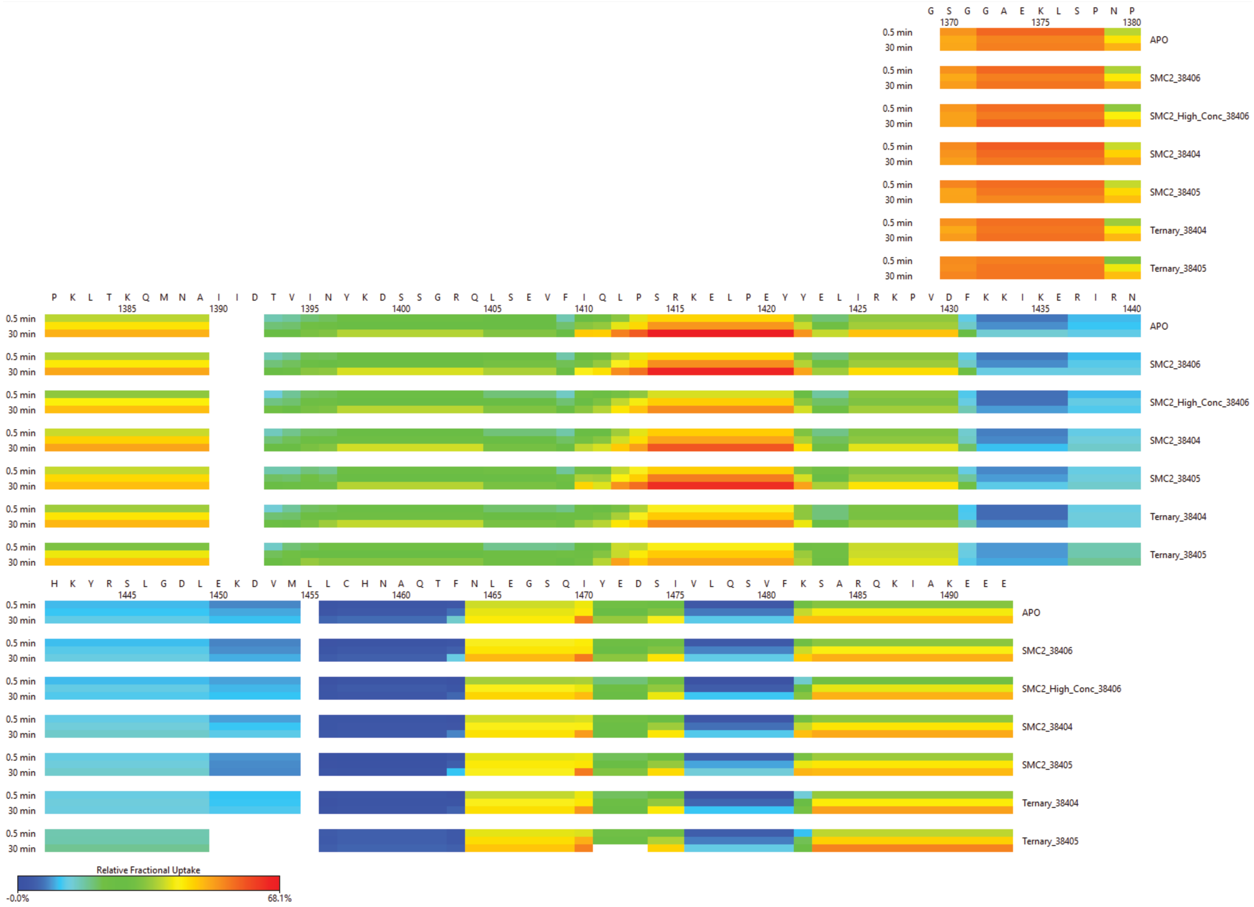


**Supplementary Figure 40:** Relative uptake heat map of HDX-MS exchange data of all PROTAC 1, PROTAC 2, and ACBI1 degraders bound to binary and ternary state iso2-SMARCA2$^{BD}$.

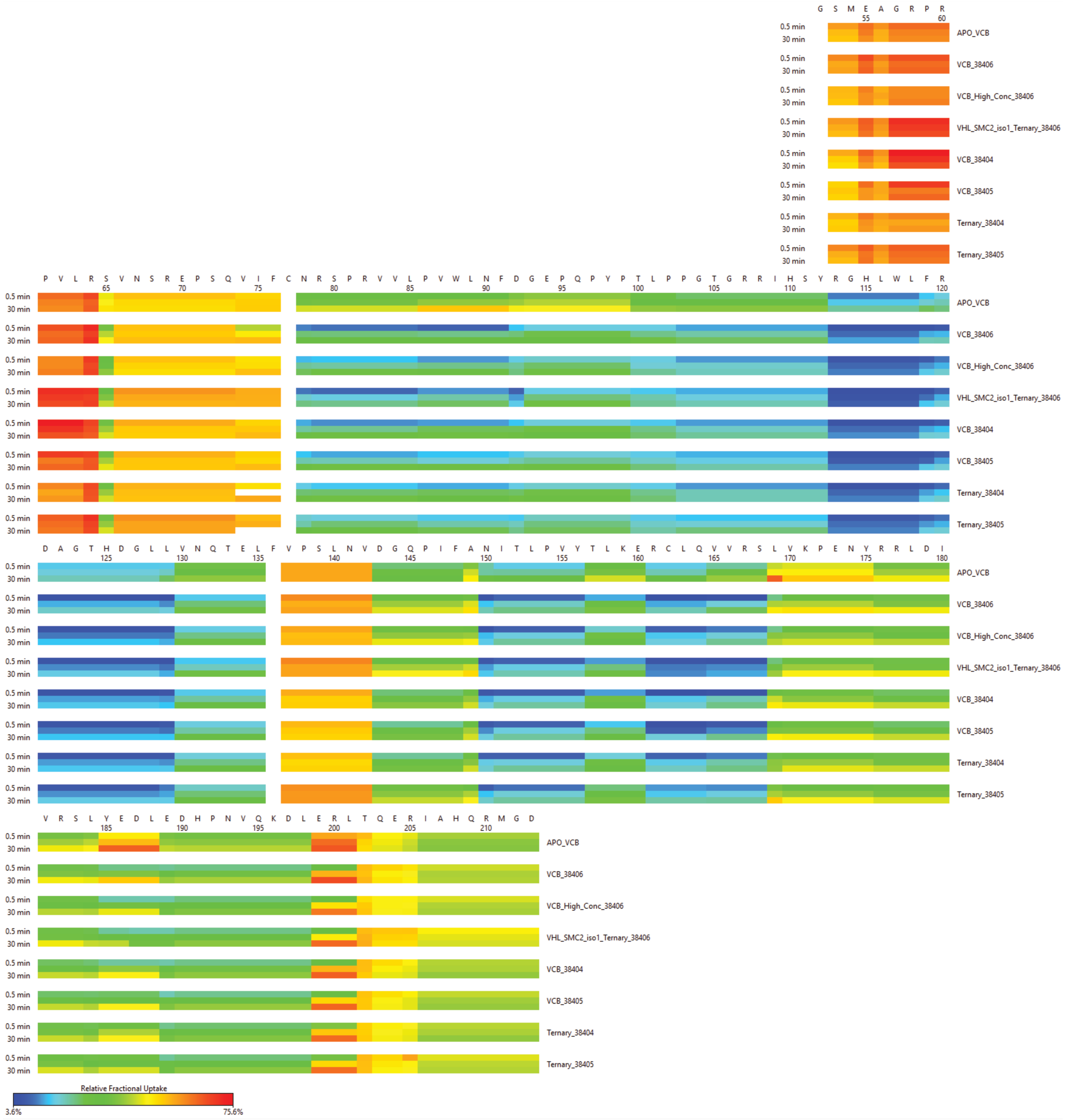

**Supplementary Figure 41:** Relative uptake heat map of HDX-MS exchange data of all PROTAC 1, PROTAC 2, and ACBI1 degraders bound to binary and ternary state VHL.

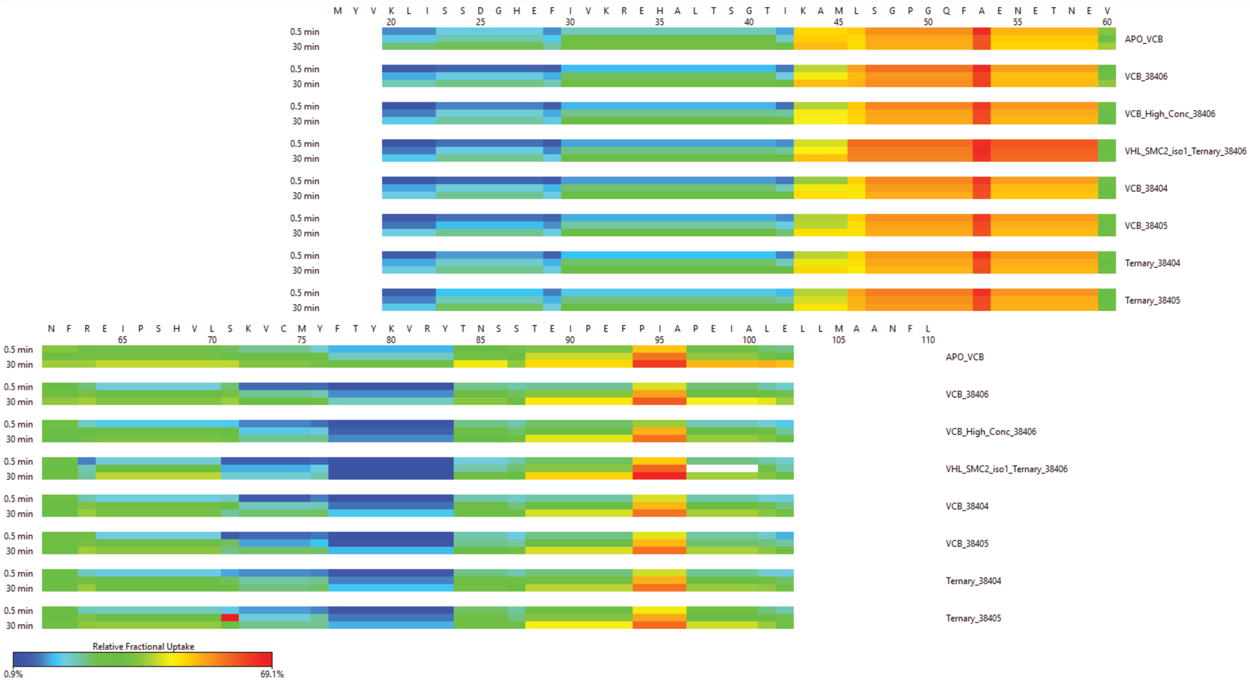


**Supplementary Figure 42:** Relative uptake heat map of HDX-MS exchange data of all PROTAC 1, PROTAC 2, and ACBI1 degraders bound to binary and ternary state Elongin C.

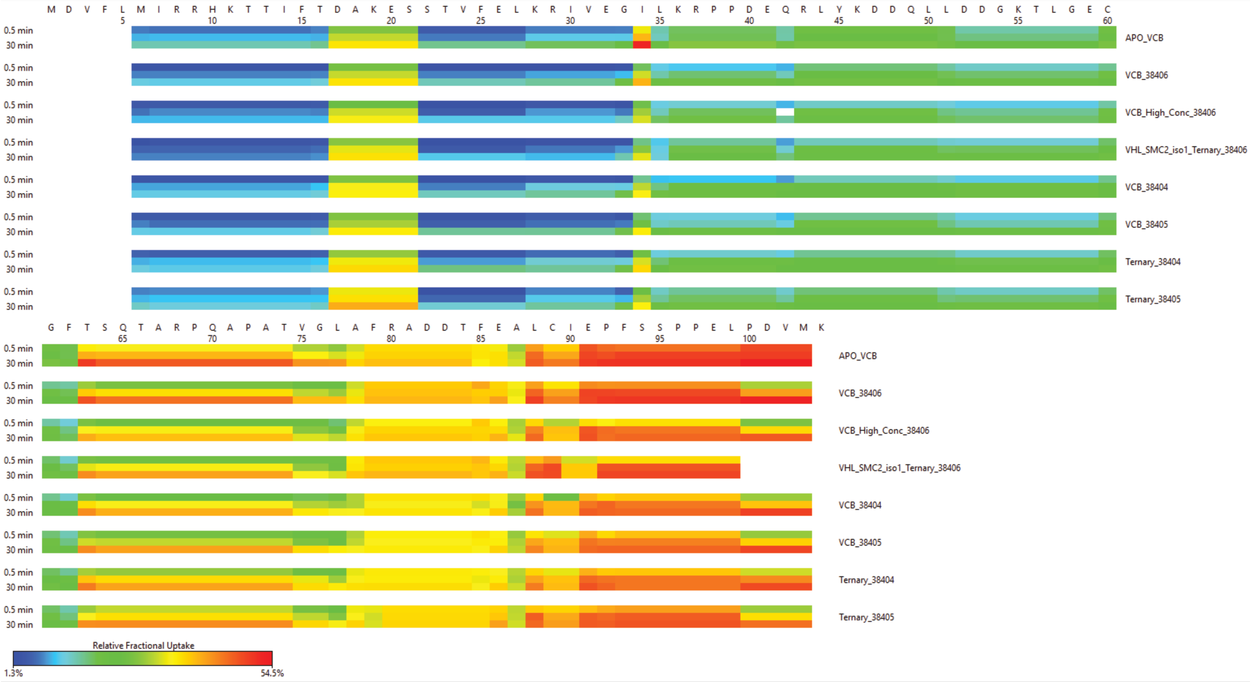


**Supplementary Figure 43:** Relative uptake heat map of HDX-MS exchange data of all PROTAC 1, PROTAC 2, and ACBI1 degraders bound to binary and ternary state Elongin B.

# Supplementary Tables

**Supplementary Table 1:** Binding affinity ($K_D$), efficiencies (IC50, DC50), and cooperativity ($\alpha$) of PROTAC 1, PROTAC 2, and ACBI1 degraders. Ternary IC50 and binary (SMARCA2) DC50 values are reported; the cooperativity is the ratio of binary over ternary IC50. Table adapted from Farnaby et al.[60]

| Degrader | $K_D$, VHL (nM) | $K_D$, SMARCA2 (nM) | IC50 (nM) | DC50 (nM) | Dmax (%) | $\alpha$ |
|---|---|---|---|---|---|---|
| PROTAC 1 | 98 ± 26 | 4500 ± 480 | 205 ± 15 | 300 | 65 | 12 |
| PROTAC 2 | 100 ± 10 | 770 ± 51 | 45 ± 9 | 70 | 90 | 18 |
| ACBI1 | 250 ± 64 | 1800 ± 980 | 26 ± 3 | 6 | ∼100 | 30 |

**Supplementary Table 2:** Crystallographic table for protein crystal structure iso2-SMARCA2[BD]:ACBI1:VHL (PDB ID: 7S4E). Asterisks (*) denote values obtained for the highest-resolution shell.

| | |
|---|---|
| Data collection | |
| Space group | P 21 21 21 |
| Cell dimensions | |
| a, b, c (Å) | 80.14, 116.57, 122.32 |
| $\alpha$, $\beta$, $\gamma$ (°) | 90, 90, 90 |
| Resolution (Å) | 37.89-2.25 (2.31-2.25)* |
| $R_{merge}$ | 0.15 (2.602)* |
| $< \mid \sigma \mid >$ | 8.0 (1.27) |
| CC(1/2) | 0.998 (0.317)* |
| Completeness (%) | 99.9 (99.9)* |
| Redundancy | 7.4 (7.4)* |
| Refinement | |
| Resolution (Å) | 2.25 |
| No. Reflections | 52206 |
| $R_{work}/R_{free}$ | 21.9/25.9 |
| No. Atoms | 7356 |
| Protein | 7070 |
| Ligand/Ions | 196 |
| Water | 90 |
| B factors | |
| Protein | 61.19 |
| Ligand/Ions | 57.66 |
| Water | 53.56 |
| R.M.S. deviations | |
| Bond length (Å) | 0.009 |
| Bond angles (°) | 1.519 |
| Ramachandran favored (%) | 96.73 |
| Ramachandran outliers (%) | 0.00 |
| Clashscore | 3.64 |

**Supplementary Table 3:** HDX data summary for iso2-SMARCA2 (binary).

| Dataset | iso2-SMARCA2 (binary) | | | |
|---|---|---|---|---|
| | APO | ACBI1 | PROTAC 1 | PROTAC 2 |
| HDX reaction retails | 93% $D_2O$, Labeling pH (read): 7.0, pD (assuming +0.4): 7.4<br>Quench pH: 2.5, Labeling Temperature: 10°C, Quench Temperature: 1°C | | | |
| HDX time course | 0s, 30s, 300s, 1800s | | | |
| HDX controls | Relative comparison of same protein,<br>APO used as control measured against liganded states | | | |
| Back-exchange | Back exchange not calculated for comparison of alternative states of the same protein, thus results are reported as Relative Deuterium Exchange | | | |
| Number of Peptides | 66 | | | |
| Sequence coverage | 98.4% | | | |
| Redundancy | 7.11 | | | |
| Replicates | 2 (technical) | | | |
| Average peptide length | 11.5 | | | |
| Repeatability | 0.085 | 0.118 | 0.149 | 0.118 |
| Significant differences in HDX | | 0.382 D (98.99%) | | |

**Supplementary Table 4:** HDX data summary for VHL/EloB/EloC (binary).

| Dataset | VHL/EloB/EloC (binary) | | | |
|---|---|---|---|---|
| | APO | ACBI1 | PROTAC 1 | PROTAC 2 |
| HDX reaction retails | 93% $D_2O$, Labeling pH (read): 7.0, pD (assuming +0.4): 7.4<br>Quench pH: 2.5, Labeling Temperature: 10°C, Quench Temperature: 1°C | | | |
| HDX time course | 0s, 30s, 300s, 1800s | | | |
| HDX controls | Relative comparison of same protein,<br>APO used as control measured against liganded states | | | |
| Back-exchange | Back exchange not calculated for comparison of alternative states of the same protein, thus results are reported as Relative Deuterium Exchange | | | |
| Number of Peptides | 87/65/62 | | | |
| Sequence coverage | 100%/95.2%/89.4% | | | |
| Redundancy | 6.72/7.70/9.60 | | | |
| Replicates | 2 (technical) | | | |
| Average peptide length | 10.3/10.9/9.7 | | | |
| Repeatability | 0.197/0.211/0.143 | 0.109/0.160/0.076 | 0.158/0.166/0.126 | 0.158/0.192/0.182 |
| Significant differences in HDX | | 0.451 D/0.411 D/ 0.384 D (95%) | 0.537 D/0.482 D/ 0.422 D (95%) | 0.542 D/0.459 D/ 0.442 D (95%) |

**Supplementary Table 5:** HDX data summary for iso2-SMARCA2/VHL/EloB/EloC (ternary).

| Dataset | iso2-SMARCA2/VHL/EloB/EloC (ternary) | | |
|---|---|---|---|
| | ACBI1 | PROTAC 1 | PROTAC 2 |
| HDX reaction retails | 93% $D_2O$, Labeling pH (read): 7.0, pD (assuming +0.4): 7.4<br>Quench pH: 2.5, Labeling Temperature: 10°C, Quench Temperature: 1°C | | |
| HDX time course | 0s, 30s, 300s, 1800s | | |
| HDX controls | Relative comparison of same protein,<br>APO used as control measured against liganded states | | |
| Back-exchange | Back exchange not calculated for comparison of alternative states of the same protein, thus results are reported as Relative Deuterium Exchange | | |
| Number of Peptides | Same as binary (see Supplementary Tables 3 & 4) | | |
| Sequence coverage | Same as binary (see Supplementary Tables 3 & 4) | | |
| Redundancy | Same as binary (see Supplementary Tables 3 & 4) | | |
| Replicates | 2 (technical) | | |
| Average peptide length | Same as binary (see Supplementary Tables 3 & 4) | | |
| Repeatability | 0.130/0.190/0.201/0.230 | 0.126/0.182/0.163/0.235 | 0.154/0.130/0.274/0.105 |
| Significant differences in HDX | 0.391 D (98.99%)/0.455 D/<br>0.419 D/0.381 D (95%) | 0.329 D/0.377 D/<br>0.355 D/0.350 D (95%) | 0.579 D/0.371 D/<br>0.355 D/0.350D (95%) |

**Supplementary Table 6:** HDX data summary iso1-SMARCA2 (binary).

| Dataset | iso1-SMARCA2 (binary) | |
|---|---|---|
| | APO | ACBI1 |
| HDX reaction retails | 93% $D_2O$, Labeling pH (read): 7.0, pD (assuming +0.4): 7.4<br>Quench pH: 2.5, Labeling Temperature: 10°C, Quench Temperature: 1°C | |
| HDX time course | 0s, 30s, 300s, 1800s | |
| HDX controls | Relative comparison of same protein,<br>APO used as control measured against liganded states | |
| Back-exchange | Back exchange not calculated for comparison of alternative states of the same protein, thus results are reported as Relative Deuterium Exchange | |
| Number of Peptides | 68 | |
| Sequence coverage | 96.5% | |
| Redundancy | 6.54 | |
| Replicates | 2 (technical) | |
| Average peptide length | 11.5 | |
| Repeatability | 0.149 | 0.069 |
| Significant differences in HDX | 0.367 D (98.99%) | |

**Supplementary Table 7:** HDX data summary iso1-SMARCA2/VHL/EloB/EloC (ternary).

| Dataset | iso1-SMARCA2/VHL/EloB/EloC (ternary)<br>ACBI1 |
|---|---|
| HDX reaction retails | 93% $D_2O$, Labeling pH (read): 7.0, pD (assuming +0.4): 7.4<br>Quench pH: 2.5, Labeling Temperature: 10°C, Quench Temperature: 1°C |
| HDX time course | 0s, 30s, 300s, 1800s |
| HDX controls | Relative comparison of same protein,<br>APO used as control measured against liganded states |
| Back-exchange | Back exchange not calculated for comparison of alternative states of the same protein, thus results are reported as Relative Deuterium Exchange |
| Number of Peptides | Same as binary (see Supplementary Tables 6 & 4) |
| Sequence coverage | Same as binary (see Supplementary Tables 6 & 4) |
| Redundancy | Same as binary (see Supplementary Tables 6 & 4) |
| Replicates | 2 (technical) |
| Average peptide length | Same as binary (see Supplementary Tables 6 & 4) |
| Repeatability | 0.128/0.165/0.158/0.102 |
| Significant differences in HDX | 0.372 D/0.487 D/<br>0.460 D (98.99%)/0.273 (95%) |

**Supplementary Table 8:** Protected residues on VHL and SMARCA2$^{BD}$ used in the WE-HDX simulations.

| Protein | Protected residues |
|---|---|
| VHL | R60, V62, L63, R64, S65, V66, N67, S68, R69, E70, S72, Q73 |
| SMARCA2$^{BD}$ | F1409, I1410, Q1411, L1412, S1414, R1415, K1416, E1417, L1418, E1420, Y1421, Y1422, E1423, L1424, L1456, C1457, H1458, N1459, A1460, Q1461, T1462, F1463, N1464, L1465, E1466, G1467, S1468, Q1469, I1470 |

**Supplementary Table 9:** Comparison of ternary complex binding rate constants between simulation and experiment for the PROTAC 1, PROTAC 2, and ACBI1 systems. The experimental rate for PROTAC 2 has not been determined yet. The reported predicted binding rates are averaged as a function of time over all replicate simulations as shown in Figure 3c in the main text. The final values are reported here.

| Degrader | Predicted rate ($M^{-1}s^{-1}$) | Experimental rate ($M^{-1}s^{-1}$) |
|---|---|---|
| PROTAC 1 | $9.6 * 10^5 \pm 7.8 * 10^5$ | $2.9 * 10^5$ |
| PROTAC 2 | $1.8 * 10^2 \pm 1.7 * 10^2$ | N/A |
| ACBI1 | $2.9 * 10^5 \pm 2.4 * 10^5$ | $2.4 * 10^5$ |

**Supplementary Table 10:** Distributions of predicted docking structures over the CAPRI quality categories (PDB ID: 6HAX). The 35 docking runs have been performed both with and without HDX-derived restraints to assure reproducibility of the results. The classification of predictions into the quality categories was done with the conventional CAPRI criteria. In the columns: *mean* is the mean number of models in a given category estimated over the 35 runs; *CI* is the Confidence Interval, i.e. the range within which the *mean* value will be found with the probability 0.95.

| | | High | | Medium | | Acceptable | | Incorrect | |
|---|---|---|---|---|---|---|---|---|---|
| | | mean | CI | mean | CI | mean | CI | mean | CI |
| Top-10 | no HDX | 0.0 | [0.0, 0.0] | 0.0 | [0.0, 0.1] | 0.3 | [0.1, 0.5] | 8.6 | [8.4, 8.8] |
| | with HDX | 0.9 | [0.6, 1.3] | 2.2 | [1.8, 2.6] | 2.7 | [2.3, 3.1] | 3.2 | [2.7, 3.7] |
| Top-50 | no HDX | 0.0 | [0.0, 0.0] | 0.4 | [0.2, 0.6] | 6.0 | [5.4, 6.8] | 43 | [42, 43] |
| | with HDX | 3.8 | [3.1, 4.6] | 13 | [12, 14] | 16 | [15, 17] | 16 | [15, 17] |
| Top-100 | no HDX | 0.4 | [0.1, 0.8] | 3.5 | [2.8, 4.2] | 21 | [19, 22] | 74 | [73, 76] |
| | with HDX | 6.8 | [6.0, 7.7] | 30 | [28, 31] | 30 | [29, 32] | 32 | [31, 34] |

**Supplementary Table 11:** Structural parameters from the SAXS data acquisition.

| Sample details | | |
|---|---|---|
| | iso1-SMARCA2:ACBI1:VCB | iso2-SMARCA2:ACBI1:VCB |
| Organism | *Homo sapiens* | *Homo sapiens* |
| Source | *E. coli* expression | *E. coli* expression |
| Uniprot sequence ID | *SMARCA2 (isoform 1)*: P51531-1 (residues 1373-1511)<br>*VHL*: P40337 (residues 54-213)<br>*EloB*: Q15370 (residues 1-104)<br>*EloC*: Q15369 (residues 17-112) | *SMARCA2 (isoform 2)*: P51531-2 (residues 1373-1511, delete 1400-1417)<br>*VHL*: P40337 (residues 54-213)<br>*EloB*: Q15370 (residues 1-104)<br>*EloC*: Q15369 (residues 17-112) |
| Molecular weight (Da) | iso1-SMARCA2: 16395.69 Da<br>VHL: 18676.3 Da<br>EloB: 11733.42 Da<br>EloC: 10832.45 Da<br>ACBI1: 936.12 Da | iso2-SMARCA2: 14397.5 Da<br>VHL: 18676.3 Da<br>EloB: 11733.42 Da<br>EloC: 10832.45 Da<br>ACBI1: 936.12 Da |
| Guinier analysis | | |
| I(0) ($cm^{-1}$) | 0.0251 ± 0.0002 | 0.0188 ± 0.0001 |
| $R_g$ (Å) | 33.3 ± 0.3 | 32.2 ± 0.3 |
| q-range ($Å^{-1}$) | 0.0160 - 0.0388 | 0.0134 - 0.0401 |
| q*$R_g$ max | 1.2904 | 1.297 |
| $R^2$ | 0.9938 | 0.9902 |
| Molecular weight estimation (kDa) | | |
| Vc[61] | 57.8 | 51.8 |
| Vp[62] | 64.8 | 58.0 |
| Bayesian inference[63] | 58.1 | 49.8 |
| Shape & size[64] | 61.6 | 54.7 |
| P(r) analysis (GNOM) | | |
| I(0) ($cm^{-1}$) | 0.0253 ± 0.0002 | 0.0191 ± 0.0001 |
| $R_g$ (Å) | 34.3 ± 0.3 | 33.2 ± 0.2 |
| Dmax (Å) | 125 | 111 |
| q-range ($Å^{-1}$) | 0.0160 - 0.3494 | 0.0134 - 0.3985 |
| $\chi^2$ | 0.8055 | 0.9071 |
| Total estimate | 0.8335 | 0.841 |

**Supplementary Table 12:** SAXS data-collection parameters.

| | |
|---|---|
| Instrument/Data processing | BioXolver L SAXS (Xenocs)/BioXTAS RAW 2.0.3 |
| Wavelength (Å) | 1.34 |
| Beam size ($\mu$M) | 80 x 20 |
| q measurement range (Å$^{-1}$) | 0.0134 - 0.5793 |
| Exposure time | Continous 60 sec data-frame measurements |
| Sample temperature | 20°C |
| Detector | Dectris PILATUS3 R 300K |
| SEC-SAXS column | Superdex 200 increase 10/300 on GE Healthcare ÄKTAmicro |
| Loading concentration | 275 $\mu$M : 275 $\mu$M : 275 $\mu$M (SMARCA2:ACBI1:VCB) |
| Injection volume | 500 $\mu$L |
| Flow rate | 0.05 mL/min |
| Solvent (buffer frames taken before the elution peak) | 20 mM HEPES, pH 7.5, 150 mM NaCl and 1mM DTT |

**Supplementary Table 13:** Details of HREMD simulations. Protein complexes, number of atoms in a simulation box, number of replicas used and the aggregate length of the simulations are listed.

| ID | Complex | # of atoms | # of replicas | Aggregate length ($\mu$s) |
|---|---|---|---|---|
| Sys1 | iso1-SMARCA2$^{BD}$:ACBI1:VHL | 116,254 | 20 | 10 |
| Sys2 | iso1-SMARCA2$^{BD}$:ACBI1:VCB | 220,573 | 24 | 12 |
| Sys3 | iso2-SMARCA2$^{BD}$:ACBI1:VHL | 117,256 | 20 | 10 |
| Sys4 | iso2-SMARCA2$^{BD}$:ACBI1:VCB | 234,724 | 24 | 12 |
| Sys5 | iso2-SMARCA2$^{BD}$:PROTAC 1:VHL | 137,347 | 20 | 10 |
| Sys6 | iso1-SMARCA2$^{BD}$:PROTAC 2:VHL | 69,696 | 20 | 10 |
| Sys7 | iso2-SMARCA2$^{BD}$:PROTAC 2:VHL | 68,820 | 20 | 10 |
| Sys8 | iso2-SMARCA2$^{BD}$:PROTAC 2:VCB | 119,082 | 24 | 12 |

**Supplementary Table 14:** SMARCA2 lysine residues identified as ubiquitinated upon ACBI1 treatment. The change in abundance of ACBI1-treated ubiquitination levels compared to the vehicle (DMSO) treated sample are shown with associated Benjamini-Hochberg FDR corrected p-values. A pairwise t-test, with Benjamini-Hochberg mutiple comparison, was used for comparing two conditions in triplicate measurements. The residues (or sites) marked with an asterisk (*) are shared sites with SMARCA4 protein. All sites are for iso2-SMARCA2, unless otherwise noted.

| Ubiquitination site | $Log_2$ Fold Change | p-value (BH corrected) |
|---|---|---|
| K381 | 0.02 | 0.97 |
| K413 | 1.03 | 0.03 |
| K460* | 1.94 | 0.32 |
| K672 | 0.56 | 0.21 |
| K822* | 3.35 | 0.03 |
| K984 | 0.66 | 0.26 |
| K996 | 0.32 | 0.29 |
| K1051* | 2.89 | 0.02 |
| K1197* | 3.30 | 0.06 |
| K1197/K1207* | 3.95 | 0.02 |
| K1207* | 4.23 | 0.02 |
| K1286* | 2.22 | 0.38 |
| K1323 | 3.73 | 0.00 |
| K1358 | 1.01 | 0.04 |
| K1375 | 1.94 | 0.00 |
| K1385 | 1.08 | 0.12 |
| iso1-SMC2:K1398 | 0.98 | 0.08 |
| K1398 | 4.03 | 0.00 |
| K1416* | 5.97 | 0.00 |

**Supplementary Table 15:** Movies rendered from MD simulation trajectories.

| **Movie filename** | **Download link** |
|---|---|
| wes_6hax_ternary_complex_formation.mp4 | Click here |
| hremd_6HAX.mp4 | Click here |
| hremd_6HAY.mp4 | Click here |
| hremd_7S4E.mp4 | Click here |
| hremd_iso1_smrc2_acbi1_vhl.mp4 | Click here |
| meta-eabf_CRL_SMARCA2$^{BD}$_ACBI1_VHL_25to40Angs.mp4 | Click here |
| meta-eabf_CRL_SMARCA2$^{BD}$_ACBI1_VHL_25to75Angs.mp4 | Click here |

# Supplementary References

(1) Donyapour, N., Roussey, N. M. & Dickson, A. Revo: Resampling of ensembles by variation optimization. *Journal of Chemical Physics* **150** (2019).

(2) Saglam, A. S. & Chong, L. T. Protein–protein binding pathways and calculations of rate constants using fully-continuous, explicit-solvent simulations. *Chemical Science* **10**, 2360–2372, (2018).

(3) Drummond, M. L., Henry, A., Li, H. & Williams, C. I. Improved Accuracy for Modeling PROTAC-Mediated Ternary Complex Formation and Targeted Protein Degradation via New In Silico Methodologies. *Journal of Chemical Information and Modeling* **60**, 5234–5254, (2020).

(4) Devaurs, D., Antunes, D. A. & Borysik, A. J. Computational modeling of molecular structures guided by hydrogen-exchange data. *Journal of the American Society for Mass Spectrometry* **33**, 215–237, (2022).

(5) Anand, G. S. *et al.* Identification of the protein kinase A regulatory R I $\alpha$-catalytic subunit interface by amide H/ 2 H exchange and protein docking. *Proceedings of the National Academy of Sciences* **100**, 13264–13269, (2003).

(6) Zhang, M. M. *et al.* An integrated approach for determining a protein–protein binding interface in solution and an evaluation of hydrogen–deuterium exchange kinetics for adjudicating candidate docking models. *Analytical Chemistry* **91**, 15709–15717, (2019).

(7) Rampler, E. *et al.* Comprehensive Cross-Linking Mass Spectrometry Reveals Parallel Orientation and Flexible Conformations of Plant HOP2-MND1. *Journal of Proteome Research* **14**, 5048–62, (2015).

(8) Lin, S.-J. *et al.* Structural Insights to the Heterotetrameric Interaction between the

Vibrio parahaemolyticus PirAvp and PirBvp Toxins and Activation of the Cry-Like Pore-Forming Domain. *Toxins* **11**, 233, (2019).

(9) Pandit, D. *et al.* Mapping of discontinuous conformational epitopes by amide hydrogen/deuterium exchange mass spectrometry and computational docking. *Journal of Molecular Recognition* **25**, 114–24, (2012).

(10) Roberts, V. A., Pique, M. E., Hsu, S. & Li, S. Combining H/D Exchange Mass Spectrometry and Computational Docking To Derive the Structure of Protein–Protein Complexes. *Biochemistry* **56**, 6329–6342, (2017).

(11) Rey, M. *et al.* Mass Spec Studio for Integrative Structural Biology. *Structure* **22**, 1538–1548, (2014).

(12) Merkle, P. S. *et al.* The T-Cell Receptor Can Bind to the Peptide-Bound Major Histocompatibility Complex and Uncomplexed $\beta$ 2 -Microglobulin through Distinct Binding Sites. *Biochemistry* **56**, 3945–3961, (2017).

(13) Komolov, K. E. *et al.* Structural and Functional Analysis of a $\beta$ 2 -Adrenergic Receptor Complex with GRK5. *Cell* **169**, 407–421.e16, (2017).

(14) Eron, S. J. *et al.* Structural Characterization of Degrader-Induced Ternary Complexes Using Hydrogen–Deuterium Exchange Mass Spectrometry and Computational Modeling: Implications for Structure-Based Design. *ACS Chemical Biology* (2021).

(15) Brodie, N. I., Popov, K. I., Petrotchenko, E. V., Dokholyan, N. V. & Borchers, C. H. Solving protein structures using short-distance cross-linking constraints as a guide for discrete molecular dynamics simulations. *Science Advances* **3**, e1700479, (2017).

(16) Marsh, J. A. & Forman-Kay, J. D. Structure and disorder in an unfolded state under nondenaturing conditions from ensemble models consistent with a large number of experimental restraints. *Journal of Molecular Biology* **391**, 359–74, (2009).

(17) Martens, C., Shekhar, M., Lau, A. M., Tajkhorshid, E. & Politis, A. Integrating hydrogen–deuterium exchange mass spectrometry with molecular dynamics simulations to probe lipid-modulated conformational changes in membrane proteins. *Nature Protocols* **14**, 3183–3204, (2019).

(18) Jia, R. *et al.* Hydrogen-deuterium exchange mass spectrometry captures distinct dynamics upon substrate and inhibitor binding to a transporter. *Nature Communications* **11**, 6162, (2020).

(19) Zhang, H. *et al.* Structure of the full-length glucagon class B G-protein-coupled receptor. *Nature* **546**, 259–264, (2017).

(20) Harrison, R. A. *et al.* Structural Dynamics in Ras and Related Proteins upon Nucleotide Switching. *Journal of Molecular Biology* **428**, 4723–4735, (2016).

(21) Xiao, Y., Shaw, G. S. & Konermann, L. Calcium-Mediated Control of S100 Proteins: Allosteric Communication via an Agitator/Signal Blocking Mechanism. *Journal of the American Chemical Society* **139**, 11460–11470, (2017).

(22) Singh, J. & Udgaonkar, J. B. Unraveling the Molecular Mechanism of pH-Induced Misfolding and Oligomerization of the Prion Protein. *Journal of Molecular Biology* **428**, 1345–1355, (2016).

(23) Petruk, A. A. *et al.* Molecular Dynamics Simulations Provide Atomistic Insight into Hydrogen Exchange Mass Spectrometry Experiments. *Journal of Chemical Theory and Computation* **9**, 658–669, (2012).

(24) Shan, Y., Arkhipov, A., Kim, E. T., Pan, A. C. & Shaw, D. E. Transitions to catalytically inactive conformations in EGFR kinase. *Proceedings of the National Academy of Sciences* **110**, 7270–7275, (2013).

(25) Huang, L., So, P.-K. & Yao, Z.-P. Protein Dynamics Revealed by Hydrogen Deuterium Exchange Mass Spectrometry: Correlation between Experiments and Simulation. *Rapid Communications in Mass Spectrometry* **33**, 83–89, (2018).

(26) Sheinerman, F. B. & Brooks, C. L. Molecular picture of folding of a small $\alpha/\beta$ protein. *Proceedings of the National Academy of Sciences* **95**, 1562–1567, (1998).

(27) McAllister, R. G. & Konermann, L. Challenges in the Interpretation of Protein H/D Exchange Data: A Molecular Dynamics Simulation Perspective. *Biochemistry* **54**, 2683–2692, (2015).

(28) Fazelinia, H., Xu, M., Cheng, H. & Roder, H. Ultrafast Hydrogen Exchange Reveals Specific Structural Events during the Initial Stages of Folding of Cytochrome c. *Journal of the American Chemical Society* **136**, 733–740, (2013).

(29) Skinner, J. J., Lim, W. K., Bédard, S., Black, B. E. & Englander, S. W. Protein dynamics viewed by hydrogen exchange: Protein Dynamics from Hydrogen Exchange. *Protein Science* **21**, 996–1005, (2012).

(30) Ma, B. & Nussinov, R. Polymorphic triple beta-sheet structures contribute to amide hydrogen/deuterium (H/D) exchange protection in the Alzheimer amyloid beta42 peptide. *The Journal of Biological Chemistry* **286**, 34244–53, (2011).

(31) Hernández, G., Anderson, J. S. & LeMaster, D. M. Assessing the native state conformational distribution of ubiquitin by peptide acidity. *Biophysical Chemistry* **153**, 70–82, (2010).

(32) Hernández, G., Anderson, J. S. & LeMaster, D. M. Experimentally assessing molecular dynamics sampling of the protein native state conformational distribution. *Biophysical Chemistry* **163-164**, 21–34, (2012).

(33) Xu, J., Lee, Y., Beamer, L. J. & Doren, S. R. V. Phosphorylation in the catalytic cleft stabilizes and attracts domains of a phosphohexomutase. *Biophysical Journal* **108**, 325–37, (2015).

(34) Devaurs, D. *et al.* Coarse-Grained Conformational Sampling of Protein Structure Improves the Fit to Experimental Hydrogen-Exchange Data. *Frontiers in Molecular Biosciences* **4**, 13, (2017).

(35) Wan, H., Ge, Y., Razavi, A. & Voelz, V. A. Reconciling Simulated Ensembles of Apomyoglobin with Experimental Hydrogen/Deuterium Exchange Data Using Bayesian Inference and Multiensemble Markov State Models. *Journal of Chemical Theory and Computation* **16**, 1333–1348, (2020).

(36) Radou, G., Dreyer, F., Tuma, R. & Paci, E. Functional Dynamics of Hexameric Helicase Probed by Hydrogen Exchange and Simulation. *Biophysical Journal* **107**, 983–990, (2014).

(37) Adhikary, S., Deredge, D. J., Nagarajan, A., Forrest, L. R., Wintrode, P. L. & Singh, S. K Conformational dynamics of a neurotransmitter:sodium symporter in a lipid bilayer. *Proceedings of the National Academy of Sciences* **114**, E1786–E1795, (2017).

(38) Borysik, A. J. Simulated Isotope Exchange Patterns Enable Protein Structure Determination. *Angewandte Chemie International Edition* **56**, 9396–9399, (2017).

(39) Devaurs, D. *et al.* Native state of complement protein C3d analysed via hydrogen exchange and conformational sampling. *International Journal of Computational Biology and Drug Design* **11**, 90, (2018).

(40) Markwick, P. R. L., Peacock, R. B. & Komives, E. A. Accurate Prediction of Amide Exchange in the Fast Limit Reveals Thrombin Allostery. *Biophysical Journal* **116**, 49–56, (2019).

(41) Aytenfisu, A. H. *et al.* Insights into substrate recognition and specificity for IgG by Endoglycosidase S2. *PLOS Computational Biology* **17**, e1009103, (2021).

(42) Kihn, K. C. *et al.* Modeling the native ensemble of PhuS using enhanced sampling MD and HDX-ensemble reweighting. *Biophysical Journal* **120**, 5141–5157, (2021).

(43) Bai, N., Kirubakaran, P. & Karanicolas, J. Rationalizing PROTAC-mediated ternary complex formation using Rosetta. *J. Chem. Inf. Model.* **61**, 1368–1382, (2021).

(44) Pracht, P., Bohle, F. & Grimme, S. Automated exploration of the low-energy chemical space with fast quantum chemical methods. *Phys. Chem. Chem. Phys.* **22**, 7169–7192, (2020).

(45) Méndez, R., Leplae, R., De Maria, L. & Wodak, S. J. Assessment of blind predictions of protein–protein interactions: Current status of docking methods. *Proteins* **52**, 51–67, (2003).

(46) Bussi, G. Hamiltonian replica exchange in gromacs: a flexible implementation. *Molecular Physics* **112**, 379–384, (2014).

(47) Wang, L., Friesner, R. A. & Berne, B. J. Replica exchange with solute scaling: a more efficient version of replica exchange with solute tempering (rest2). *The Journal of Physical Chemistry B* **115**, 9431–9438, (2011).

(48) Shrestha, U. R. *et al.* Generation of the configurational ensemble of an intrinsically disordered protein from unbiased molecular dynamics simulation. *Proceedings of the National Academy of Sciences* **116**, 20446–20452, (2019).

(49) Shrestha, U. R., Smith, J. C. & Petridis, L. Full structural ensembles of intrinsically disordered proteins from unbiased molecular dynamics simulations. *Communications Biology* **4**, 243, (2021).

(50) Cong, X. & Golebiowski, J. Allosteric na+-binding site modulates cxcr4 activation. *Phys. Chem. Chem. Phys.* **20**, 24915–24920, (2018).

(51) Cong, X., Chéron, J.-B., Golebiowski, J., Antonczak, S. & Fiorucci, S. Allosteric modulation mechanism of the mglur5 transmembrane domain. *Journal of Chemical Information and Modeling* **59**, 2871–2878, (2019).

(52) Abraham, M. J., Murtola, T., Schulz, R., Szilard, P., Smith, J. C., Hess, E., & Lindahl, E. GROMACS: High performance molecular simulations through multi-level parallelism from laptops to supercomputers. *SoftwareX* **1-2**, 19–25, (2015).

(53) Szilard, P., Abraham, M. J., Kutzner, C., Hess, B., & Lindahl, E. Tackling Exascale Software Challenges in Molecular Dynamics Simulations with GROMACS. *Solving Software Challenges for Exascale* **8759**, 3–27, (2015).

(54) Hess, B., Kutzner, C., van der Spoel, D., & Lindahl, E. GROMACS 4: Algorithms for Highly Efficient, Load-Balanced, and Scalable Molecular Simulation. *Journal of Chemical Theory and Computation* **4**, 435–447, (2008).

(55) Bonomi, M. *et al.* Plumed: A portable plugin for free-energy calculations with molecular dynamics. *Computer Physics Communications* **180**, 1961–1972, (2009).

(56) Bonomi, M. *et al.* Promoting transparency and reproducibility in enhanced molecular simulations. *Nature methods* **16**, 670–673, (2019).

(57) Tribello, G. A., Bonomi, M., Branduardi, D., Camilloni, C. & Bussi, G. Plumed 2: New feathers for an old bird. *Computer Physics Communications* **185**, 604–613, (2014).

(58) Sugita, Y. & Okamoto, Y. Replica-exchange molecular dynamics method for protein folding. *Chemical Physics Letters* **314**, 141–151, (1999).

(59) Okabe, T., Kawata, M., Okamoto, Y. & Mikami, M. Replica-exchange monte carlo

method for the isobaric–isothermal ensemble. *Chemical Physics Letters* **335**, 435–439, (2001).

(60) Farnaby, W. *et al.* Baf complex vulnerabilities in cancer demonstrated via structure-based protac design. *Nature chemical biology* **15**, 672–680 (2019).

(61) Rambo, R. P., & Tainer, J. A. Super-Resolution in Solution X-Ray Scattering and Its Applications to Structural Systems Biology. *Annual Review of Biophysics* **42**, 415–441, (2013).

(62) Piiadov, V., Ares de Araújo, E., Neto, M. O., Craievich, A. F., & Polikarpov, I. SAXS-MoW 2.0: Online calculator of the molecular weight of proteins in dilute solution from experimental SAXS data measured on a relative scale. *Protein Science* **28**, 454–463, (2019).

(63) Hajizadeh, N. R., Franke, D., Jeffries, C. M., & Svergun, D. I. Consensus Bayesian assessment of protein molecular mass from solution X-ray scattering data. *Scientific Reports* **8**, 7204, (2018).

(64) Franke, D., Jeffries, C. M., & Svergun, D. I. Machine Learning Methods for X-Ray Scattering Data Analysis from Biomacromolecular Solutions. *Biophysical Journal* **114**, 2485–2492, (2018).
