# Supplementary material for: Predicting the structural basis of targeted protein degradation by integrating molecular dynamics simulations with structural mass spectrometry
Source: Nat Commun. 2022 Oct 6;13:5884. doi: 10.1038/s41467-022-33575-4 (PMC9537307; doi:10.1038/s41467-022-33575-4)
Supplement: Supplementary file 2 — Description of Additional Supplementary Files [file 41467_2022_33575_MOESM2_ESM.docx]

File Name: Supplementary Data 1

Description: All raw relative uptake plots of the deuterium exchange for each state and experiment. 
 
 

File Name: Supplementary Data 2

Description: Peptides and sites identified from ubiquitin-enriched sample from Hela cells
