# Supplementary material for: Predicting the structural basis of targeted protein degradation by integrating molecular dynamics simulations with structural mass spectrometry
Source: Nat Commun. 2022 Oct 6;13:5884. doi: 10.1038/s41467-022-33575-4 (PMC9537307; doi:10.1038/s41467-022-33575-4)
Supplement: Supplementary file 3 — Supplementary Dataset 1 [file 41467_2022_33575_MOESM3_ESM.pdf]

Supplementary Data 1

All raw relative uptake plots of the deuterium exchange for each state and experiment.

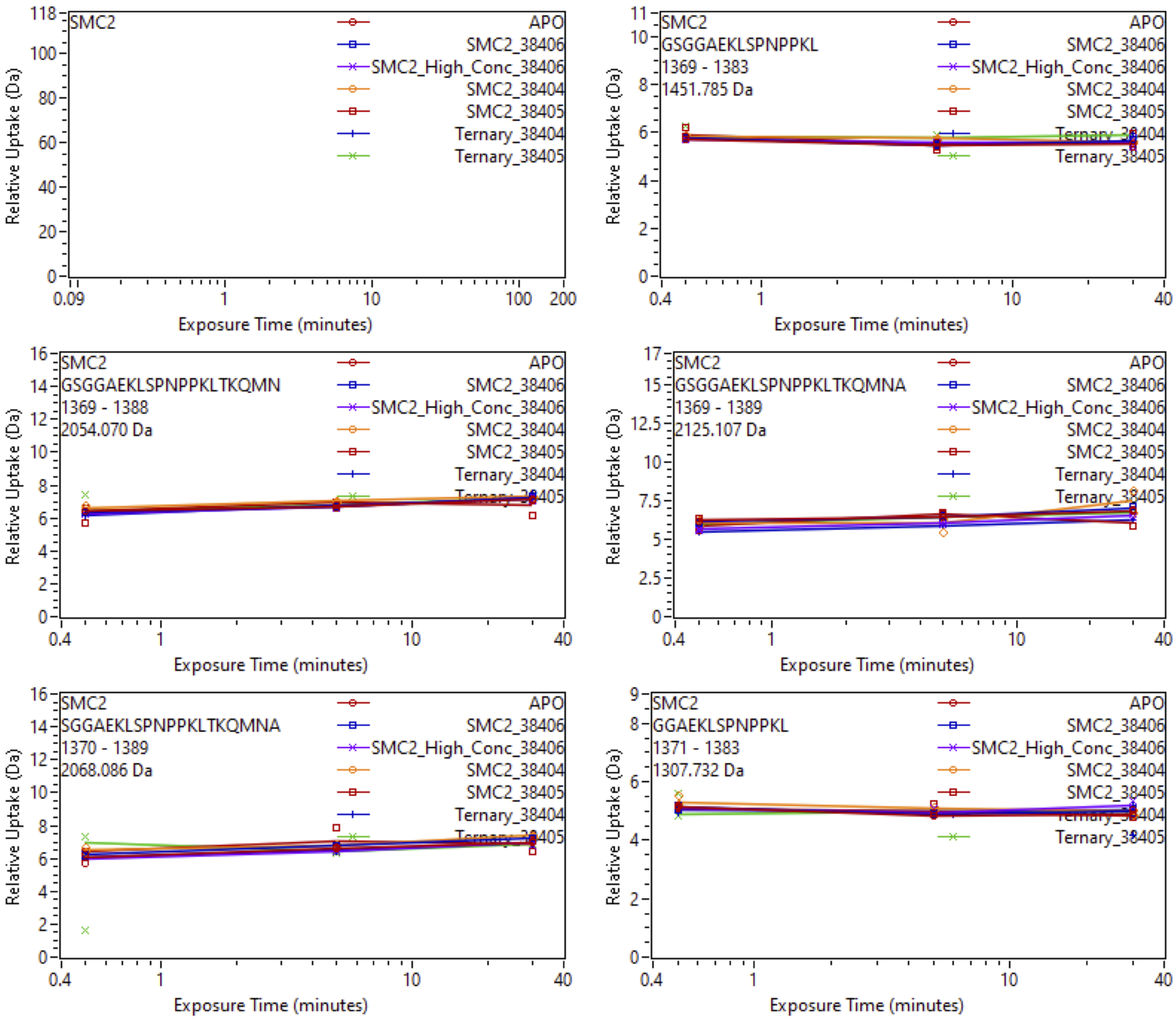

Relative deuterium uptake plots of peptic peptides of iso2-SMARCA2^{BD} in the APO, Binary with SiTX-0038404 (PROTAC 1), SiTX-0038405(PROTAC 2), SiTX-0038406(ACBI1) or Ternary complex with 404, 405, 406 + VCB.

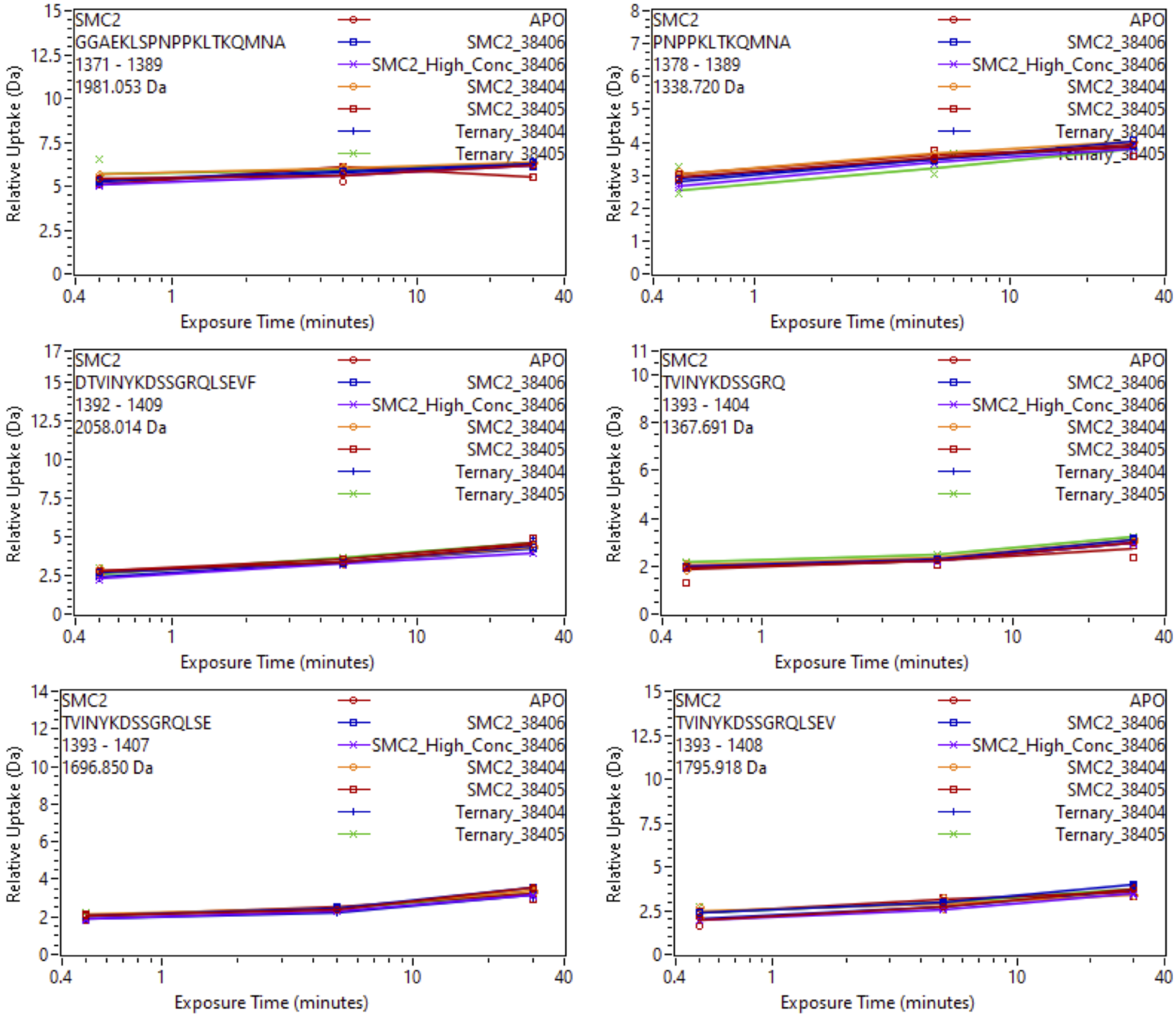

Relative deuterium uptake plots of peptic peptides of iso2-SMARCA2^{BD} in the APO, Binary with SiTX-0038404 (PROTAC 1), SiTX-0038405(PROTAC 2), SiTX-0038406(ACBI1) or Ternary complex with 404, 405, 406 + VCB.

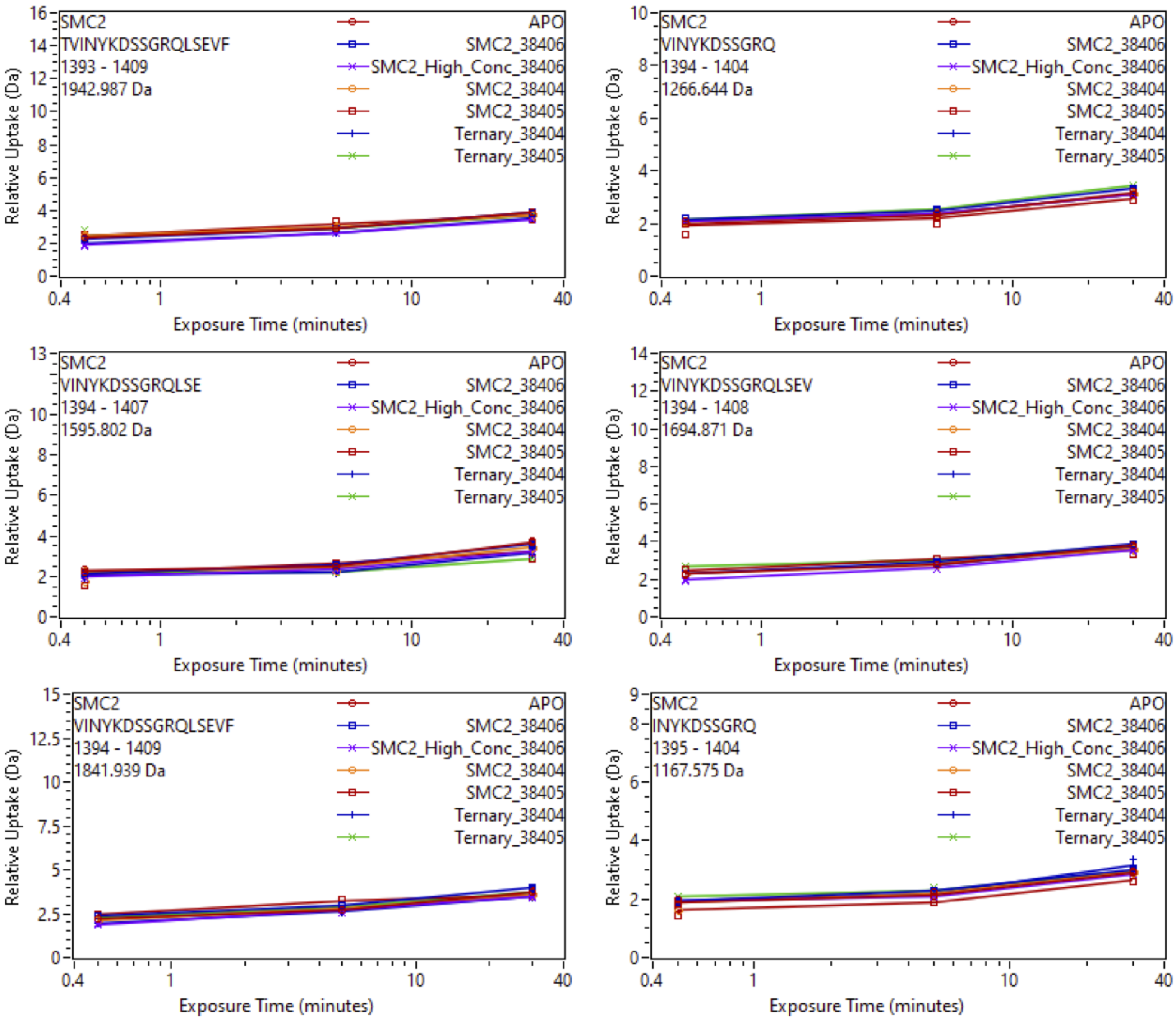

Relative deuterium uptake plots of peptic peptides of iso2-SMARCA2^{BD} in the APO, Binary with SiTX-0038404 (PROTAC 1), SiTX-0038405(PROTAC 2), SiTX-0038406(ACBI1) or Ternary complex with 404, 405, 406 + VCB.

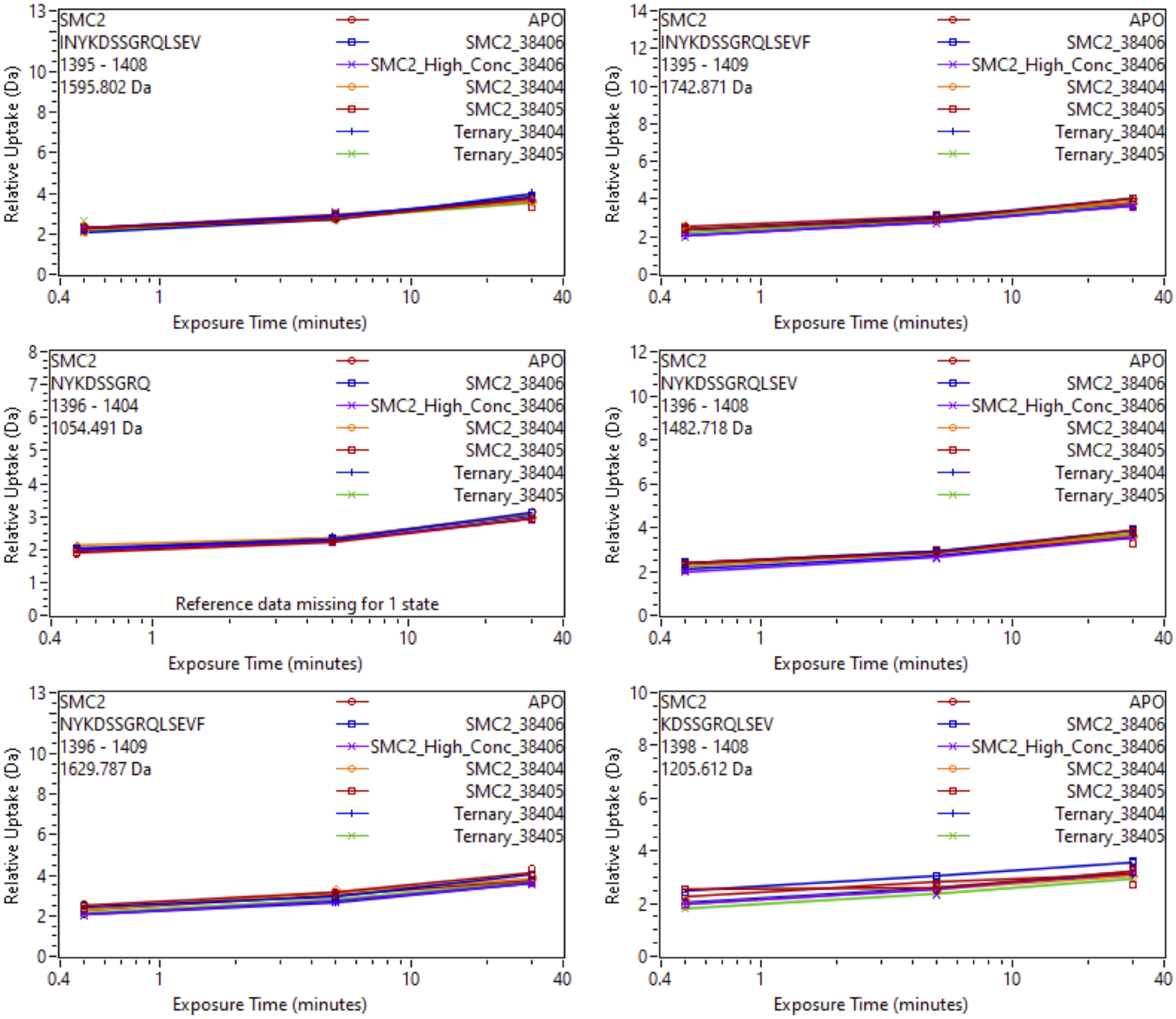

Relative deuterium uptake plots of peptic peptides of iso2-SMARCA2^{BD} in the APO, Binary with SiTX-0038404 (PROTAC 1), SiTX-0038405(PROTAC 2), SiTX-0038406(ACBI1) or Ternary complex with 404, 405, 406 + VCB.

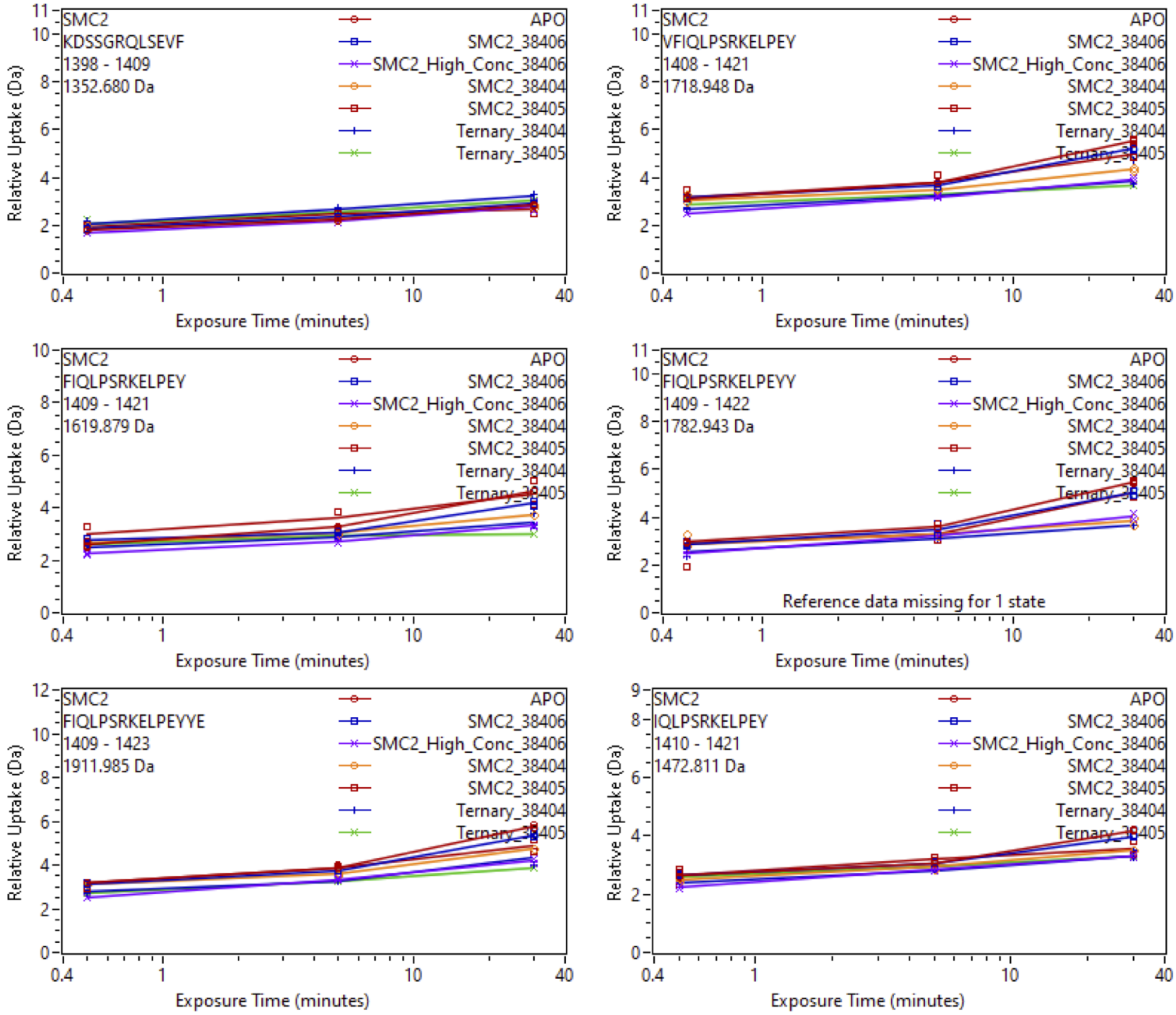

Relative deuterium uptake plots of peptic peptides of iso2-SMARCA2^{BD} in the APO, Binary with SiTX-0038404 (PROTAC 1), SiTX-0038405(PROTAC 2), SiTX-0038406(ACBI1) or Ternary complex with 404, 405, 406 + VCB.

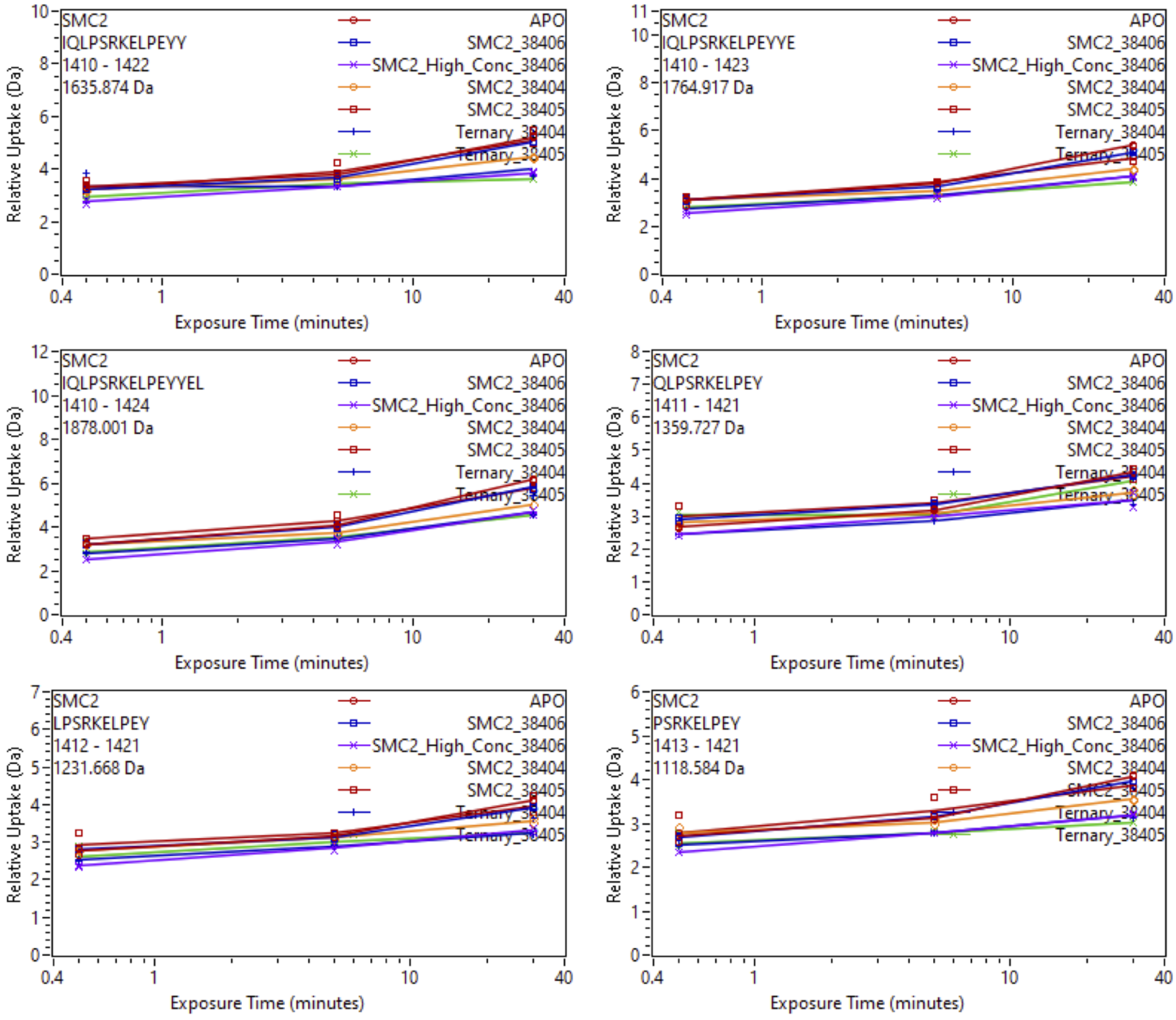

Relative deuterium uptake plots of peptic peptides of iso2-SMARCA2^{BD} in the APO, Binary with SiTX-0038404 (PROTAC 1), SiTX-0038405(PROTAC 2), SiTX-0038406(ACBI1) or Ternary complex with 404, 405, 406 + VCB.

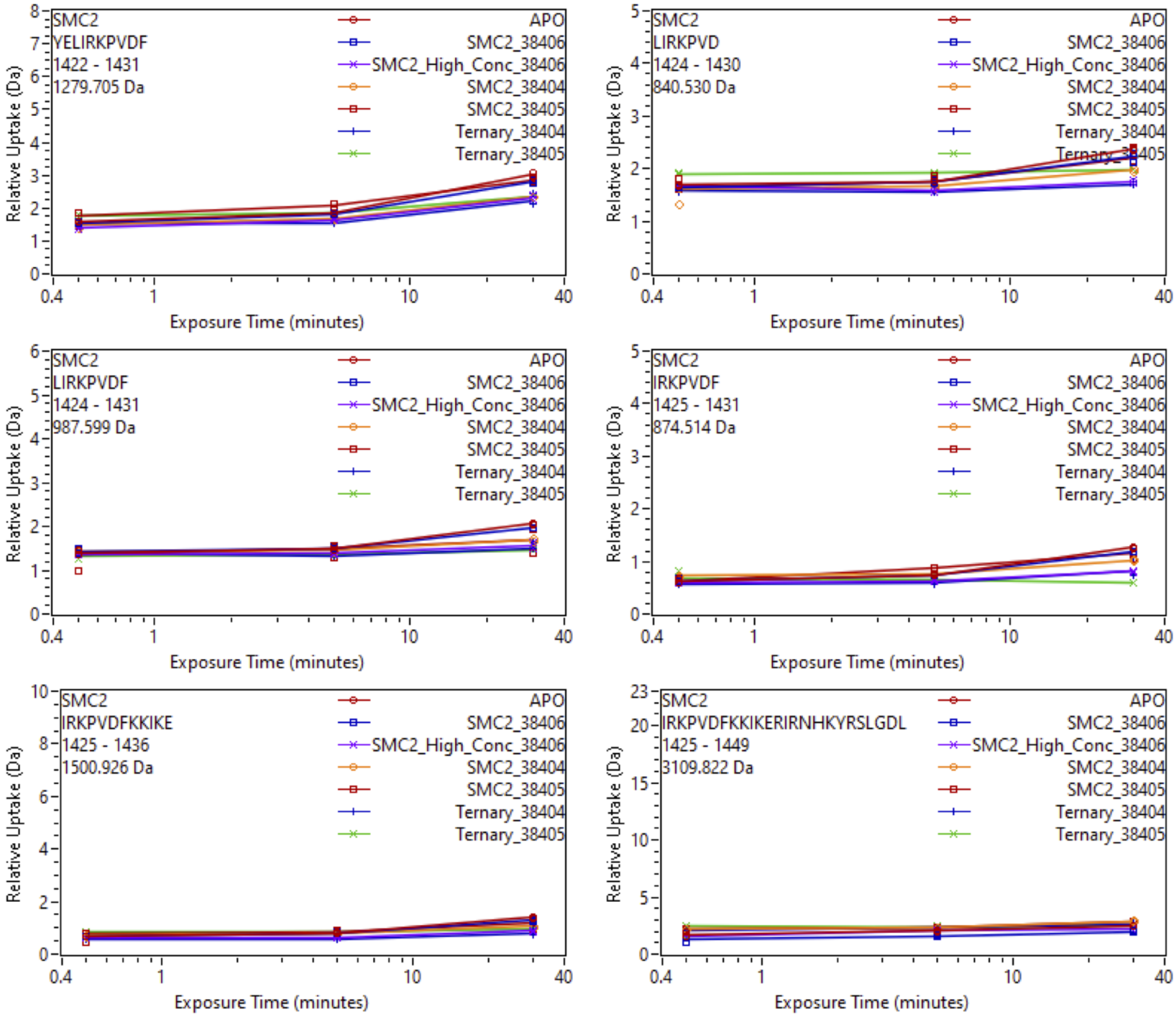

Relative deuterium uptake plots of peptic peptides of iso2-SMARCA2^{BD} in the APO, Binary with SiTX-0038404 (PROTAC 1), SiTX-0038405(PROTAC 2), SiTX-0038406(ACBI1) or Ternary complex with 404, 405, 406 + VCB.

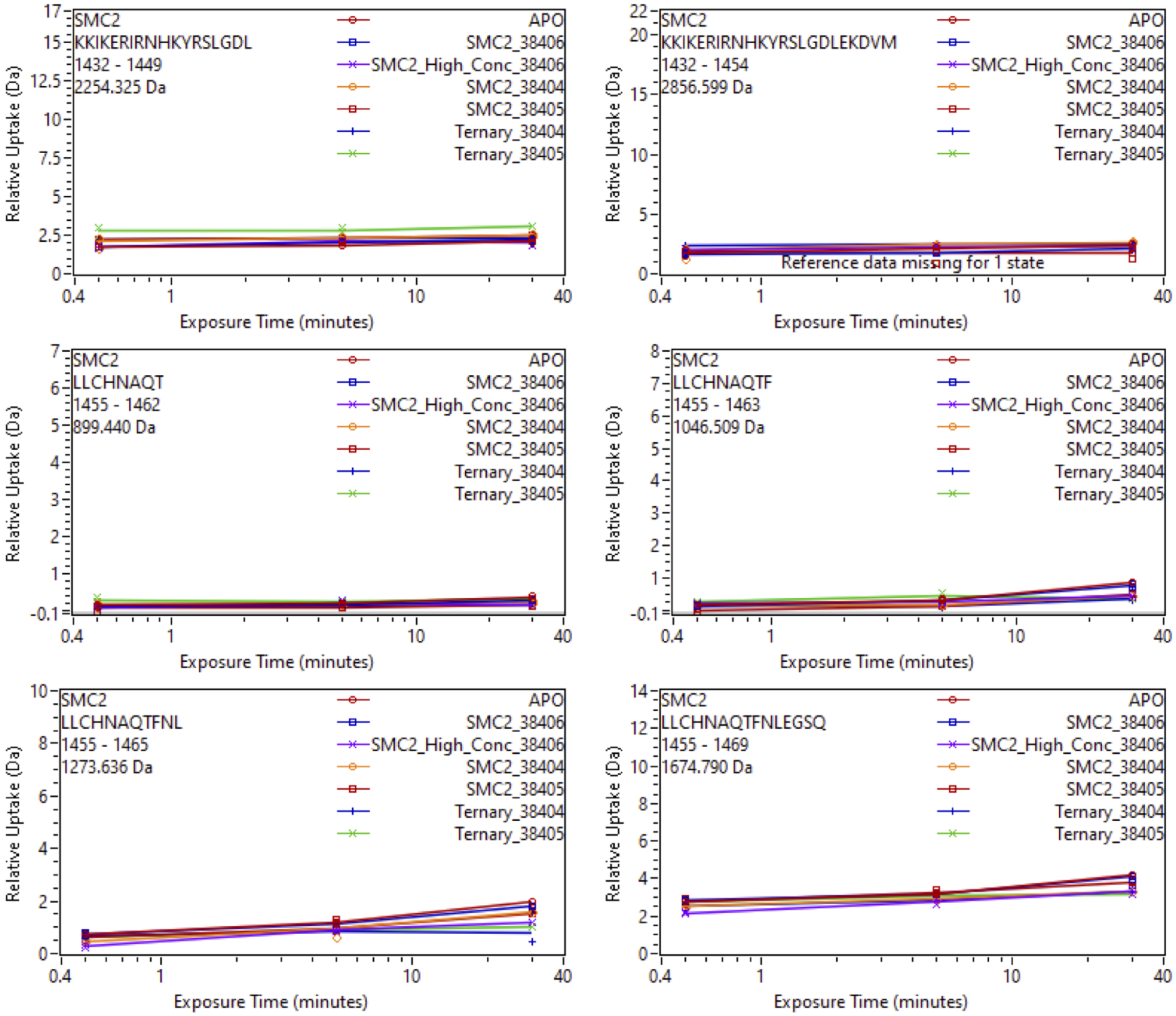

Relative deuterium uptake plots of peptic peptides of iso2-SMARCA2^{BD} in the APO, Binary with SiTX-0038404 (PROTAC 1), SiTX-0038405(PROTAC 2), SiTX-0038406(ACBI1) or Ternary complex with 404, 405, 406 + VCB.

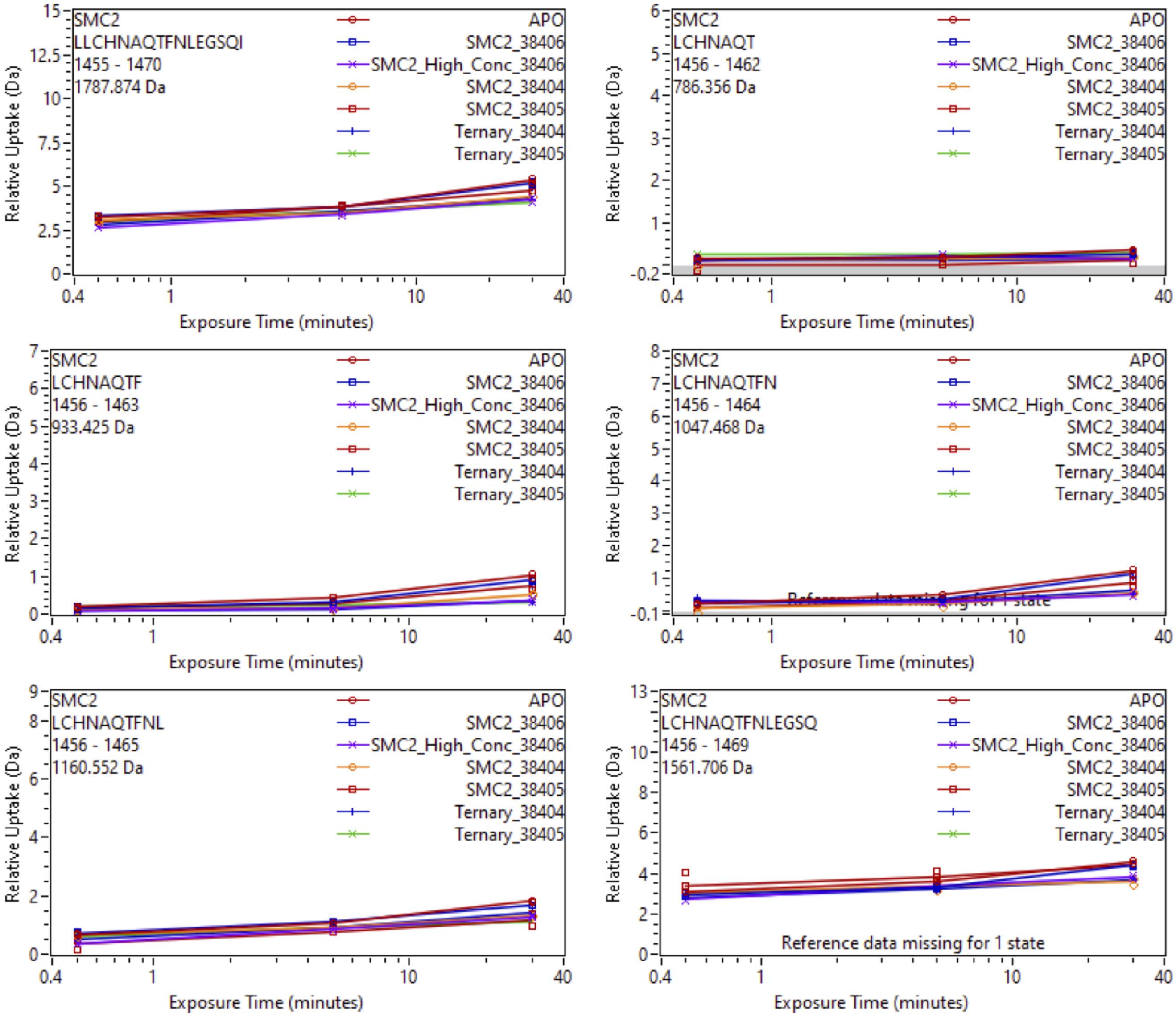

Relative deuterium uptake plots of peptic peptides of iso2-SMARCA2^{BD} in the APO, Binary with SiTX-0038404 (PROTAC 1), SiTX-0038405(PROTAC 2), SiTX-0038406(ACBI1) or Ternary complex with 404, 405, 406 + VCB.

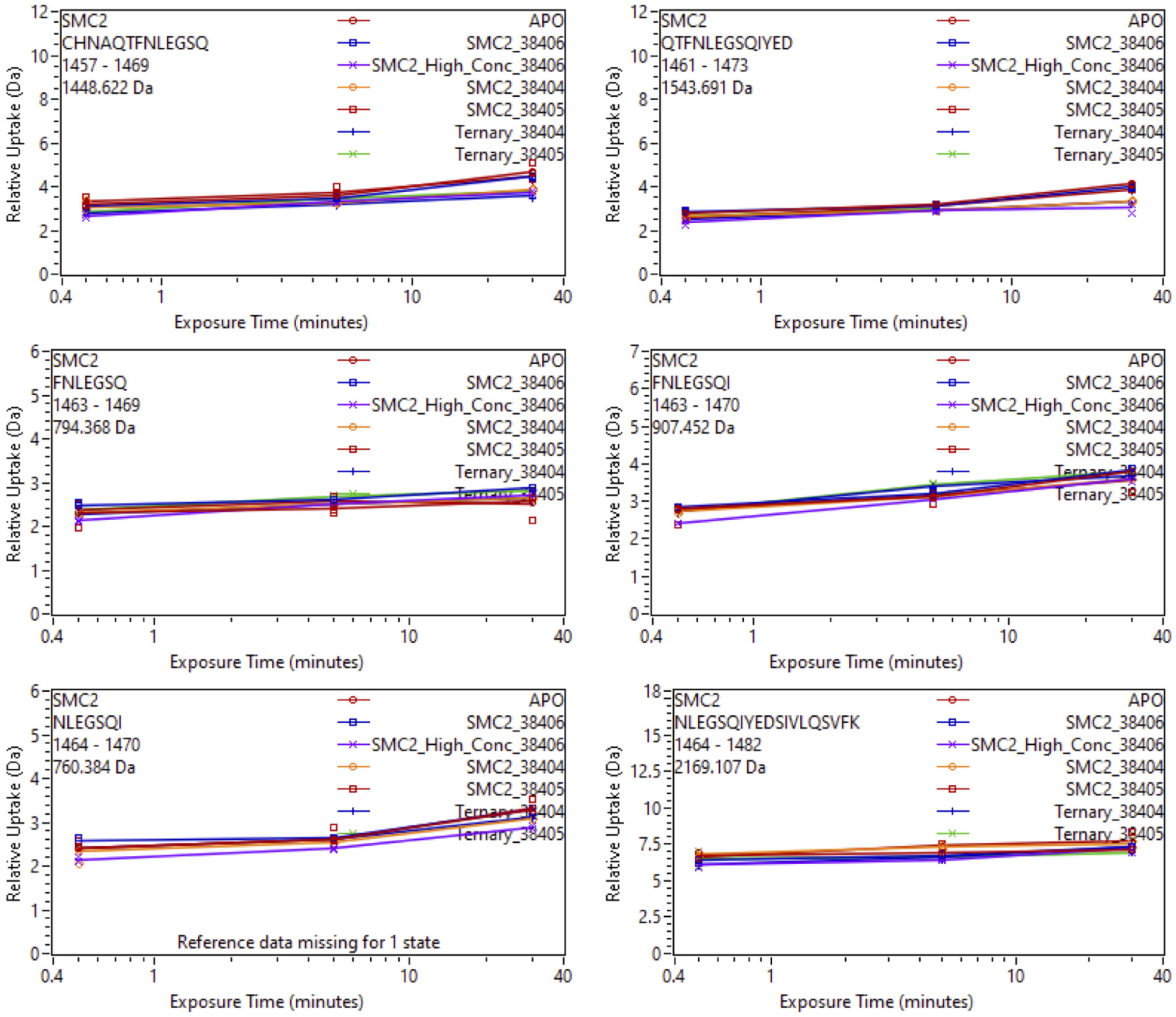

Relative deuterium uptake plots of peptic peptides of iso2-SMARCA2^{BD} in the APO, Binary with SiTX-0038404 (PROTAC 1), SiTX-0038405(PROTAC 2), SiTX-0038406(ACBI1) or Ternary complex with 404, 405, 406 + VCB.

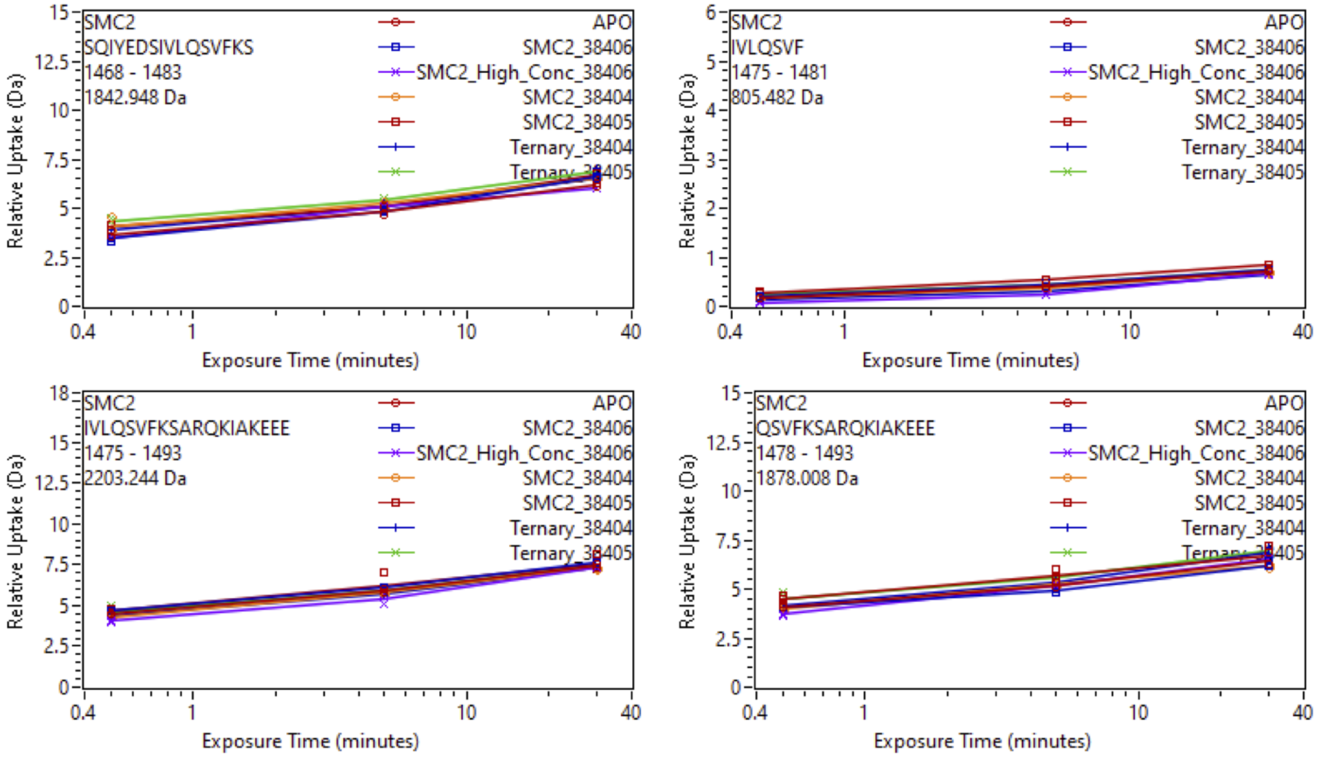

Relative deuterium uptake plots of peptic peptides of iso2-SMARCA2^{BD} in the APO, Binary with SiTX-0038404 (PROTAC 1), SiTX-0038405(PROTAC 2), SiTX-0038406(ACBI1) or Ternary complex with 404, 405, 406 + VCB.

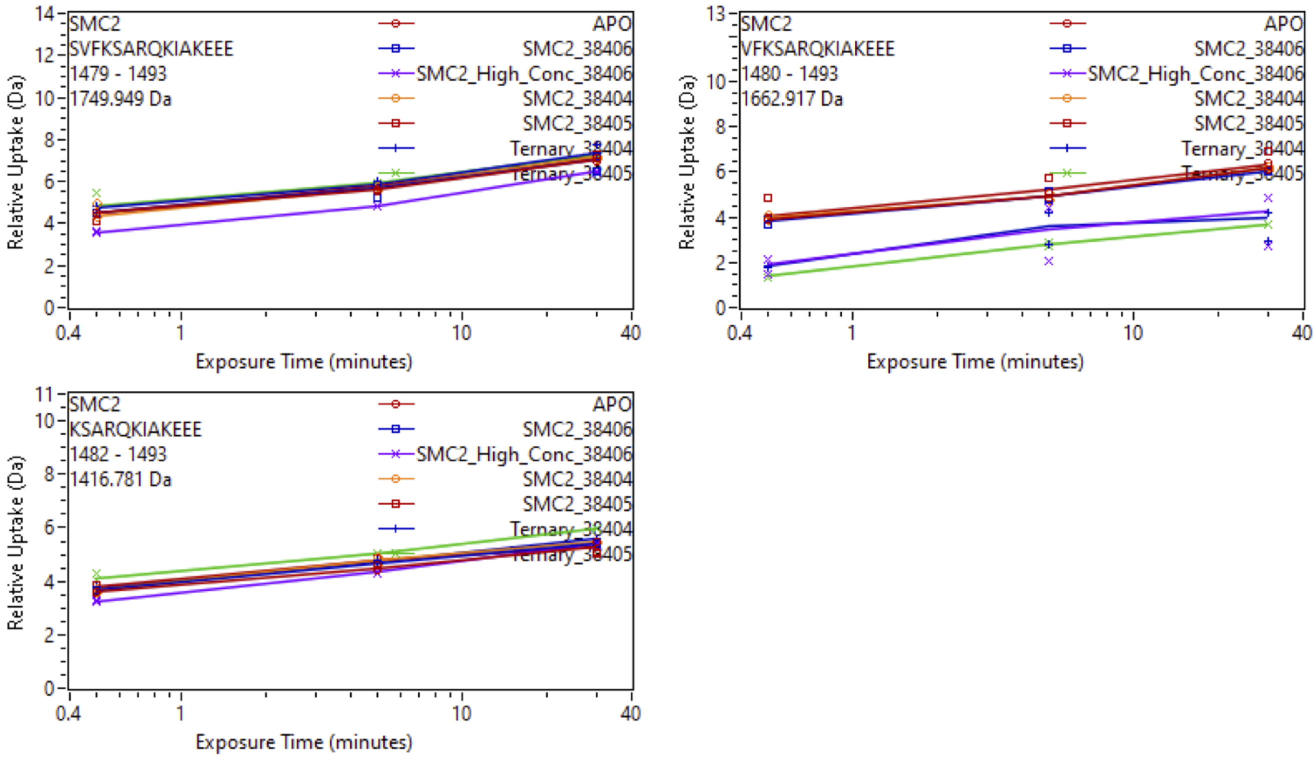

Relative deuterium uptake plots of peptic peptides of iso2-SMARCA2^{BD} in the APO, Binary with SiTX-0038404 (PROTAC 1), SiTX-0038405(PROTAC 2), SiTX-0038406(ACBI1) or Ternary complex with 404, 405, 406 + VCB.

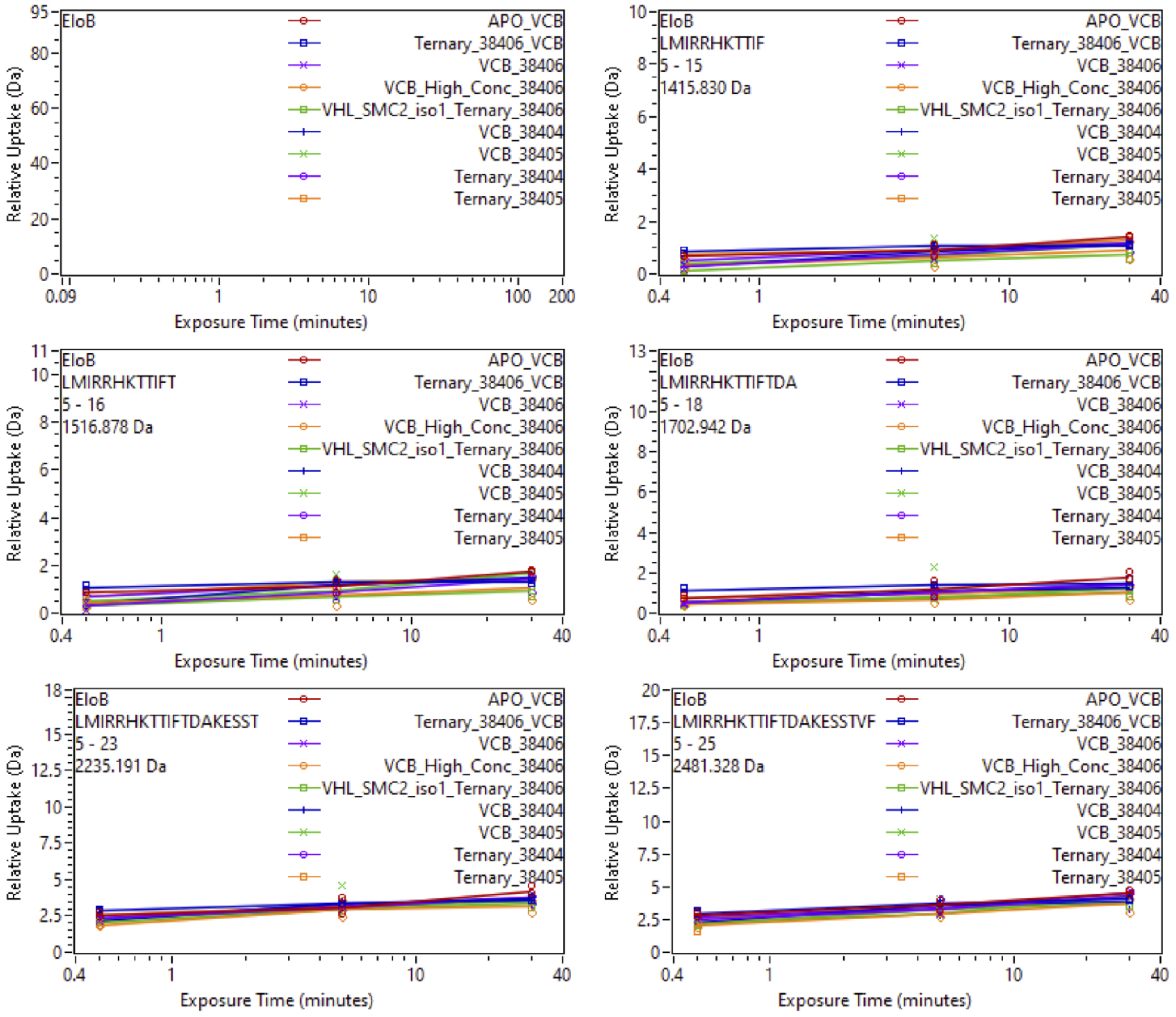

Relative deuterium uptake plots of peptic peptides Elongin B of the VCB complex in the APO, Binary with SiTX-0038404 (PROTAC 1), SiTX-0038405(PROTAC 2), SiTX-0038406(ACBI1) or Ternary complex with 404, 405, 406 + SMARCA2^{BD}.

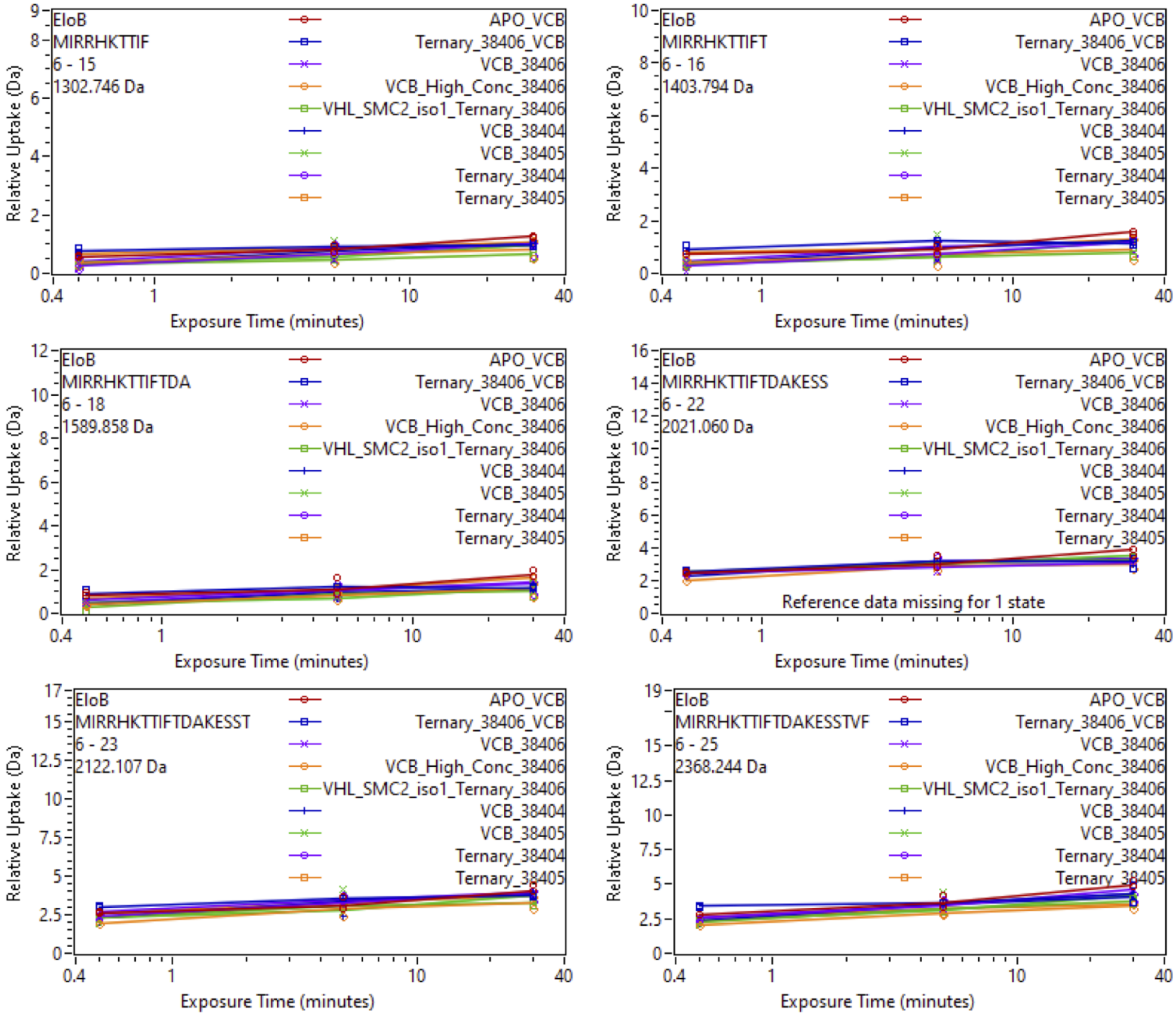

Relative deuterium uptake plots of peptic peptides Elongin B of the VCB complex in the APO, Binary with SiTX-0038404 (PROTAC 1), SiTX-0038405(PROTAC 2), SiTX-0038406(ACBI1) or Ternary complex with 404, 405, 406 + SMARCA2^{BD}.

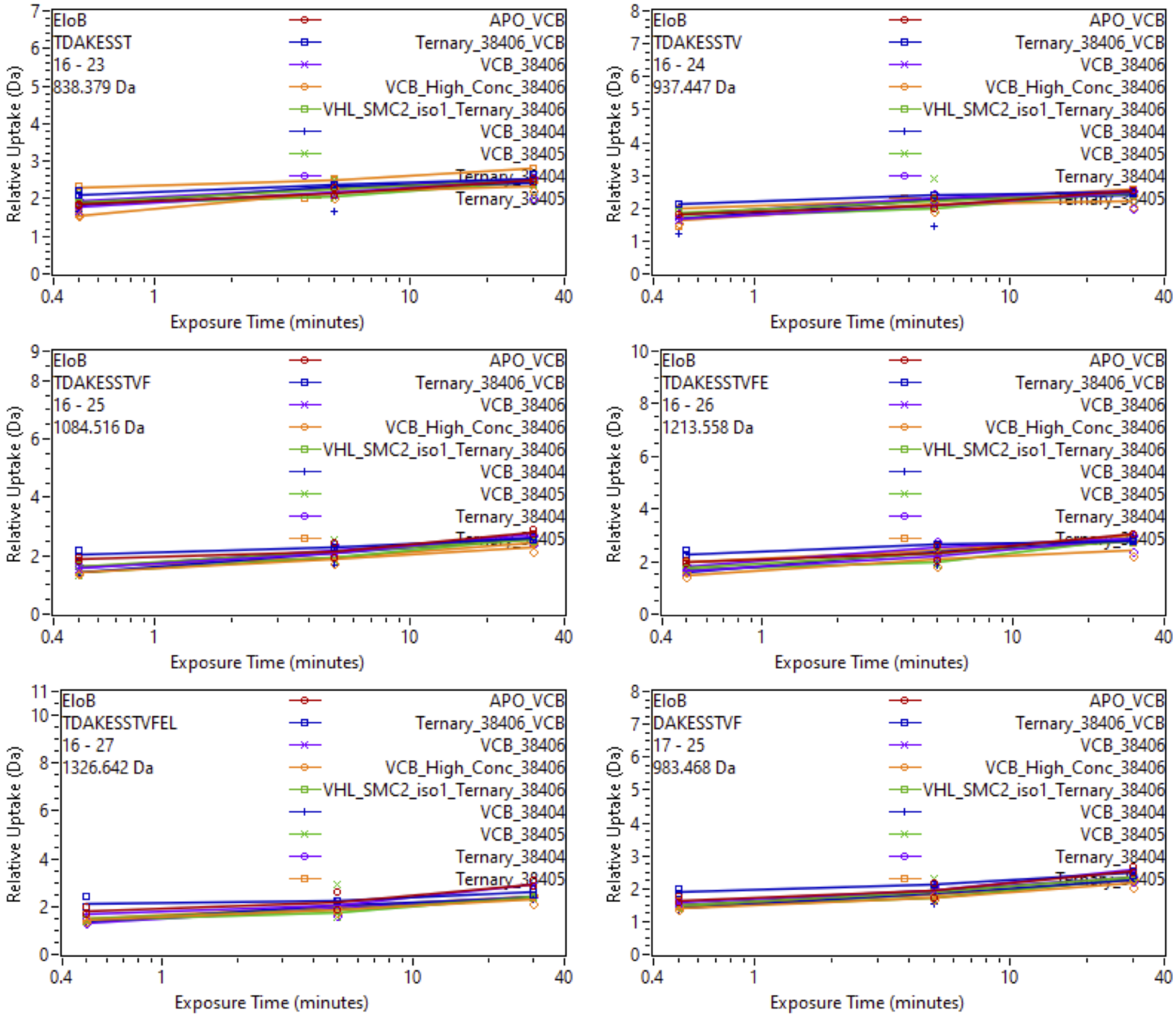

Relative deuterium uptake plots of peptic peptides Elongin B of the VCB complex in the APO, Binary with SiTX-0038404 (PROTAC 1), SiTX-0038405(PROTAC 2), SiTX-0038406(ACBI1) or Ternary complex with 404, 405, 406 + SMARCA2^{BD}.

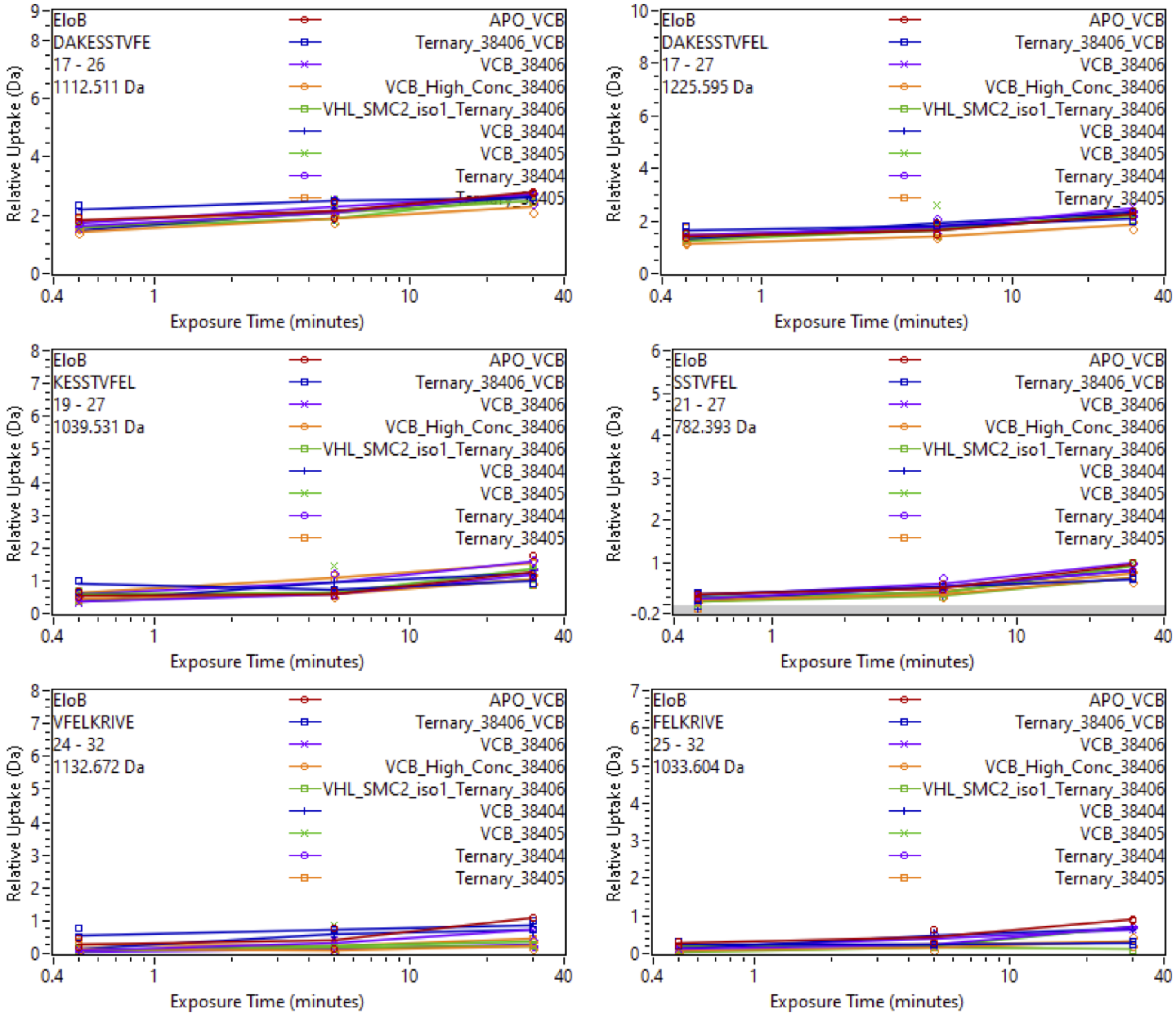

Relative deuterium uptake plots of peptic peptides Elongin B of the VCB complex in the APO, Binary with SiTX-0038404 (PROTAC 1), SiTX-0038405(PROTAC 2), SiTX-0038406(ACBI1) or Ternary complex with 404, 405, 406 + SMARCA2^{BD}.

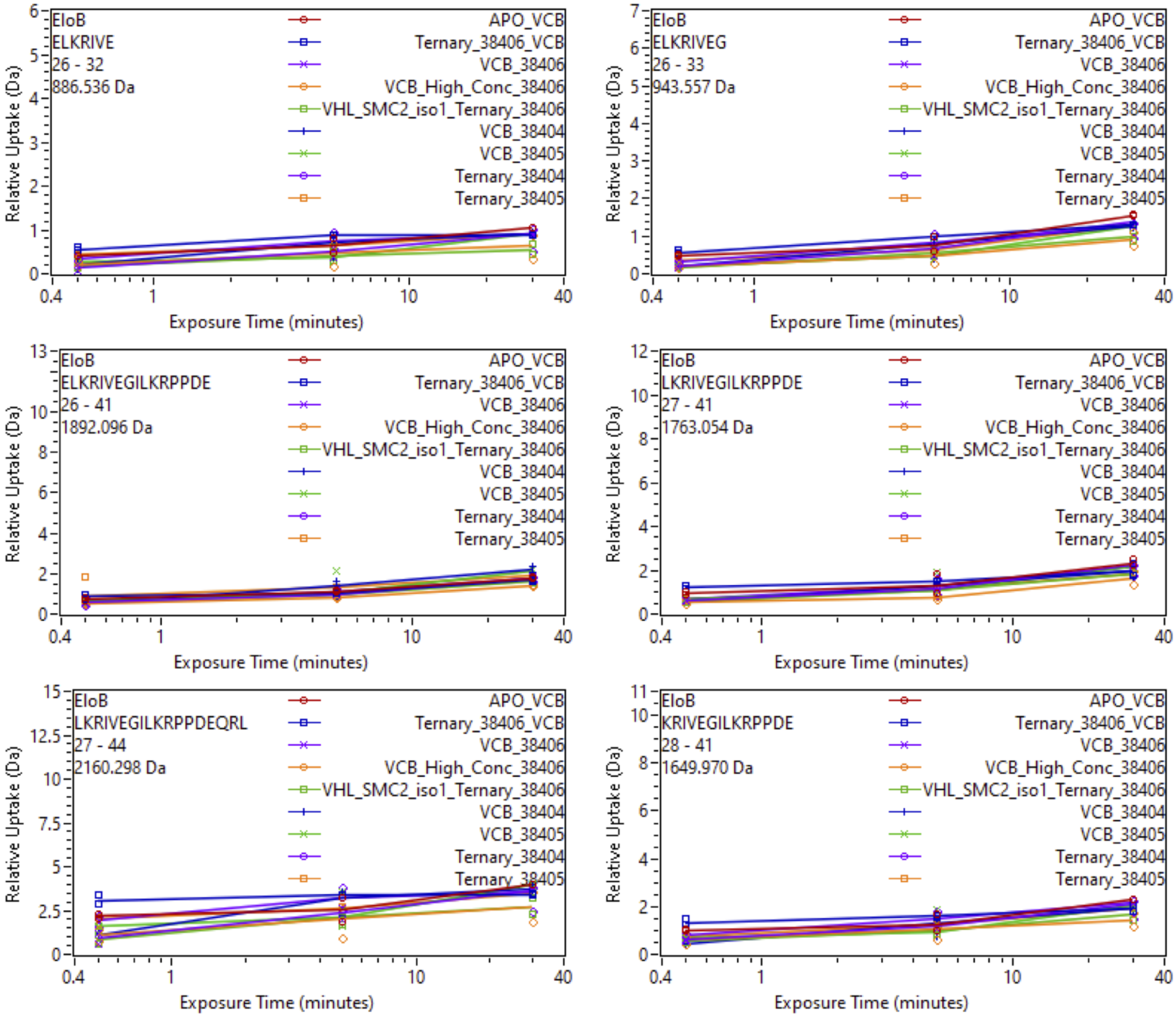

Relative deuterium uptake plots of peptic peptides Elongin B of the VCB complex in the APO, Binary with SiTX-0038404 (PROTAC 1), SiTX-0038405(PROTAC 2), SiTX-0038406(ACBI1) or Ternary complex with 404, 405, 406 + SMARCA2^{BD}.

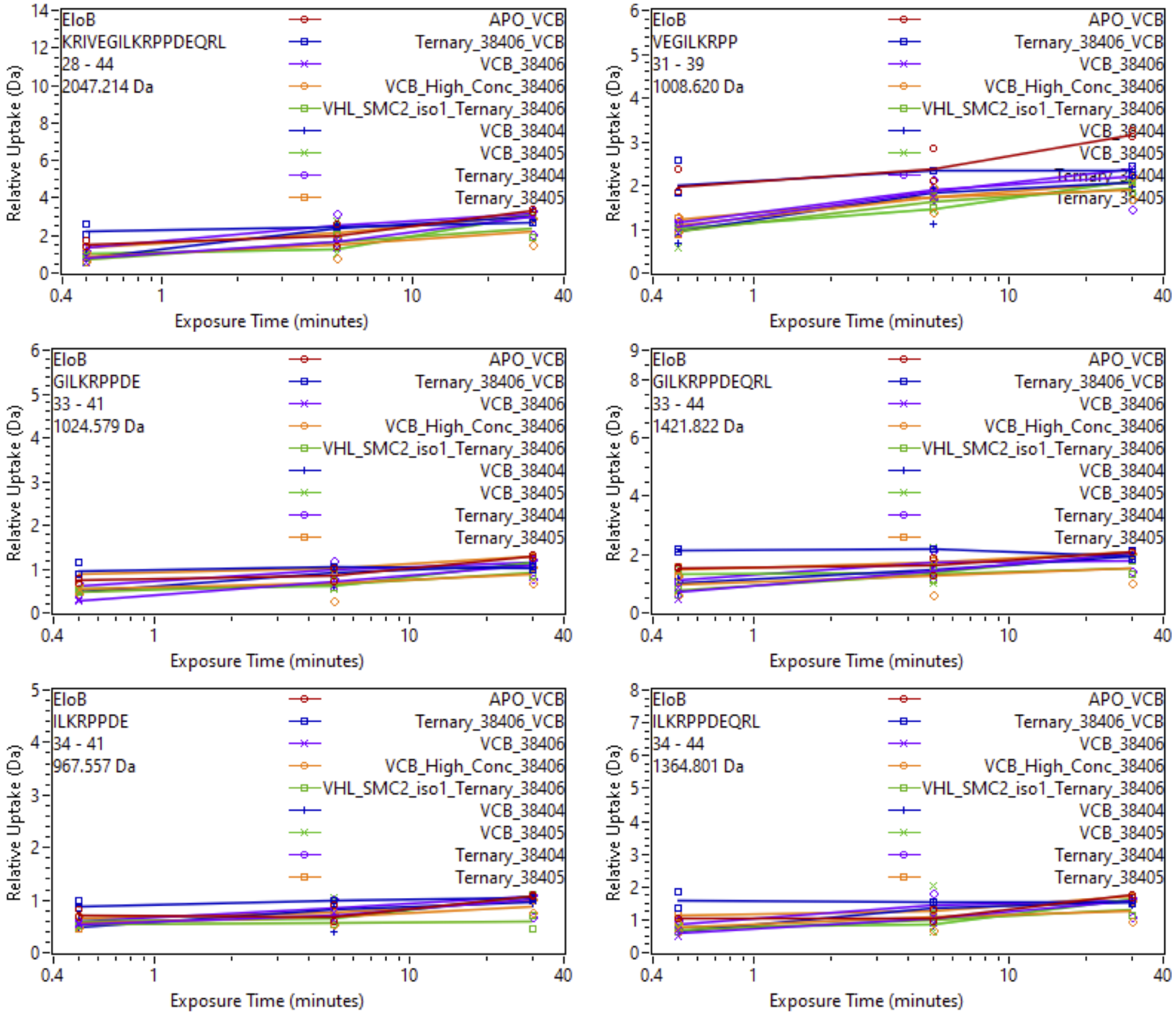

Relative deuterium uptake plots of peptic peptides Elongin B of the VCB complex in the APO, Binary with SiTX-0038404 (PROTAC 1), SiTX-0038405(PROTAC 2), SiTX-0038406(ACBI1) or Ternary complex with 404, 405, 406 + SMARCA2^{BD}.

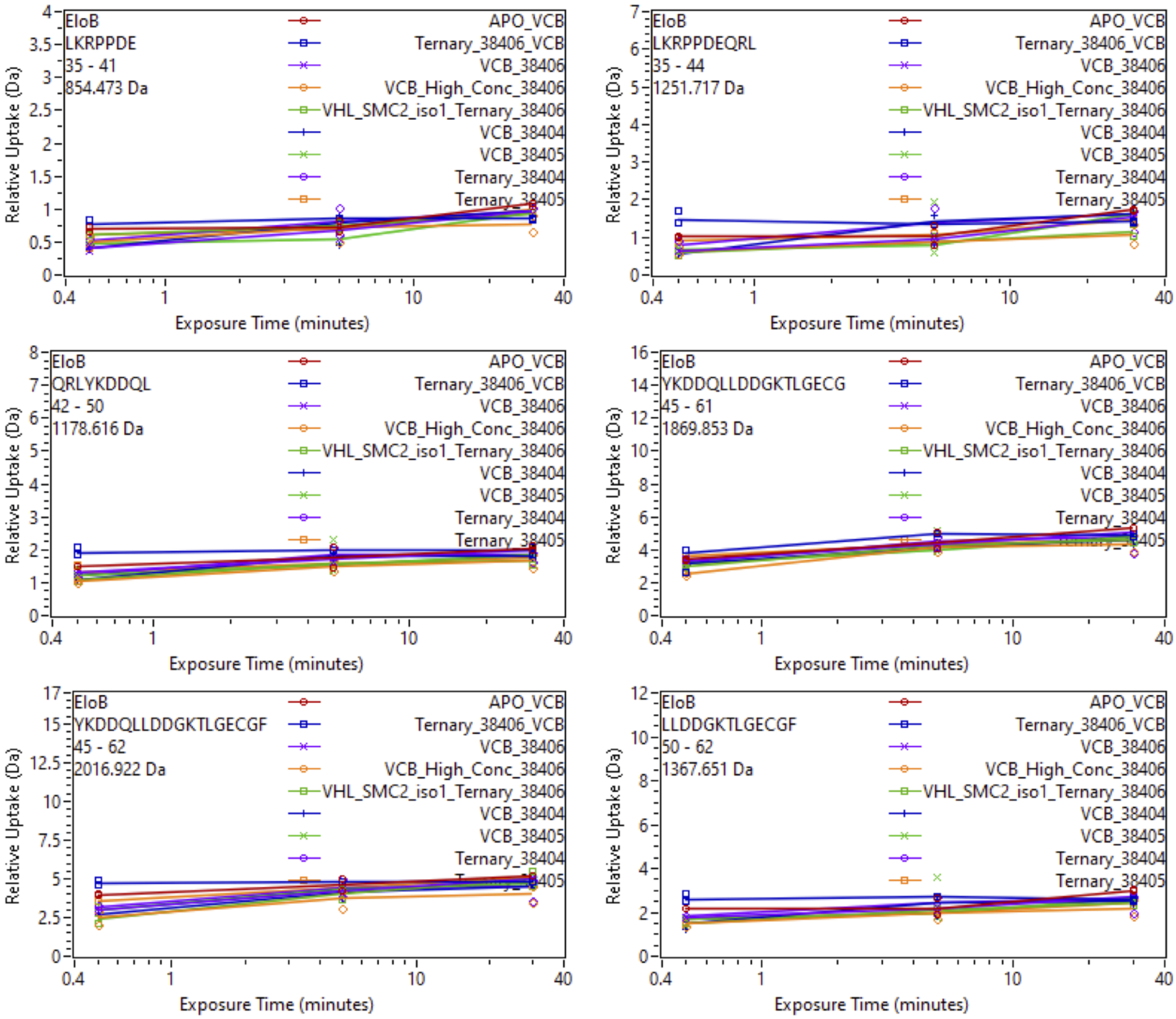

Relative deuterium uptake plots of peptic peptides Elongin B of the VCB complex in the APO, Binary with SiTX-0038404 (PROTAC 1), SiTX-0038405(PROTAC 2), SiTX-0038406(ACBI1) or Ternary complex with 404, 405, 406 + SMARCA2^{BD}.

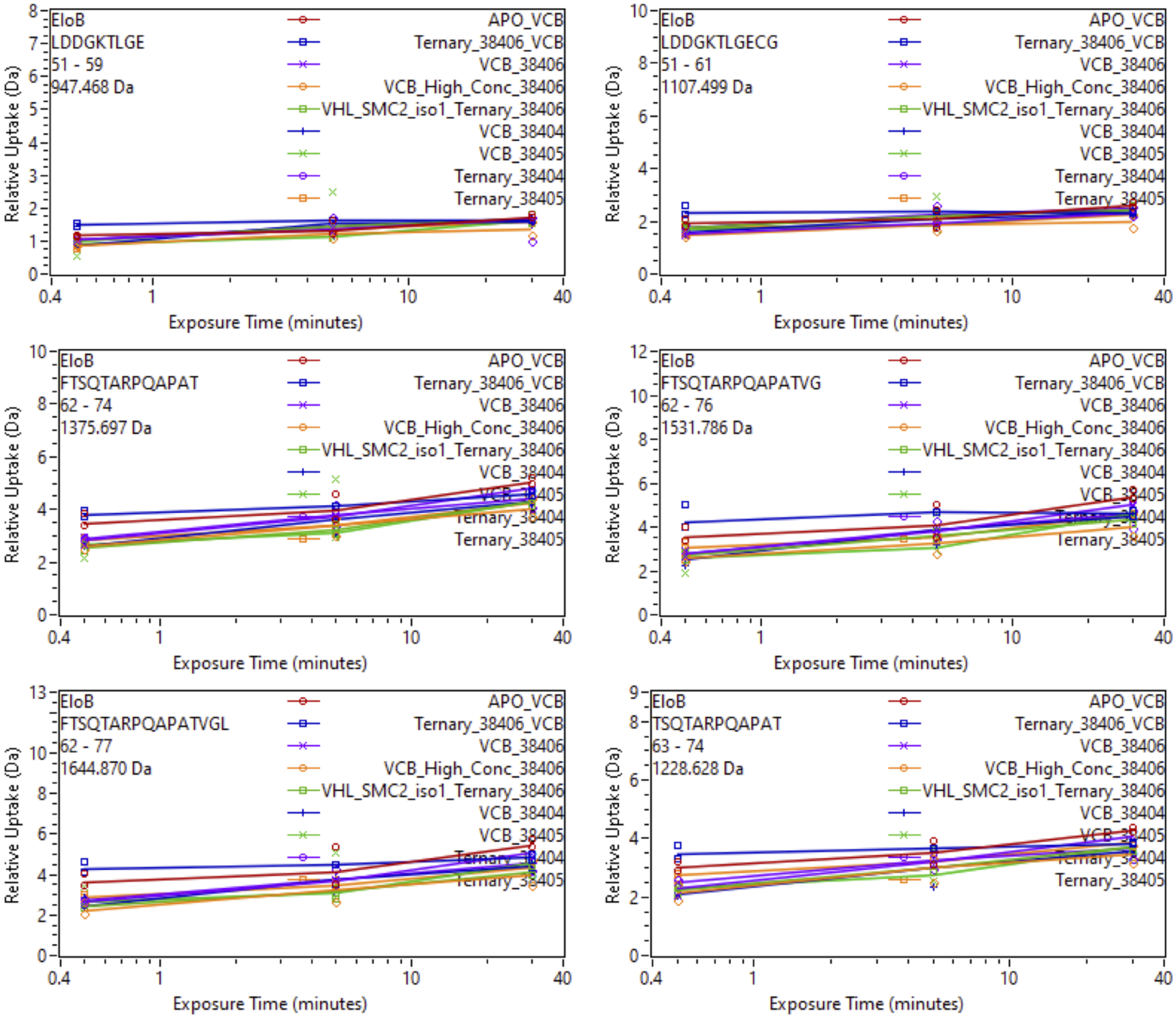

Relative deuterium uptake plots of peptic peptides Elongin B of the VCB complex in the APO, Binary with SiTX-0038404 (PROTAC 1), SiTX-0038405(PROTAC 2), SiTX-0038406(ACBI1) or Ternary complex with 404, 405, 406 + SMARCA2^{BD}.

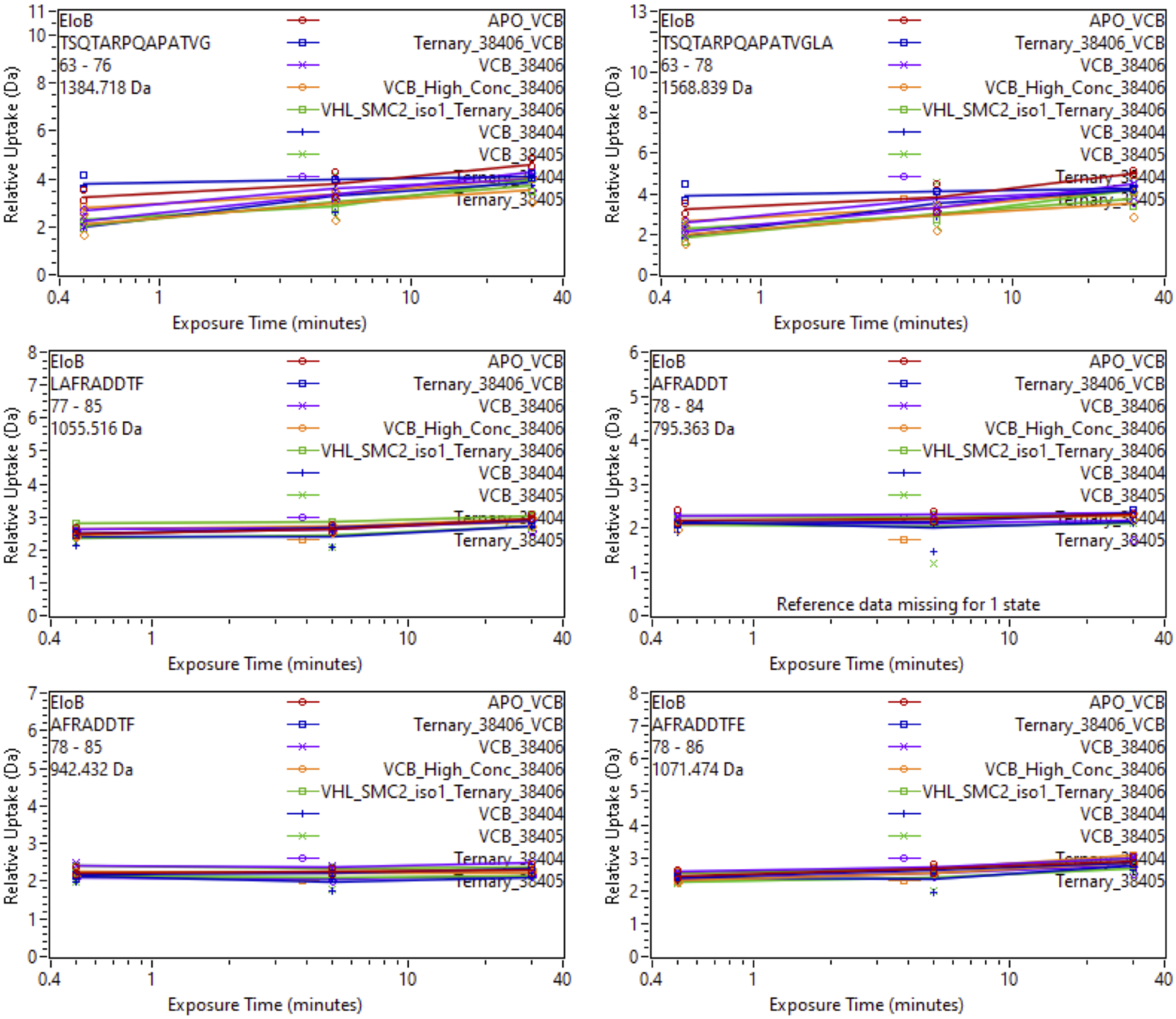

Relative deuterium uptake plots of peptic peptides Elongin B of the VCB complex in the APO, Binary with SiTX-0038404 (PROTAC 1), SiTX-0038405(PROTAC 2), SiTX-0038406(ACBI1) or Ternary complex with 404, 405, 406 + SMARCA2^{BD}.

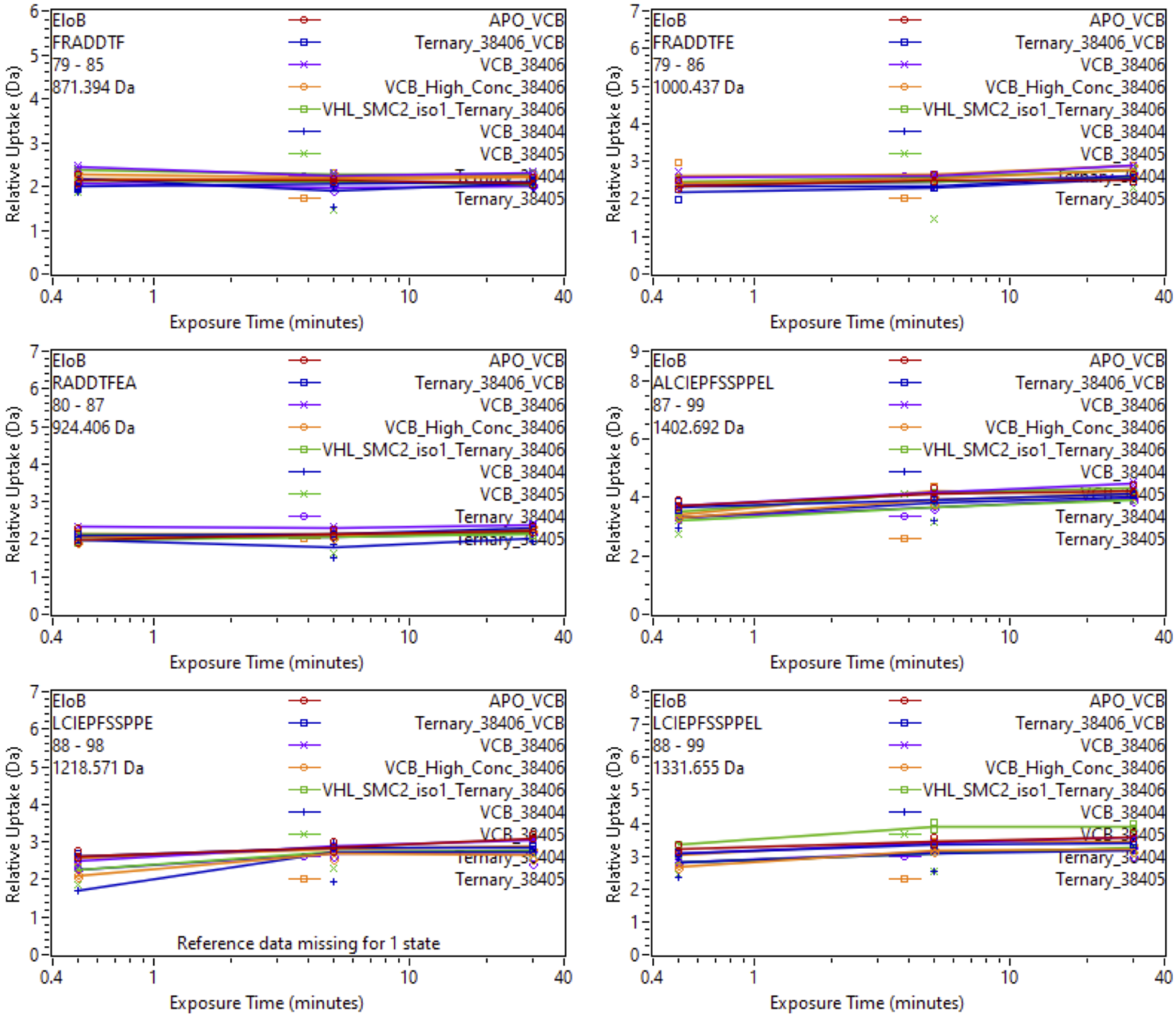

Relative deuterium uptake plots of peptic peptides Elongin B of the VCB complex in the APO, Binary with SiTX-0038404 (PROTAC 1), SiTX-0038405(PROTAC 2), SiTX-0038406(ACBI1) or Ternary complex with 404, 405, 406 + SMARCA2^{BD}.

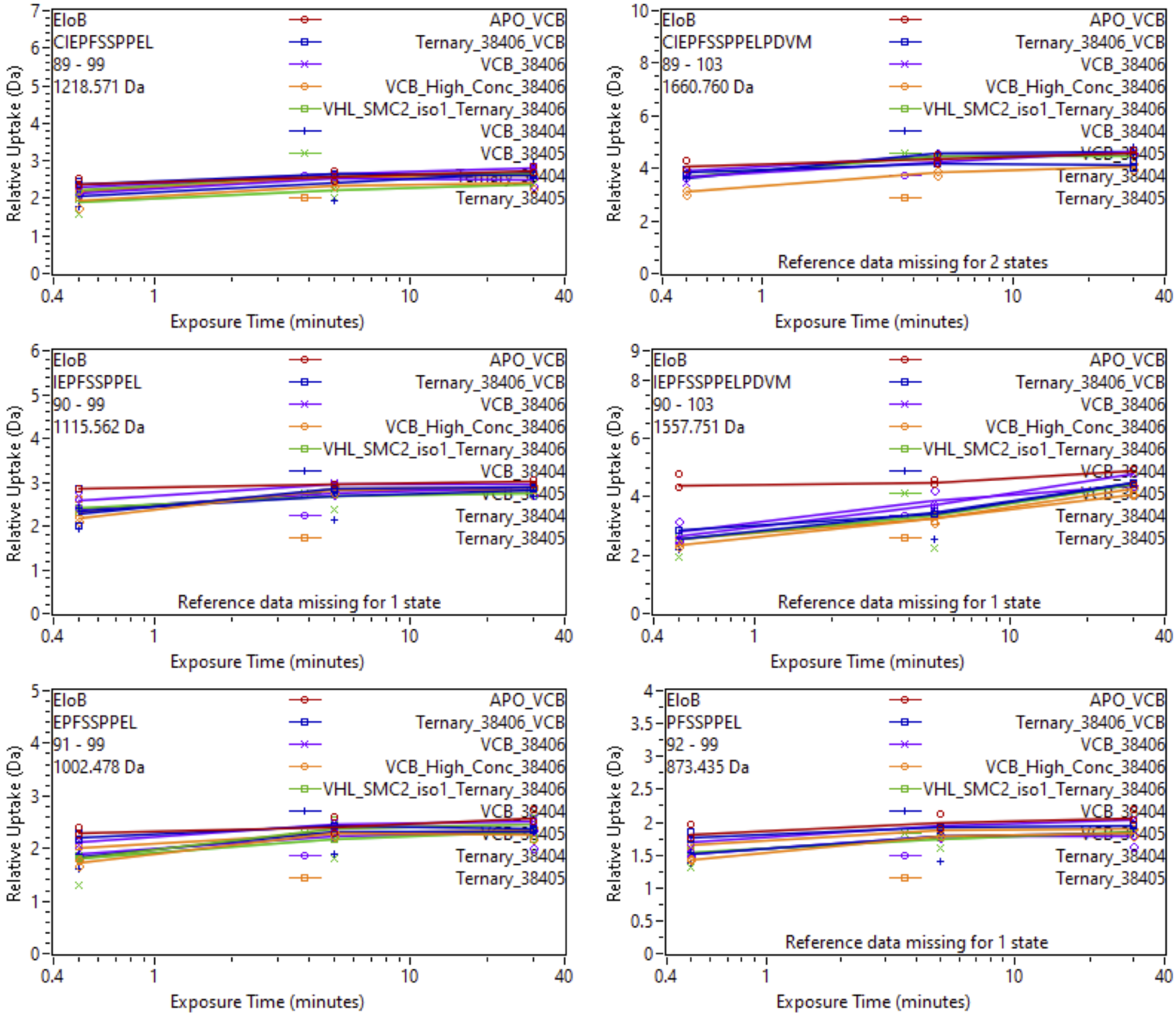

Relative deuterium uptake plots of peptic peptides Elongin B of the VCB complex in the APO, Binary with SiTX-0038404 (PROTAC 1), SiTX-0038405(PROTAC 2), SiTX-0038406(ACBI1) or Ternary complex with 404, 405, 406 + SMARCA2^{BD}.

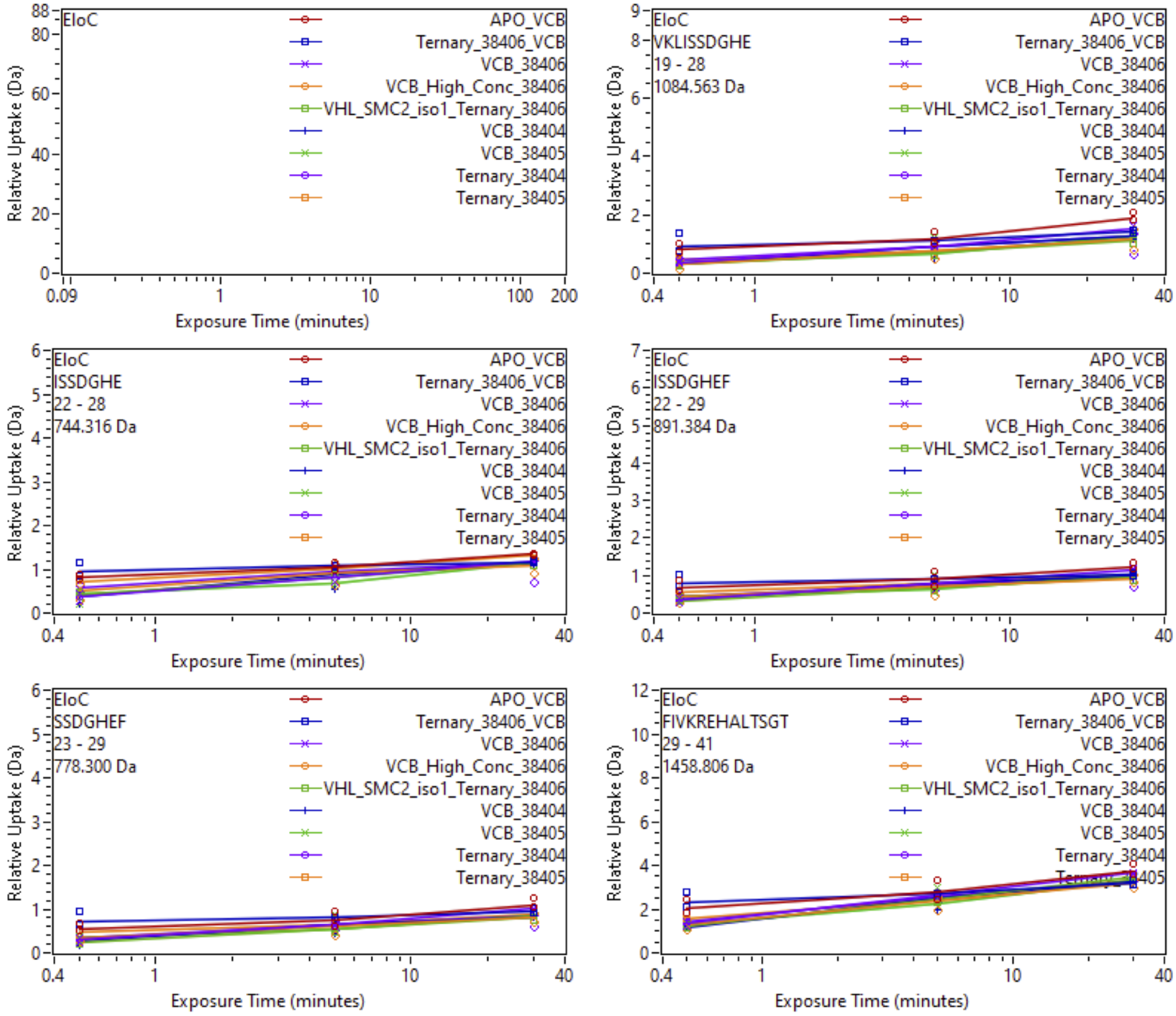

Relative deuterium uptake plots of peptic peptides of Elongin C of the VCB complex in the APO, Binary with SiTX-0038404 (PROTAC 1), SiTX-0038405(PROTAC 2), SiTX-0038406(ACBI1) or Ternary complex with 404, 405, 406 + SMARCA2^{BD}.

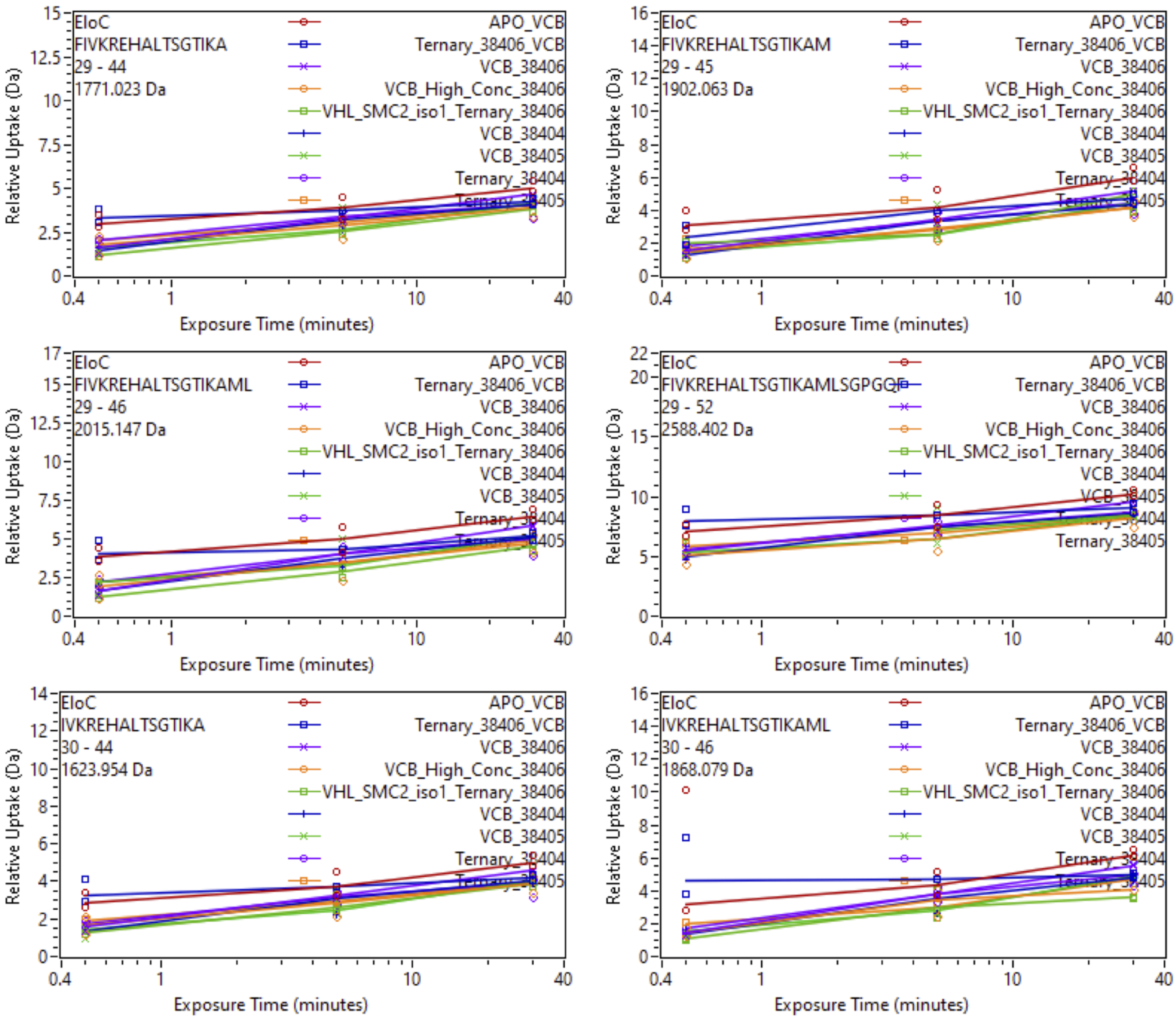

Relative deuterium uptake plots of peptic peptides of Elongin C of the VCB complex in the APO, Binary with SiTX-0038404 (PROTAC 1), SiTX-0038405(PROTAC 2), SiTX-0038406(ACBI1) or Ternary complex with 404, 405, 406 + SMARCA2^{BD}.

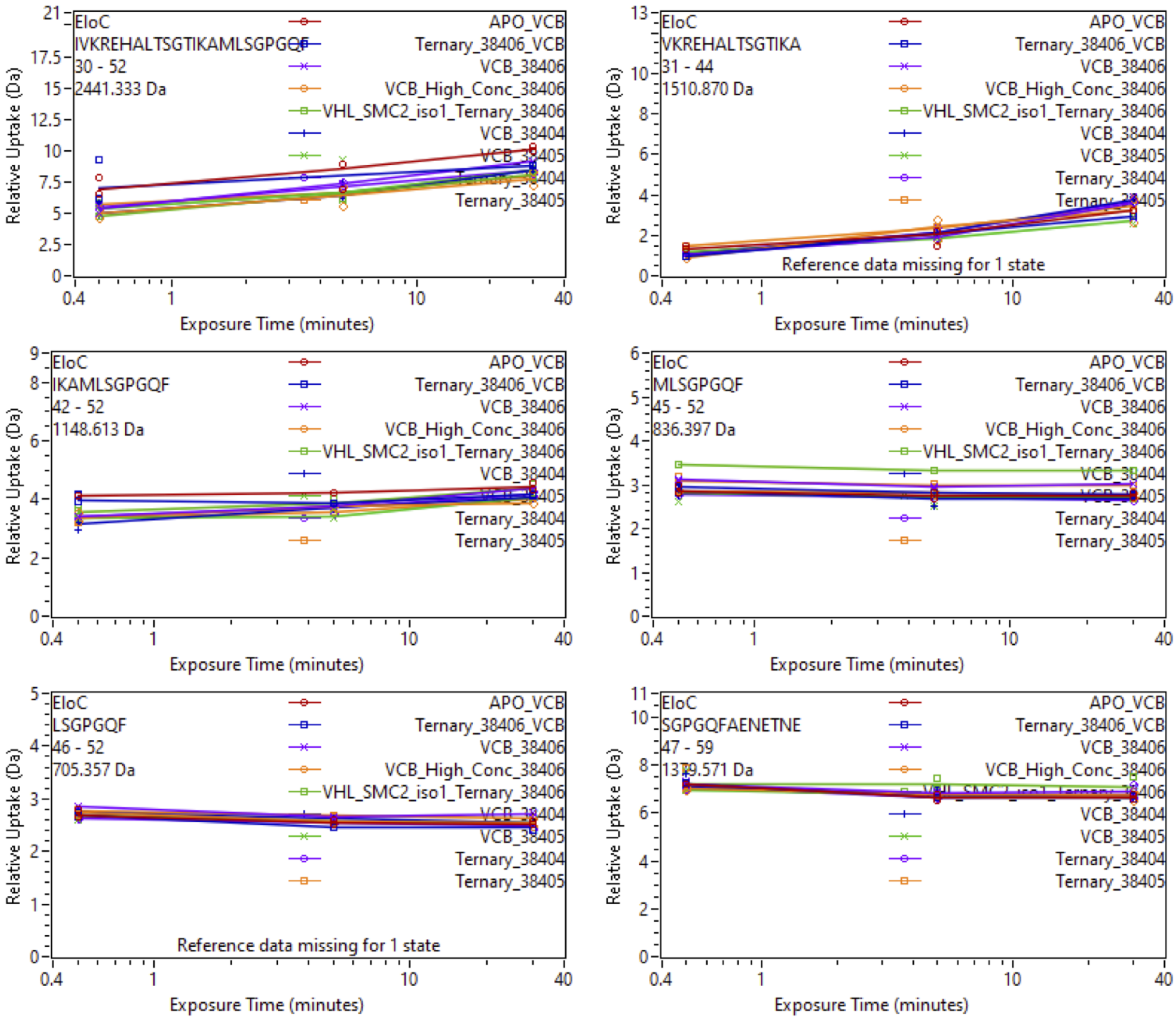

Relative deuterium uptake plots of peptic peptides of Elongin C of the VCB complex in the APO, Binary with SiTX-0038404 (PROTAC 1), SiTX-0038405(PROTAC 2), SiTX-0038406(ACBI1) or Ternary complex with 404, 405, 406 + SMARCA2^{BD}.

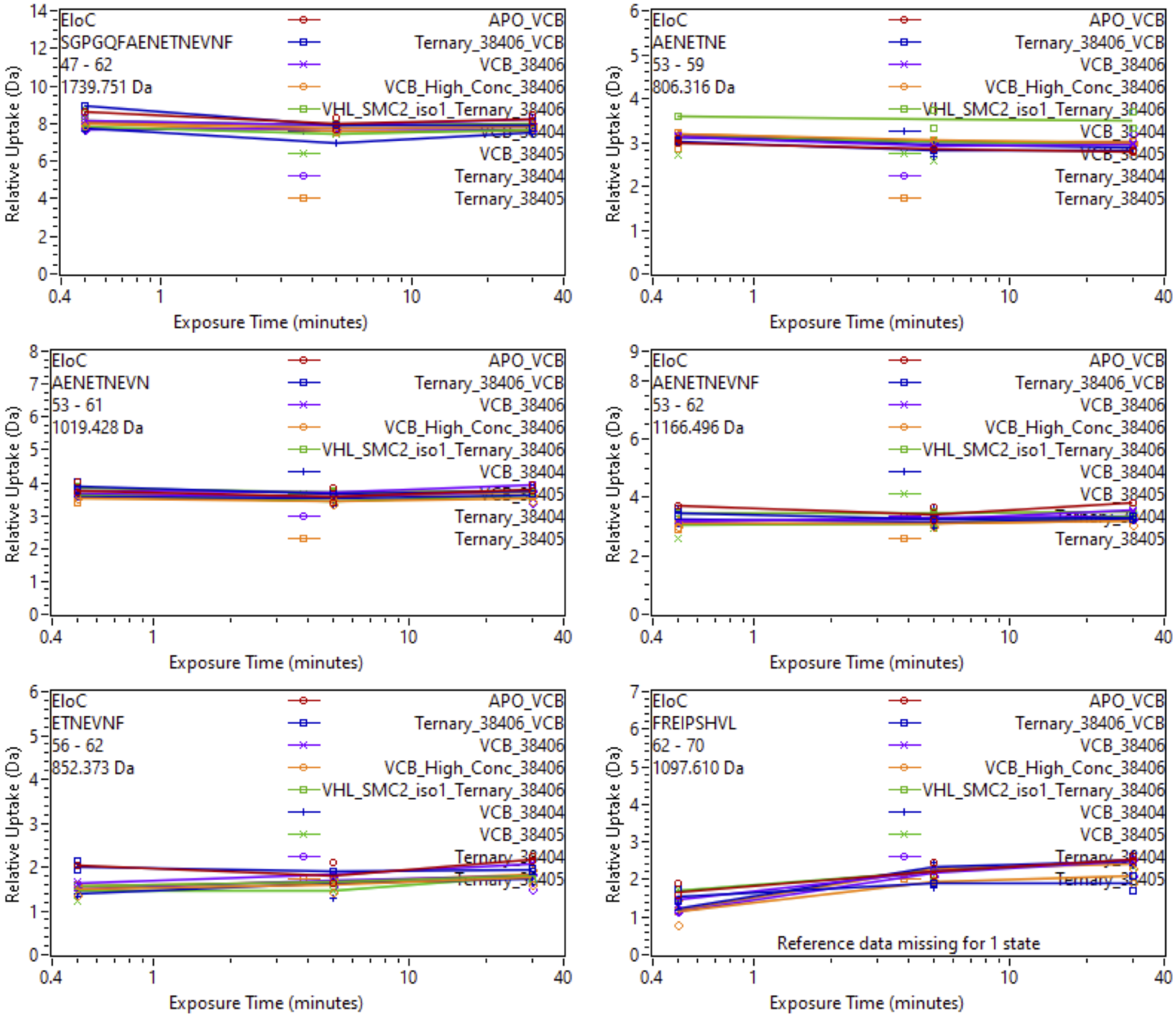

Relative deuterium uptake plots of peptic peptides of Elongin C of the VCB complex in the APO, Binary with SiTX-0038404 (PROTAC 1), SiTX-0038405(PROTAC 2), SiTX-0038406(ACBI1) or Ternary complex with 404, 405, 406 + SMARCA2^{BD}.

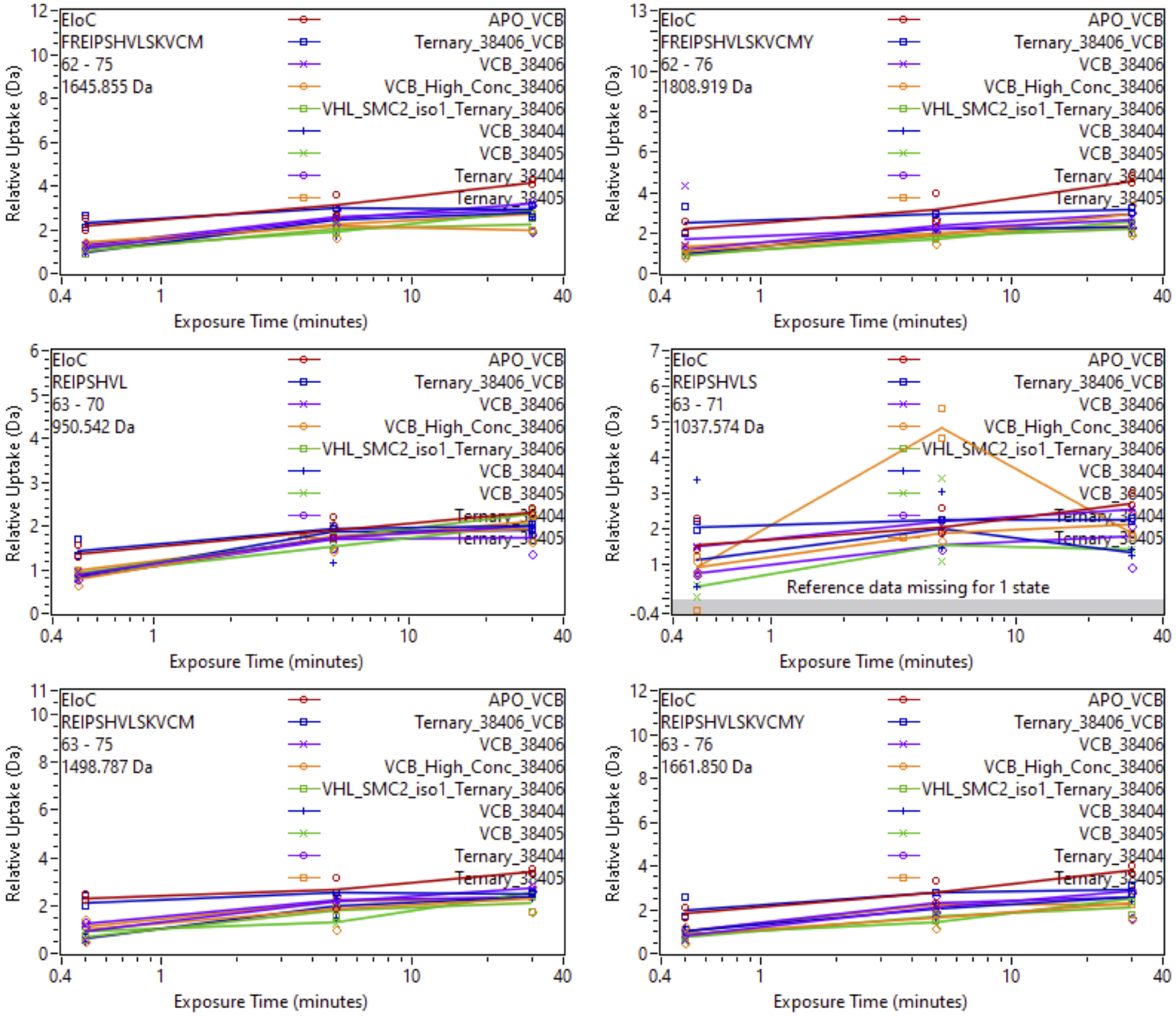

Relative deuterium uptake plots of peptic peptides of Elongin C of the VCB complex in the APO, Binary with SiTX-0038404 (PROTAC 1), SiTX-0038405(PROTAC 2), SiTX-0038406(ACBI1) or Ternary complex with 404, 405, 406 + SMARCA2^{BD}.

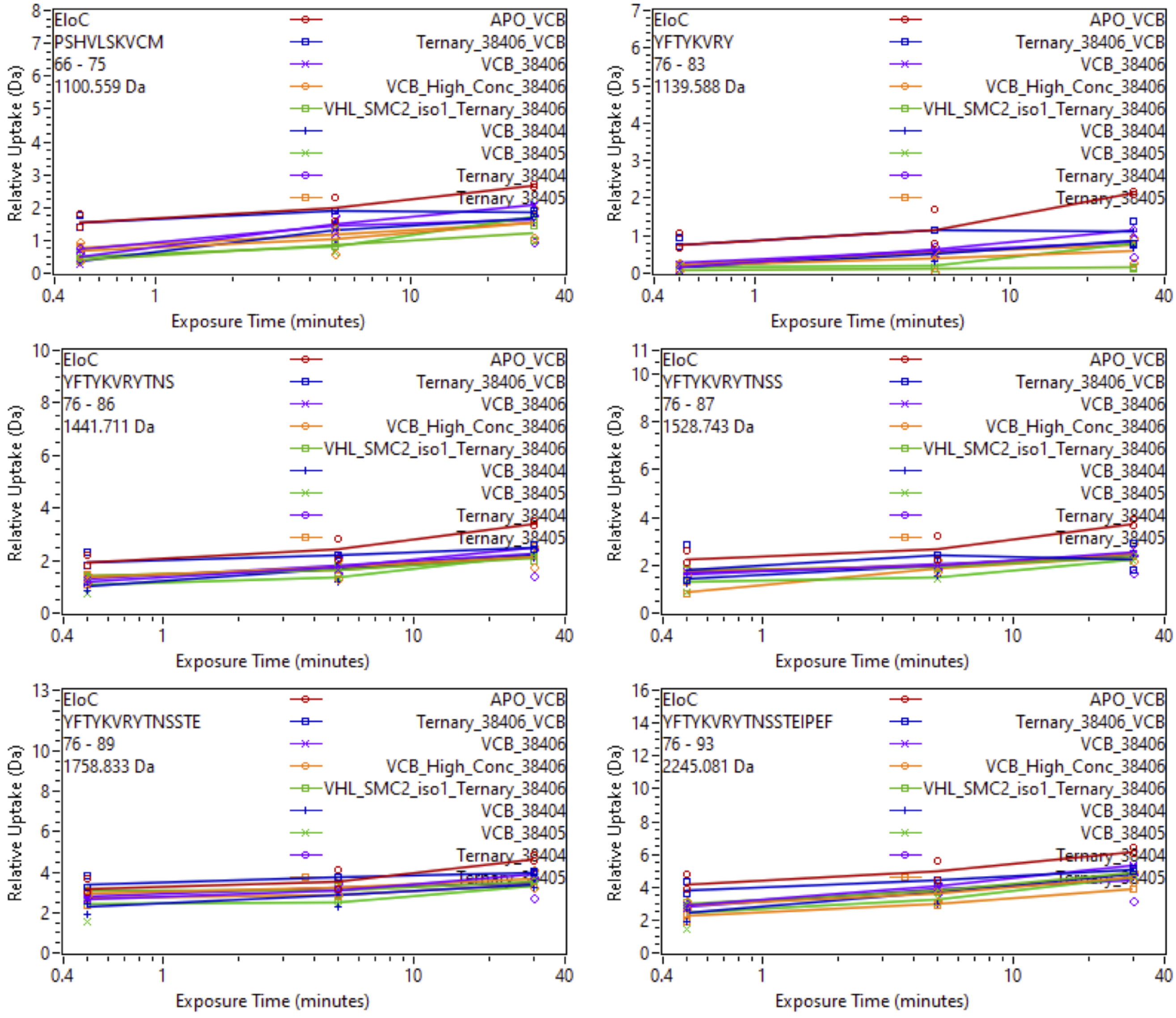

Relative deuterium uptake plots of peptic peptides of Elongin C of the VCB complex in the APO, Binary with SiTX-0038404 (PROTAC 1), SiTX-0038405(PROTAC 2), SiTX-0038406(ACBI1) or Ternary complex with 404, 405, 406 + SMARCA2^{BD}.

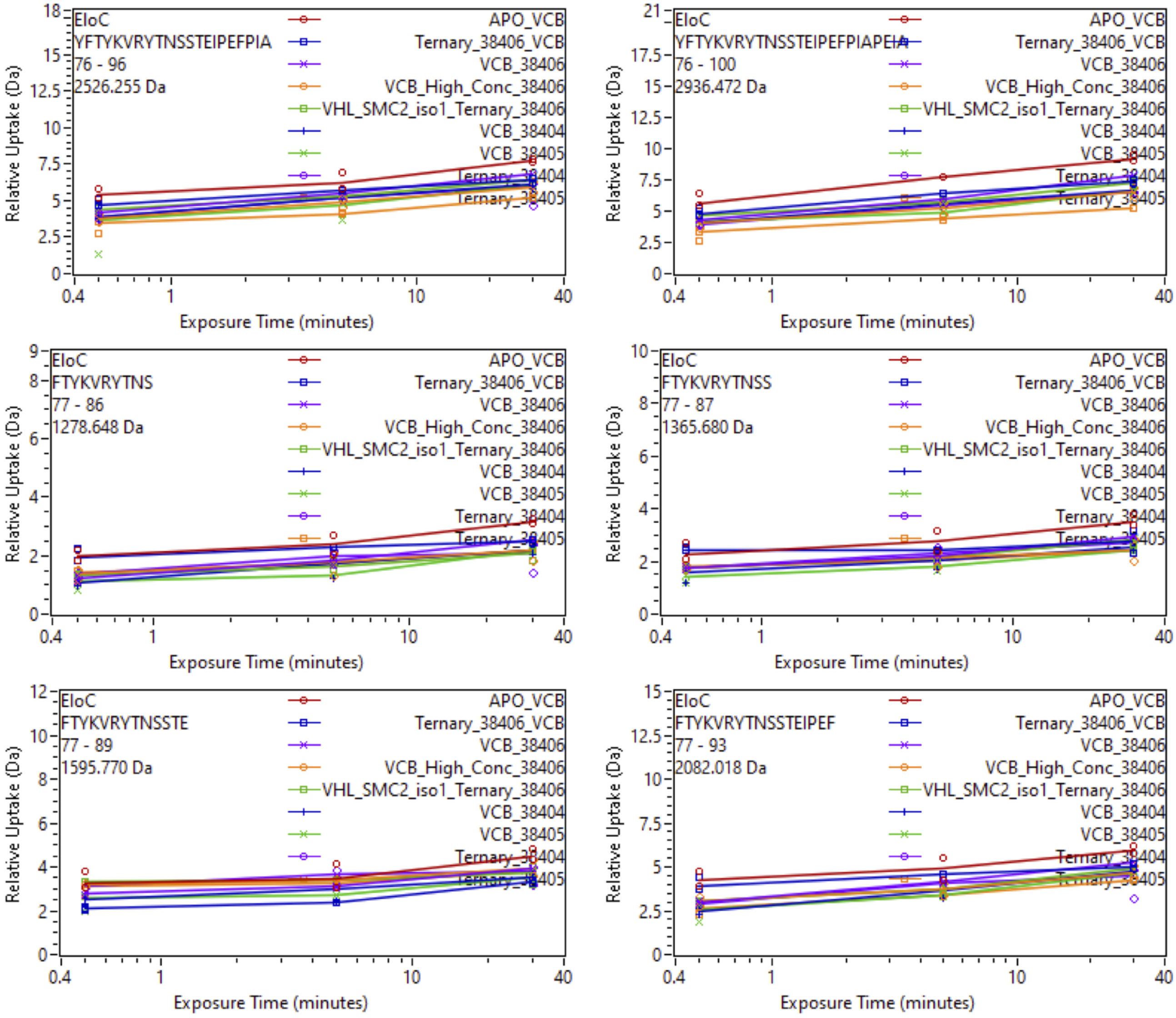

Relative deuterium uptake plots of peptic peptides of Elongin C of the VCB complex in the APO, Binary with SiTX-0038404 (PROTAC 1), SiTX-0038405(PROTAC 2), SiTX-0038406(ACBI1) or Ternary complex with 404, 405, 406 + SMARCA2^{BD}.

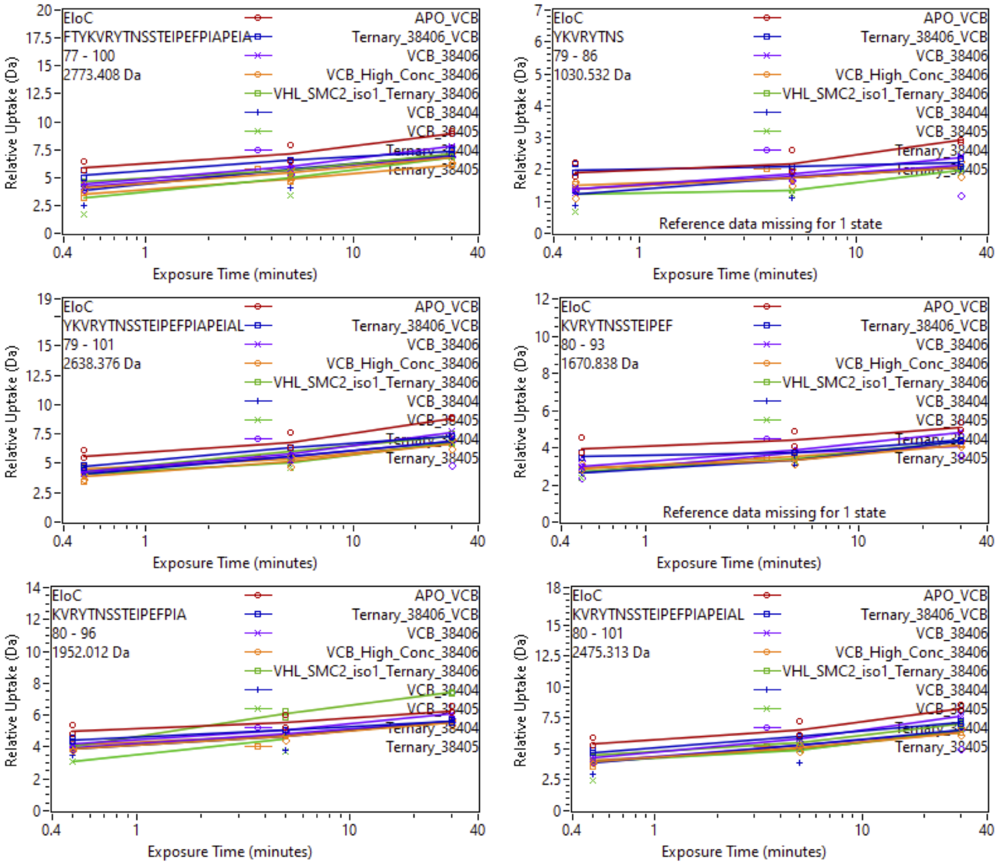

Relative deuterium uptake plots of peptic peptides of Elongin C of the VCB complex in the APO, Binary with SiTX-0038404 (PROTAC 1), SiTX-0038405(PROTAC 2), SiTX-0038406(ACBI1) or Ternary complex with 404, 405, 406 + SMARCA2^{BD}.

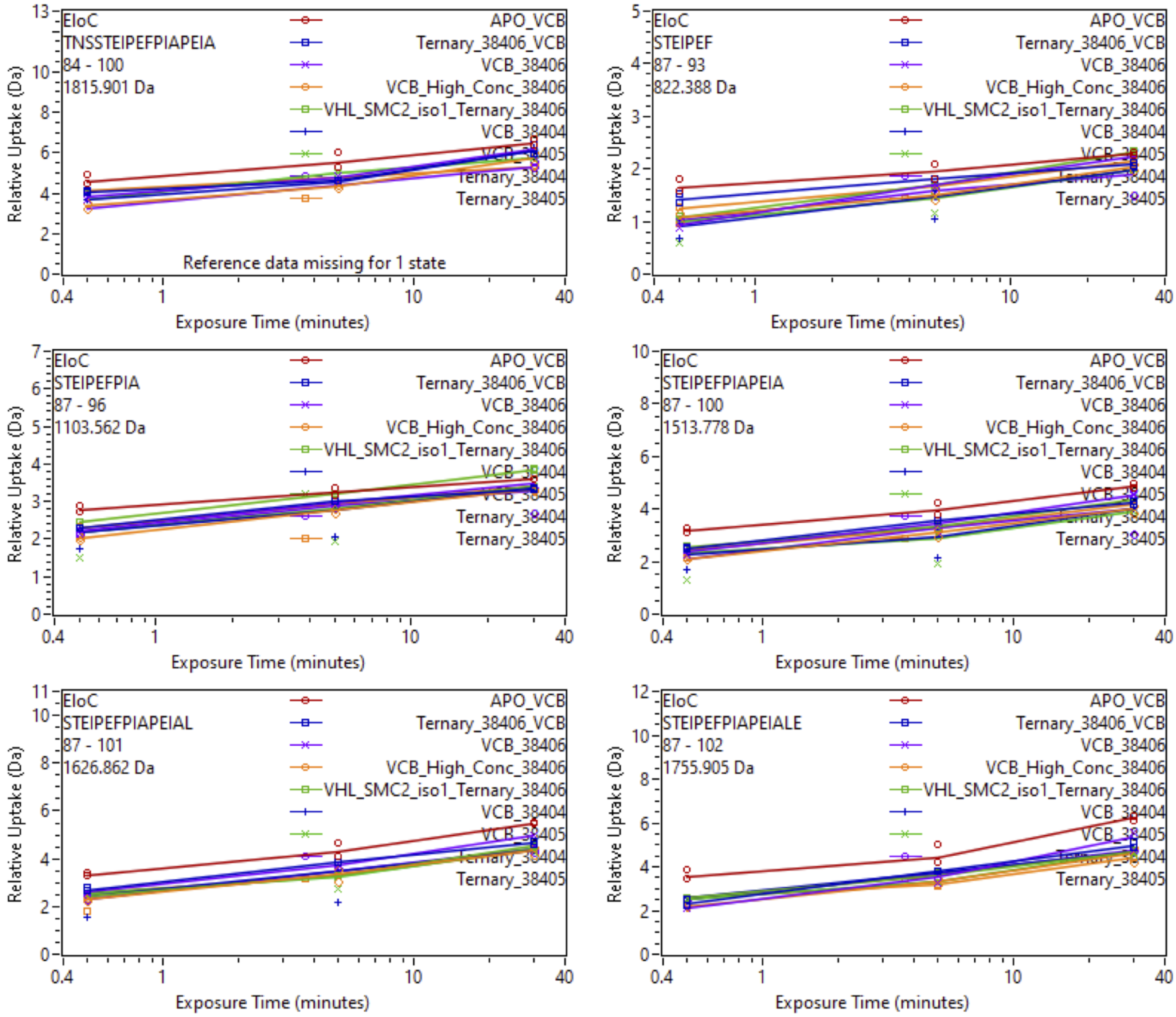

Relative deuterium uptake plots of peptic peptides of Elongin C of the VCB complex in the APO, Binary with SiTX-0038404 (PROTAC 1), SiTX-0038405(PROTAC 2), SiTX-0038406(ACBI1) or Ternary complex with 404, 405, 406 + SMARCA2^{BD}.

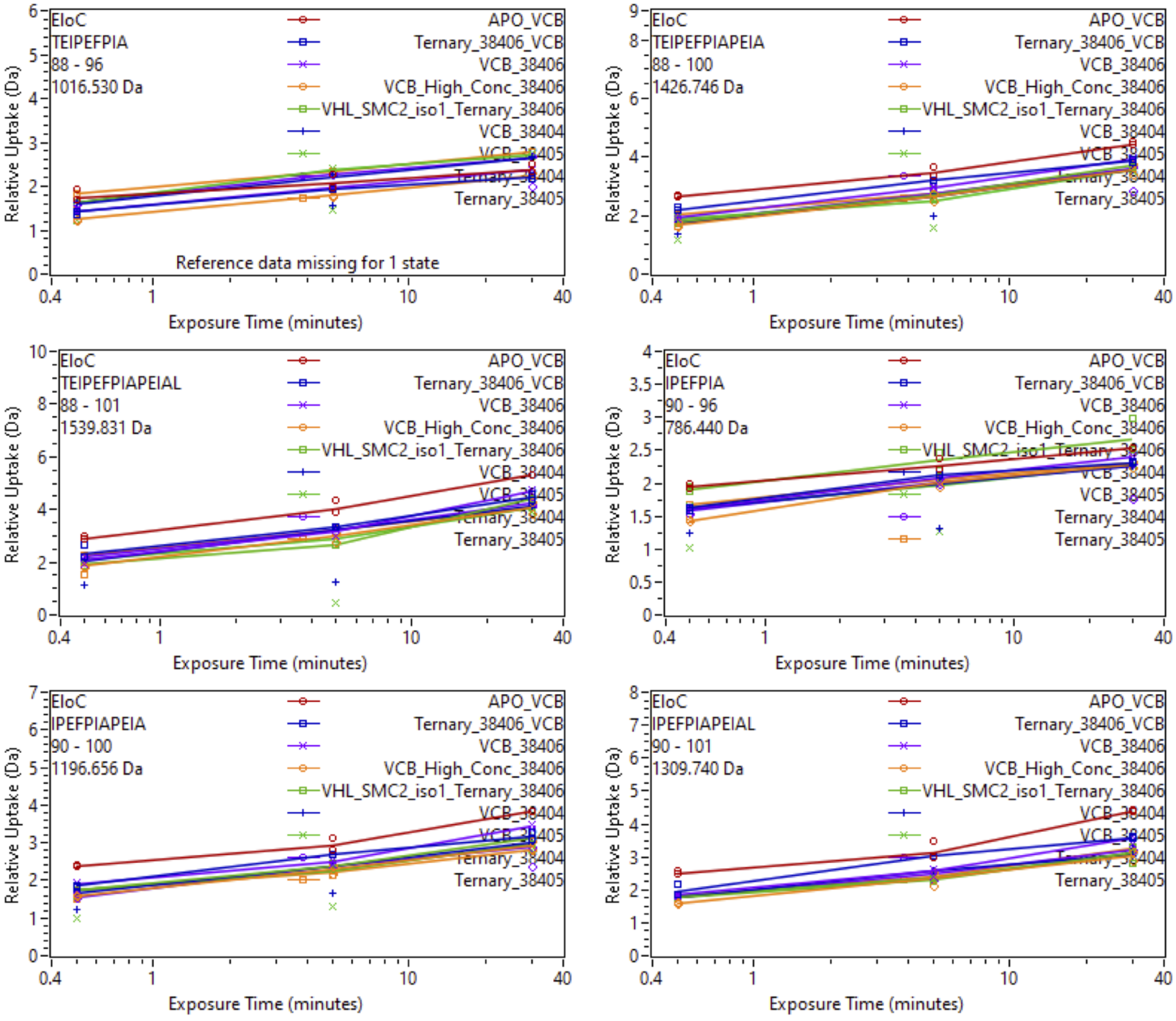

Relative deuterium uptake plots of peptic peptides of Elongin C of the VCB complex in the APO, Binary with SiTX-0038404 (PROTAC 1), SiTX-0038405(PROTAC 2), SiTX-0038406(ACBI1) or Ternary complex with 404, 405, 406 + SMARCA2^{BD}.

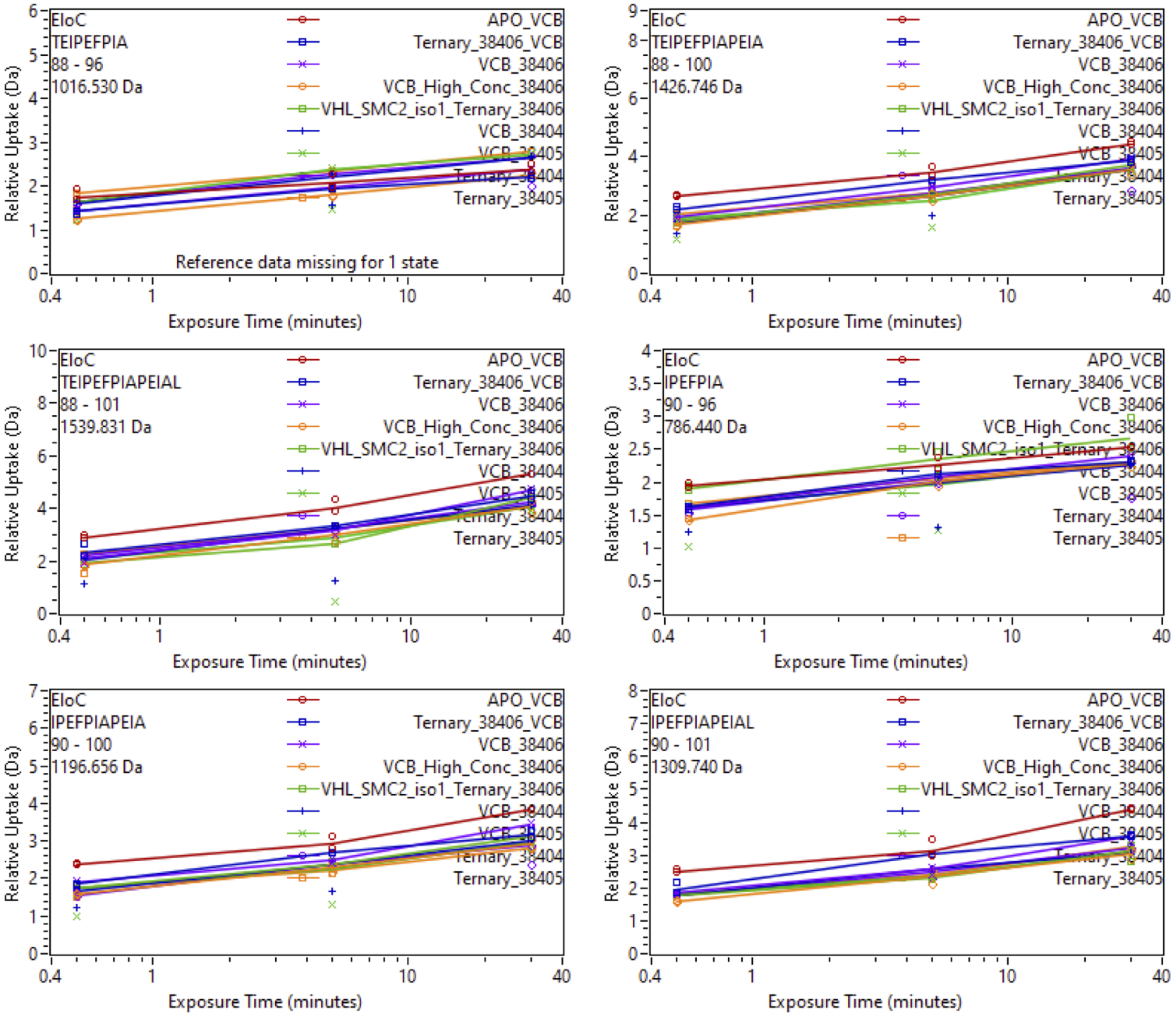

Relative deuterium uptake plots of peptic peptides of VHL of the VCB complex in the APO, Binary with SiTX-0038404 (PROTAC 1), SiTX-0038405(PROTAC 2), SiTX-0038406(ACBI1) or Ternary complex with 404, 405, 406 + SMARCA2^{BD}.

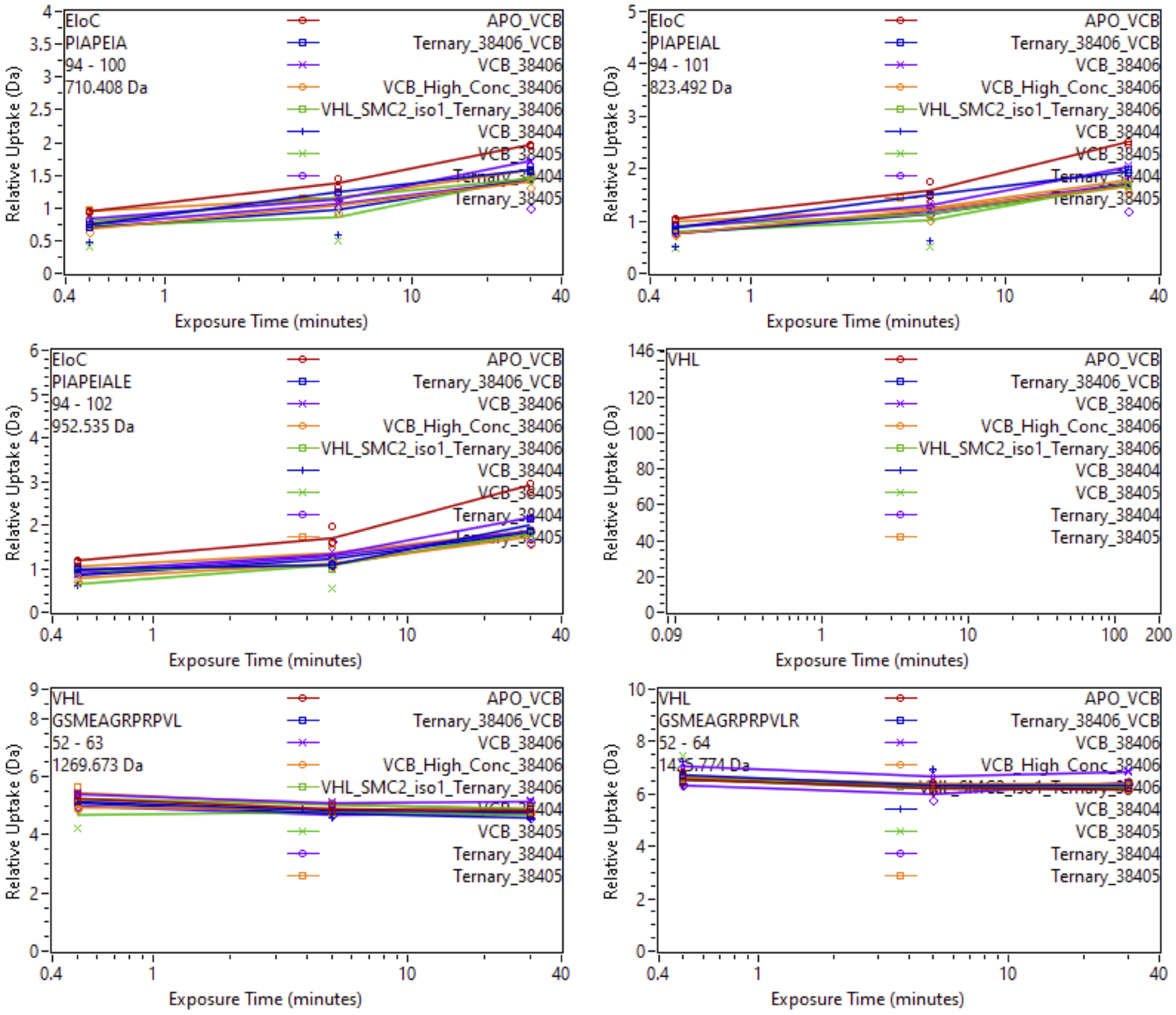

Relative deuterium uptake plots of peptic peptides of VHL of the VCB complex in the APO, Binary with SiTX-0038404 (PROTAC 1), SiTX-0038405(PROTAC 2), SiTX-0038406(ACBI1) or Ternary complex with 404, 405, 406 + SMARCA2^{BD}.

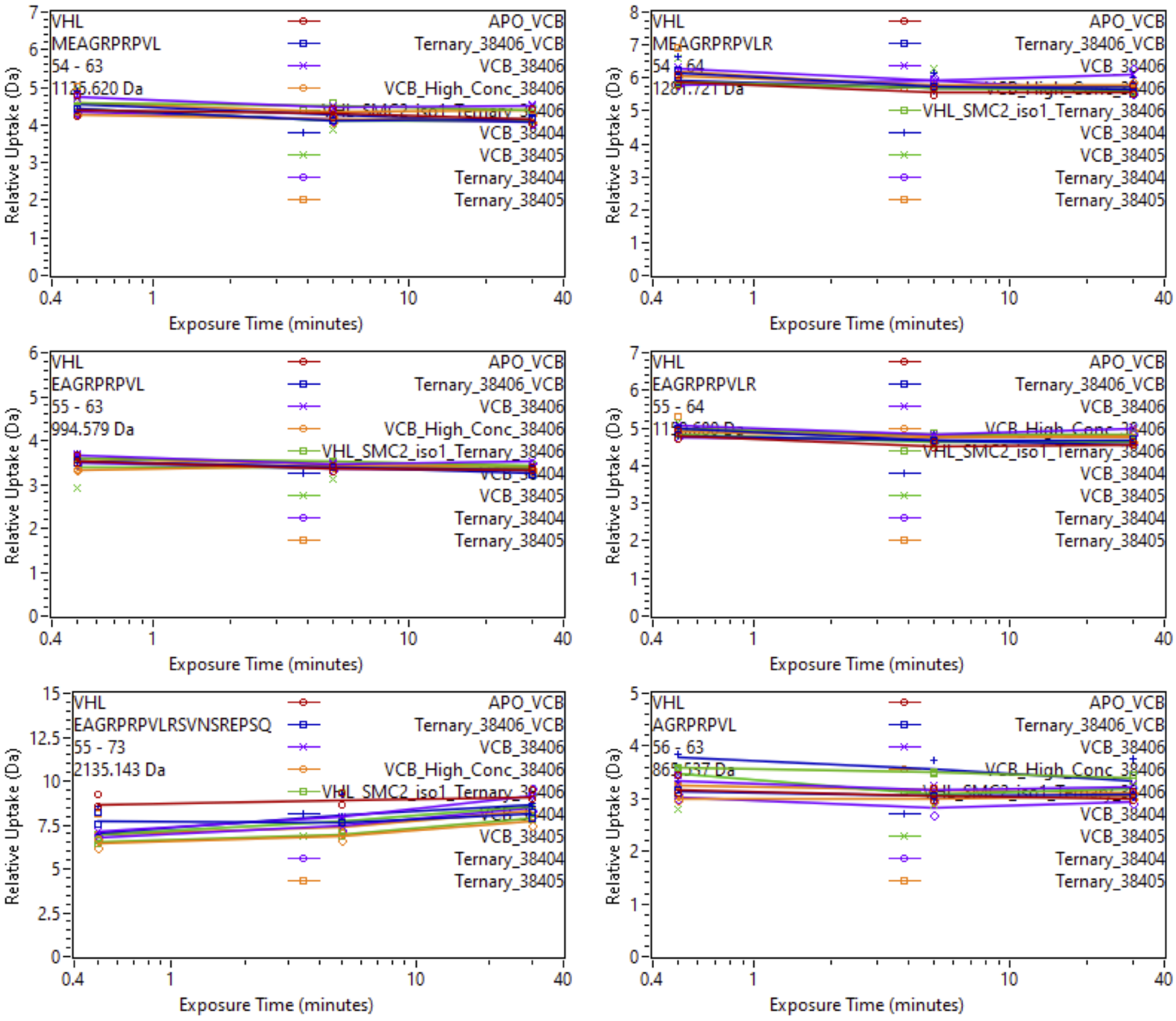

Relative deuterium uptake plots of peptic peptides of VHL of the VCB complex in the APO, Binary with SiTX-0038404 (PROTAC 1), SiTX-0038405(PROTAC 2), SiTX-0038406(ACBI1) or Ternary complex with 404, 405, 406 + SMARCA2^{BD}.

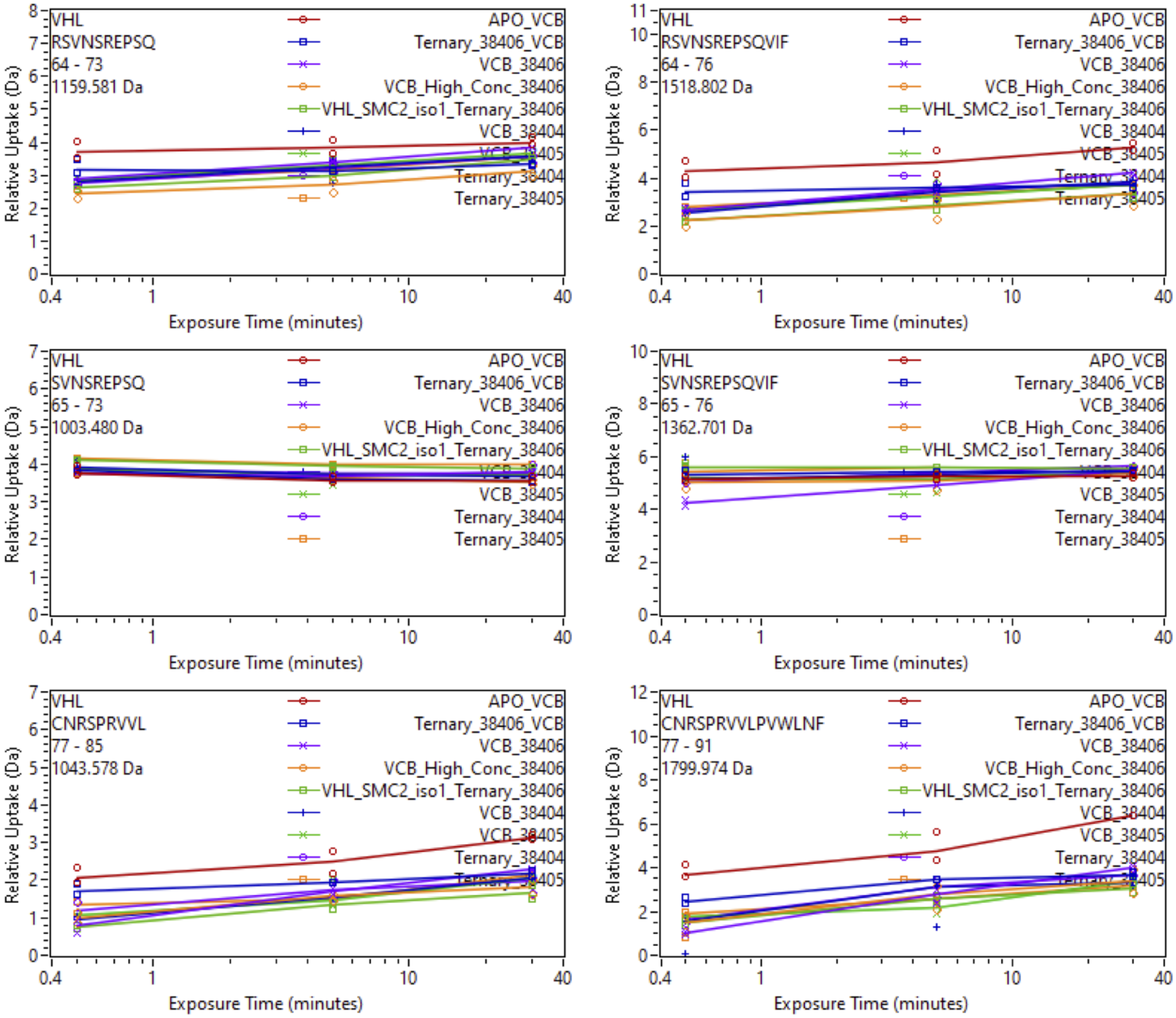

Relative deuterium uptake plots of peptic peptides of VHL of the VCB complex in the APO, Binary with SiTX-0038404 (PROTAC 1), SiTX-0038405(PROTAC 2), SiTX-0038406(ACBI1) or Ternary complex with 404, 405, 406 + SMARCA2^{BD}.

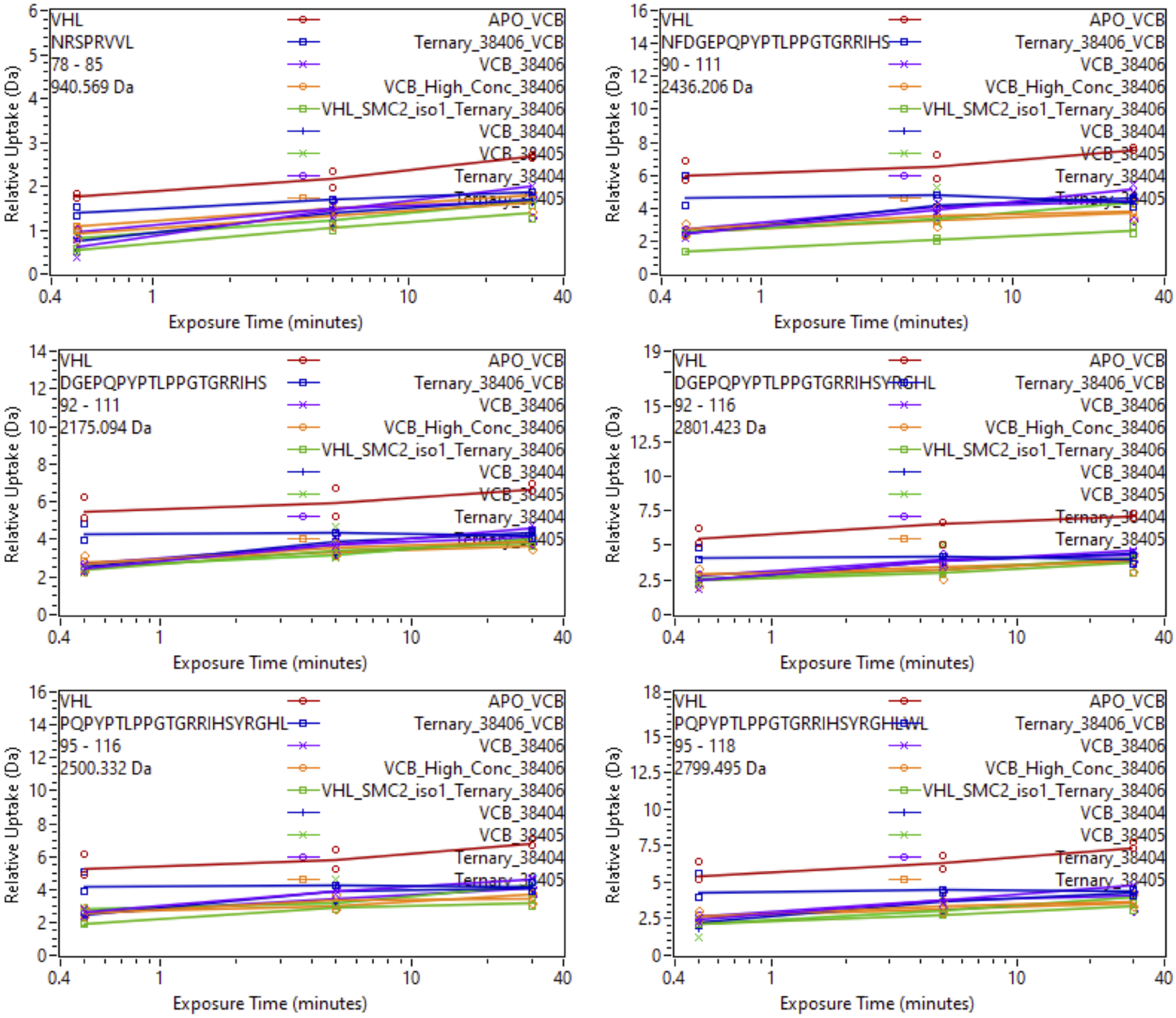

Relative deuterium uptake plots of peptic peptides of VHL of the VCB complex in the APO, Binary with SiTX-0038404 (PROTAC 1), SiTX-0038405(PROTAC 2), SiTX-0038406(ACBI1) or Ternary complex with 404, 405, 406 + SMARCA2^{BD}.

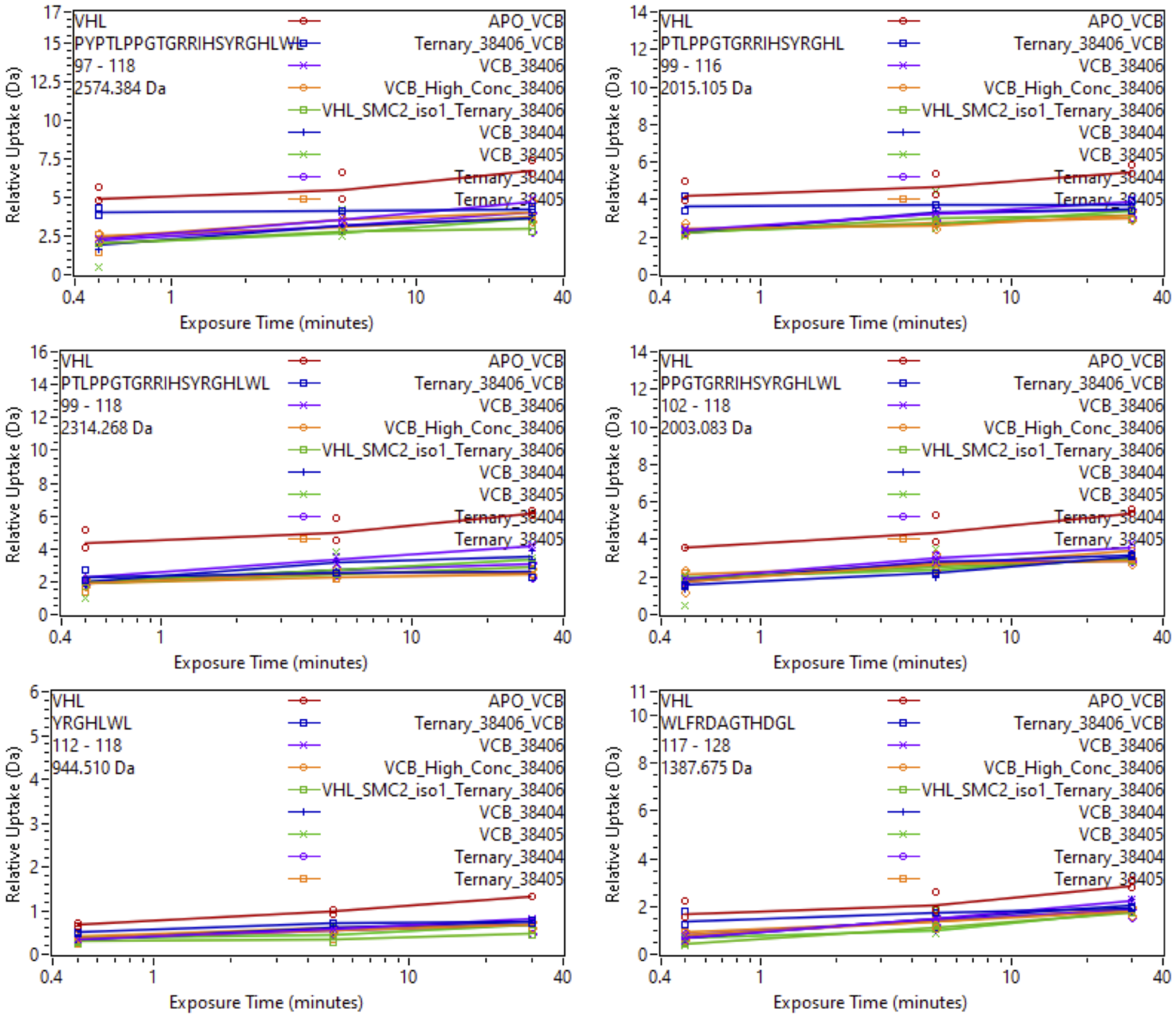

Relative deuterium uptake plots of peptic peptides of VHL of the VCB complex in the APO, Binary with SiTX-0038404 (PROTAC 1), SiTX-0038405(PROTAC 2), SiTX-0038406(ACBI1) or Ternary complex with 404, 405, 406 + SMARCA2^{BD}.

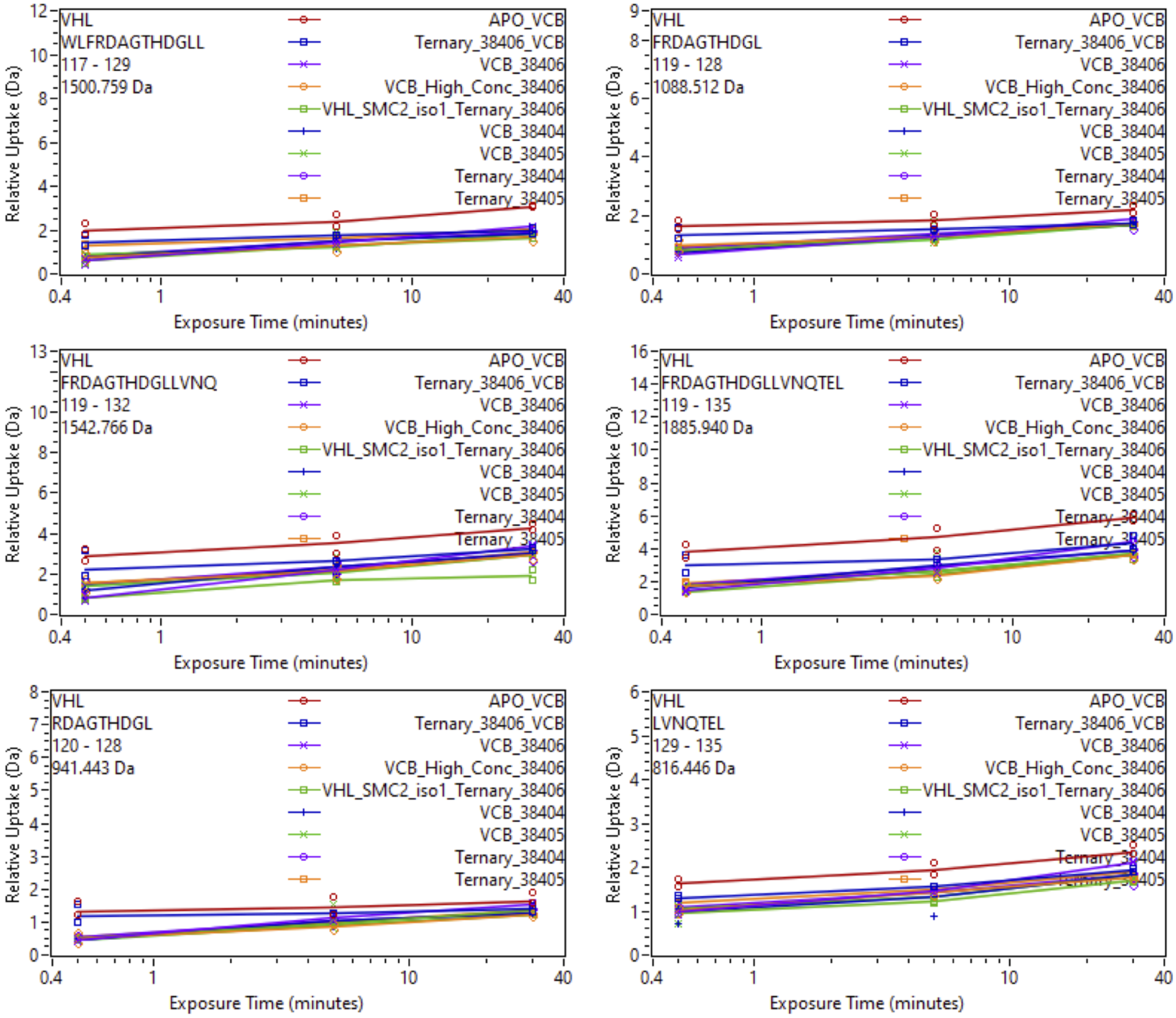

Relative deuterium uptake plots of peptic peptides of VHL of the VCB complex in the APO, Binary with SiTX-0038404 (PROTAC 1), SiTX-0038405(PROTAC 2), SiTX-0038406(ACBI1) or Ternary complex with 404, 405, 406 + SMARCA2^{BD}.

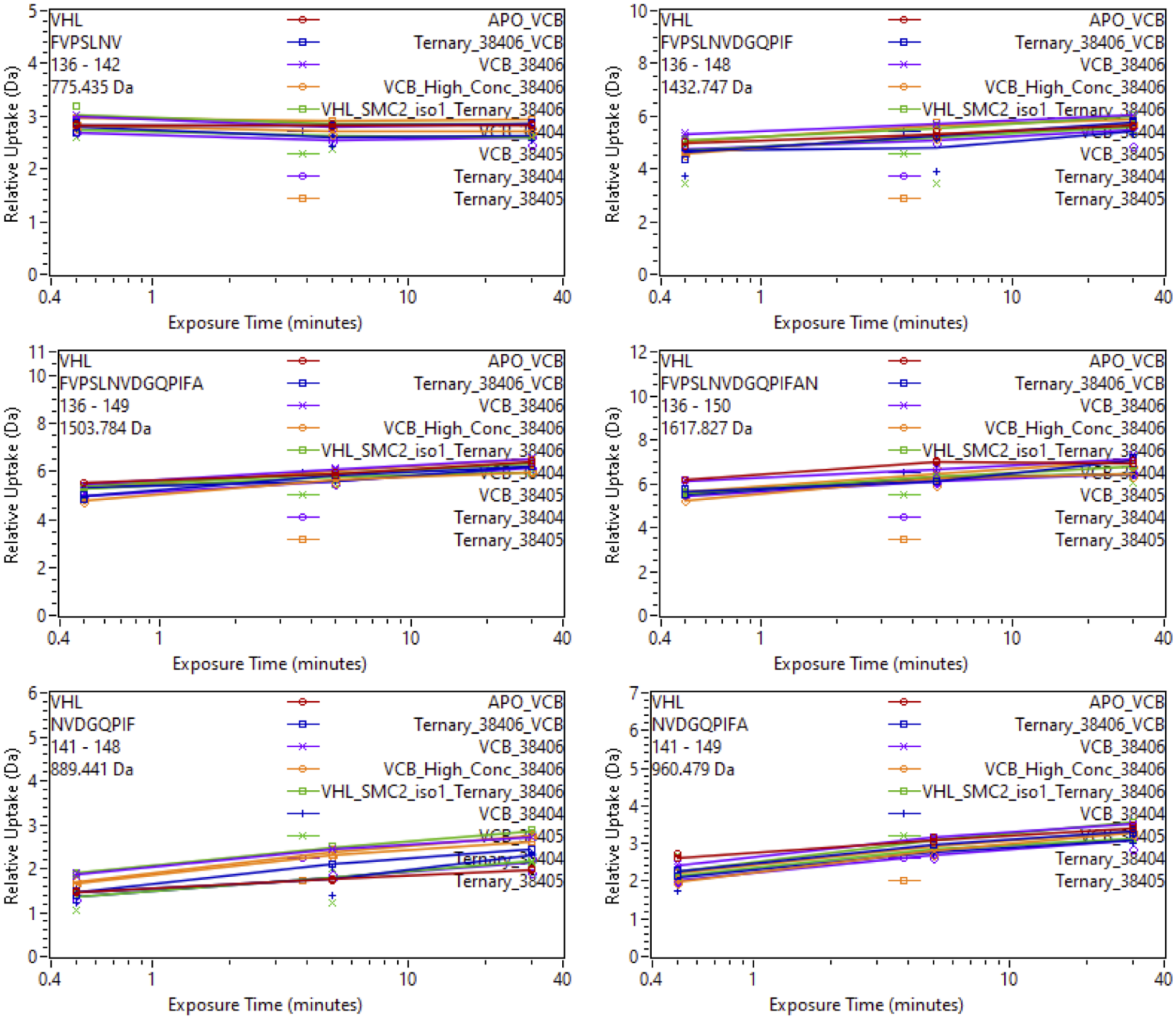

Relative deuterium uptake plots of peptic peptides of VHL of the VCB complex in the APO, Binary with SiTX-0038404 (PROTAC 1), SiTX-0038405(PROTAC 2), SiTX-0038406(ACBI1) or Ternary complex with 404, 405, 406 + SMARCA2^{BD}.

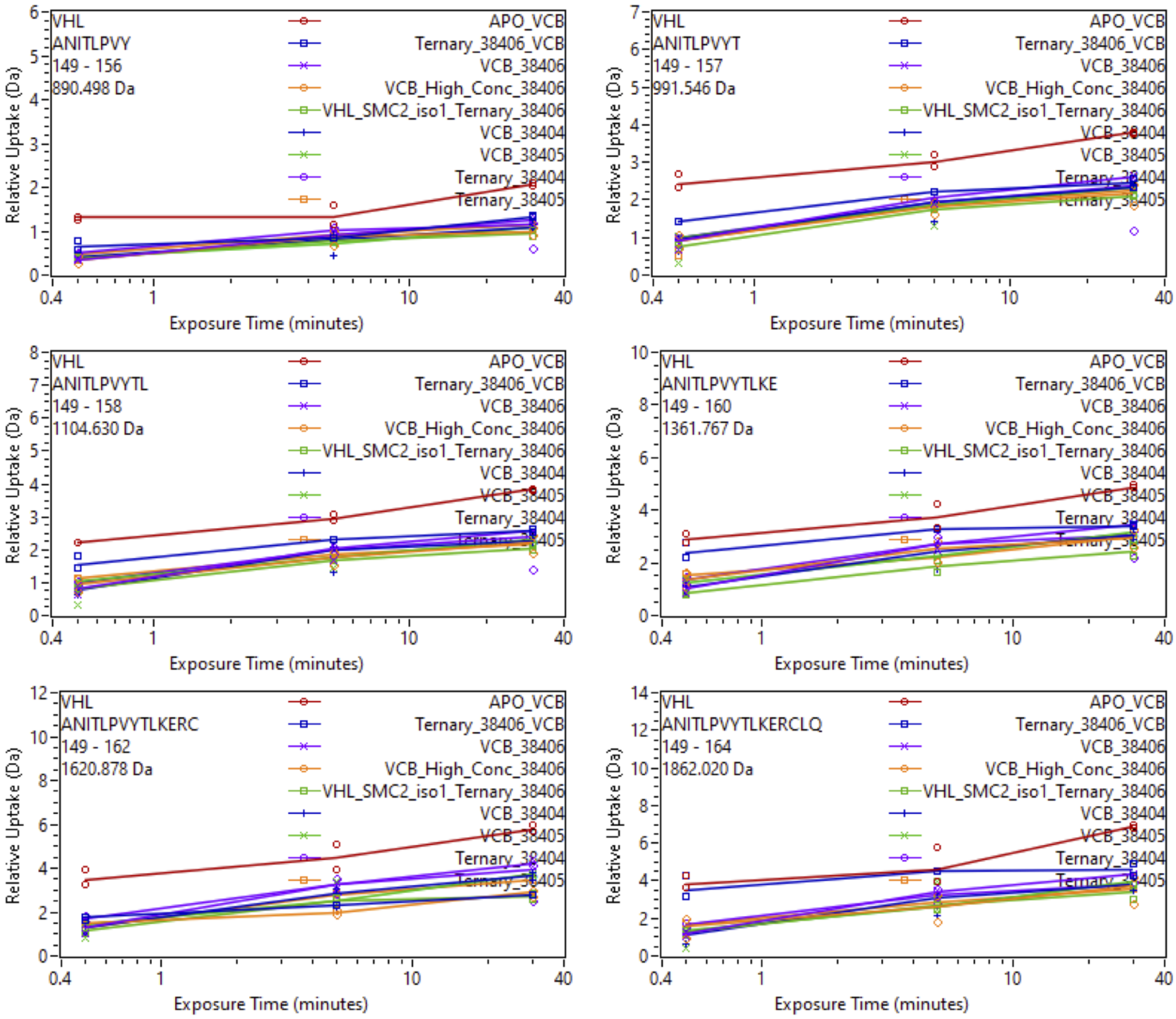

Relative deuterium uptake plots of peptic peptides of VHL of the VCB complex in the APO, Binary with SiTX-0038404 (PROTAC 1), SiTX-0038405(PROTAC 2), SiTX-0038406(ACBI1) or Ternary complex with 404, 405, 406 + SMARCA2^{BD}.

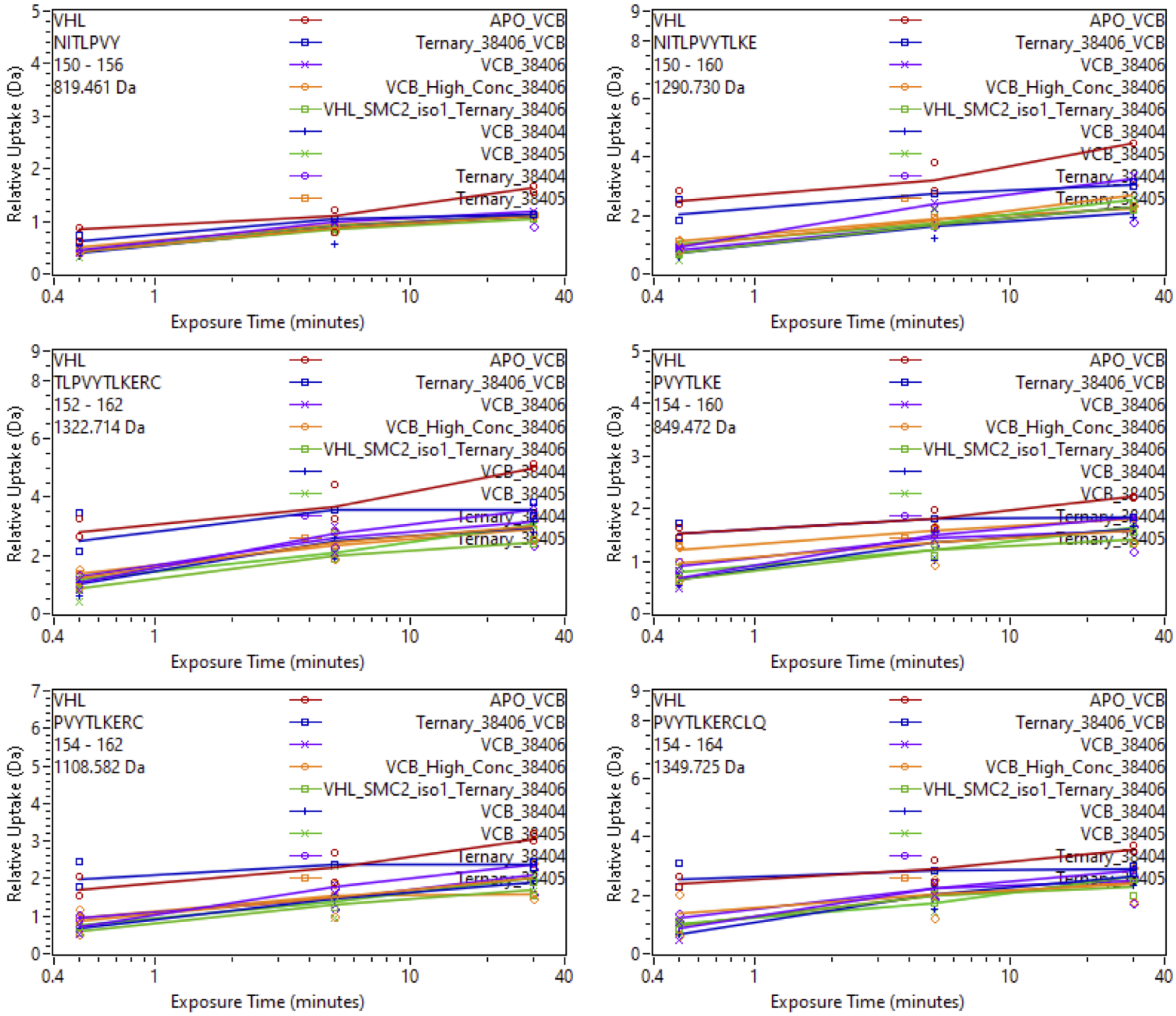

Relative deuterium uptake plots of peptic peptides of VHL of the VCB complex in the APO, Binary with SiTX-0038404 (PROTAC 1), SiTX-0038405(PROTAC 2), SiTX-0038406(ACBI1) or Ternary complex with 404, 405, 406 + SMARCA2^{BD}.

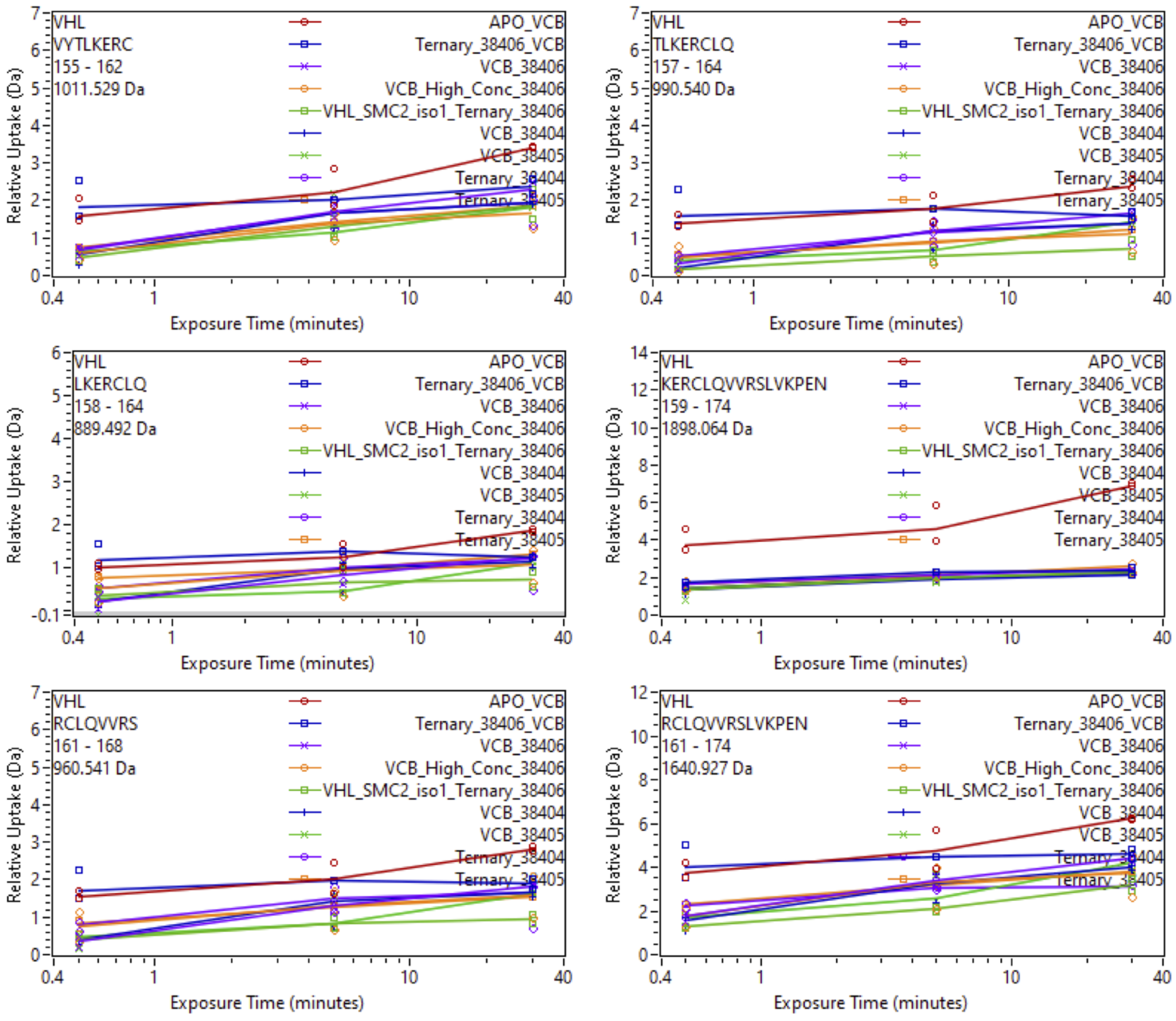

Relative deuterium uptake plots of peptic peptides of VHL of the VCB complex in the APO, Binary with SiTX-0038404 (PROTAC 1), SiTX-0038405(PROTAC 2), SiTX-0038406(ACBI1) or Ternary complex with 404, 405, 406 + SMARCA2^{BD}.

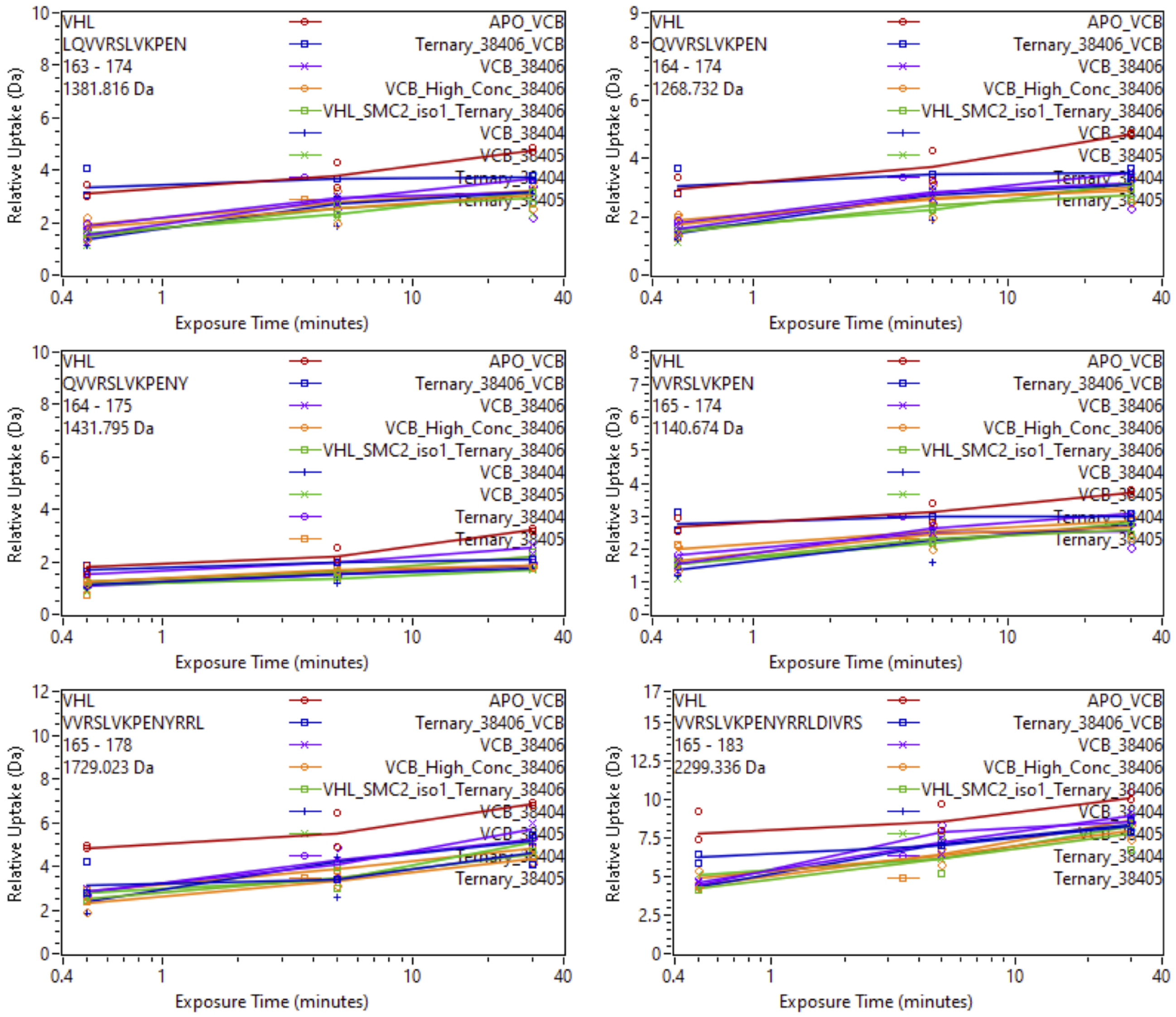

Relative deuterium uptake plots of peptic peptides of VHL of the VCB complex in the APO, Binary with SiTX-0038404 (PROTAC 1), SiTX-0038405(PROTAC 2), SiTX-0038406(ACBI1) or Ternary complex with 404, 405, 406 + SMARCA2^{BD}.

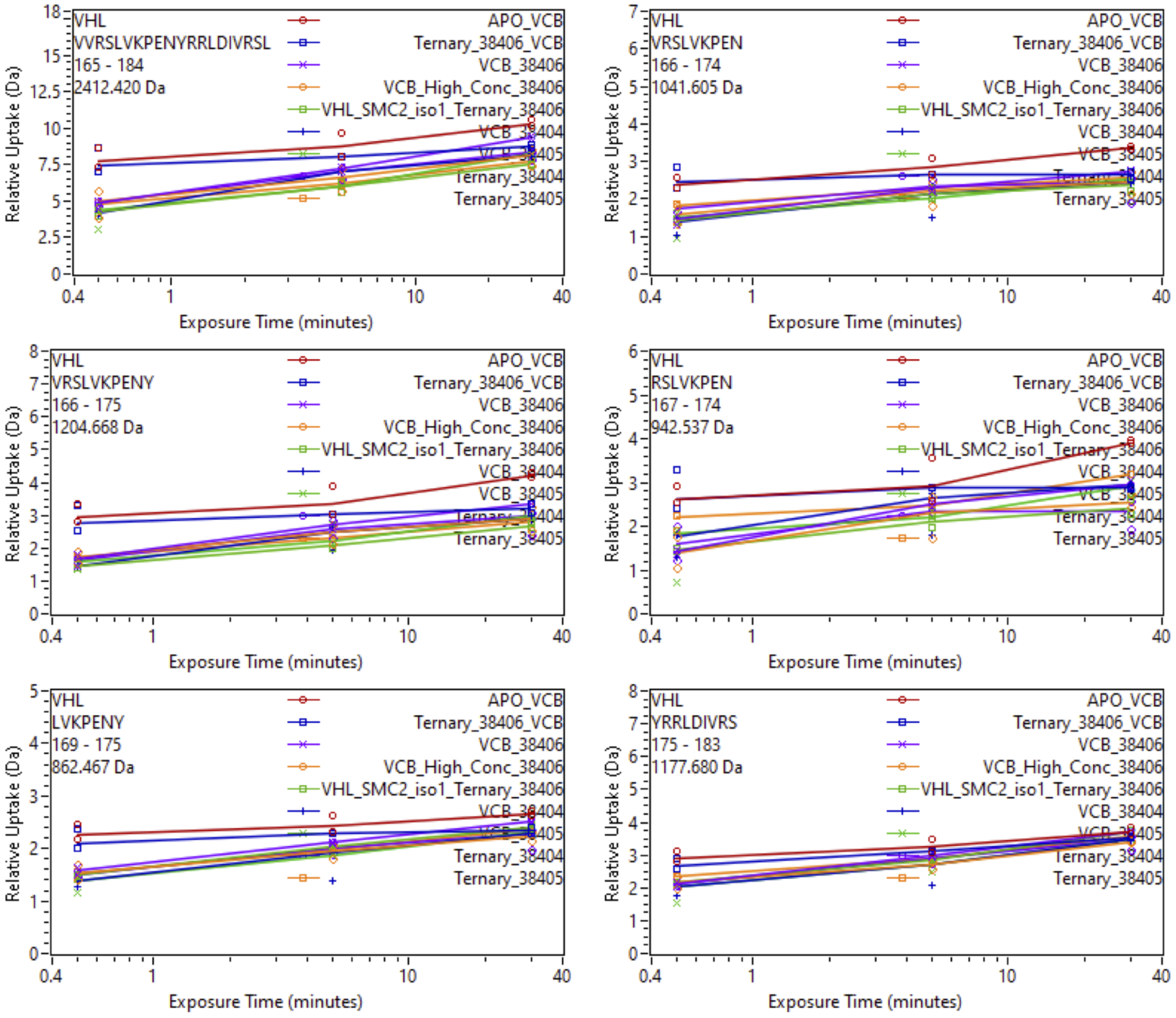

Relative deuterium uptake plots of peptic peptides of VHL of the VCB complex in the APO, Binary with SiTX-0038404 (PROTAC 1), SiTX-0038405(PROTAC 2), SiTX-0038406(ACBI1) or Ternary complex with 404, 405, 406 + SMARCA2^{BD}.

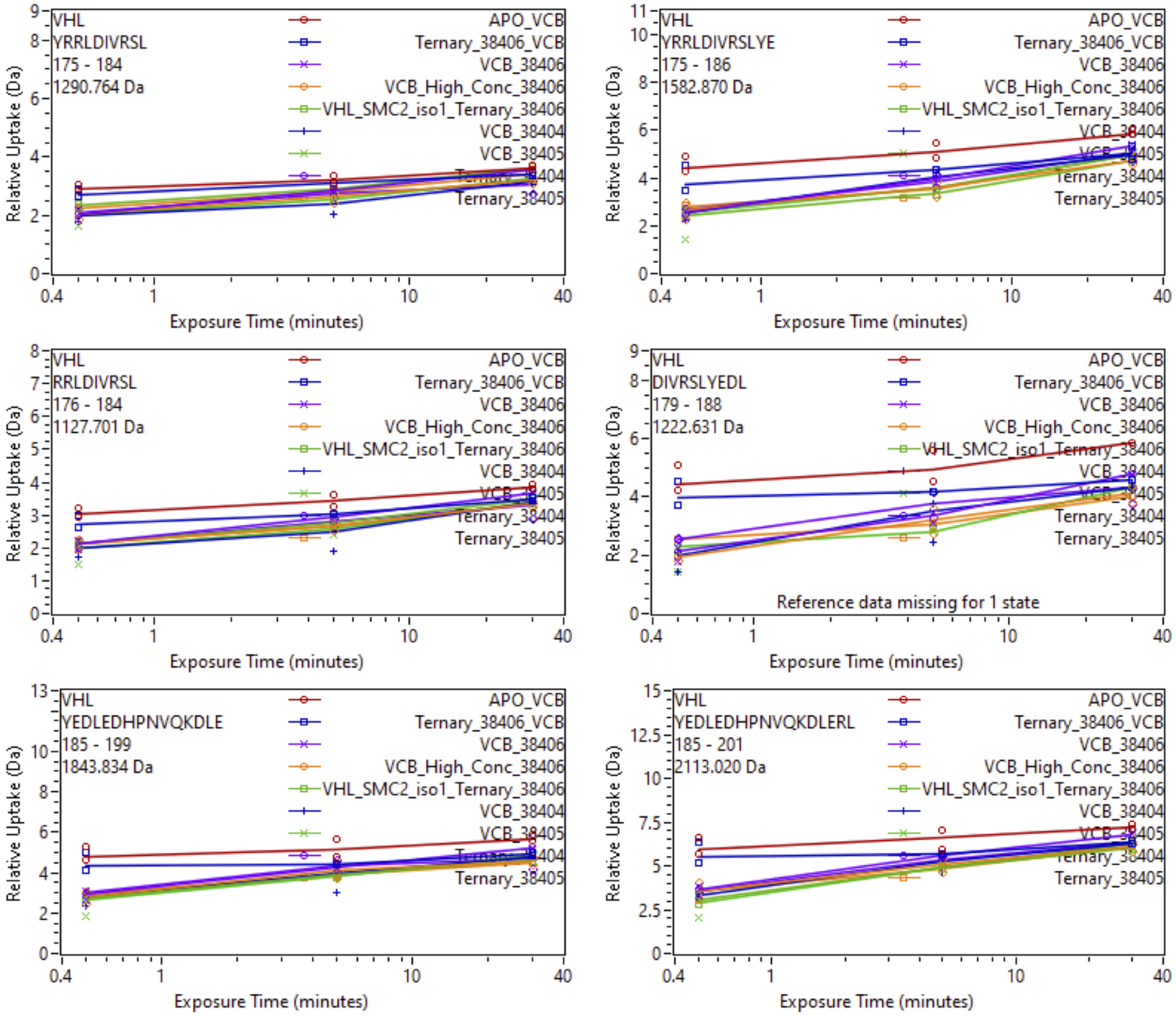

Relative deuterium uptake plots of peptic peptides of VHL of the VCB complex in the APO, Binary with SiTX-0038404 (PROTAC 1), SiTX-0038405(PROTAC 2), SiTX-0038406(ACBI1) or Ternary complex with 404, 405, 406 + SMARCA2^{BD}.

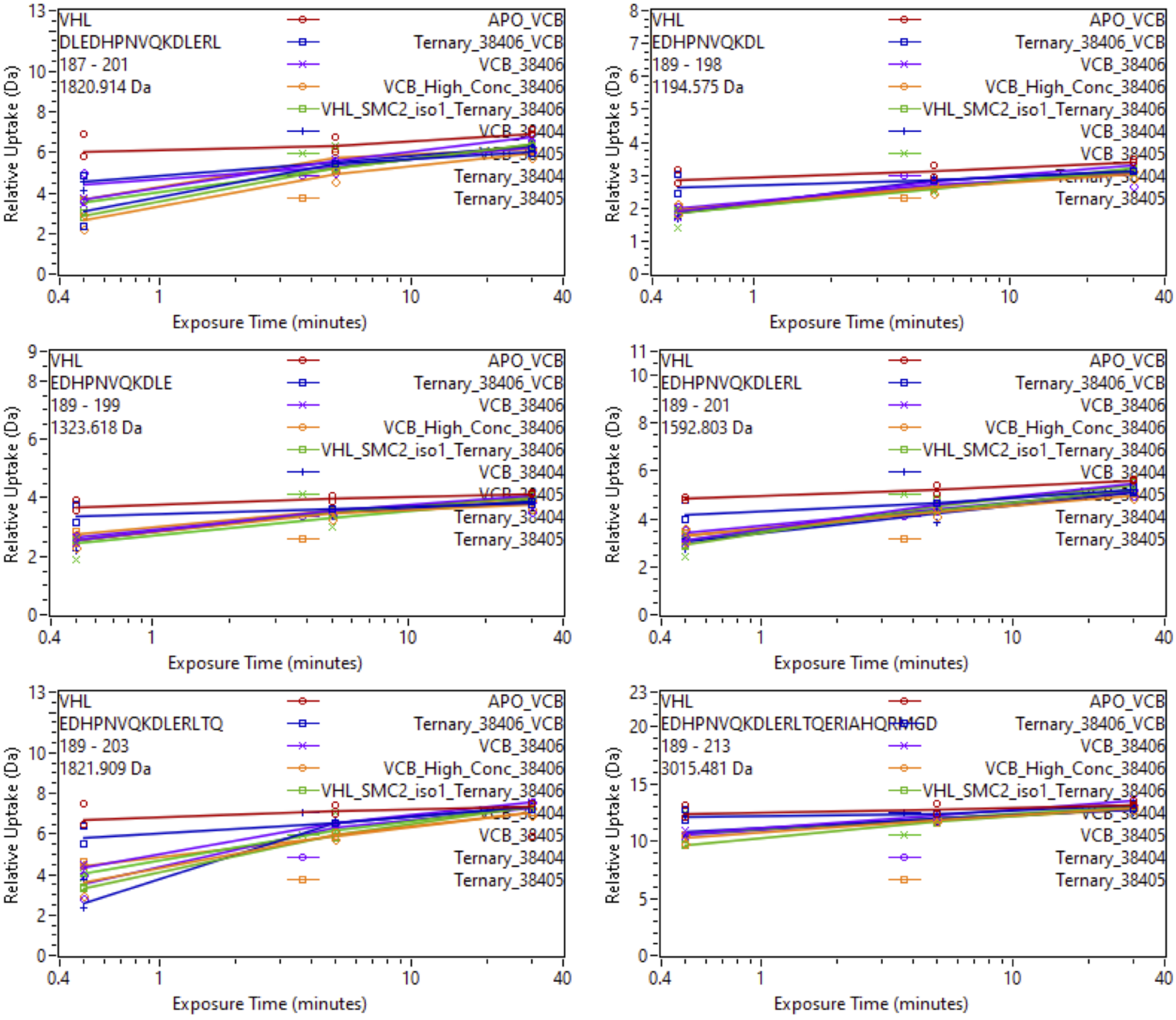

Relative deuterium uptake plots of peptic peptides of VHL of the VCB complex in the APO, Binary with SiTX-0038404 (PROTAC 1), SiTX-0038405(PROTAC 2), SiTX-0038406(ACBI1) or Ternary complex with 404, 405, 406 + SMARCA2^{BD}.

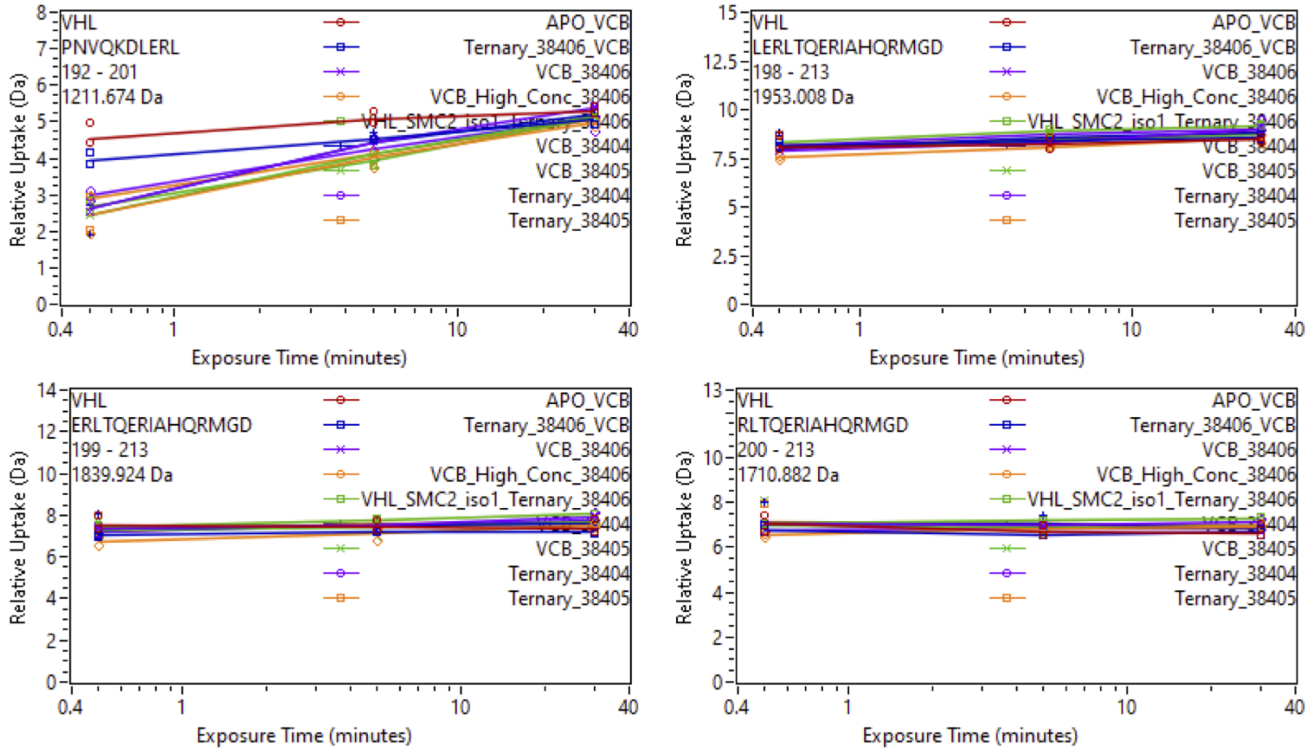

Relative deuterium uptake plots of peptic peptides of VHL of the VCB complex in the APO, Binary with SiTX-0038404 (PROTAC 1), SiTX-0038405(PROTAC 2), SiTX-0038406(ACBI1) or Ternary complex with 404, 405, 406 + SMARCA2^{BD}.

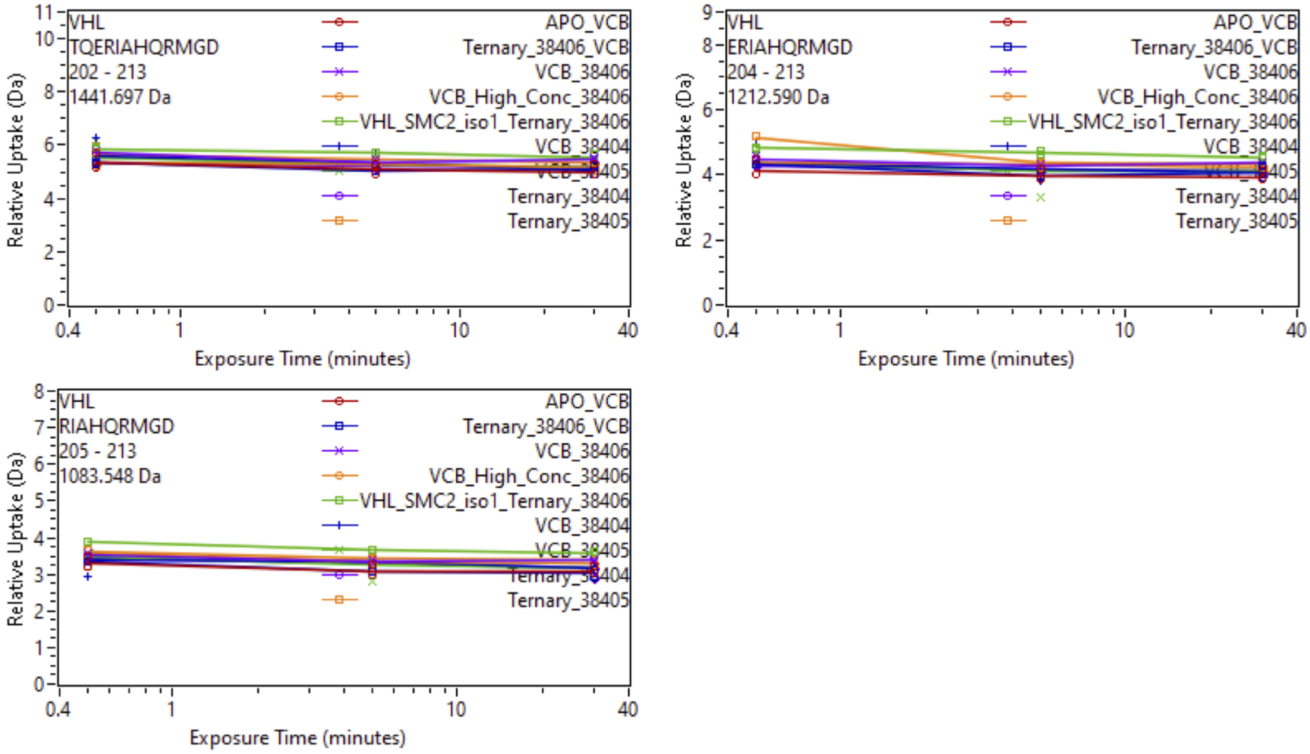

Relative deuterium uptake plots of peptic peptides of VHL of the VCB complex in the APO, Binary with SiTX-0038404 (PROTAC 1), SiTX-0038405(PROTAC 2), SiTX-0038406(ACBI1) or Ternary complex with 404, 405, 406 + SMARCA2^{BD}.

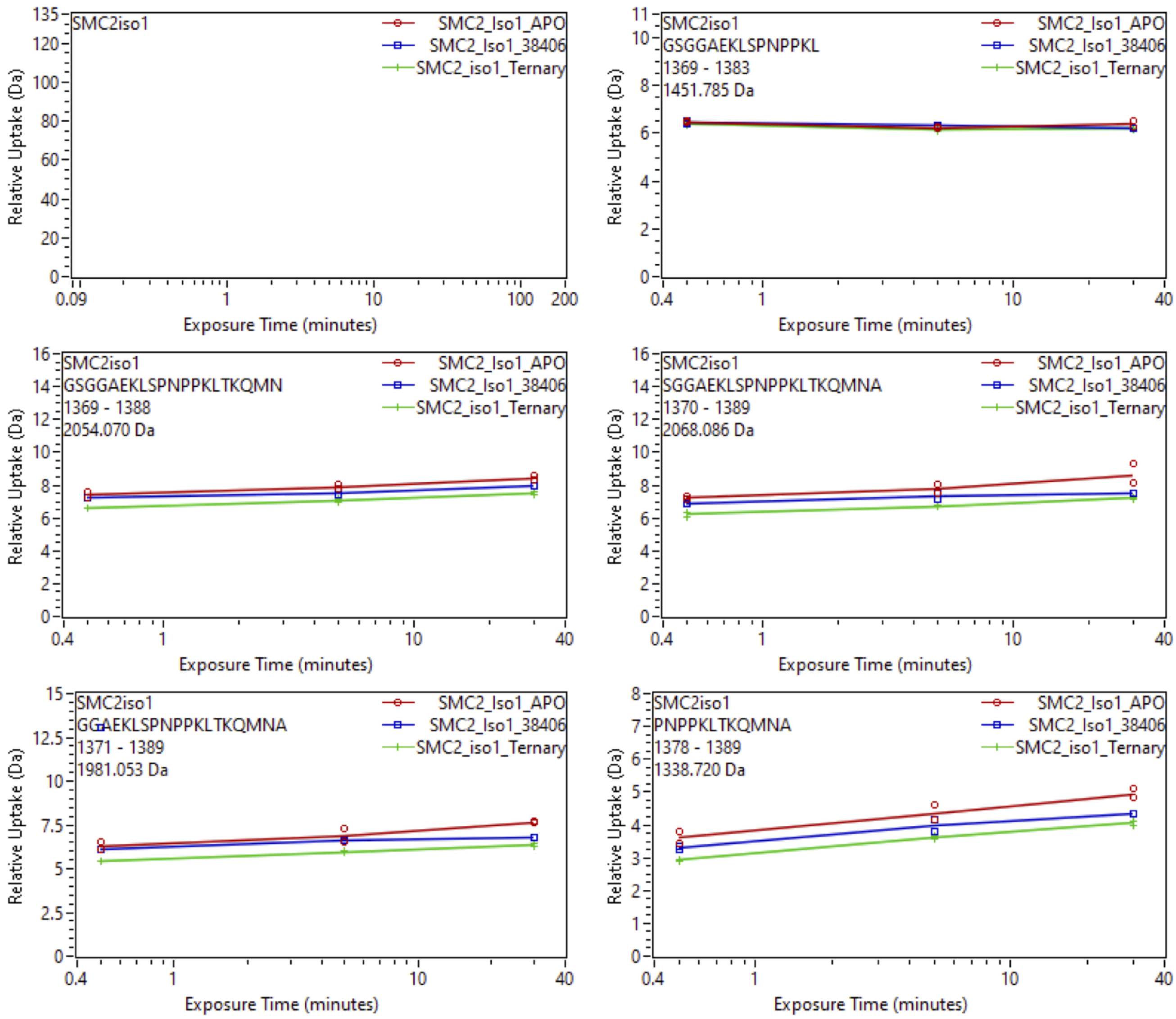

Relative deuterium uptake plots of peptic peptides of iso1-SMARCA2^{BD} in the APO, Binary with SiTX-0038404 (PROTAC 1), SiTX-0038405(PROTAC 2), SiTX-0038406(ACBI1) or Ternary complex with 404, 405, 406 + VCB.

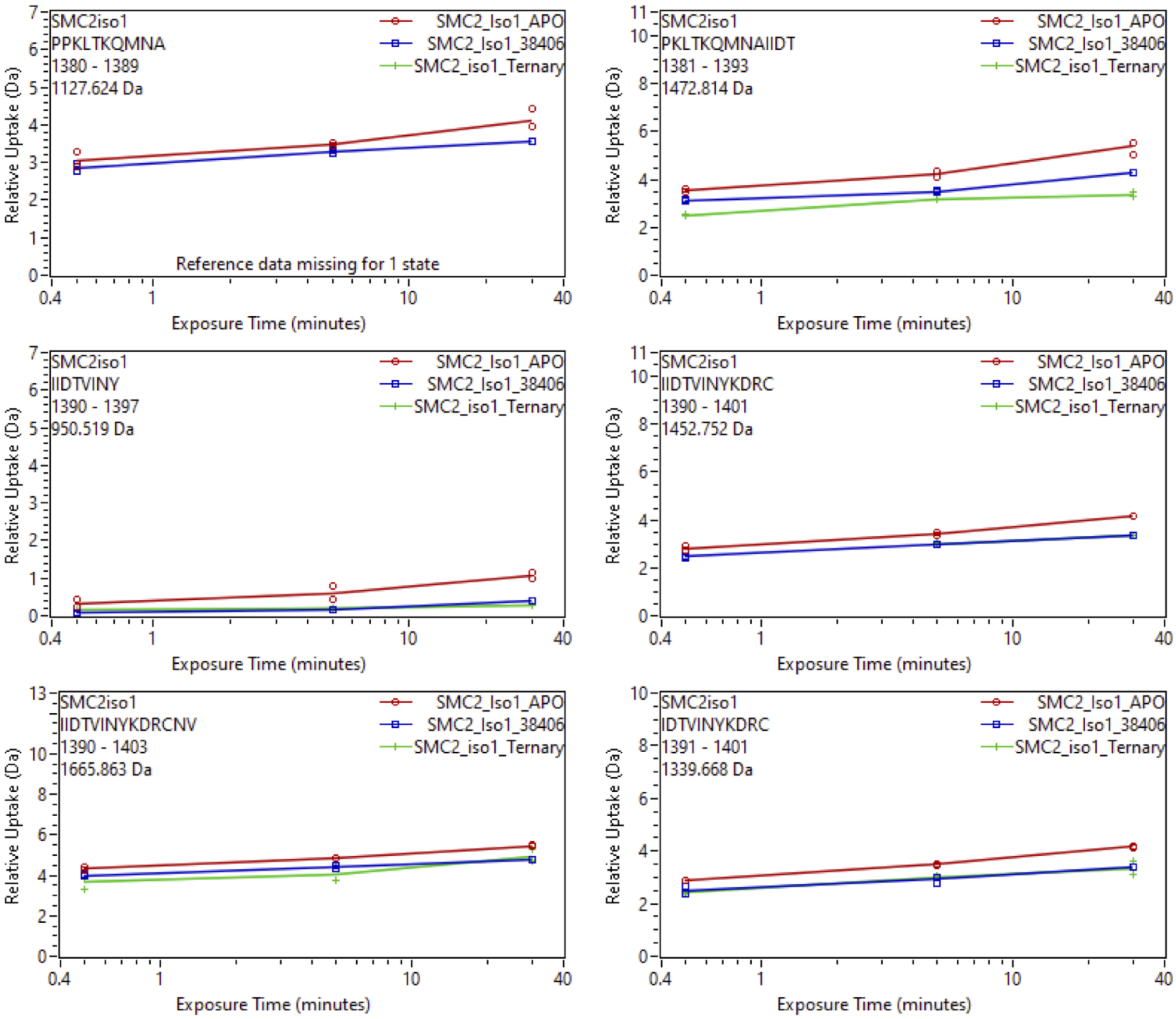

Relative deuterium uptake plots of peptic peptides of iso1-SMARCA2^{BD} in the APO, Binary with SiTX-0038404 (PROTAC 1), SiTX-0038405(PROTAC 2), SiTX-0038406(ACBI1) or Ternary complex with 404, 405, 406 + VCB.

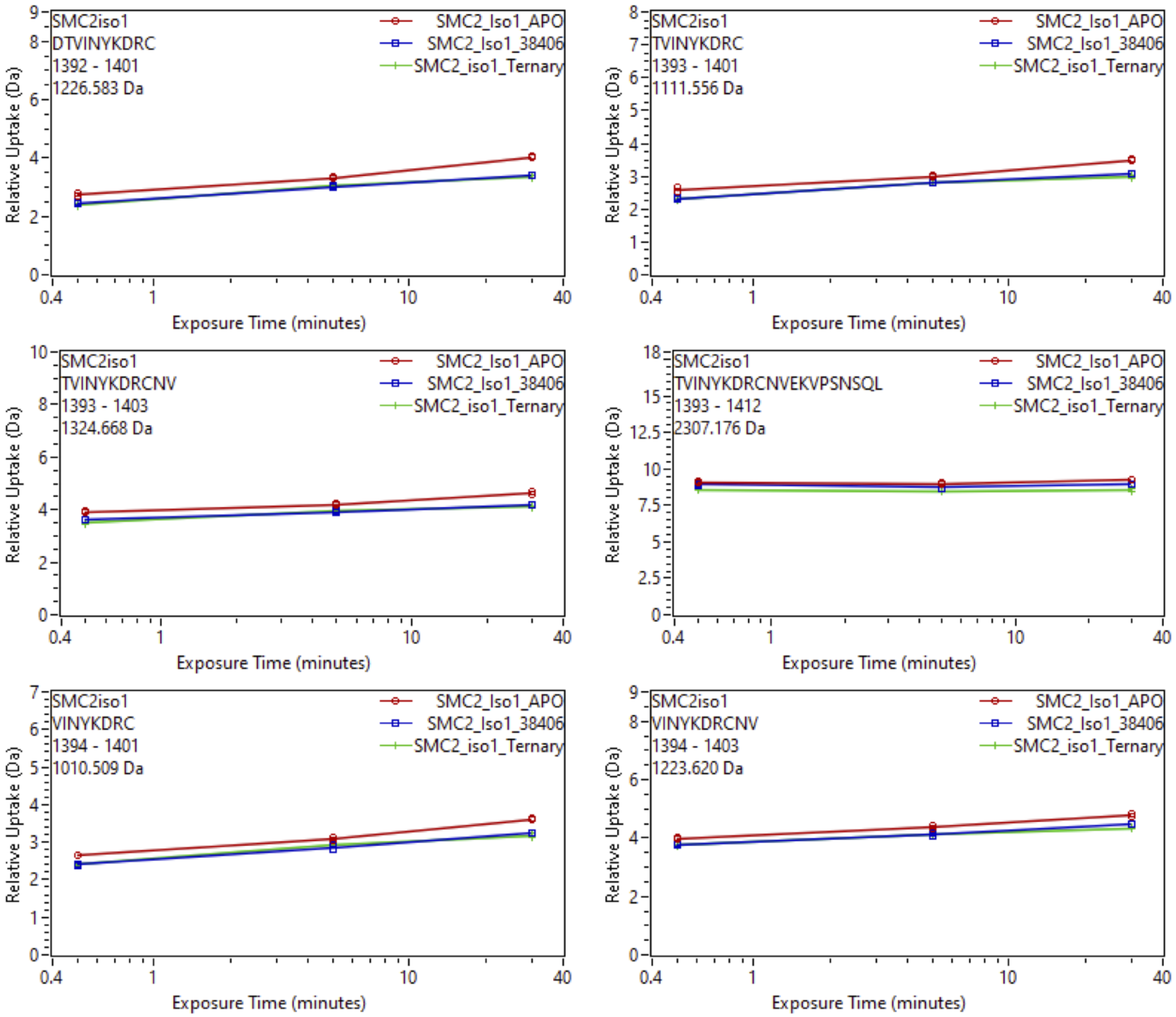

Relative deuterium uptake plots of peptic peptides of iso1-SMARCA2^{BD} in the APO, Binary with SiTX-0038404 (PROTAC 1), SiTX-0038405(PROTAC 2), SiTX-0038406(ACBI1) or Ternary complex with 404, 405, 406 + VCB.

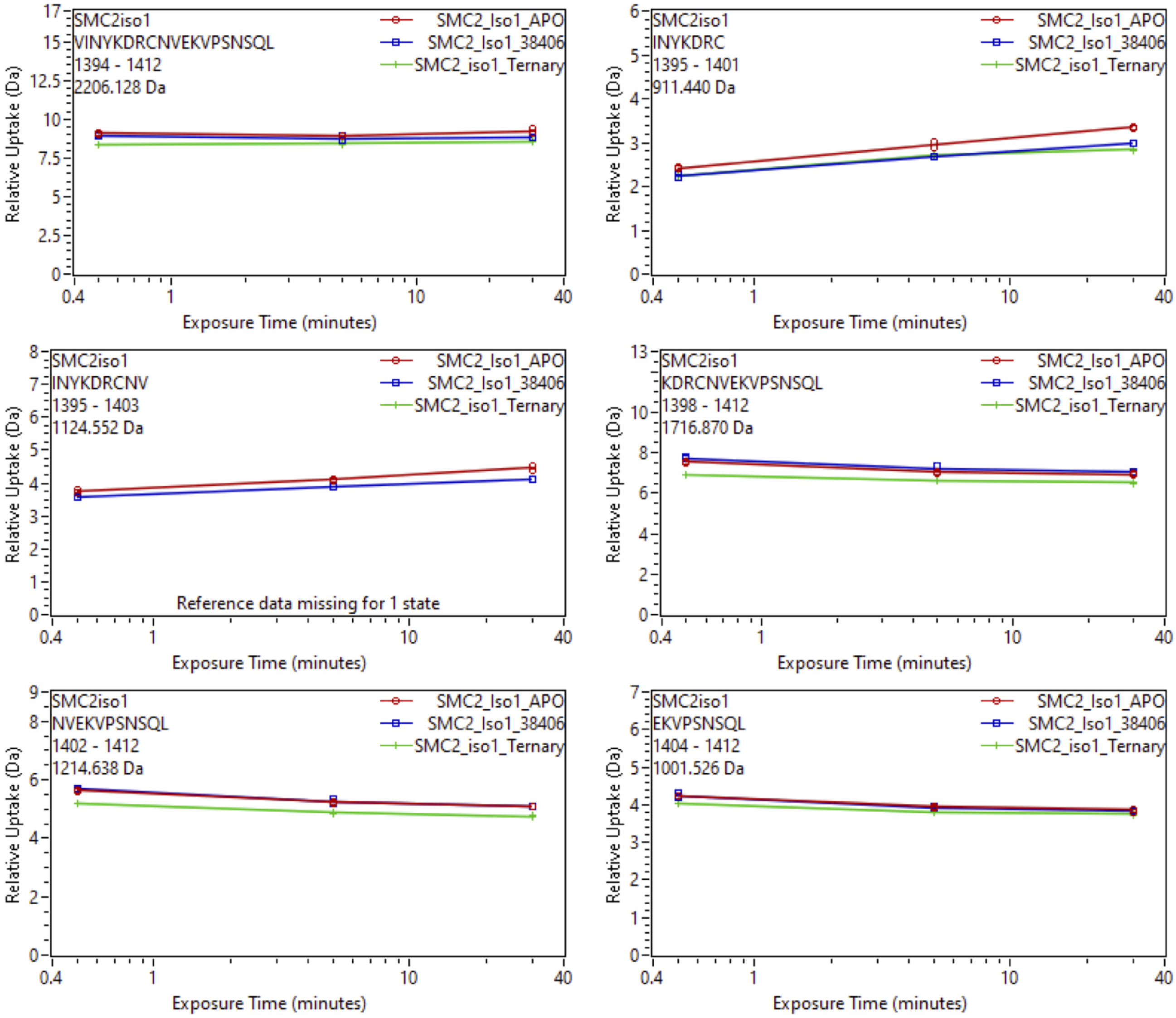

Relative deuterium uptake plots of peptic peptides of iso1-SMARCA2^{BD} in the APO, Binary with SiTX-0038404 (PROTAC 1), SiTX-0038405(PROTAC 2), SiTX-0038406(ACBI1) or Ternary complex with 404, 405, 406 + VCB.

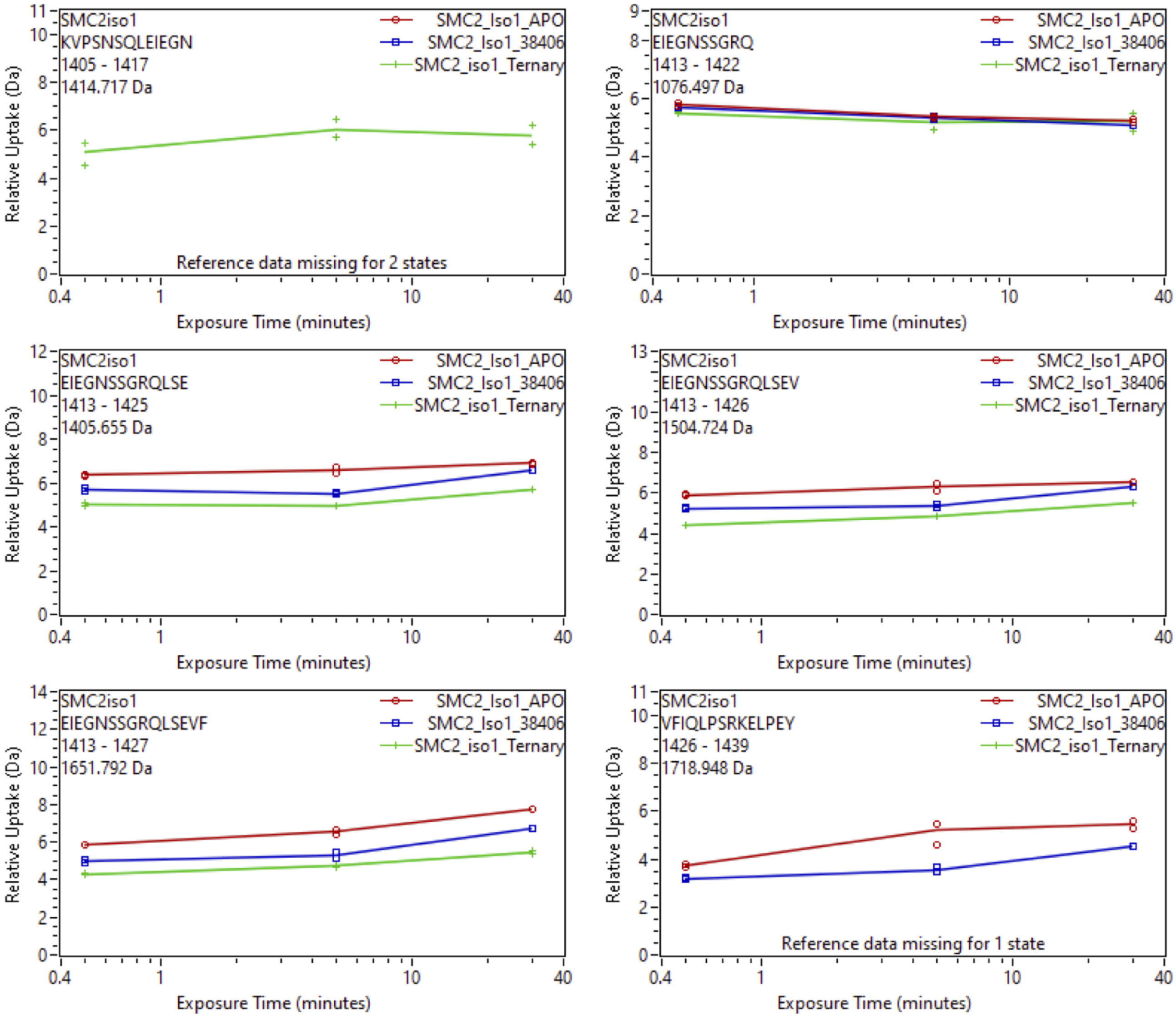

Relative deuterium uptake plots of peptic peptides of iso1-SMARCA2^{BD} in the APO, Binary with SiTX-0038404 (PROTAC 1), SiTX-0038405(PROTAC 2), SiTX-0038406(ACBI1) or Ternary complex with 404, 405, 406 + VCB.

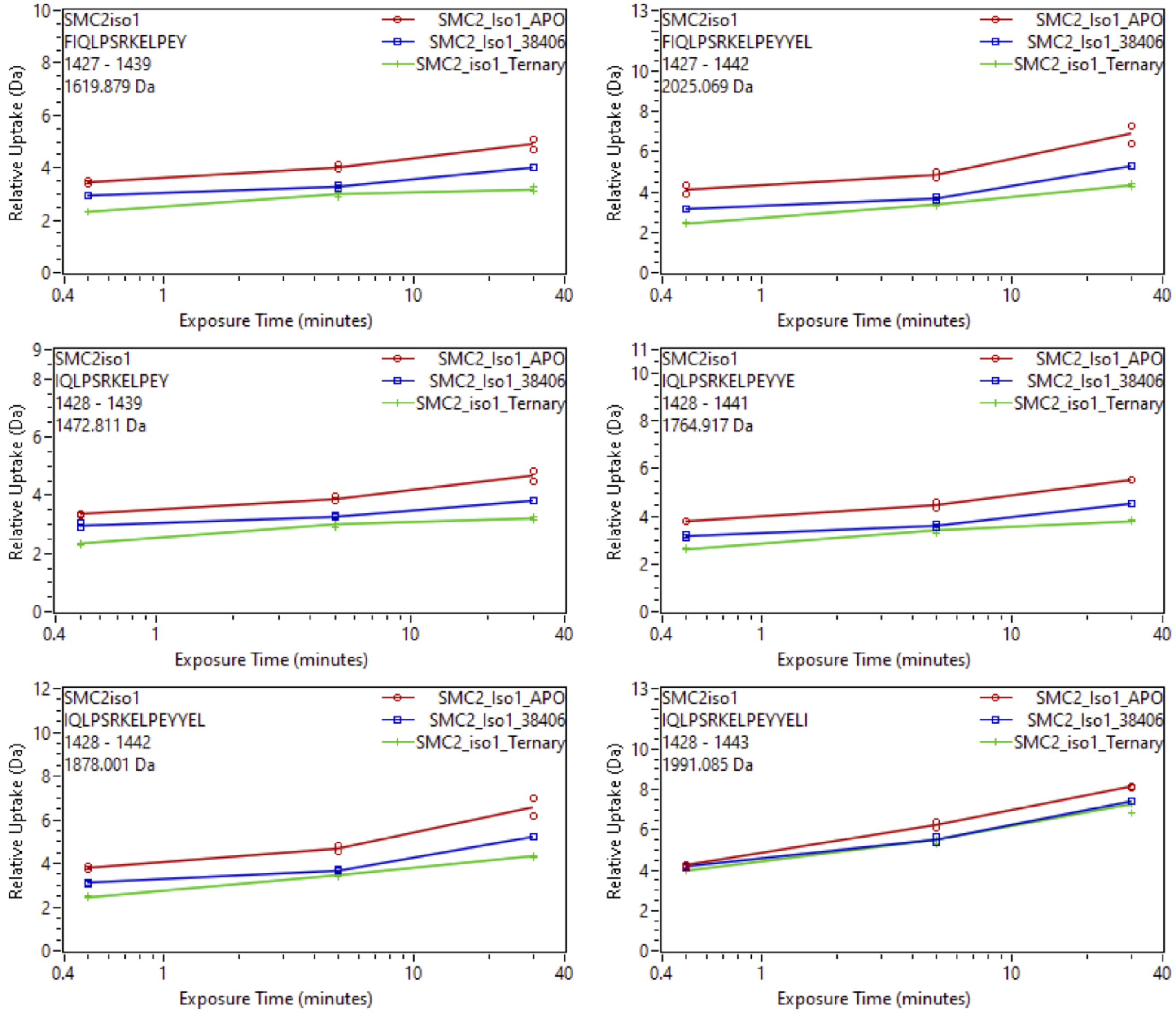

Relative deuterium uptake plots of peptic peptides of iso1-SMARCA2^{BD} in the APO, Binary with SiTX-0038404 (PROTAC 1), SiTX-0038405(PROTAC 2), SiTX-0038406(ACBI1) or Ternary complex with 404, 405, 406 + VCB.

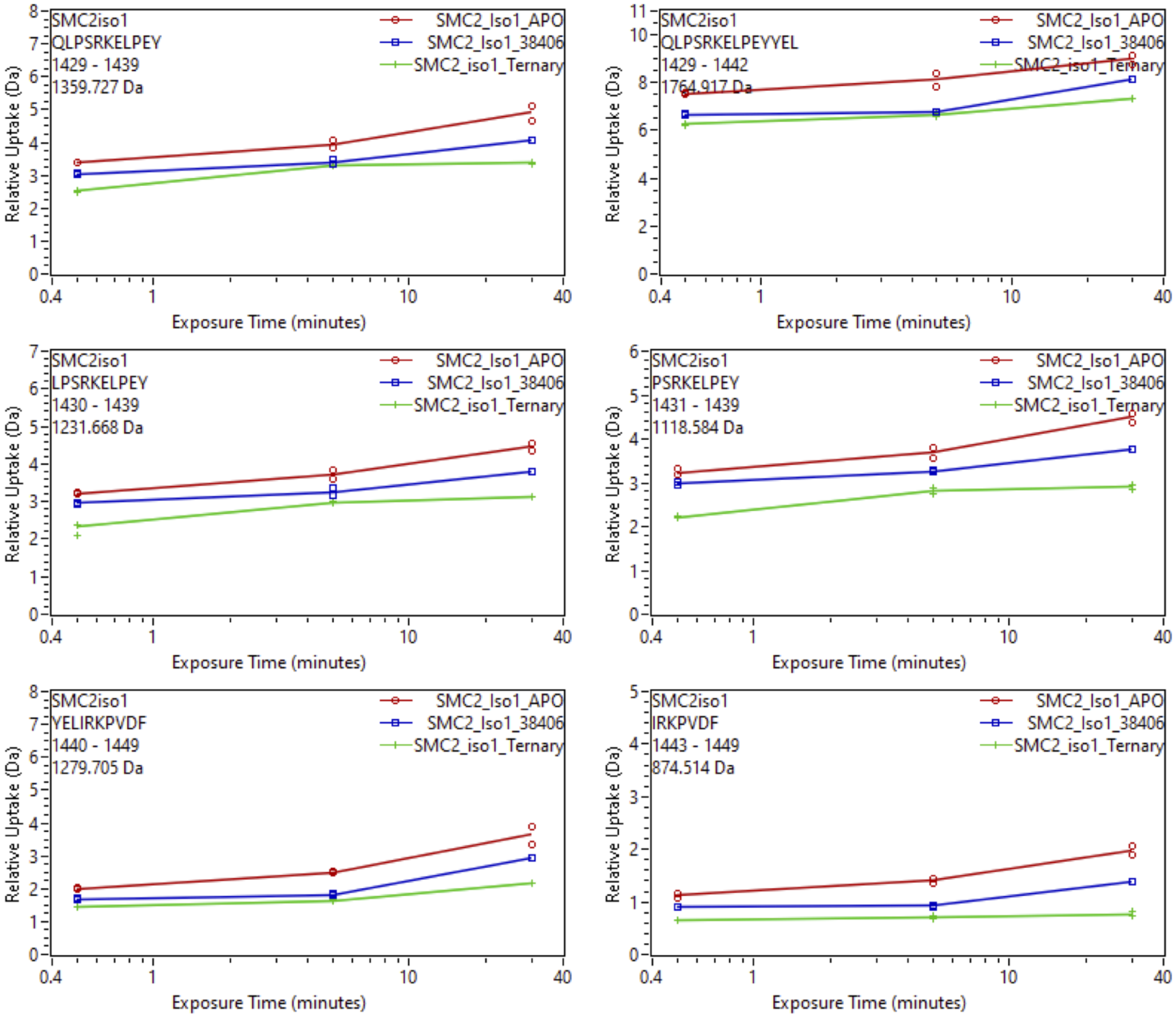

Relative deuterium uptake plots of peptic peptides of iso1-SMARCA2^{BD} in the APO, Binary with SiTX-0038404 (PROTAC 1), SiTX-0038405(PROTAC 2), SiTX-0038406(ACBI1) or Ternary complex with 404, 405, 406 + VCB.

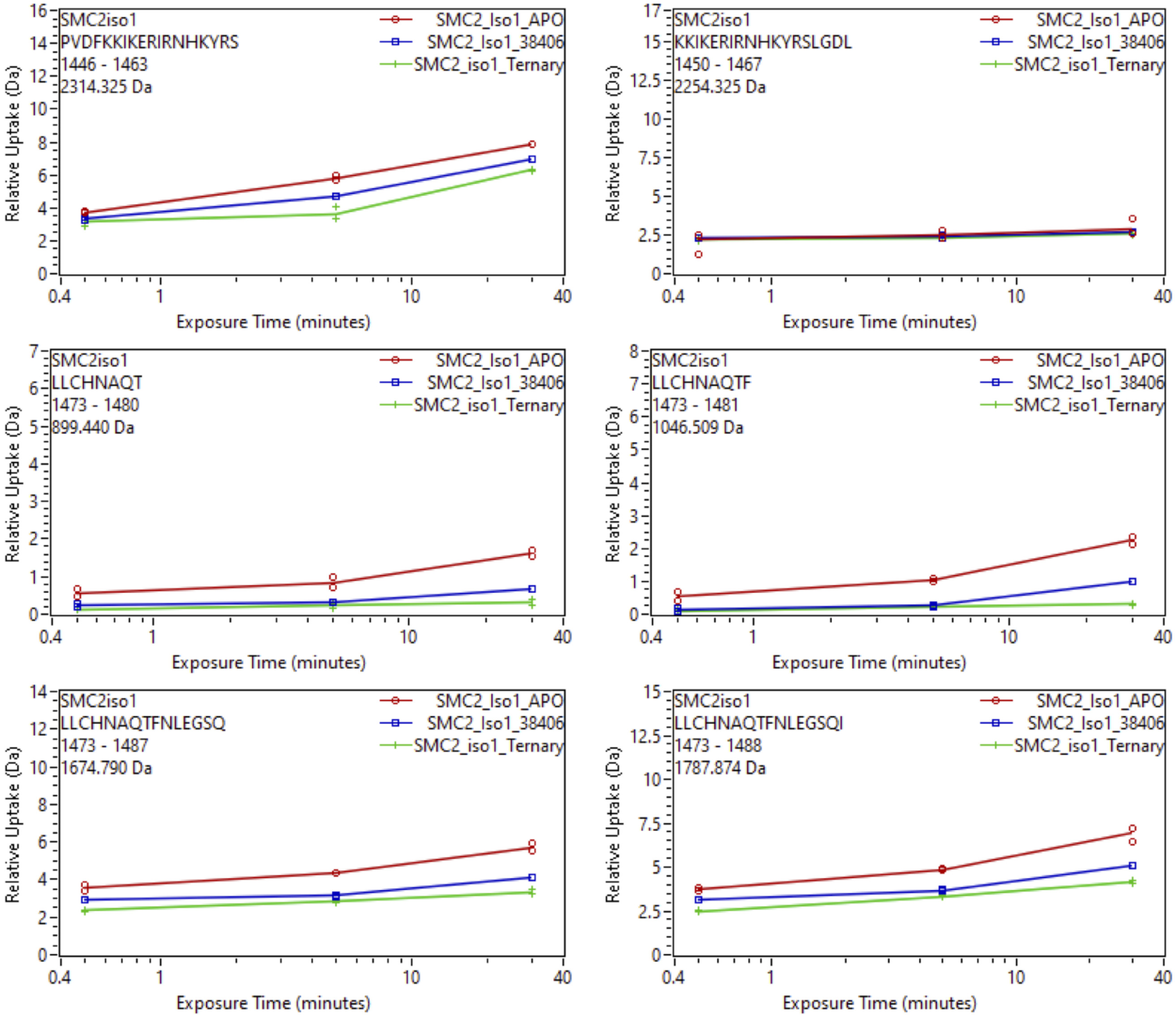

Relative deuterium uptake plots of peptic peptides of iso1-SMARCA2^{BD} in the APO, Binary with SiTX-0038404 (PROTAC 1), SiTX-0038405(PROTAC 2), SiTX-0038406(ACBI1) or Ternary complex with 404, 405, 406 + VCB.

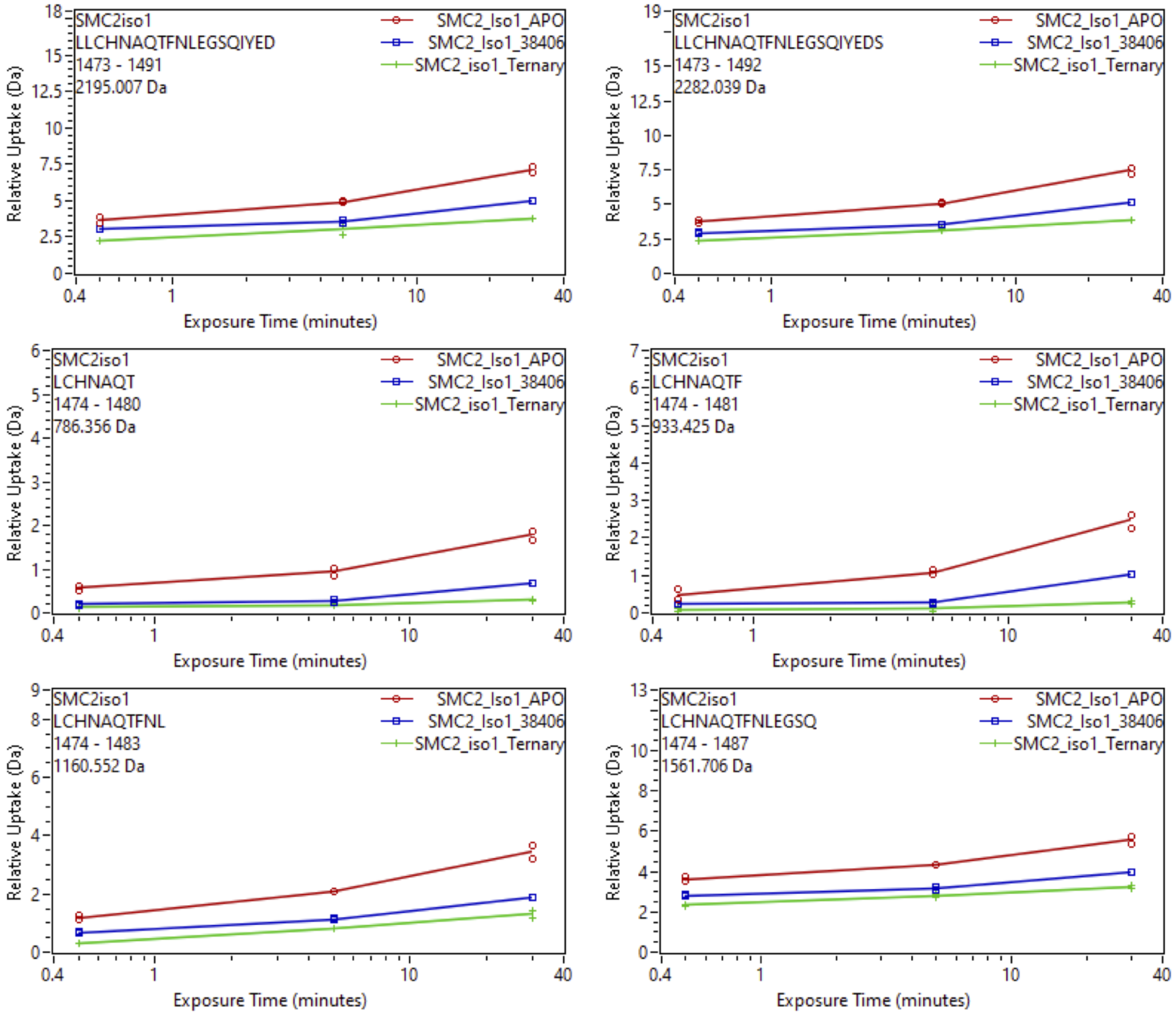

Relative deuterium uptake plots of peptic peptides of iso1-SMARCA2^{BD} in the APO, Binary with SiTX-0038404 (PROTAC 1), SiTX-0038405(PROTAC 2), SiTX-0038406(ACBI1) or Ternary complex with 404, 405, 406 + VCB.

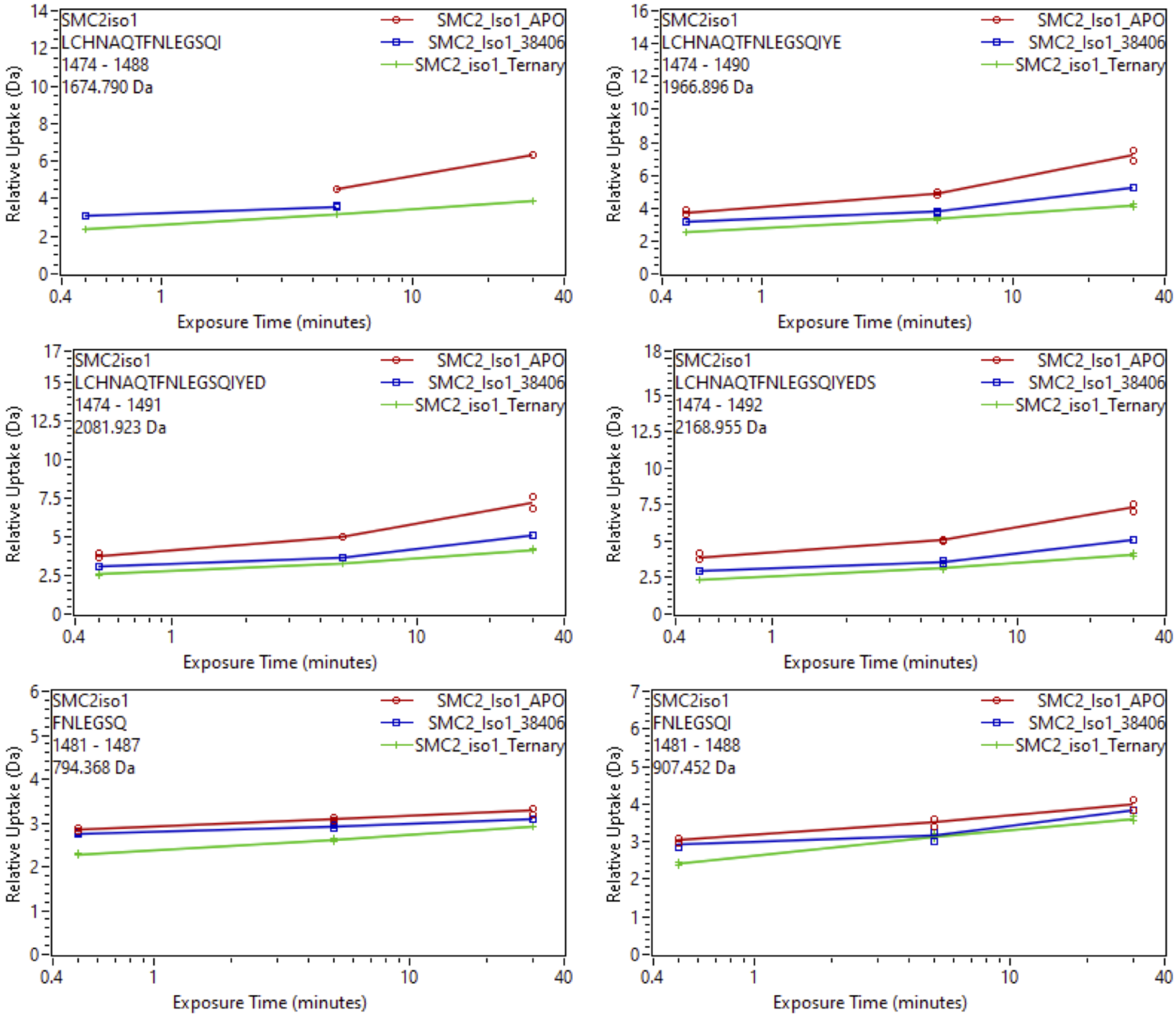

Relative deuterium uptake plots of peptic peptides of iso1-SMARCA2^{BD} in the APO, Binary with SiTX-0038404 (PROTAC 1), SiTX-0038405(PROTAC 2), SiTX-0038406(ACBI1) or Ternary complex with 404, 405, 406 + VCB.

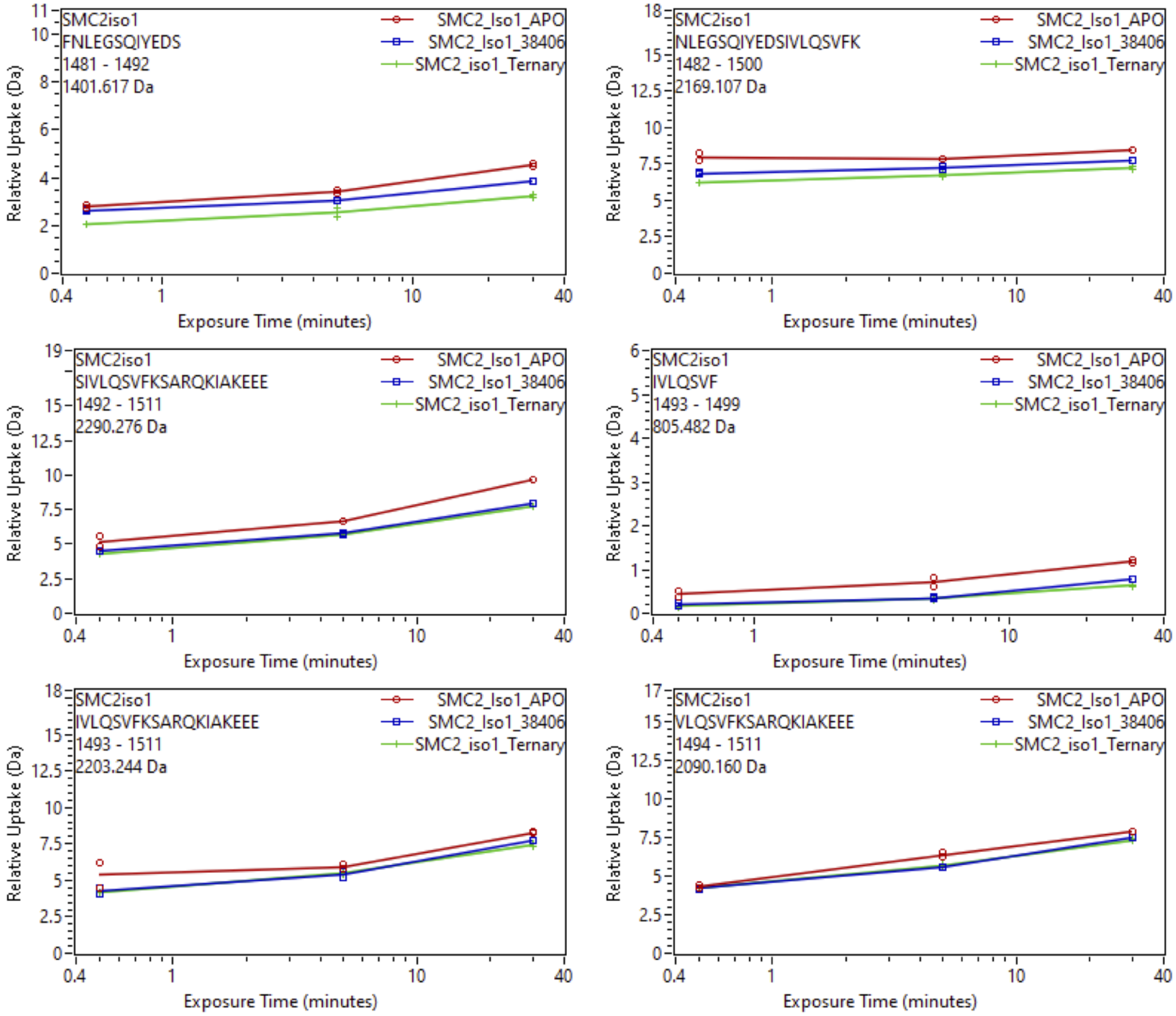

Relative deuterium uptake plots of peptic peptides of iso1-SMARCA2^{BD} in the APO, Binary with SiTX-0038404 (PROTAC 1), SiTX-0038405(PROTAC 2), SiTX-0038406(ACBI1) or Ternary complex with 404, 405, 406 + VCB.

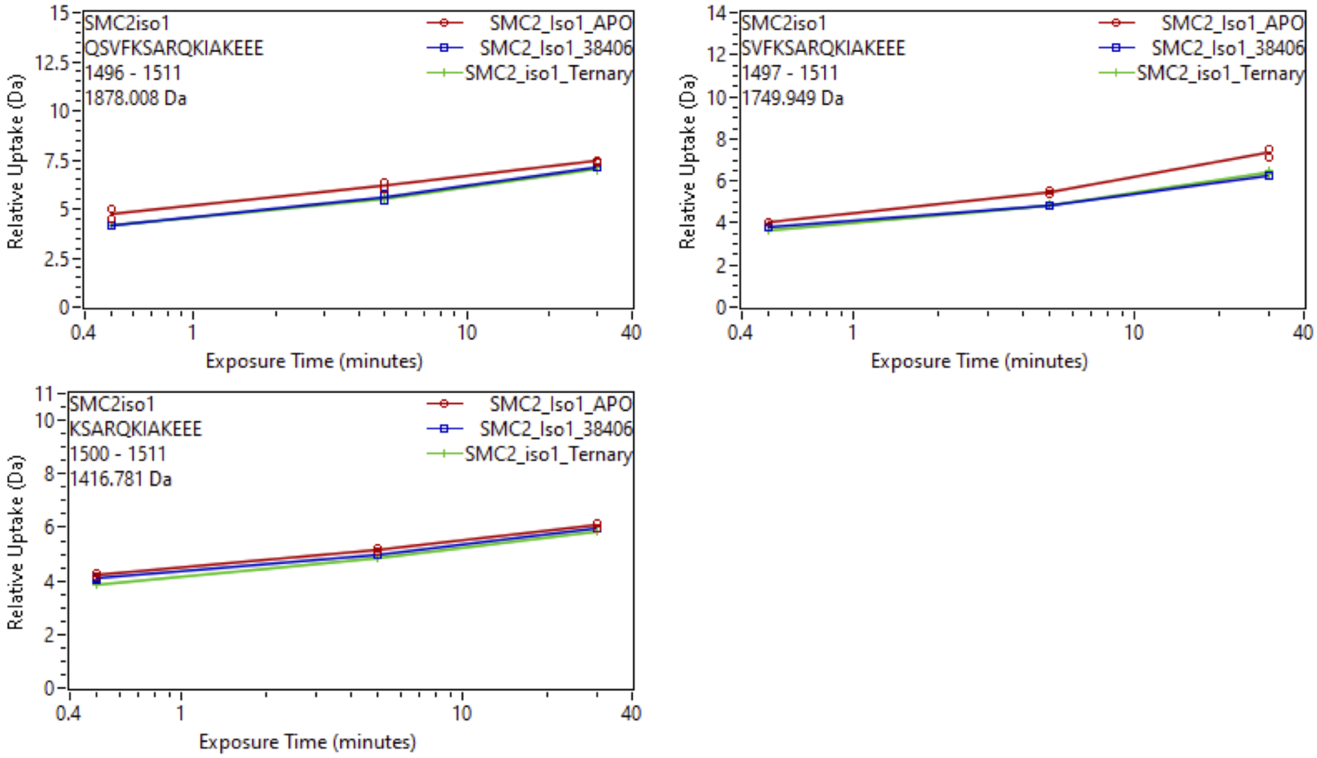

Relative deuterium uptake plots of peptic peptides of iso1-SMARCA2^{BD} in the APO, Binary with SiTX-0038404 (PROTAC 1), SiTX-0038405(PROTAC 2), SiTX-0038406(ACBI1) or Ternary complex with 404, 405, 406 + VCB.
