# Supplementary material for: Predicting the structural basis of targeted protein degradation by integrating molecular dynamics simulations with structural mass spectrometry
Source: Nat Commun. 2022 Oct 6;13:5884. doi: 10.1038/s41467-022-33575-4 (PMC9537307; doi:10.1038/s41467-022-33575-4)
Supplement: Supplementary file 5 — Reporting Summary [file 41467_2022_33575_MOESM5_ESM.pdf]

nature portfolio

Corresponding author(s): Alex Dickson
Huafeng Xu
Woody Sherman
Jesus A. Izaguirre

Last updated by author(s): Aug 24, 2022

Reporting Summary

Nature Portfolio wishes to improve the reproducibility of the work that we publish. This form provides structure for consistency and transparency in reporting. For further information on Nature Portfolio policies, see our Editorial Policies and the Editorial Policy Checklist.

Statistics

For all statistical analyses, confirm that the following items are present in the figure legend, table legend, main text, or Methods section.

n/a	Confirmed	
☐	☒	The exact sample size (n) for each experimental group/condition, given as a discrete number and unit of measurement
☐	☒	A statement on whether measurements were taken from distinct samples or whether the same sample was measured repeatedly
☐	☒	The statistical test(s) used AND whether they are one- or two-sided<br>*Only common tests should be described solely by name; describe more complex techniques in the Methods section.*
☒	☐	A description of all covariates tested
☒	☐	A description of any assumptions or corrections, such as tests of normality and adjustment for multiple comparisons
☐	☒	A full description of the statistical parameters including central tendency (e.g. means) or other basic estimates (e.g. regression coefficient) AND variation (e.g. standard deviation) or associated estimates of uncertainty (e.g. confidence intervals)
☐	☒	For null hypothesis testing, the test statistic (e.g. F, t, r) with confidence intervals, effect sizes, degrees of freedom and P value noted<br>*Give P values as exact values whenever suitable.*
☒	☐	For Bayesian analysis, information on the choice of priors and Markov chain Monte Carlo settings
☒	☐	For hierarchical and complex designs, identification of the appropriate level for tests and full reporting of outcomes
☒	☐	Estimates of effect sizes (e.g. Cohen's d, Pearson's r), indicating how they were calculated

Our web collection on statistics for biologists contains articles on many of the points above.

Software and code

Policy information about availability of computer code

Data collection	AMBER v.20 (molecular dynamics simulation software)<br>GROMACS v.2018.8 (molecular dynamics simulation software)<br>OpenMM v.7.5.0. (molecular dynamics simulation software)
Data analysis	CCP4 8.0.004 (crystallography analysis software)<br>DynamX version 3.0 (HDX data analysis software)<br>BioXTAS RAW 2.0.3 (SAXS data analysis software)<br>CRYSOL 3.0 (SAXS data analysis software)<br>YSARA Model (molecular modeling software)<br>RosettaDock (molecular docking software)<br>VMD v. 1.9.3. (molecular visualization and analysis software<br>PyMOL v. 2.5. (molecular visualization and analysis software)

For manuscripts utilizing custom algorithms or software that are central to the research but not yet described in published literature, software must be made available to editors and reviewers. We strongly encourage code deposition in a community repository (e.g. GitHub). See the Nature Portfolio guidelines for submitting code & software for further information.

Data

Policy information about availability of data

All manuscripts must include a data availability statement. This statement should provide the following information, where applicable:

- Accession codes, unique identifiers, or web links for publicly available datasets
- A description of any restrictions on data availability
- For clinical datasets or third party data, please ensure that the statement adheres to our policy

Data availability:

All data presented in this study as main text or Supplementary Figures and Tables are provided in the Source Data file.

The crystal structure of the ternary complex SMARCA2BD:ACBI1:VHL resolved in this study has been deposited in the Protein Data Bank under accession code 7S4E [https://www.rcsb.org/structure/7S4E]. (Note that this deposition is held for release and will be assigned a digital object identifier upon publication of this study.)

The proteomics data obtained in this study as part of the HDX-MS experiments have been deposited to the ProteomeXchange Consortium via the PRIDE partner repository under accession code PXD033849 [http://proteomecentral.proteomexchange.org/cgi/GetDataset?ID=PXD033849].

The proteomics data obtained in this study as part of the ubiquitinomics experiments have been deposited to the ProteomeXchange Consortium via the PRIDE partner repository under accession code PXD033763 [http://proteomecentral.proteomexchange.org/cgi/GetDataset?ID=PXD033763].

The small-angle X-ray scattering data obtained in this study for the ternary complex iso1-SMARCA2^BD:ACBI1:VCB has been deposited in the Small Angle Scattering Biological Data Bank under accession code SASDPE8 [https://www.sasbdb.org/data/SASDPE8]. (Note that this deposition is held for release upon publication of this study.)

The small-angle X-ray scattering data obtained in this study for the ternary complex iso2-SMARCA2^BD:ACBI1:VCB has been deposited in the Small Angle Scattering Biological Data Bank under accession code SASDPF8 [https://www.sasbdb.org/data/SASDPF8]. (Note that this deposition is held for release upon publication of this study.)

The crystal structure of the ternary complex SMARCA2BD:PROTAC 1:VHL, used in this study for comparison and for simulation, is available in the Protein Data Bank under accession code 6HAY [http://doi.org/10.2210/pdb6HAY/pdb].

The crystal structure of the ternary complex SMARCA2BD:PROTAC 2:VHL, used in this study for comparison and for simulation, is available in the Protein Data Bank under accession code 6HAX [https://doi.org/10.2210/pdb6HAX/pdb].

The crystal structure of the NEDD8 protein, used in this study for simulation, is available in the Protein Data Bank under accession code 6TTU [https://www.rcsb.org/structure/6TTU].

The crystal structures of the Cullin2 and RBX1 proteins, used in this study for simulation, are available in the Protein Data Bank under accession code 5N4W [https://www.rcsb.org/structure/5N4W].

The crystal structures of the VHL, Elongin C, and Elongin B proteins, used in this study for simulation, are available in the Protein Data Bank under accession code 1LQB [https://www.rcsb.org/structure/1LQB].

The gene sequence of the bromodomain of SMARCA2 (isoform 1), used in this study for cloning and protein expression as well as for homology modeling, is available in the Uniprot database under accession code P51531-1 [https://www.uniprot.org/uniprotkb/P51531-1].

The gene sequence of the bromodomain of SMARCA2 (isoform 2), used in this study for cloning and protein expression, is available in the Uniprot database under accession code P51531-2 [https://www.uniprot.org/uniprotkb/P51531-2].

The gene sequence of VHL, used in this study for cloning and protein expression, is available in the Uniprot database under accession code P40337 [https://www.uniprot.org/uniprotkb/P40337].

The gene sequence of EloC, used in this study for cloning and protein expression, is available in the Uniprot database under accession code Q15369 [https://www.uniprot.org/uniprotkb/Q15369].

The gene sequence of EloB, used in this study for cloning and protein expression, is available in the

Uniprot database under accession code Q15370 [https://www.uniprot.org/uniprotkb/Q15370].

The initial and final configurations of all Molecular Dynamics simulation trajectories performed in this study can be accessed at http://doi.org/10.5281/zenodo.7017263.

Full-length simulation trajectories can be accessed at https://console.cloud.google.com/storage/browser/paperdata

Human research participants

Policy information about studies involving human research participants and Sex and Gender in Research.

Reporting on sex and gender	N/A
Population characteristics	N/A
Recruitment	N/A
Ethics oversight	N/A

Note that full information on the approval of the study protocol must also be provided in the manuscript.

Field-specific reporting

Please select the one below that is the best fit for your research. If you are not sure, read the appropriate sections before making your selection.

- [x] Life sciences
- [ ] Behavioural & social sciences
- [ ] Ecological, evolutionary & environmental sciences

For a reference copy of the document with all sections, see nature.com/documents/nr-reporting-summary-flat.pdf

Life sciences study design

All studies must disclose on these points even when the disclosure is negative.

Sample size

Experimental sample sizes were not predetermined.

In the crystallography experiment, the beam of X radiation is diffracted from a crystal that contains many copies of the purified ternary complex in a symmetric lattice. Reflected beams from multiple crystal orientations are detected and recorded, which leads to a large, undetermined, sample size in a diffraction pattern.

Both HDX-MS and SAXS are solution-phase experimental techniques, i.e., an undetermined, large number of molecules are sampled. SAXS was performed on distinct frames of the SEC elution that correspond to the ternary complex (12 frames for iso1-SMARCA2:ACBI1:VCB and 14 frames for iso2-SMARCA2:ACBI1:VCB; see Supplementary Figure 15).

The ubiquitination of SMARCA2 on Hela cells was evaluated on >12,500 ubiquitination sites on >5300 proteins (see Supplementary Data 2).

All simulation trajectory sizes are determined.

WE-HDX simulation trajectories were each 96,000 frames in length.

The HREMD simulationsm at the lowest-rank replica (i.e. unscaled Hamiltonian), consisted of 100,001 frames.

Folding@home simulation trajectories consisted of 1,114,000 frames on aggregate spread over 9,800 trajectory files (in total for 100 distinct runs).

Data exclusions

Among the experiments, the SEC elution peaks not corresponding to the ternary complex were excluded (see Supplementary Figure 15).

In the WE-HDX simulations, any frames with I-RMSD > 2 Å were excluded for the rate constant calculation (Figure 3c) and, in addition, those with <30 contacts were excluded for the performance statistics shown in Figure 5.

The Folding@home simulations were analyzed at a frame rate of 1/(5 ns), i.e., any sampled structures in between were excluded.

Replication

The HDX-MS were performed twice; all other experiments were performed once.

All simulations were performed in multiple independent runs.

WE-HDX simulations were performed 7-fold for SMARCA2^BD:PROTAC 2:VHL and 3-fold for both SMARCA2^BD:PROTAC 1:VHL and SMARCA2^BD:ACBI1:VHL.

	HREMD simulations were performed for 8 distinct systems, each of which consisted of 20-24 replicas (see Supplementary Table 13). Only the lowest-rank replica (i.e. unscaled Hamiltonian) was analyzed (100,001 frames).<br><br>Brute-force MD simulations on Folding@home were performed 100-fold.
Randomization	Local randomization is inherent to all the experiments performed, due to the nature of crystal formation and the solution-phase scattering, deuterium-exchange, or ubiquitination.<br><br>Randomization in the MD simulations is achieved implicitly by the Langevin thermostat that applies a random force to simulate particle friction. In particular, in the WE-HDX simulations, the merging and pruning algorithm applied on the simulation walkers leads to a random sampling effect within the given simulation. In the HREMD simulations, the neighboring replicas exchange when their difference in Boltzmann factors is small which will lead to a random walk in Hamiltonian space.
Blinding	The inherent randomization in these controlled experiments and simulations described above minimizes the experimental bias and thus the need for blinding. The experiments are based on signal detection and automated data evaluation and thus are not subject to confirmation bias.<br><br>Similarly, the molecular simulations are based on interaction energy functions derived from first principles and tested against experiments, thus yielding results that are not biased by human expectation.

Reporting for specific materials, systems and methods

We require information from authors about some types of materials, experimental systems and methods used in many studies. Here, indicate whether each material, system or method listed is relevant to your study. If you are not sure if a list item applies to your research, read the appropriate section before selecting a response.

Materials & experimental systems

n/a	Involved in the study
☐	☒ Antibodies
☐	☒ Eukaryotic cell lines
☒	☐ Palaeontology and archaeology
☒	☐ Animals and other organisms
☒	☐ Clinical data
☒	☐ Dual use research of concern

Methods

n/a	Involved in the study
☒	☐ ChIP-seq
☒	☐ Flow cytometry
☒	☐ MRI-based neuroimaging

Antibodies

Antibodies used	Ubiquitin anti-diGly remnant antibodies were used from Cell Signaling Technology
Validation	Cell Signaling Technology (CST) has validated the antibodies as described on their website:<br>https://www.cellsignal.com/about-us/cst-antibody-validation-principles<br>In particular, CST adapted the work by Uhlen, et. al., ("A Proposal for Validation of Antibodies." Nature Methods (2016)) to build the Hallmarks of Antibody Validation,

Eukaryotic cell lines

Policy information about cell lines and Sex and Gender in Research

Cell line source(s)	Hela cells, aquired from ATCC, were cultured in this study.
Authentication	The cell lines were authenticated:<br>https://www.atcc.org/the-science/authentication
Mycoplasma contamination	The cell lines were tested for mycoplasma contamination:<br>https://www.atcc.org/the-science/authentication/mycoplasma-contamination
Commonly misidentified lines<br>(See ICLAC register)	No commonly misidentified cell lines were used in this study.
